# Investigation of naked oat (*Avena sativa* ssp. *nuda*) domestication provides insights into yield and nutritional gaps in oats

**DOI:** 10.1038/s42003-026-10626-w

**Published:** 2026-07-11

**Authors:** Xiaolong Dong, Kaiquan Yu, Zilin Zhao, Wubishet A. Bekele, Qinkun Li, Laichun Guo, Xiaotian Liang, Xinyi Zhang, Chunlong Wang, Yongjie Zhang, Xingjia Zhang, Yuzhen Zhang, Ruizhen Yang, Changzhong Ren, Yuanying Peng

**Affiliations:** 1 State Key Laboratory of Crop Gene Exploration and Utilization in Southwest China, Sichuan Agricultural University, Chengdu, China; 2 Triticeae Research Institute, Sichuan Agricultural University, Chengdu, China; 3 Ottawa Research and Development Centre, Agriculture and Agri-Food Canada, Ottawa, ON Canada; 4 National Oat Improvement Center, Baicheng Academy of Agricultural Sciences, Baicheng, China

**Keywords:** Plant breeding, Genetic markers

## Abstract

Oats are valuable for global food and forage security due to their excellent nutrition and polyploidy. Cultivated oats (*Avena sativa* L.) are categorized into hulled and naked types based on lemma morphology, and naked oats possess superior processing and nutritional quality. Here, we decipher the genetic architecture of 666 globally collected oat accessions via population structure, genetic diversity and gene flow analyses, revealing genetic admixture and lineage differentiation driven by geographic isolation and divergent selection. Population evolutionary analyses indicate that Chinese naked oat landraces diverge from the Central and Eastern Eurasian hulled oats 6124 generations ago and undergo secondary domestication in North China under strong artificial selection. Multi-environment phenotyping of 19 traits from 2010 to 2021 reveals that hulled oats outperform in grain size and yield, while naked oats have higher crude protein (+25.5%) and crude fat (+16.9%). Genome-wide scans identify 883 directional selection genes related to nutrient metabolism, whereas balancing selection on carbon metabolism genes restricts yield potential. Furthermore, integrated GWAS identify three favorable haplotypes associated with favorable agronomic and quality traits, providing key genetic resources for oat molecular breeding.

## Abstract (web summary)

Analysis of the genetic architecture of globally collected oat accessions reveals transcontinental gene flow and divergent domestication, with nutritionally superior naked oats evolving from high-yielding hulled oats.

## Introduction

While natural selection drives evolution by favoring traits that enhance reproductive success and environmental adaptation, cultivated crops acquire production-oriented characteristics through targeted artificial selection during domestication^1^. Crucially, genomic signatures of artificial selection exhibit robust correlations with key agronomic traits including yield, quality, and stress resilience^1,2^. Amidst escalating challenges of climate volatility and population-driven food insecurity, germplasm innovation emerges as the cornerstone of sustainable agriculture, wherein identifying superior allelic variants and developing transformative varieties constitute pivotal strategies for resilience.

Cultivated oat (*Avena sativa* L.), a nutritionally rich allohexaploid crop, serves dual grain-forage functions^3,4^. Despite historically ranking as the world’s eighth-largest cereal, its complex allohexaploid genome has constrained genomic tool innovation and application^5^. Research on yield and quality improvement in oats lags behind that of staple cereals like rice and wheat, resulting in a narrow genetic base of cultivated oat varieties and sluggish per-hectare yield growth^6,7^. Oats diverge into globally dominant hulled oats and China-predominant naked oats. In hulled oats, the caryopsis is tightly enclosed by a rigid lemma that remains attached after threshing, providing physical protection but necessitating dehulling prior to utilization. By contrast, in naked oats, the lemma detaches freely from the mature grain, as in wheat, permitting direct utilization but rendering the grain more susceptible to mechanical damage^8^. Meanwhile, naked oats exhibit superior nutritional profiles aligned with modern health demands, including elevated protein, balanced amino acids, and unsaturated fatty acids^9^. Crucially, their yield deficit versus hulled oats^10^ establishes the yield-quality trade-off as the central genetic improvement challenge.

High-throughput sequencing has enabled identification of oat quantitative trait loci (QTLs) associated with traits like β-glucan content and disease resistance^11–14^. Recently, the release of multiple high-quality reference genomes has laid the groundwork for deciphering the genetic architecture of oat yield, quality and adaptation^15–21^. While systematic genetic frameworks for naked grain traits have been established in sorghum, wheat, and teosinte *(Zea mays* ssp. *parviglumis*)^22–24^, parallel research in oats remains limited. Regarding the origin of naked oats, Loskutov et al.^25^ first proposed the “Chinese Mutation Hypothesis” that naked oats originated from hulled oat mutants, but this lacks genetic evidence. Recently, Nan et al. ^26^ inferred naked independent domestication via divergence dating, and Bekele et al. ^27^ inferred the potential independent domestication origin of Chinese naked oats from the haplotype distribution of a large-scale inversion on chr7D. While Li et al. ^21^, based on subpopulation *F*_ST_ values, suggested naked oats derived from European hulled oats, identifying genomic differentiation evidence via SNPs and SVs. However, limited landrace materials and methodologies have hindered systematic characterization of naked oats’ domestication and dispersal. Additionally, the lack of integrated analysis between genomic differentiation and multi-environment phenotypic data restricts understanding of the genomic basis for phenotypic divergence, thereby limiting the application of superior haplotypes in breeding.

To address the genetic origin and phenotypic trait divergence between hulled and naked oats, this study leverages 127,563 high-quality genome-wide SNPs to systematically dissect the population structure, genetic differentiation, and domestication history of 666 hexaploid oat accessions (659 with previously published GBS data^15^; 7 newly generated in this study) from 65 countries. By comparing population differences among various subpopulations, particularly between accessions from China (the center of diversity for naked oats) and those from other geographical regions, we revealed the differentiation characteristics of hulled and naked oats with respect to genetic structure, diversity distribution, gene flow events, and domestication pathways. Furthermore, we integrated phenotypic datasets of 19 agronomic and grain quality traits evaluated across multiple environments from 2010–2021 to clarify phenotypic divergence and breeding selection preferences between hulled and naked oats. By combining selection signatures during naked oat improvement with RNA-seq data from globally representative accessions, we aimed to elucidate the genetic basis of the nutrition-yield trade-off, identify candidate genes, and establish a foundation for nutrition-oriented oat breeding.

## Results

### Population structure and differentiation

**Fig. 1:**
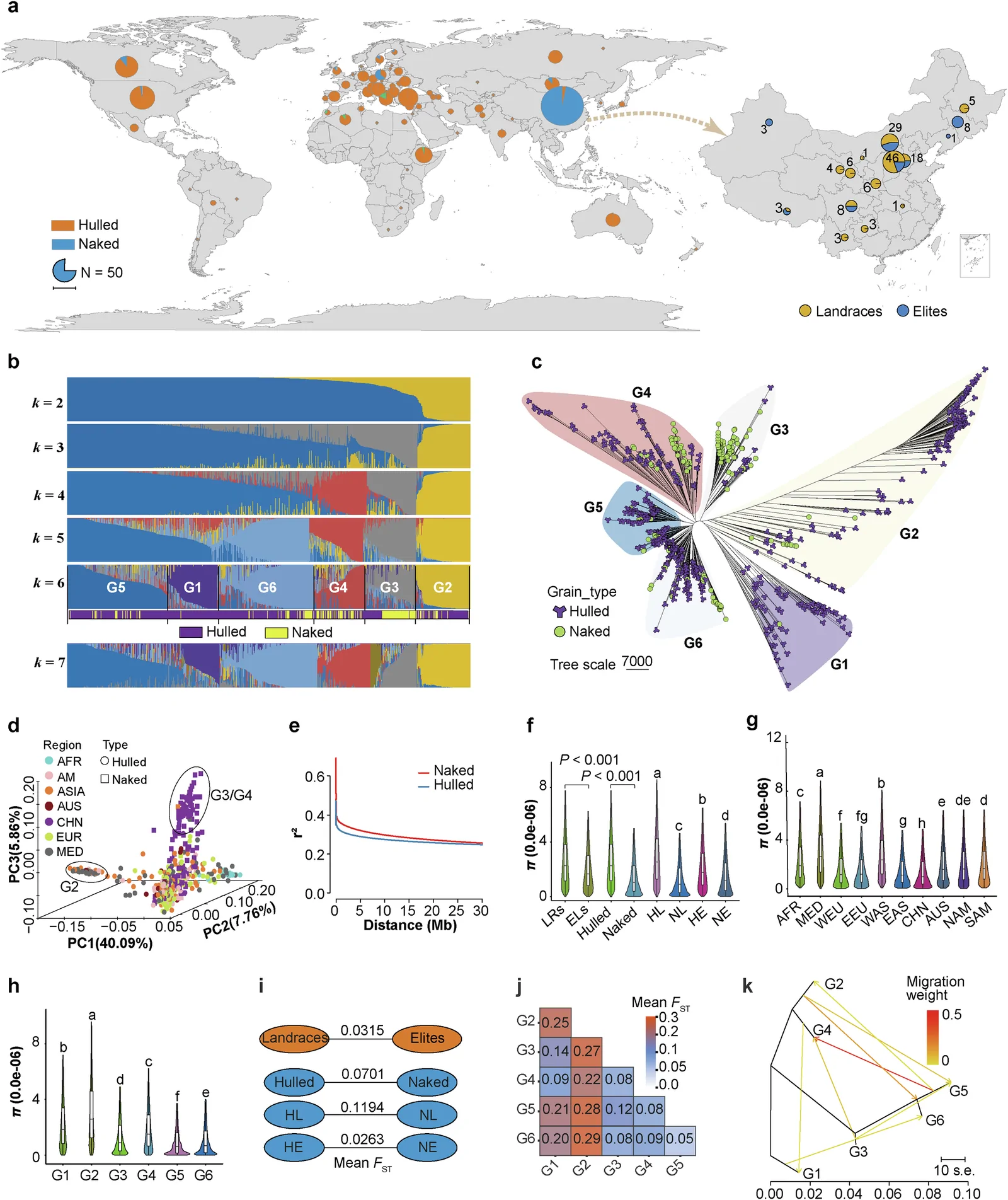
Population structure and genetic diversity analysis of 666 oat germplasm resources based on 127,563 SNP markers. **a** Global distribution of 666 tested accessions and distribution map of 162 Chinese naked oat accessions; (**b**) Genetic ancestry components under different assumed population numbers (*K* = 2–7) and subgroup assignment (G1–G6) at *K* = 6. Source data are provided in Supplementary Data 2 (Table S9a); (**c**) Unrooted phylogenetic tree: subgroups (G1–G6) and hulled/naked grain distribution; (**d**) Three-dimensional (3D) plot of the first three principal components from principal component analysis (PCA) of 666 oat germplasms based on 127,563 SNP markers (Abbreviations: AFR, Africa; AM, Americas; ASIA, Asia; AUS, Australia; CHN, China; EUR, Europe; MED, Mediterranean region); (**e**) Linkage disequilibrium (LD) half-decay analysis of hulled and naked oats; (**f**–**h**) Comparative analysis of nucleotide diversity (*π*) among different oat groups (*P* < 0.05) (Abbreviations: WEU, Western Europe; EEU, Eastern Europe; WAS, West Asia; EAS, East Asia; NAM, North America; SAM, South America; LRs, landraces; ELs, elite cultivars; HL, hulled landraces; NL, naked landraces; HE, hulled elites; NE, naked elites). Source data for (**f**–**h**) are provided in Supplementary Data 1 (Table S2.1a) and Supplementary Data 2 (Table S10a, b); (**i**, **j**) Analysis of genetic differentiation among different oat groups based on fixation index (*F*_ST_); (**k**) Phylogenetic topology and gene flow direction of G1-G6 subgroups with eight fitted migration events.

Based on 127,563 bi-allelic SNPs filtered with minor allele frequency (MAF) ≥ 0.05 and missing rate ≤ 0.1 (Fig. S1), we characterized the global population structure and genetic diversity of 666 oat accessions (659 with previously published GBS data^15^; 7 newly generated in this study) from 65 countries and regions (Fig. 1a and Supplementary Data 1, Table S1.1). By integrating Admixture cross-validation (*K* = 1–20) with neighbor-joining (NJ) phylogenetic analysis using a threshold of >0.5 for ancestry proportion, we divided these accessions into six subgroups (G1–G6) (Figs. 1b, c and S2; Supplementary Data 1, Table S1.2). While accessions of each subpopulation exhibit distinct geographic origins, prevalent genetic admixture between hulled and naked oats and cross- regional gene flow are present, with no strict geographic-genetic correspondence. Specifically, G1 represents the population convergence of West Asia (WAS) and Africa (AFR), comprising 57 hulled oats predominantly from Ethiopia and Egypt, 7 wild relatives, and one Chinese naked landrace from Hebei. G2 constitutes a hulled oat landrace core subpopulation from the Mediterranean (MED; 57 accessions) and West Asia (34 accessions). G3 is a subpopulation dominated by Chinese (CHN) naked oats, which account for 79.2% of their accessions. G4 represents trans-Pacific gene flow, consisting of 43 North American (NAM) hulled oats and 41 Chinese naked oats from northern domestication hubs. G5 is a Sino-Eurasian mosaic comprising 53 European (EUR) hulled oat elites and 13 Chinese naked oat landraces from the Shanxi-Hebei-Inner Mongolia region. G6 is a typical geographically adjacent admixed subpopulation, comprising 54 Mediterranean hulled oat landraces, 28 European hulled oats, and 55 Asian accessions (including 28 Chinese naked oats). Principal component analysis (PCA) supports this population structure and widespread genetic admixture: only G2 forms independent clustering on PC1, and G3 (the core naked oat subpopulation) clusters exclusively on PC3; extensive grain-type and geographic overlap further confirms pervasive global germplasm admixture (Fig. 1d and Supplementary Data 1, Table S1.3).

Hulled oats exhibit significantly higher nucleotide diversity (*π* = 2.79 × 10^−6^) and faster linkage disequilibrium decay (0.96 Mb) than naked oats (*π* = 1.64 × 10^−6^; 5.32 Mb) (Fig. 1e, f and Supplementary Data 1, Table S2.1). Together with the higher proportion of long runs of homozygosity (ROH, 1 Mb–3 Mb) in naked oats (14.4%) relative to hulled oats (9.5%), naked oats harbour a proportion of loci with Tajima’s *D* < 0 that is 18.4-fold that of hulled oats. These results demonstrate that naked oats underwent a stronger genetic bottleneck and intensified domestication selection pressure (Figs. S3c and S4a, b and Supplementary Data 1, Tables S2.2 and S3.1). Among the six subgroups (Figs. 1g–j and S3 and Supplementary Data 1, Tables S2.5 and S2.6), G2 exhibited the weakest domestication selection (Tajima’s *D* < 0, 1.4%–4.8%) and the highest nucleotide diversity (*π* = 3.31 × 10^−6^). G3 exhibits intense domestication selection, alongside markedly reduced genetic diversity, with the proportion of loci with Tajima’s *D* < 0 11.7–16.3-fold that of G2. G5 displays the lowest nucleotide diversity (*π* = 1.26 × 10^−6^) due to intensive artificial breeding and domestication pressure. Weak overall genetic differentiation between hulled and naked oats (*F*_ST_ = 0.0701) aligns with their substantial germplasm overlap. Subpopulation differentiation patterns are highly heterogeneous, driven by long-term geographic isolation and divergent selection pressure. Specifically, G2 shows strong genetic differentiation from all other subgroups (*F*_ST_ > 0.15). In contrast, G4 shows weak differentiation from most subgroups (*F*_ST_ = 0.05–0.09) and retains high nucleotide diversity (*π* = 2.07 × 10^−6^).

ROH scanning, ABBA-BABA tests, and TreeMix analysis revealed extensive inter-subpopulation introgression shaping global oat germplasm diversity. We detected robust introgression from hulled oat elites into naked oats (*D* = 0.35, *P* < 0.01), which explains the weak interspecific differentiation and germplasm overlap between the two grain types (Figs. S4, S6a and Supplementary Data 1, Table S3.1). By contrast, among the six subgroups (Fig. 1k, S5, S6b–r and Supplementary Data 1, Table S3.2), moderate-to-high introgression from Mediterranean (MED) hulled landraces (G2) is widespread across all geographical regions and all other genetic subgroups. Supported by significant genetic differentiation estimates (*D* = 0.18–0.48, *P* < 0.05), these findings collectively establish G2 as the primary genetic donor within the global oat germplasm network. Additionally, frequent and bidirectional introgression occurs between G4 (NAM hulled oats) and MED/EUR hulled oats, along with high proportions of shared long homozygous linkage fragments (1–3 Mb; 26.2%–37.8%), which demonstrating that G4 acts as the core fusion hub subgroup of global high-quality oat germplasm resources. Moderate introgression from East Asian (EAS)/CHN oat clades into MED, EUR, and NAM germplasm was also detected. Collectively, multidirectional transcontinental introgression, divergent domestication pressures, and geographic isolation have shaped the global genomic landscape of cultivated oats. Identifying G2 as the primary genetic donor and G4 as a modern breeding hub provides a foundation for future genetic improvement and global utilization of oat germplasm.

### Domestication pathway of naked oats

**Fig. 2:**
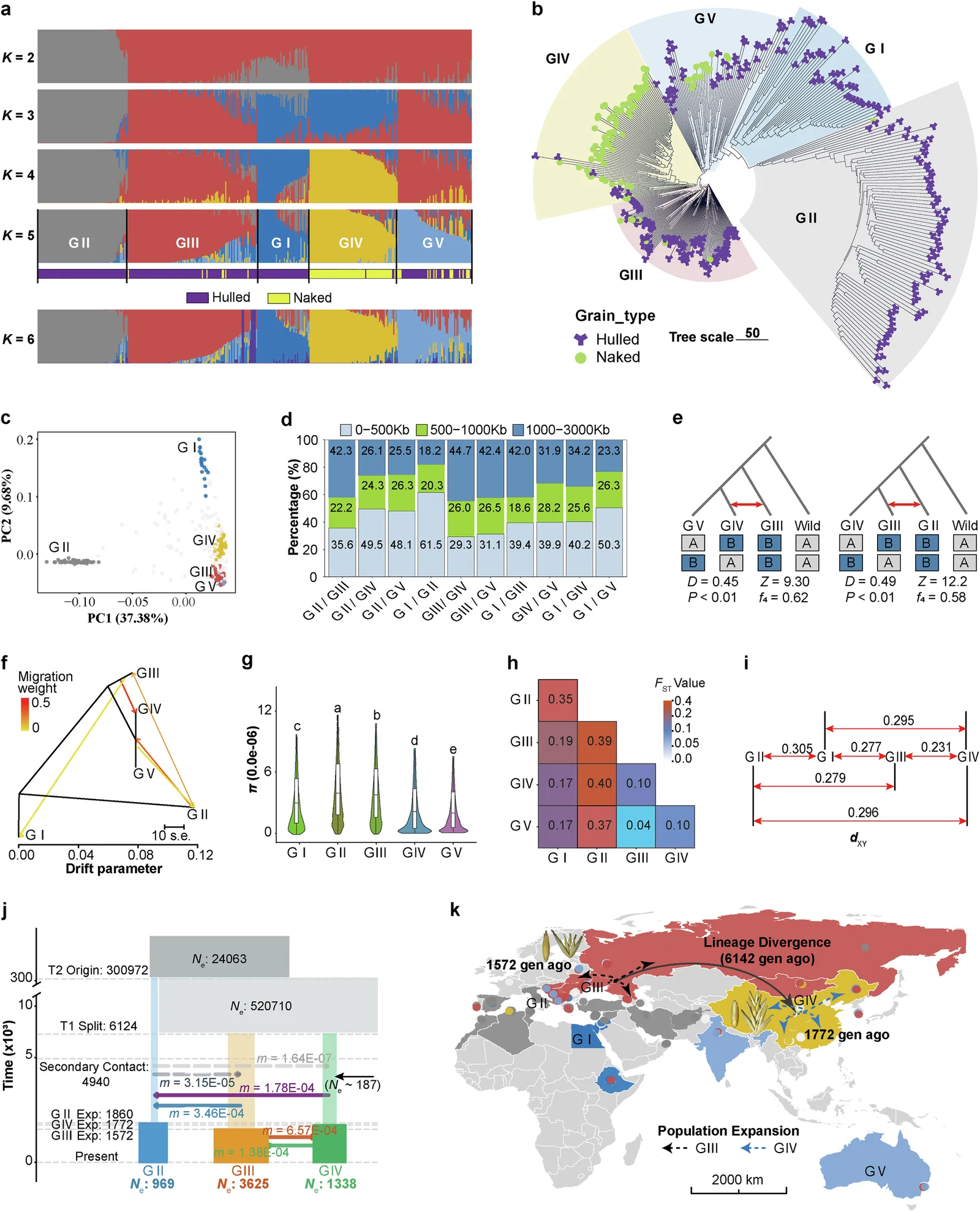
Analysis of the domestication pathway of naked oats based on 366 landraces and 7 wild accessions of hexaploid oat. **a** Genetic ancestry components of 366 landraces and 7 wild oats under different assumed population numbers (*K* = 2–6), and subgroup assignment results at *K* = 5. Source data are provided in Supplementary Data 2 (Table S9b); (**b**) Unrooted phylogenetic tree of 373 oats: composition and distribution of each subgroup (GⅠ-GⅤ); (**c**) Population clustering of 366 landraces and 7 wild oats based on the PC1 and PC2 (Gray dots represent admixed accessions with ancestry proportion < 0.8. This definition also applies to Fig. 2k); (**d**) Proportions of shared homozygous haplotypes of different lengths (10 kb–3 Mb) among subgroups; (**e**) ABBA-BABA test revealing significant gene flow between GⅡ and GⅢ-GⅣ subgroups, and between GⅢ and GⅣ-GⅤ subgroups; (**f**) Phylogenetic topology and gene flow direction among clustered subgroups with 5 fitted migration events; (**g**) Nucleotide diversity levels among different subgroups (*P* < 0.05). Source data are provided in Supplementary Data 2 (Table S10c); (**h**) Fixation index (*F*_ST_) values among different subgroups; (**i**) *d*_xy_-based phylogenetic tree showing genetic diversity differentiation among GⅠ–GⅣ subgroups; (**j**) Demographic history reconstruction of GⅡ, GⅢ, and GⅣ using fastsimcoal2; (**k**) Geographic origin distribution of accessions in each subgroup (GⅠ–GⅤ), as well as a schematic diagram of the divergence time and recent population expansion between the core subgroup of Chinese naked oats (GⅣ) and its ancestral subgroup GⅢ (hulled oats in Central-Eastern Eurasia).

The domestication origin of naked oats has long remained controversial regarding whether it originated independently from cultivated hulled oats. To minimize confounding gene flow from modern breeding and resolve this controversy, we selected 366 landraces with conserved ancient genetic backgrounds, together with seven wild hexaploid oats, for population evolutionary and gene flow analyses. Population structure analysis partitioned the 373 oats into 5 subpopulations (Figs. 2a–c and S9a; Supplementary Data 1, Table S4.1): three hulled oat-dominated subpopulations (GⅠ–GⅢ), corresponding to the Northeast African and West Asian group (GⅠ: 45 accessions, including 7 wild accessions), the Mediterranean Basin group (GⅡ: 77 accessions), and the Central and Eastern Eurasian group (GⅢ: 110 accessions); a core naked oat subpopulation (GⅣ), where over 74.0% of Chinese naked oats form a single isolated clade; and a mixed subpopulation (GⅤ) consisting of 63 admixed hulled and naked oats from multiple geographic origins. Although most Chinese naked oats formed a distinct subpopulation, 26.0% of accessions showed genetic admixture with other subpopulations and geographically diverse hulled oats (Supplementary Data 1 and Table S4.1). Gene flow analysis revealed 7.5%–16.8% shared haplotypes across subpopulations and significant multidirectional introgression in most pairwise comparisons (*D* > 0.45, *Z* > 9, *P* < 0.001) (Figs. 2d–f and S9b and Supplementary Data 1, Table S4.2). Substantial inter-subpopulation gene flow persisted between hulled and naked oat landraces after removing all modern elite cultivars. These findings reveal that naked oats have not established complete reproductive isolation from hulled oat landraces and consequently fail to support the hypothesis of fully independent domestication for naked oats.

We next explored patterns of genetic differentiation and diversity among subpopulations to delineate the ancestral hulled oat lineages and the immediate donor lineages of Chinese naked oats. Genetic differentiation analysis identified the Mediterranean Basin group (GⅡ) as the most divergent (*F*_ST_ = 0.35–0.40) and genetically diverse (*π* = 2.75 × 10^−6^), suggesting it is the ancestral diversity center retaining ancient landrace variation (Fig. 2g, h and Supplementary Data 1, Table S4.2). In contrast, extensive gene flow (*D* = 0.45, *Z* = 9.3, *P* < 0.001), minimal differentiation (*F*_ST_ = 0.10), and the highest proportion of shared long ROH fragments (1–3 Mb; 44.7%) occur between the Central and Eastern Eurasian group (GⅢ) and Chinese naked oats (GⅣ) (Fig. 2d–h and Supplementary Data 1, Table S4.2). Phylogenetic analysis based on absolute genetic distance (*d*_xy_) further quantified these relationships: GⅣ shows the smallest *d*_xy_ with GⅢ (0.231), confirming their close genetic affinity, whereas GⅡ exhibits the largest *d*_xy_ with all other groups (0.279–0.305), consistent with its early divergence (Fig. 2i). These findings position GⅢ as the most direct genetic source for GⅣ.

To validate the inferred evolutionary relationships and quantify divergence times, we performed demographic modeling using fastsimcoal2. We pre-filtered accessions within GⅡ, GⅢ, and GⅣ with ancestral component contributions below 0.8 to mitigate confounding from recent introgression. The no-migration model was rejected across all pairwise comparisons, verifying extensive post-divergence gene flow between these lineages (Supplementary Data 1 and Table S4.3). The optimal three-population model confirmed that GⅣ is a recent derivative of the GⅢ lineage (Fig. 2j, k and Supplementary Data 1, Table S4.3): it diverged from the GⅢ ancestral pool ~6124 generations ago, underwent an early isolation period of 1184 generations (~1200 generations), and initiated secondary population contact ~4940 generations ago. Subsequently, GⅣ underwent demographic expansion at 1772 generations ago, while GⅢ initiated its expansion at 1572 generations ago, with renewed prominent genetic exchange during their recent expansion phases. During early divergence, GⅣ experienced an extreme founder bottleneck (*N*_e_ ≈ 187), with strong genetic drift accelerating the fixation of unique naked grain traits. This expansion is consistent with the rapid adaptation and diffusion typical of derived lineages post-domestication. GⅡ’s *N*_e_ (969) was markedly lower than the model-predicted value (1,860), while GⅢ’s *N*_e_ (3625) exceeded expectations (1572) and GⅣ’s *N*_e_ (1338) closely matched the predicted value (1772). Integrating archaeological and historical records, the evolutionary chronology of the GⅢ and GⅣ lineages aligns well with the eastward spread of West Asian cereal agriculture into North China; additionally, local naked oat landraces in North China exhibit the highest levels of nucleotide diversity and selection pressure among all Chinese naked oat germplasms (Fig. S9c, d and Supplementary Data 1, Table S4.4), and these lines of evidence support North China as a secondary domestication center for naked oats. Synthesizing all population genetic and demographic evidence, we propose a stepwise domestication pathway for oats: Hulled oats underwent primary domestication from wild hexaploid species in the Mediterranean Basin, which subsequently dispersed southeastward to East Africa and eastward to Central and Eastern Eurasia. During this eastward dispersal, naked oats diverged from the Central and Eastern Eurasian hulled oat lineage (GⅢ) ~6124 generations ago, followed by secondary domestication and rapid expansion in North China.

### Phenotypic divergence analysis

**Fig. 3:**
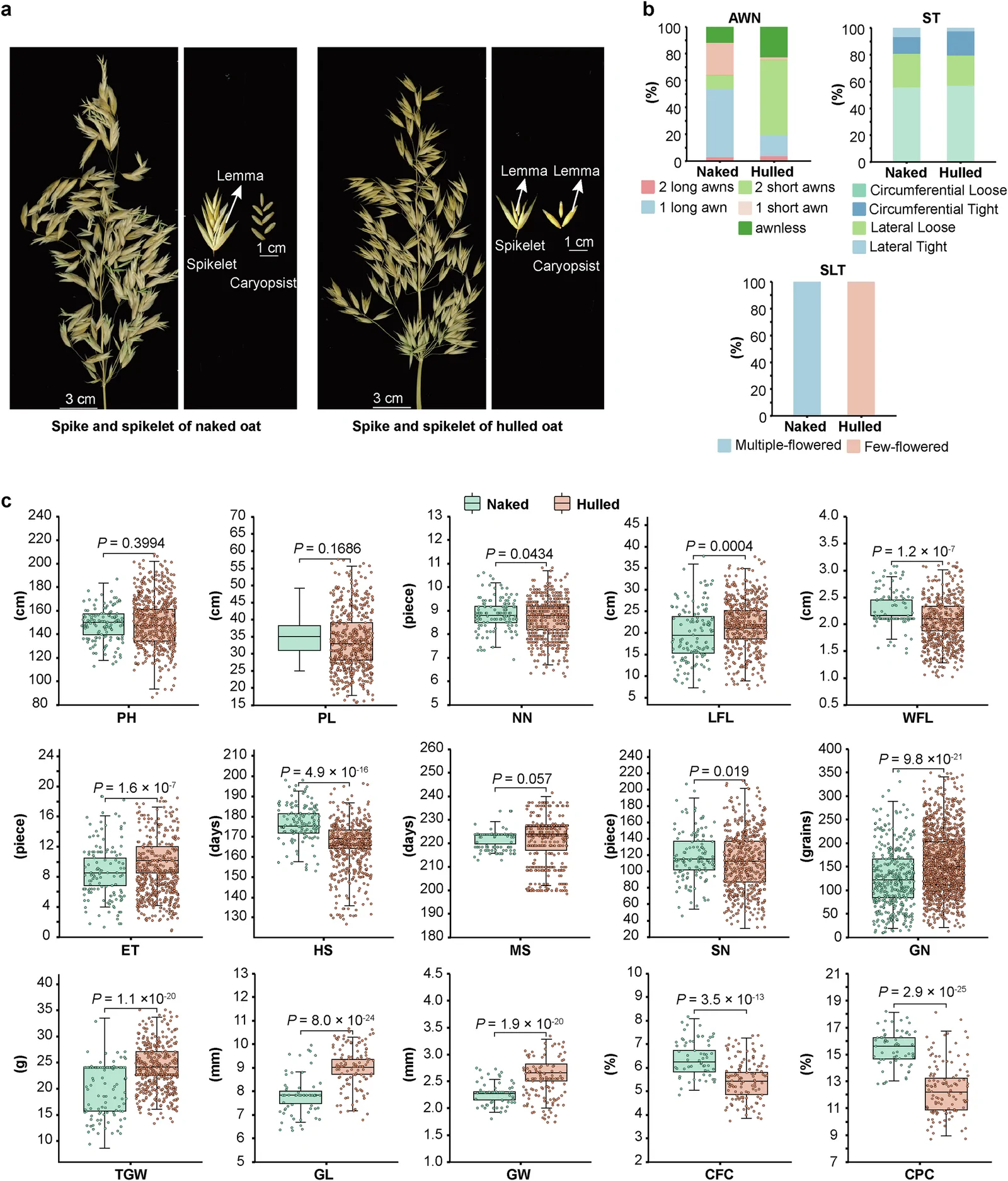
Comparative analysis of panicle morphological and phenotypic differences between hulled and naked oats. **a** Morphological characteristics of panicles, spikelets and grains in mature hulled and naked oats; (**b**) Proportional statistics of accessions with different awn types, panicle types and spikelet types in hulled and naked oats; (**c**) Box plots for comparative analysis of 8 growth and development traits, 5 grain yield-related traits and 2 grain quality-related traits between hulled and naked oats. Abbreviations and corresponding units of all traits in the figure are as follows: Grain Circumference Index (GCI, mm); Crude Fat Content & Crude Protein Content (CFC & CPC, %); Flag-Leaf Length (LFL, cm); Flag-Leaf Width (WFL, mm); Grain Length (GL, mm); Grain Width (GW, mm); Heading Stage & Milking Stage (HS & MS, in 7-day increments); Grain Number per Panicle (GN, in 5-unit increments); Node Number (NN, in 5-unit increments); Plant Height (PH, dm); Panicle Length (PL, cm); Thousand-Grain Weight (TGW, g); Hulled/Naked Grain Type (H/N). Source data for (**b**, **c**) are provided in Supplementary Data 2 (Table S11).

Building on population structure analysis, we dissected the genetic basis of 19 oat agronomic traits using multi-environment phenotypic data. Optimal genetic models and broad-sense heritability (*H*²) were determined via LRT-*P* and BIC. Model analysis showed that hulled/naked trait and spikelet type were regulated solely by variety main effects; awn presence, panicle type, grain width, crude protein, and crude fat were influenced by variety and variety × year interactions; most other traits were environmentally regulated. Heritability estimates were moderate for PH, HS, and ET (*H*² = 0.41–0.53) and high for all other traits (*H*² ≥ 0.6), indicating predominant genetic control (Supplementary Data 1, Table S5.1). Using whole-genome SNP markers, genetic correlation analysis among the 19 traits implemented in GCTA (v1.93.2) revealed significant negative correlations between yield-related traits (TGW, GL, GW) and quality traits (CFC, CPC) (*r* = –0.22 to –0.34, *P* < 0.05). Hulled traits correlated positively with yield traits (*r* = 0.28–0.49, *P* < 0.01), while naked traits correlated positively with quality traits (*r* = 0.53–0.67, *P* < 0.01), reflecting domestication-driven phenotypic specialization (Fig. S10 and Supplementary Data 1, Table S5.2). Multi-environment evaluation of the above traits in 504 hulled and 162 naked oat accessions revealed significant domestication-driven phenotypic divergence (Fig. 3). For growth and development traits, naked oats exhibited 44.9% of multiflowered spikelets and long-awned phenotypes (20.2% in hulled oats), indicating divergent awn trait selection; both hulled and naked oats favored umbellate spikes to optimize light interception and lodging resistance (Fig. 3b and Supplementary Data 1, Table S5.3). In terms of phenological and yield-related traits, hulled oats exhibited earlier heading and extended filling periods (11 days longer), which facilitated photosynthetic accumulation and superior yield traits (thousand-grain weight increased by 21.5%; grain length increased by 15.2%). Critically, naked oats showed 23.2% fewer grains per spikelet despite higher spikelet counts, implicating seed-setting efficiency as the yield-limiting bottleneck. In contrast, naked oats achieved nutritional superiority, with crude protein increasing by 25.5% (*P* < 0.001) and crude fat increasing by 16.9% (*P* < 0.001) (Fig. 3c and Supplementary Data 1, Table S5.3).

Principal component analysis (PCA) of phenotypic data showed that the top 10 principal components captured 88.92% cumulative variance Among the major eigenvectors contributing to the principal components, grain size, thousand-grain weight, and quality-related traits (PC1, 24.38%), together with developmental traits like plant height, spike length, and heading date (PC2, 17.80%), accounted for substantial phenotypic genetic variation. PC3 primarily captured variation in growth cycle and the status of lemma covering status (Supplementary Data 1 and Table S5.4). Phenotypic PCA revealed population differentiation consistent with marker-based analysis, reflecting the breeding characteristics of distinct genetic backgrounds and yield-quality divergence between hulled and naked oats (Figs. S11, S12 and Supplementary Data 1, Table S5.5). Consistent with this observation, G3, dominated by naked oats, showed the highest crude protein (15.06%) and crude fat (6.22%) contents. G2, composed mainly of Mediterranean and West Asian hulled landraces, exhibited superior thousand-grain weight (26.48 g) and kernel length (9.45 mm), reflecting a large-kernel, high-yield phenotype distinct from G3. G4, comprising North American elite cultivars, was characterized by early heading (135.28 d), large flag-leaf area (34.52 cm²), and high thousand-grain weight (26.47 g), aligning with breeding objectives for earliness and yield. These phenotypic differences establish a robust link between variation and genetic background, providing key evidence for dissecting the underlying genomic regions and functional genes.

### Genome-wide scanning of selection signatures in naked oats

**Fig. 4:**
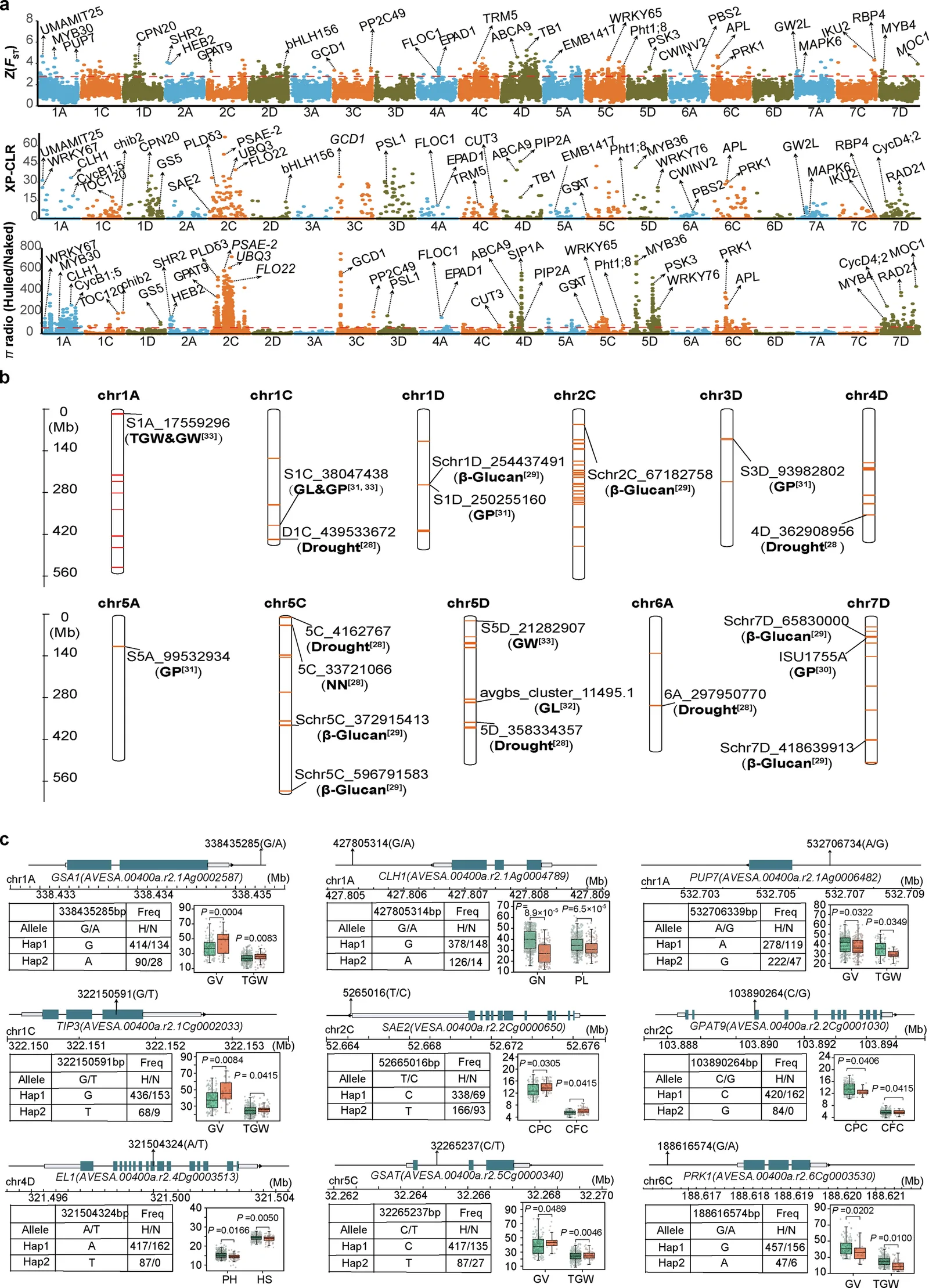
Genome-wide scan analysis of selected gene loci in naked oats. **a** Manhattan plot of genome-wide selected loci in naked oats identified by combining *Z*(*F*_ST_), nucleotide diversity ratio and XP-CLR values with hulled oats as the reference; (**b**) Colocalization results of selected genes in naked oats with previously published QTLs associated with grain yield, quality and stress traits in oats (red horizontal lines in the chromosome schematics indicate the positions of selected genes); (**c**) Box plots of comparative phenotypic differences among different haplotypes of selected genes in naked oats, constructed based on the genotypic data of tested accessions. Abbreviations and corresponding units of all traits in the figure are as follows: Crude Fat Content & Crude Protein Content (CFC & CPC, %); Grain dimension (GV, mm^3^); Heading Stage (HS, in 7-day increments); Grain Number per Panicle (GN, in 5-unit increments); Plant Height (PH, dm); Panicle Length (PL, cm); Thousand-Grain Weight (TGW, g); Hulled/Naked Grain Type (H/N). Source data for (**c**) are provided in Supplementary Data 1 (Table S8.2a) and Supplementary Data 2 (Table S11).

The pronounced phenotypic differences between hulled and naked oats in growth, yield, and quality traits indicate substantial genomic differentiation between the two oat types. Building upon established population structure and phenotypic differentiation between hulled and naked oats, we quantified chromosomal-level genetic divergence during oat domestication using Tajima’s *D* and nucleotide diversity (*π*). The results showed that except for chromosomes 2D, 3A, 6D, and 7C (*π* differences, *P* > 0.05; Tajima’s *D* ≥ 0 spanning > 83%), the remaining 17 chromosomes, naked oats showed significantly reduced *π* (loss rate: 37.75%–73.92%, *P* < 0.001) and elevated selection pressure (*D* < 0; 29-fold increase) relative to hulled oats (Fig. S13 and Supplementary Data 1, Table S2.4), indicating that naked oats underwent a stronger genetic bottleneck and more intensive artificial selection during domestication, thereby laying the genomic foundation for their distinct phenotypic differentiation. Integrating the population differentiation index (*F*_ST_), *π* ratio (hulled/naked), and XP-CLR, we screened for potential selective sweeps in naked oats (*Z*(*F*_ST_) > 2.80, *P* < 0.001, and the top 1% of windows identified by *π* ratio and XP-CLR analyses). We identified 1504 putative selected genes (Fig. 4a and Supplementary Data 1, Table S6.2). Among these, 883 exhibited directional selection (*D* < 0), and 621 tended toward balancing selection (*D* ≥ 0) (Fig. S14 and Supplementary Data 1, Table S6.2). Functional enrichment analyses showed selected genes retain core cellular metabolism but diverge markedly in domestication pathways. Directionally selected genes significantly enriched lipid, amino acid, and vitamin biosynthesis pathways (GO:0009110, GO:0019375, ko00591), directly correlating with enhanced nutrient accumulation in naked oats (Fig. S15 and Supplementary Data 1, Table S6.4). In contrast, balancing-selected genes clustered in chlorophyll a oxidase activity (GO:0016049, ko00195) and butanoate metabolism (ko00650), suggesting compromised photosynthetic efficiency and growth regulation in naked oats (Fig. S16 and Supplementary Data 1, Table S6.5).

To investigate the association between selection signals and phenotypic differentiation, we colocalized selected genes in naked oats with published QTLs. We identified selected genes associated with 20 known QTLs^28–33^ across 11 chromosomes, and their selection patterns were highly consistent with phenotypic differentiation between hulled and naked oats. By trait category, only 11 of 51 genes linked to yield-related QTLs (e.g., thousand-grain weight, grain size) showed directional selection. In contrast, 38 of 50 genes associated with crude protein content and 86 of 113 genes for β‑glucan content exhibited signatures of directional selection (Fig. 4b and Supplementary Data 1, Table S6.2), indicating stronger selection on quality‑related genes during naked oat domestication. Moreover, 117 of 178 genes linked to five drought‑responsive QTLs showed directional selection (Fig. 4b and Supplementary Data 1, Tables S6.2 and S6.6), implying enhanced adaptation to specific ecological niches. Homology annotation and allele frequency analysis further identified multiple selected genes linked to phenotypic divergence between hulled and naked oats. For example, accumulation of beneficial haplotypes of glyceride synthesis gene *AsGPAT9* and seed ovule development gene *AsSAE2* in naked oats exhibit consistency with higher grain crude protein and fat. Conversely, selective sweeps of favorable haplotypes in grain development regulator *AsGSA1* and aquaporin gene *AsTIP3* exhibit consistency with reduced naked oat yield traits. Furthermore, the selective sweep of the Hap2 haplotype in photodamage repair gene *AsCLH1* exhibit consistency with shorter spikes and fewer tillers in naked oats. Similarly, Hap2 sweep in photoperiod regulator *AsEL1* likely underlies delayed flowering. Haplotype–phenotype associations for these selected genes confirmed the link between genome-wide selection signals and phenotypic divergence, providing valuable candidate targets for marker-assisted selection (MAS) in oat breeding (Fig. 4c and Supplementary Data 1, Tables S6.3 and S8.2).

### Transcriptional regulatory divergence in hulled and naked oats

To investigate transcriptional regulatory divergence between hulled and naked oats, we conducted RNA-seq analysis on pooled root, stem, leaf, and spike tissues from 13 naked oat and 9 hulled oat accessions with geographically and genetically diverse backgrounds, and further integrated 16 public transcriptome datasets^15,16,34–38^ (7 for seed development, 9 for salinity and drought stress) for comprehensive multi-dimensional comparisons (Supplementary Data 1 and Table S7.2). Enrichment analysis of differentially expressed genes (DEGs) during seed development revealed that upregulated genes in naked oats were primarily associated with pathways related to nutritional quality accumulation, including developmental signaling, sterol-lipid synthesis, and ion channel activity (e.g., GO:0009873, GO:0009867, GO:0000149, ko00906, ko00543; *q* < 0.05). This suggests transcriptional reprogramming favors nutrient synthesis and accumulation in the grain. In contrast, upregulated genes in hulled oats during seed development were primarily enriched in pathways for cell wall organization, transmembrane transport, and basal metabolism (e.g., GO:0009834, GO:0070592, ko02010, ko00430; *q* < 0.05), potentially linking to nutrient transport efficiency and growth regulation. Under abiotic stress, naked oats upregulated genes involved in ion homeostasis, stress signal transduction, and specific secondary metabolism (GO:0006561, GO:0017157, ko99988, ko00906), whereas hulled oats exhibited enrichment in pathways for cell wall remodeling, terpenoid biosynthesis, and ABC transporter-mediated transport (GO:0009834, GO:0016102, GO:0006721, ko02010; *q* < 0.05), highlighting distinct transcriptional bases underlying their differential stress adaptation strategies (Figs. S17, S18 and Supplementary Data 1, Tables S7.3–S7.5).

**Fig. 5:**
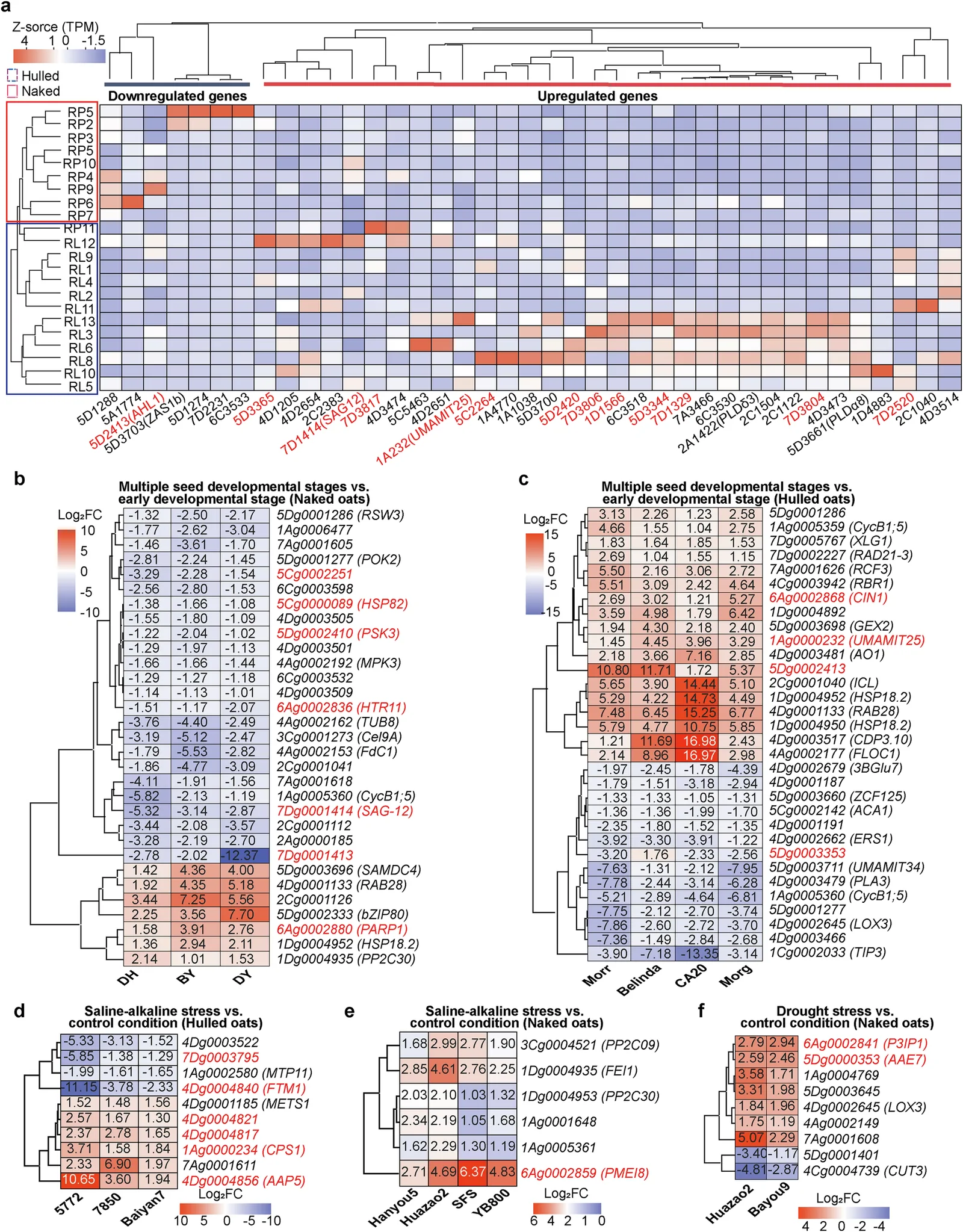
Expression heatmap of selected and differentially expressed genes in naked oats based on Log_2_FC values (Genes marked in red are associated with previously published oat QTLs). **a** Intersection of DEGs between hulled and naked oats from bulk RNA-seq and selected genes in naked oats; (**b**) Intersection of DEGs from seed development transcriptome of naked oats and selected genes in naked oats; (**c**) Intersection of DEGs from seed development transcriptome of hulled oats and selected genes in naked oats; (**d**) Intersection of DEGs from saline-alkali stress transcriptome of hulled oats and selected genes in naked oats; (**e**) Intersection of DEGs from saline-alkali stress transcriptome of naked oats and selected genes in naked oats; (**f**) Intersection of DEGs from drought stress transcriptome of naked oats and selected genes in naked oats.

To identify key genes underlying hulled-naked oat differentiation, we integrated 1,504 candidate selected genes in naked oats with multi-context transcriptome data to construct a combined selection signal-expression association profile. Our results revealed that during core physiological processes, we identified 39 selected genes exhibiting differential expression between hulled and naked oats, comprising 31 upregulated and 8 downregulated genes (e.g., *AsUMAMIT25*, *AsAHL1, AsSAG12*, *Asygl8*, *AsWOX13*). Among these, 22 genes under directional selection and upregulated expression were significantly enriched in pathways such as lipid metabolism and post-translational protein modification (Fig. 5a and Supplementary Data 1, Table S7.6). During the seed development stage, 31 candidate selected genes were detected. Among them, upregulated genes (e.g., *AsPARP1*, *AsRAB28*, *AsPP2C30*, *AsHSP18.2*) may be involved in embryonic cell division and differentiation and ABA signal response, whereas genes under balancing selection and downregulated expression (e.g., *AsMPK3*, *AsCycB1;5*, *AsRSW3*) were enriched in cell cycle regulation pathways (*q* < 0.05) (Fig. 5b and Supplementary Data 1, Table S7.6). In contrast, the 32 differentially expressed genes related to seed development in hulled oats exhibited a pattern of cooperatively upregulating energy metabolism and signal transduction while downregulating redundant hormone pathways (Fig. 5c and Supplementary Data 1, Table S7.6). Furthermore, in response to abiotic stress, 25 selected genes specifically expressed in naked oats (e.g., *AsPP2C09*, *AsFEI1*, *AsPME18*, *AsPIP3I*) generally displayed signatures of directional selection and upregulated expression, suggesting their potential functions in stress adaptation (Fig. 5d–f and Supplementary Data 1, Table S7.6). Further analysis revealed that 33 of the aforementioned 127 associated genes co-localized with published QTLs for oat yield and quality traits (Fig. 5 and Supplementary Data 1, Table S7.6, highlighted in red), significantly enhancing the credibility of these candidate genes in trait differentiation.

### Discovery of beneficial haplotypes facilitating oat improvement

**Fig. 6:**
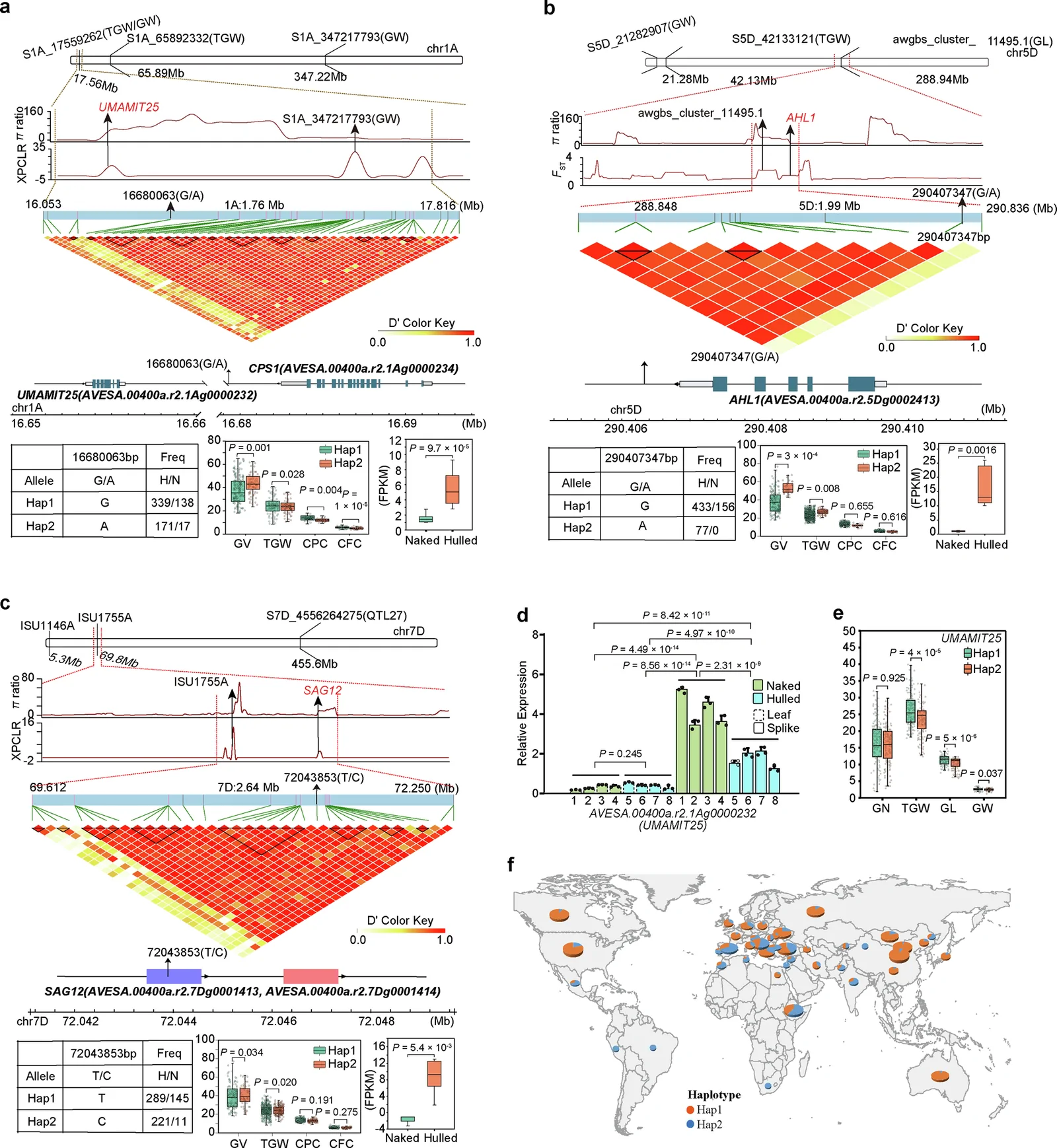
Identification of elite haplotypes for oat genetic improvement. **a**–**c** Haplotype analysis of three selected genes in naked oats linked to QTLs for grain yield and quality traits, based on genotypic and phenotypic data of 666 tested accessions. From top to bottom, the panels show: the genetic linkage map of oat yield and quality QTLs identified in previous studies; the linkage disequilibrium heatmap of genomic segments harboring the selected genes associated with target QTLs; the structural features of target genes and SNP site information reflecting haplotype divergence; boxplots comparing thousand-grain weight (TGW, g), grain volume (GV, mm³), crude protein content and crude fat content (CPC & CFC, %) across different haplotypes; and bar charts of the relative expression levels of target genes in hulled and naked oats based on FPKM values. Source data for (a–c) are provided in Supplementary Data 1 (Table S8.1a) and Supplementary Data 2 (Table S11); (**d**) qRT-PCR analysis of *AsUMAMIT25* in leaves and spikelets of hulled and naked oats at the grain-filling stage. Source data are provided in Supplementary Data 1 (Table S8.4a); (**e**) Boxplots of grain number per spike (GN), thousand-grain weight (TGW, g), grain length (GL, mm) and grain width (GW, mm) across different haplotypes identified by KASP markers. Source data are provided in Supplementary Data 1 (Table S8.3a) and Supplementary Data 2 (Table S11); (**f**) Geographical distribution of oat germplasm carrying the beneficial *AsUMAMIT25* haplotype (HAP2).

Comprehensive analysis of the 33 genes showing multi-dimensional evidence from phenotype association, selection signals, and transcriptional expression revealed that three were significantly associated with previously reported oat grain yield QTLs. Distinct haplotypes of these genes exhibited marked variation in yield and quality traits across 666 accessions. Notably, a haplotype block on Chr1A, comprising the amino acid transporter gene *AsUMAMIT25* (*AVESA.00400a.r2.1Ag0000232*) and the gibberellin biosynthesis gene *Ascps1* (*AVESA.00400a.r2.1Ag0000234*), showed strong associations with yield-related traits. Within this block, a G/A SNP upstream of *AsUMAMIT25* defined the beneficial Hap2 (A) haplotype, significantly correlating with increased grain size and thousand-grain weight (Fig. 6a and Supplementary Data 1, Table S8.1). Geographically, it predominates in Mediterranean hulled oat landraces and North American hulled breeding lines, while concentrating in northern China (Gansu, Shanxi, Inner Mongolia) for naked oats (Fig. 6f and Supplementary Data 1, Table S8.1). KASP markers from this variant discriminated genotypes across 268 hexaploid accessions, with phenotypes matching predictions (Figs. 6e and S19a and Supplementary Data 1, Table S8.3), providing a direct genetic resource to narrow the naked oat yield gap via molecular breeding. The gene *AsAHL1* (*AVESA.00400a.r2.5Dg0002413*) modulated photosynthetic capacity by regulating chlorophyll biosynthesis. Haplotype analysis indicated that 77 hulled oat accessions carrying the Hap2 (A) haplotype, which are predominantly landraces distributed across the Mediterranean and Eurasia, maintain grain yield advantages without compromising quality traits (Fig. 6b and Supplementary Data 1, Table S8.1). These accessions represent valuable germplasm for breeding and research into coordinated high-yield and high-quality mechanisms in oats. Furthermore, during domestication, directional selection for beneficial haplotypes (Hap1/T) of the proteolytic enzyme-related gene *AsSAG12* (*AVESA.00400a.r2.7Dg0001413*, *AVESA.00400a.r2.7Dg0001414*) in naked oats strongly correlated with increased grain crude protein content (Fig. 6c and Supplementary Data 1, Table S8.1). Collectively, these findings pinpoint actionable targets for bridging yield and nutritional gaps between hulled and naked oats.

To validate expression patterns, we performed qRT-PCR analysis on leaf and spikelet tissues at the grain-filling stage for the three candidate genes (*AsSAG12*, *AsUMAMIT25*, *AsAHL1*) (Figs. 6d and S19b and Supplementary Data 1, Table S8.4). Results implied that *AsSAG12* likely directionally regulates grain quality formation, whereas *AsUMAMIT25* and *AsAHL1* may directionally influence grain size. Additionally, integration of multiple public transcriptome datasets from the Sequence Read Archive (SRA) of the National Center for Biotechnology Information (NCBI, https://www.ncbi.nlm.nih.gov/sra)^15,16,34,39–41^ revealed pronounced expression of these genes specifically in developing oat spikes and grains (Fig. S20 and Supplementary Data 1, Table S8.5). This tissue-specific expression pattern provided additional theoretical support for their potential utility in oat breeding programs.

## Discussion

The population structure and distribution patterns of hulled and naked oats exhibit high complexity, arising from the interplay between geographic isolation and gene flow, as well as the balance of artificial and natural selection^13,14^. In addition, extensive structural variations (SVs) from oat allohexaploid evolutionary processes^15,16,42^, including recurrent Chr1A-Chr1C translocations^21,27,43^, significantly influence population differentiation and gene flow^27^. This study revealed that global oat genetic architecture features a G2-centered gene flow network coexisting with moderate differentiation (Fig. 1j, k and S3 and S5 and Supplementary Data 1, Table S2.5 and S3.2). This pattern arises from historical isolation and selection shaping an isolation-by-distance framework, where geographic proximity increased haplotype sharing while regional pressures reinforced lineage boundaries-exemplified by G3 (naked oats) experiencing stronger domestication bottlenecks than G2 (hulled oats), manifested as phenotypic divergence (G3: high protein/fat; G2: yield advantages) (Fig. S12 and Supplementary Data 1, Table S5.5). In addition, modern breeding has broken down geographic barriers via widespread introduction and interspecific hybridization, with G4 (North American hulled elites) serving as a “bridge population” that maintained diversity (*π* = 2.07 × 10⁻⁶) while potentially accumulating favorable alleles for yield and adaptability (Figs. 1g, h and S12 and Supplementary Data 1, Table S5.5). These mechanisms inform naked oat breeding via: exploiting favorable alleles from bridge populations (e.g., G4) and employing pan-genome strategies to aggregate traits from diverse ecotypes (e.g., Mediterranean yield potential with Chinese naked oat quality).

Furthermore, integrated analysis of population structure, gene flow, and genetic differentiation reveals that naked oats originated from a key divergence during the eastward dispersal of Mediterranean hulled oat landraces, undergoing independent domestication and diversification in China. This conclusion is supported by multiple lines of genetic evidence from globally representative samples. First, 26.8% of Chinese naked oat landraces exhibit genetic admixture with Eurasian hulled oats (Fig. 2a, b and Supplementary Data 1, Table S4.1), and ancient gene flow plus long shared haplotypes between GⅣ and hulled subpopulations (Fig. 2d–f and Supplementary Data 1, Table S4.2) indicate non-isolated differentiation. Second, GⅡ (Mediterranean hulled oats) is the ancestral lineage with the highest diversity (Fig. 2g). Significant gene flow occurred from GⅡ to GⅢ (Central/Eastern Eurasian hulled) and then to GⅣ (Chinese naked) (Fig. 2e, f and Supplementary Data 1, Table S4.2), with *d*_xy_ and fastsimcoal2 confirming the closest GⅢ-GⅣ affinity (Fig. 2i–k and Supplementary Data 1, Table S4.3). Population demographic analyses reveal that the effective population size of GⅡ (*N*_e_ = 969) is lower than model predictions (Fig. 2j), which does not contradict its ancestral status inferred from nucleotide diversity and other evolutionary indices. Nucleotide diversity reflects long-term accumulated ancient genetic variation, whereas contemporary *N*_e_ only captures recent demographic dynamics, and the two metrics are not intrinsically correlated. As observed in numerous crop species, progenitor populations in domestication centers often exhibit reduced *N*_e_ under environmental and anthropogenic pressures, while derived lineages commonly show expanded effective population sizes accompanied by subsequent gene flow^44,45^. Multiple independent lines of evidence support the ancestral position of GⅡ, including its highest nucleotide diversity, most ancient allelic lineages, basal phylogenetic placement, and demographic simulation results (Fig. 2d–k and Supplementary Data 1, Tables S4.2, S4.3). Its reduced *N*_e_ is likely attributable to a recent population contraction within its native Mediterranean range. Severe founder bottlenecks are typically associated with genetic drift that may obscure genuine selection signals. Despite experiencing an extreme bottleneck (*N*_e_ ≈ 187), genome-wide scanning and population differentiation analyses demonstrate that the genetic divergence and phenotypic specialization of GⅣ were predominantly driven by directional artificial selection. Specifically, nucleotide diversity in GⅣ is 47.88% lower than that in GⅢ, with heterogeneous genomic distribution of this diversity loss (Fig. 2g and Supplementary Data 1, Table S4.2). GⅣ also harbors a higher proportion of genomic windows with negative Tajima’s *D* values and an elevated proportion of long runs of homozygosity (1 Mb–3 Mb ROH: 67.52% in GⅣ vs. 10.91% in GⅢ) (Figs. 2d and S9b and Supplementary Data 1, Table S4.2), consistent with strong directional selection superimposed on underlying bottleneck effects. A total of 1,179 GⅣ-specific selected genes identified via integrated screening were significantly enriched in amino acid and lipid metabolic pathways (*q* < 0.01) (Figs. S21, S22 and Supplementary Data 1, Table S4.6), and overlapped with previously reported QTLs underlying grain β-glucan content, crude protein content and drought tolerance, consistent with the phenotypic characteristics of the GⅣ subpopulation (Fig. S23; Supplementary Data 1, Table S4.6). Furthermore, the divergence time of GⅣ from GⅢ at approximately 6,124 generations ago (~6,000 BP) coincides closely with the eastward dispersal of West Asian cereal crops^44,46,47^. Archaeological remains of charred naked oat grains from northern China (4,000–3,500 BP) and historical records of naked oat cultivation dating to the Northern Wei Dynasty further validate this molecular evolutionary timeline^48–53^. Collectively, integrated genetic, archaeological, and historical evidence corroborates northern China as the secondary domestication center of global naked oats. These findings delineate a continuous differentiation gradient from the Mediterranean to the Central and Eastern Eurasian and then to Chinese naked oats (Fig. 2k).

These findings align with Loskutov’s hypothesis, which posited that naked oats arose from mutations in cultivated hulled oats and were later domesticated in China^25^, but contrast with Nan et al. ^26^, who suggested Chinese naked oats may have diverged from wild hulled oats prior to domestication, followed by independent domestication. Bekele et al.^27^ inferred from 7D-H4 haplotype distribution that Turkish hulled oats and Chinese naked oat landraces likely originated independently from weedy species such as *A. fatua*. Fine-scale Chr7D haplotype analysis with NC_GBS data^27^ supported naked oat evolution in the present study. First, the 7D-H3 haplotype dominates Chinese naked oats (55%), matching Eurasian hulled oat patterns^27^, indicating derivation from the mainstream hulled lineage characterized by the 7D-H3 haplotype. Second, the 7D-H4 haplotype, despite high frequency in North China naked landraces (30%), appears in only 1 in 23 Turkish hulled landraces, arguing against a common independent origin. Furthermore, 7D-H4 accessions show coordinated phenotypes: tall (152.23 cm), late heading (~10 d), increased protein (19.6%) and fat (6%) (Fig. S7 and Supplementary Data 1, Tables S2.2, S4.1 and S4.5), this combination of “tall stature, late maturity, and high nutritional quality” aligns with the requirements of traditional grain-forage dual-purpose systems in Northern China. Additionally, selection signals in the Chr7D inversion (256.6–412.1 Mb) in naked oats (Fig. 4a, b and Supplementary Data 1, Table S6.2) suggest local adaptation roles. Notably, naked grain loci resided on 4D^14,15,21,26^. Thus, the Chr7D inversion is more likely associates with adaptive traits, not the naked phenotype directly. However, as seen in origin studies of rice^54^, maize^55^, wheat^56^, and grape^57^, resolving oat domestication requires multidisciplinary evidence. Future work with broader geographical sampling, higher-density genomic data (including structural variations), archaeological evidence, and transitional materials will further clarify this history. By elucidating naked oat population structure and domestication history, the present study establishes the critical foundation for harnessing their genetic potential and accelerating cultivar improvement.

Integrating population genomics, multi-environment phenotyping, and transcriptomes, we showed that differentiation between hulled and naked oats reflects divergent domestication pressures rather than neutral drift. Several lines of evidence supported this. First, clear trade-offs between yield and quality traits, particularly between the G2 and G3 core subpopulations (Fig. S12), mirror the separation seen in both molecular and phenotypic PCAs (Figs. 1d and S11; Supplementary Data 1, Tables S1.3 and S5.4). Second, naked oats carried stronger bottleneck signatures and directional selection signals across 17 chromosomes (Fig. S13 and Supplementary Data 1, Table S2.1), with selected genes functionally enriched for nutritional quality and stress response^18,21^ (Fig. 4b and Supplementary Data 1, Tables S6.4–S6.6). Third, they exhibit divergent transcriptional programs during seed development and stress responses (Figs. S15, S16 and Supplementary Data 1, Table S7). By overlapping selection signals with transcriptomic data, we pinpointed 127 genes that are both under selection and differentially expressed; 33 of these fall within known QTL intervals (Fig. 5 and Supplementary Data 1, Table S7.6). Together, these results established a direct link between historical selection, transcriptional regulation, and phenotypic divergence, offering a prioritized candidate list for oat breeding. We acknowledge that broader developmental sampling and experimental validation were needed to fully elucidate gene functions and their regulatory networks. Furthermore, three functional genes identified in our study associated with oat yield QTLs^32,33^ identified in this study supply directly applicable genetic resources for bridging the yield gap and maximizing nutritional value. The loss of superior haplotypes (*AsUMAMIT25*-Hap2: A & *AsSAG12*-Hap2: C) in most naked oat accessions correlated with yield limitations, while their rare retention in northern Chinese landraces offered valuable germplasm for targeted yield enhancement (Fig. 6 and Supplementary Data 1, Table S8.1). Therefore, the elite *AsAHL1*-Hap2 haplotype, which demonstrated broad environmental adaptability among 77 hulled oats, offered a valuable resource for optimizing environmental resilience and yield stability in naked oats. The three selected gene-derived haplotypes in naked oats, significantly associated with superior yield and quality traits, provide genetic targets for bridging the yield and nutritional gap in oats.

## Methods

### Materials

666 hexaploid oat germplasm collected from 65 countries and regions worldwide (659 with previously published GBS data^15^; 7 newly generated in this study) (Fig. 3a,S1 and Supplementary Data 1, Table S1-1), including 293 bred varieties (227 hulled oats and 66 naked oats), 366 landraces (270 hulled oats and 96 naked oats), and 7 wild hulled oats used in this study. Among the 162 naked oats, 146 were from China, distributed across 16 provinces (Supplementary Data 1, Table S1-1). We selected 9 hulled oats and 13 naked oats of diverse geographical origins for RNA-seq analysis (Fig. 1e, d and Supplementary Data 1, Table S1.4).

### Genetic model analysis of phenotypic traits across multiple environments

Phenotypic variations of 12 growth and development traits, 5 grain yield-related traits and 2 grain quality traits measured on test materials across multiple environments were fitted with a mixed linear model: *y* = *Xβ* + *Z*_*g*_*g* + *Z*_*ge*_*ge* + *ε*. Here, *y* represents the vector of phenotypic observed values, *β* denotes the fixed effects including environment and experimental replication, *g* is the random genotypic effect, *ge* refers to the random genotype × environment interaction effect, and *ε* is the random residual effect; *X*, *Z*_*g*_ and *Z*_*ge*_ are the corresponding design matrices. The likelihood ratio test (LRT-*P*) and Bayesian information criterion (BIC) were used to screen the optimal genetic model: LRT-*P* verified the significance of random effects by calculating LRT = −2(log*L*_simplifiedmodel_ − log*L*_fullmodel_), while BIC was applied to select the optimal non-nested model via the formula BIC = −2log*L* + *k*logn (where *k* is the number of parameters and n is the sample size)^58,59^. Furthermore, based on the genotypic variance (*V*_*g*_), genotype × environment interaction variance (*V*_*ge*_) and residual variance (*V*_*ε*_) estimated by the optimal genetic model, the broad-sense heritability (*H*^2^) of each trait was calculated using the formula *H*^2^ = *V*_*g*_/(*V*_*g*_ + *V*_*ge*_/*E* + *V*_*ε*_/(*R* × *E*)), in which *E* is the number of environments and *R* is the number of replications in a single environment.

### Phenotypic investigation and analysis

In accordance with the Manual of *Description and Data Specifications for Oat Germplasm Resources*, we conducted field investigations on 17 agronomic traits, with five biological replicates for each trait. These traits and their abbreviations are as follows: heading stage (HS), growth period (GP), spike type (ST), spikelet type (SLT), effective tiller number (ET), plant height (PH), spike length (PL), flag leaf length (LFL), flag leaf width (WFL), stem diameter (SD), node of number (NN), awn presence (AWN), spikelet number (SN), Hulled/Naked (H/N), grains per spike (GN), thousand-grain weight (TGW), grain length (GL), grain width (GW), and grain circumference index (GCI). Additionally, plump seeds were selected, dried, and ground for analysis. Total crude protein content (CFC) was measured using a Dumas rapid nitrogen analyzer (Rapid N Exceed, Elementar, Germany), while crude fat content (CPC) was determined via the Soxhlet extraction method (EE, GB/T 6433-2006), with three biological replicates for each measurement. After assigning values to the descriptive traits, we conducted descriptive statistical analysis, correlation analysis, and dimensionality reduction on the phenotypic data using IBM SPSS Statistics 27.0. Meanwhile, based on the genotypic and phenotypic data of the test materials, the genome-based restricted maximum likelihood (GREML) bivariate model in GCTA software (v1.0)^60^ was used to construct a genetic relationship matrix (GRM) with genome-wide SNP data, simultaneously estimate the additive genetic variances and covariances for pairwise traits, and calculate the genetic correlations, which reflects the degree of phenotypic correlation between traits attributable to shared genetic factors.

### DNA extraction, GBS library construction and sequencing

The oat materials tested were planted in a row-planting pattern at Huihe Base of Sichuan Agricultural University, Wenjiang District, Chengdu, Sichuan, China. For each material, genomic DNA was extracted from leaves of 10 three-week-old seedlings using the MiniBEST Plant Genomic DNA Extraction Kit (Cat# 9768, TaKaRa, Japan). DNA concentration and quality were validated using agarose gel electrophoresis and a Nanophotometer (Thermo Fisher), ensuring ratios within acceptable ranges (A260/A280 > 1.8; A260/A230 = 1.8–2.2). DNA library construction followed the protocol described by Poland^61^. Briefly, genomic DNA was fragmented using EcoRI and MseI as restriction enzymes. Digested fragments were ligated to sequencing adapters and barcodes for sample multiplexing. After normalizing the concentration and uniformity of digested products, adapter-linked fragments were amplified by PCR. PCR products were purified to remove primer dimers and invalid short fragments, and 375–400 bp DNA fragments were recovered via electrophoresis for cluster preparation. GBS library quality, including fragment distribution, purity and effective concentration, was tested with Agilent 2100 Bioanalyzer and Qubit fluorometer to meet sequencing criteria. Qualified libraries were normalized, pooled for clustering and sequenced on the Illumina HiSeq 2000 platform. Raw reads were trimmed of adapters and filtered for low-quality bases to obtain clean reads for downstream analysis. Novogene (Beijing, China) performed all library construction, QC and sequencing following standard protocols.

### SNP calling

After obtaining the raw sequencing data, paired sequences containing adapter sequences were removed. Single-end reads with more than 10% undefined bases (*N*) relative to the total sequence length were discarded. Additionally, single-end reads with a quality score (*Q*) ≤ 5 and more than 50% of bases below this threshold were also removed. The resulting clean data were used for SNP calling. SNP calling was performed using the GATK (Genome Analysis Toolkit, v4.4.0)^62^. First, BWA-MEM (v0.7.8)^63^ was used to align the clean data to the hexaploid cultivated oat reference genome of Sanfensan^15^ with parameters set as mem -t 4 -k 32 -M. Next, SAMtools (v1.16.1)^64^ was used to sort the alignment results in SAM format, convert them to BAM format, and remove PCR duplicates. We then performed variant calling using HaplotypeCaller to generate per-sample gVCFs, followed by GenomicsDBImport to merge them, and culminating in joint genotyping across all samples using GenotypeGVCFs (v2.4-9)^65^, yielding the raw VCF file. The filtering criteria for SNP markers were as follows: loci with base quality below 10 and sequencing depth less than 30 were excluded, as were loci with more than two alleles (retaining only biallelic loci). Additionally, loci with a minor allele frequency (MAF) ≤ 0.05 and greater than 10% missing data were removed. Considering oats are strictly self-pollinating, loci with heterozygosity exceeding 10% were also excluded. The remaining variant loci were used for further analysis.

### Transcriptome sequencing

A total of 22 samples from different developmental stages of both naked and hulled oats were collected, including roots, stems, and leaves at the seedling stage; leaves and spikelets at the booting stage; and spikelets at the heading, flowering, and filling stages, for total RNA extraction. The RNA extraction kit (Catalog No.: DP432) was supplied by Beijing Tiangen Biochemical Technology Co., Ltd. After passing quality control, equal amounts of RNA from each tissue were pooled and sent to Wuhan Hope Genomics Co., Ltd. for mRNA library construction. Polyadenylated mRNA was specifically enriched from total RNA using Oligo dT magnetic beads, and the purified mRNA was randomly fragmented into short fragments under high-temperature conditions. The fragmented mRNA was used as a template for reverse transcription to synthesize first-strand cDNA, followed by second-strand cDNA synthesis. Subsequently, end repair, A-tailing, and sequencing adapter ligation were performed on the double-stranded cDNA fragments. Adapter-ligated cDNA fragments were selectively purified and PCR-amplified to construct the final mRNA library. Prior to sequencing, the constructed libraries were subjected to quality inspection to verify fragment size distribution and effective concentration. Qualified libraries were normalized and pooled to generate sequencing clusters. Finally, high-throughput transcriptome sequencing was performed on the Illumina HiSeq 2000 platform to obtain raw sequencing data for subsequent bioinformatics analysis.

### Analysis of differentially expressed genes

We integrated 38 transcriptome datasets: 16 publicly available from NCBI^15,16,34–38^, and 22 generated for this study. Public datasets included 7 seed development datasets (4 hulled, 3 naked oats)^15,16,34^, 7 saline-alkali stress datasets (3 naked, 4 hulled oats)^35,36^ and 2 drought stress datasets (naked oats)^37,38^. All datasets underwent quality control (QC) using Fastp (v0.23.4)^66^, ensuring normal distribution of sequencing quality scores (average Q30 ≥ 80%, error rate ≤ 0.1%) and GC content, plus removal of adapter-contaminated reads. Post-QC clean reads were directly subjected to gene and transcript quantification using Salmon (v0.8.2)^67^ based on three oat reference genomes: Sanfensan^15^, Sang^16^ and OT3098v2^17^. The quantification results were merged and converted into standardized gene expression matrices using Kallisto2Matrix (v1.0.0)^68^. Differentially expressed genes (DEGs) were screened via edgeR (v1.6.15)^69^ with FDR-corrected *P* < 0.05 and |log_2_Fold Change| > 1.5. Only genes with TPM > 1 (used for expression quantification) were considered for DEG selection. The identification pipeline for DEG sets across all projects was as follows: for bulk RNA-seq data, hulled oats were designated as the control group and naked oats as the treatment group to identify DEGs between the two types. For single-project RNA-seq data, blank controls or early developmental stages were used as references, with DEGs first identified for all other developmental stages and treatment conditions. Subsequently, for the same project, DEGs from multiple single-project datasets of hulled oats and naked oats were intersected separately; genes with consistent differential expression (e.g., both upregulated or downregulated during seed development) in both oat types were then excluded. Ultimately, the hulled oat-specific or naked oat-specific DEG sets were obtained for subsequent analyses.

### Population structure analysis

The VCF file was first converted into Plink-format (.bed,. bim,. fam) files, filtering out loci with linkage disequilibrium (*r*² > 0.2) using the parameters: --indep-pairwise 50 10 0.2^70^. ADMIXTURE^71^ software (v1.3.0) was then used to determine the optimal *K* value by testing values from 2 to 20, followed by the analysis of population structure at the genome-wide level. A phylogenetic tree was constructed using the neighbor-joining method in MEGA X^72^, and its visualization was enhanced using the Chiplot website (https://www.chiplot.online/). Principal component analysis (PCA) was performed with GCTA (v1.0)^60^ software, and the results were visualized using Chiplot for data presentation.

### Calculation of linkage disequilibrium half-decay distance

The LD half-decay distance was derived from 127,563 high-quality SNPs using *r*^2^. Analysis involved: (1) filtering SNPs (MAF ≥ 0.05, missing ≤ 0.2); (2) calculating intra-chromosomal pairwise *r*^2^ with PLINK (v1.07)^73^; (3) grouping *r*^2^ by distance and fitting an exponential decay model. The half-decay distance was defined as the point where *r*^2^ fell to 50% of its peak value.

### Genetic diversity analysis

We used BCFtools (v1.3)^74^ to extract and convert VCF subfiles for each subpopulation. Nucleotide diversity (*π*), Tajima’s *D* neutrality test, and the genetic differentiation index (*F*_ST_) among distinct oat subpopulations were computed using VCFtools (v0.1.16)^70^. The calculations were performed with a sliding window approach, using a window size of 500 kb and a step size of 50 kb, and intergroup differences in nucleotide diversity between hulled and naked oats were assessed using the Wilcoxon rank-sum test (Mann–Whitney U test), with a significance threshold set at *P* < 0.05. Population differentiation was classified using the classic criteria of Wright^75^ and Hart & Clark^76^: *F*_ST_ < 0.05 for negligible differentiation, 0.05 ≤ *F*_ST_ ≤ 0.15 for weak-to-moderate differentiation, and *F*_ST_ > 0.15 for high differentiation, *F*_ST_ > 0.25 for very strong genetic differentiation. Moreover, we used pixy v1.2.7^77^ to quantify the genetic differentiation among local landrace subpopulations by calculating the absolute nucleotide divergence index (*d*_xy_) and to infer their genetic relationships. The window and step sizes were set as described above, and the software’s default missing data correction parameters were retained to reduce estimation bias. Moreover, we generated a linkage disequilibrium (LD) heatmap for the target genomic region using LDBlockShow^78^.

### Demographic Inference

Demographic inference was performed using fastsimcoal2 (v2.7.0.9)^79^. The joint site frequency spectrum (jSFS) was constructed from 123,756 genome-wide biallelic SNPs using easySFS. Four demographic models were evaluated: strict isolation (SI), isolation with migration (IM), secondary contact (SC), and isolation with initial gene flow (IIF). Parameter priors included effective population size (100–1,000,000), divergence time (100–100,000 generations), and migration rate (1 × 10⁻⁹ – 1 × 10⁻² per generation). For each model, we performed 100 independent runs, each with 100,000 coalescent simulations and up to 40 ECM optimization cycles. The best-fitting model was selected using the Akaike Information Criterion (AIC), and 95% confidence intervals for core parameters were obtained from 100 parametric bootstrap replicates.

### Gene flow analysis

To analyze gene flow among hexaploid cultivated oat groups based on 127,563 high-quality SNPs. First, runs of homozygosity (ROH) were identified using the --homozyg module in Plink v1.9 (interval: 10–3000 kb, matching the LD decay of oat genome) to evaluate conserved haplotypes. Second, we used Dsuite (v0.4)^80^ for ABBA-BABA (*D*-statistic) analysis to directly test and quantify gene flow intensity, employing an outgroup model. Significant gene flow was defined by |*D*| > 0, *Z* > 1.96, and *P* < 0.05, with proportions estimated via the *f*_4_-ratio. Third, Treemix (v1.13)^81^ inferred the historical direction and number of gene flow events. A phylogenetic tree was rooted with the group with the highest genetic diversity, and the OptM package in R software was adopted to determine the optimal number of migration events (*m*) via a likelihood ratio test (LRT), which was also used to verify the reliability of gene flow events.

### Genome-wide sweeping analysis

The cross-population composite likelihood ratio test (XP-CLR)^82^ quantifies genomic allele frequency differentiation between target and reference populations to detect selection signals by fitting a skewed model of population allele frequencies. In this study, hulled oats served as the reference and naked oats as the target population for XP-CLR score calculation, with parameters set to -window 500 kb, -step 50 kb, -minsnps 20, and others default. Meanwhile, BCFtools (v1.3)^74^ extracted and reformatted VCF subfiles for 504 hulled and 162 naked oat accessions from the high-quality VCF file; VCFtools (v0.1.16)^70^ (500 kb window, 50 kb step) then calculated their nucleotide diversity (*π*) and fixation index (*F*_ST_). Based on these results, potential selection loci were the top 1% of XP-CLR scores and *π* ratio (*π*_hulled_/*π*_naked_) via the percentile method; for *F*_ST_, loci with *Z*(*F*_ST_) > 2.8 (*P* < 0.001) were the threshold. Subsequently, candidate genes were defined as those within 2 Mb upstream/downstream of each potential selection locus (LD half-decay distance: 2 Mb). Finally, genes detected by at least two methods were identified as the naked oat selected gene set for subsequent analysis.

### Identification of the Chr7D inversion haplotype

To resolve the Chr7D inversion haplotype in the tested materials, we performed an analysis based on public NC_GBS data (Bekele^27^) and a custom SNP-informed 31-mer pipeline^83^. First, high-quality SNPs (missing rate < 10%, heterozygosity < 10%) were filtered from the NC_GBS VCF file to construct SNP-centered 31-mers and a corresponding BED file. Specific k-mers were then obtained by aligning these sequences using BWA. Subsequently, k-mer counting was performed separately on the raw GBS reads from this study and on the NC_GBS data (based on DP values from the VCF) to generate count matrices. Genotypes were called based on allele frequency: homozygous reference (*r* ≤ 0.1), homozygous alternative (*r* ≥ 0.9), and heterozygous (intermediate values), followed by calculation of sample heterozygosity and homozygosity. Next, the genotype matrices from NC_GBS and this study were merged, and their concordance was evaluated by calculating correlation coefficients, identity-by-state (IBS) statistics, and the homozygous discordance rate. Finally, the R function seq_NN was used for genotype matching to identify the most genetically similar samples between the NC_GBS dataset and our materials, thereby determining the distribution of the Chr7D haplotype across 591 samples.

### GO and KEGG enrichment analysis

Functional enrichment analyses (GO and KEGG) for selected genes and differentially expressed genes of naked oats, along with queries for gene structure and functional annotation, were performed using the Oat Biological Information Database (OatBioDB; http://www.waooat.cn). The custom code for all the above analyses has been publicly archived in Zenodo at https://doi.org/10.5281/zenodo.20697674^83^.

### Development and genotyping of KASP molecular markers

Using the Oat Biological Information Database (OatBioDB; http://www.waooat.cn), sequence alignment, extraction, and primer specificity testing (all corresponding analytical code is derived from publicly released third-party tools integrated within the database^84,85^) were performed based on the G/A single nucleotide polymorphism (SNP) site located 23,230 base pairs (bp) upstream of the *AsUMAMIT25* gene. Detailed information for the developed KASP markers is provided in Supplementary Data 1 (Table S8.3a). Primer dimer formation and hairpin structure prediction were analyzed using DNAMAN software to ensure primer compatibility, and Primer3 Input (v0.4.0) was used to automatically design matching forward/reverse primers based on existing sequences. KASP genotyping was conducted using Mix reagents from Beijing Jiacheng Biotechnology Co., Ltd. The KASP primer mix was prepared by combining 60 µL of each forward primer, 150 µL of the reverse primer, and 230 µL of ddH₂O in a 1.5 mL centrifuge tube, followed by thorough mixing. This primer mix was then applied to 268 hexaploid cultivated oat accessions for DNA genotyping validation.

### qRT-PCR

Quantitative real-time PCR (qRT-PCR) was performed to detect the transcript abundance of three oat grain yield-related genes. Total RNA was isolated from oat tissues using the FastPure Universal Plant Total RNA Isolation Kit (Nanjing Vazyme Biotech Co., Ltd.) strictly following the manufacturer’s guidelines. After RNA purification, genomic DNA contamination was removed, and the purified RNA was reverse-transcribed into first-strand cDNA with the HiScript II 1st Strand cDNA Synthesis Kit (+gDNA wiper, Nanjing Vazyme Biotech Co., Ltd.). Gene-specific forward and reverse oligonucleotide primers were custom-designed for the three target genes *AsUMAMIT25*, *AsAHL1* and *AsSAG12*, and the full sequences of all primer pairs are summarized in Supplementary Data 1, Table 8.4b. The qRT-PCR amplification reaction was prepared using ChamQ Universal SYBR qPCR Master Mix (Nanjing Vazyme Biotech Co., Ltd.), with the synthesized cDNA serving as the amplification template. The oat actin gene was used as the endogenous reference gene to calibrate relative gene expression values, and all thermal cycling procedures were executed as recommended by the kit manufacturer.

### Statistics and reproducibility

All field and molecular experiments were conducted using standard protocols with independent biological replicates to ensure reproducibility. Five biological replicates were used for phenotypic assessment of 17 agronomic traits, and three biological replicates were applied for grain quality detection. A total of 666 hexaploid oat accessions were used for population genomic analysis, and 22 multi-tissue samples were used for transcriptome sequencing. All statistical analyses were performed with consistent criteria using IBM SPSS 27.0. The Wilcoxon rank-sum (Mann–Whitney U) test was used for intergroup comparisons, and differences with *P* < 0.05 were considered statistically significant. LRT, BIC, and AIC were applied for genetic and demographic model optimization. DEGs were identified based on FDR-corrected *P* < 0.05, |log₂FC| > 1.5, and TPM > 1. High-quality SNPs were filtered by unified criteria (MAF ≥ 0.05, 10%–20% missing rate, heterozygosity < 10%). All sequencing and molecular analyses were performed with standardized pipelines and consistent parameters to ensure reliable and reproducible results.

### Reporting summary

Further information on research design is available in the Nature Portfolio Reporting Summary linked to this article.

## Supplementary information

Supplementary Information

Description of Additional Supplementary Files

Supplementary Data 1

Supplementary Data 2

Reporting Summary

Transparent Peer Review file

## Data Availability

The 666-accession GBS dataset comprises 659 previously published samples (BioProject PRJNA807126^15^). Sequencing data newly generated in this study, including 7 additional GBS accessions and pooled multi-tissue RNA-seq from 22 samples, have been deposited in NCBI SRA under BioProject PRJNA1478309 and PRJNA794002, respectively. External public transcriptome datasets were downloaded from NCBI SRA and ENA. All datasets used in this study can be retrieved from corresponding repositories via their BioProject IDs. The 16 transcriptome datasets used for analyzing transcriptional regulatory divergence between hulled and naked oats include: seed development (PRJNA101123, PRJNA1089357, PRJNA727473^15^, PRJEB46365^16^, PRJNA932293^34^), saline-alkali stress (PRJNA354579^35^, PRJNA355375, PRJNA916004^36^, PRJNA957850, PRJNA1135772, PRJNA1434555), drought stress (PRJNA764537^37^, PRJNA1111847^38^), as well as transcriptome datasets for expression pattern analysis of *AsUMAMIT25*, *AsAHL1* and *AsSAG12* in oat vegetative and reproductive organs (spikelets and seeds): PRJNA727473^15^, PRJEB46365^16^, PRJNA957844, PRJNA932293^34^, PRJNA930136^39^, PRJNA1047126^40^, PRJNA564861^41^. Source data are provided as Supplementary Tables, contained in Supplementary_Data_1.xlsx (Tables S1–S8) and Supplementary_Data_2.xlsx (Tables S9–S11). The source data for each chart and graph in the main figures are specified in the corresponding figure legends.

## Code availability

All custom analysis code for this study has been permanently archived and publicly released on Zenodo (https://doi.org/10.5281/zenodo.20697674)^83^, including scripts for OatBioDB-based GO/KEGG enrichment analysis and DNA sequence extraction, as well as code for the independent SNP-informed 31-mer pipeline that does not rely on the OatBioDB platform.
