# Supplementary material for: Investigation of naked oat (Avena sativa ssp. nuda) domestication provides insights into yield and nutritional gaps in oats
Source: Commun Biol. 2026 Jul 11;9:1453. doi: 10.1038/s42003-026-10626-w (PMC13737007; doi:10.1038/s42003-026-10626-w)
Supplement: Supplementary file 1 [file 42003_2026_10626_MOESM1_ESM.pdf]

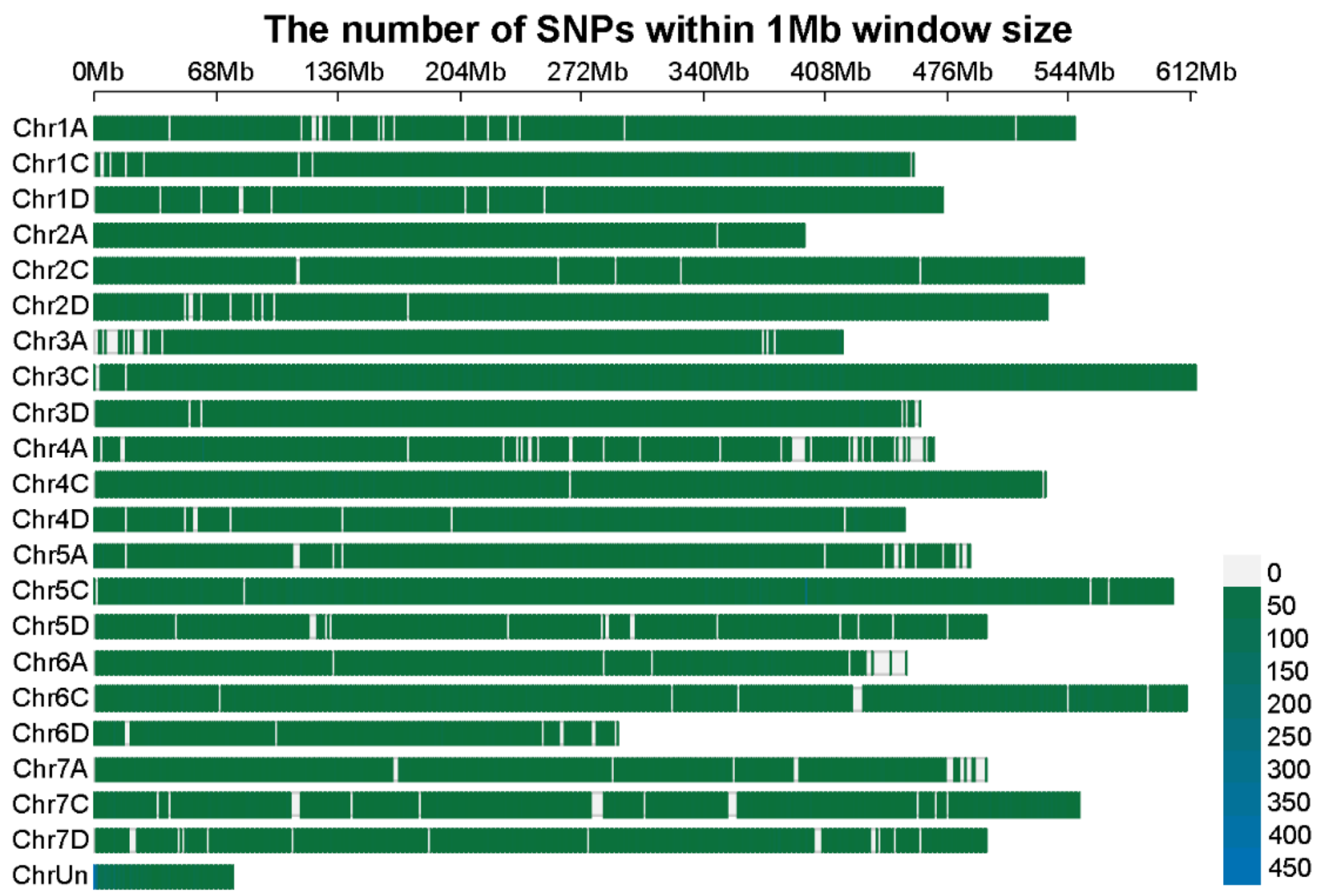

Supplementary Figure 1. Genome-wide distribution density map of 127,563 SNPs

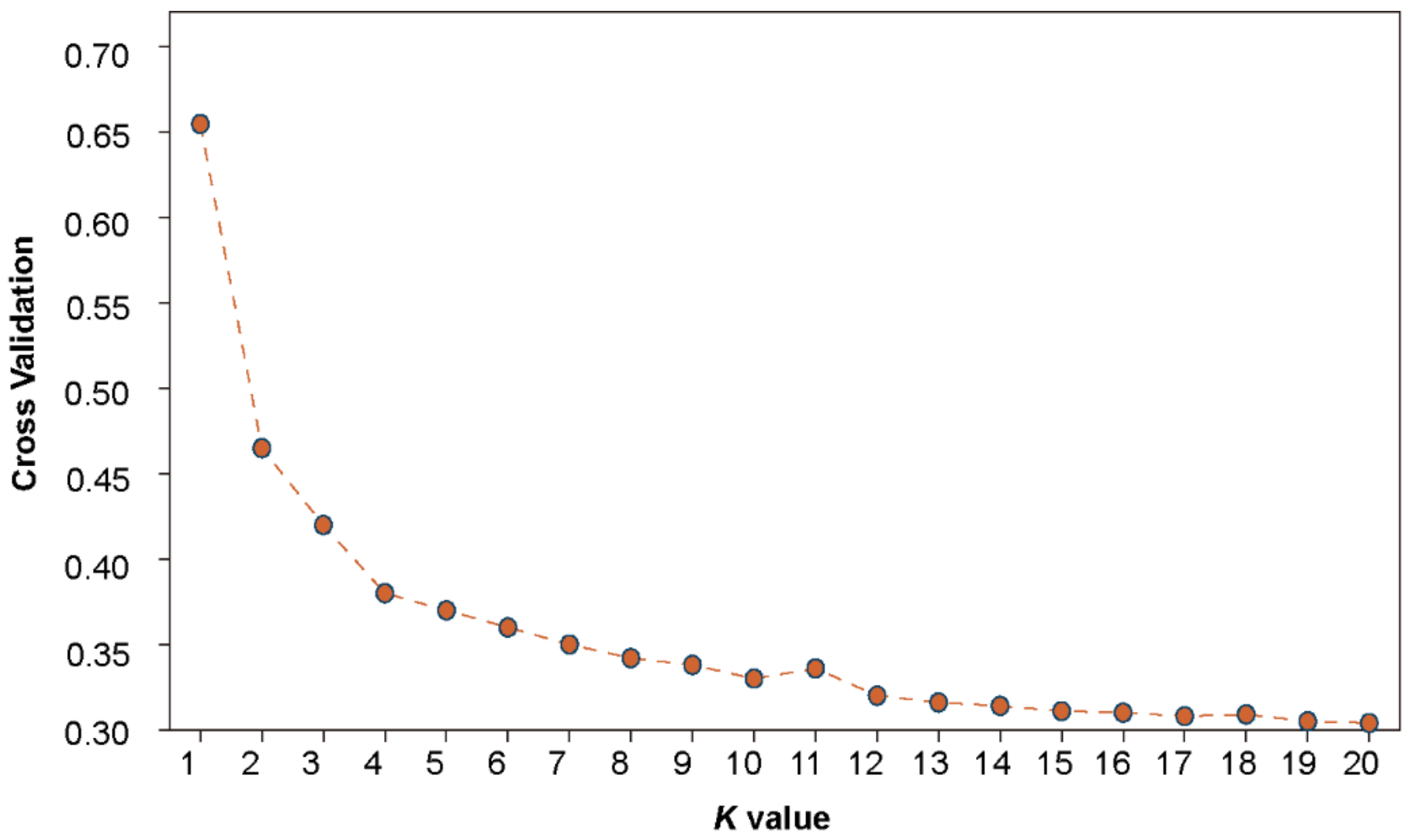

Supplementary Figure 2. Admixture cross-validation (CV) error plot for 666 oats (*K* = 2–20)

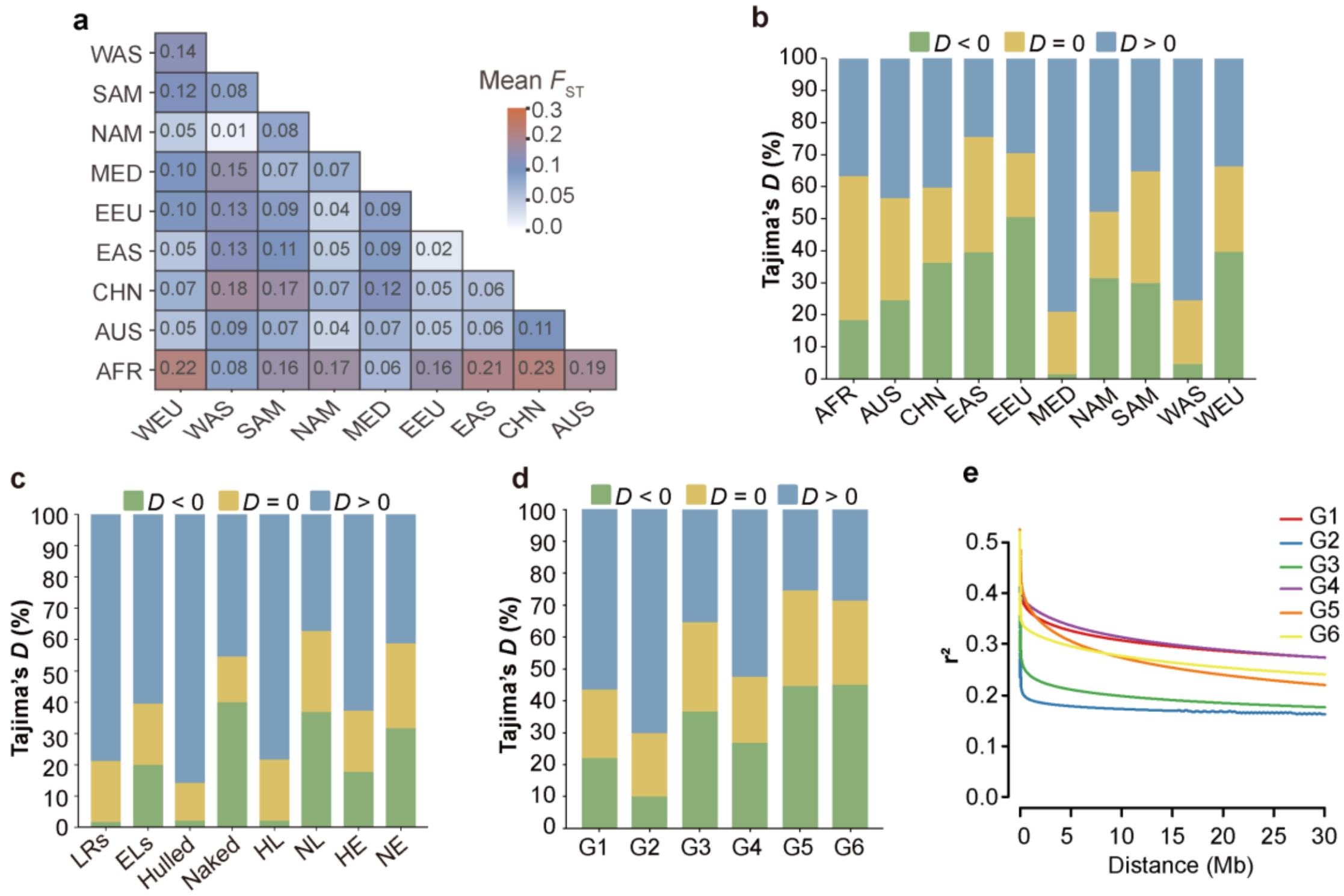

Supplementary Figure 3. Genetic diversity analysis of the 666 tested oat accessions. (a) Heatmap of F_{ST} values among oats from different geographic subpopulations; (b－d) Tajima's *D* neutral test values of oats across different germplasm types, clustered subpopulations and geographic regions; (e) LD decay curves of G1－G6.

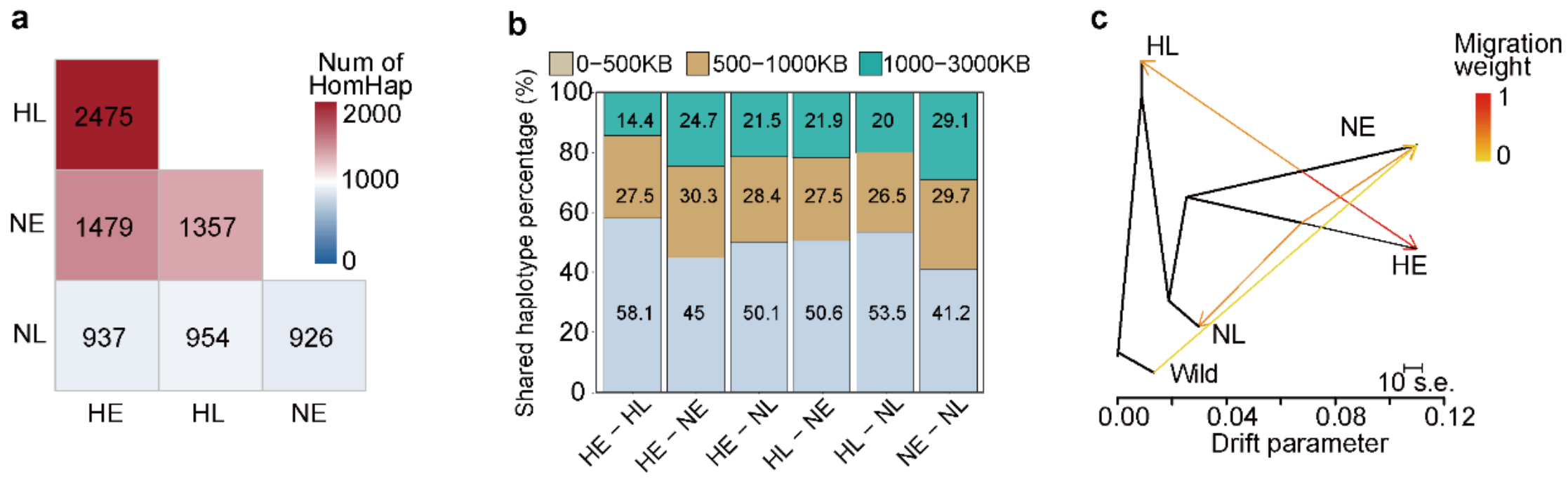

Supplementary Figure 4. Analysis of shared homozygous haplotypes between hulled and naked oats (10 kb–3000 kb). (a, b) Number and length distribution of haplotypes shared between hulled and naked oat landraces and elites；(c) Gene flow analysis and topological tree construction for hulled oats, naked oat landraces and elites based on Treemix software.

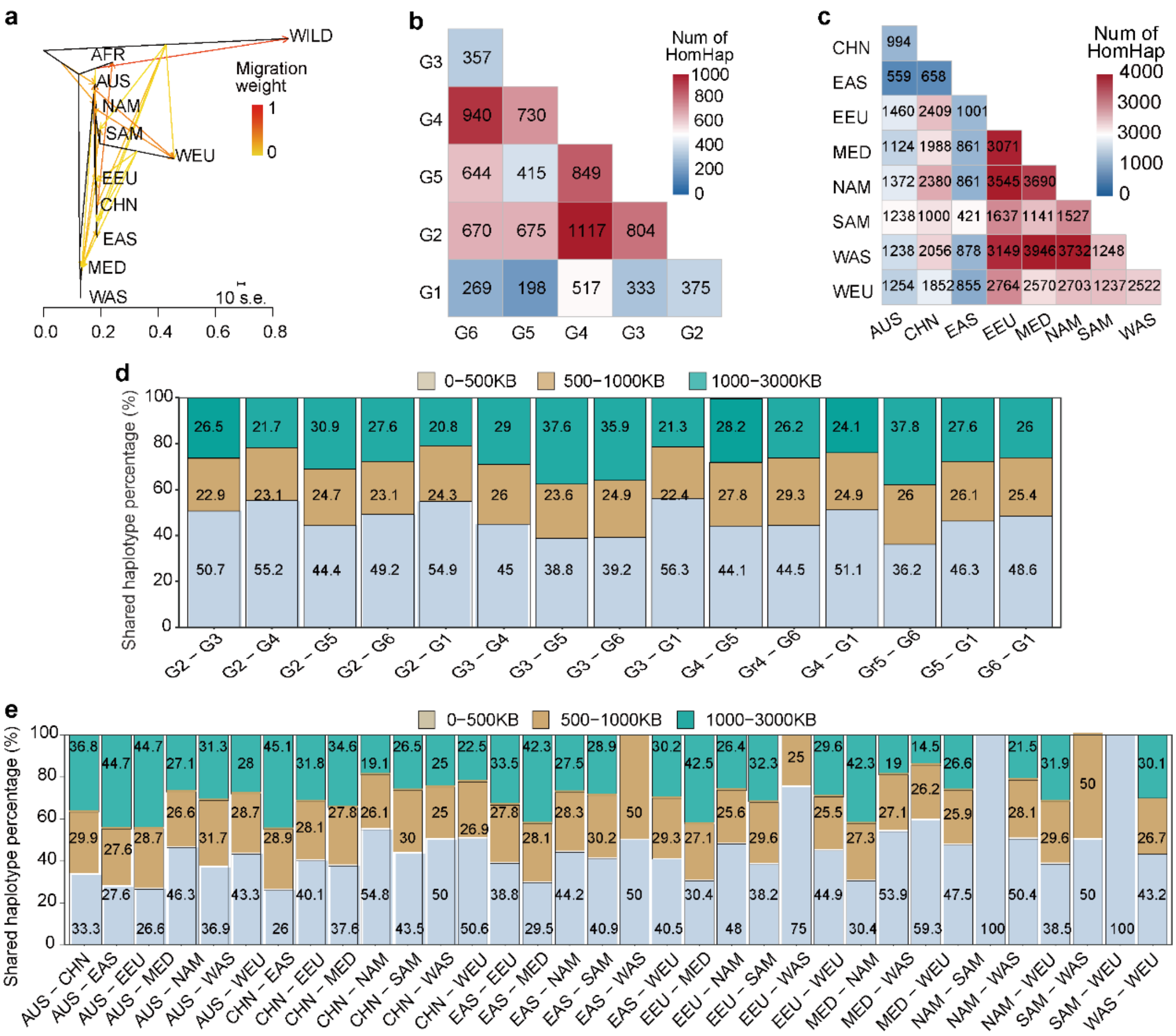

Supplementary Figure 5. Analysis of gene flow and shared homozygous haplotypes among different clustered subgroups and accessions from diverse geographical origins (10 kb–3000 kb). (a) Gene flow analysis and topological tree construction for oats from different geographic regions based on Treemix software; (b, d) Number and length distribution of haplotypes shared across different genetic subgroups; (c, e) Number and length distribution of haplotypes shared across accessions from different geographical regions.

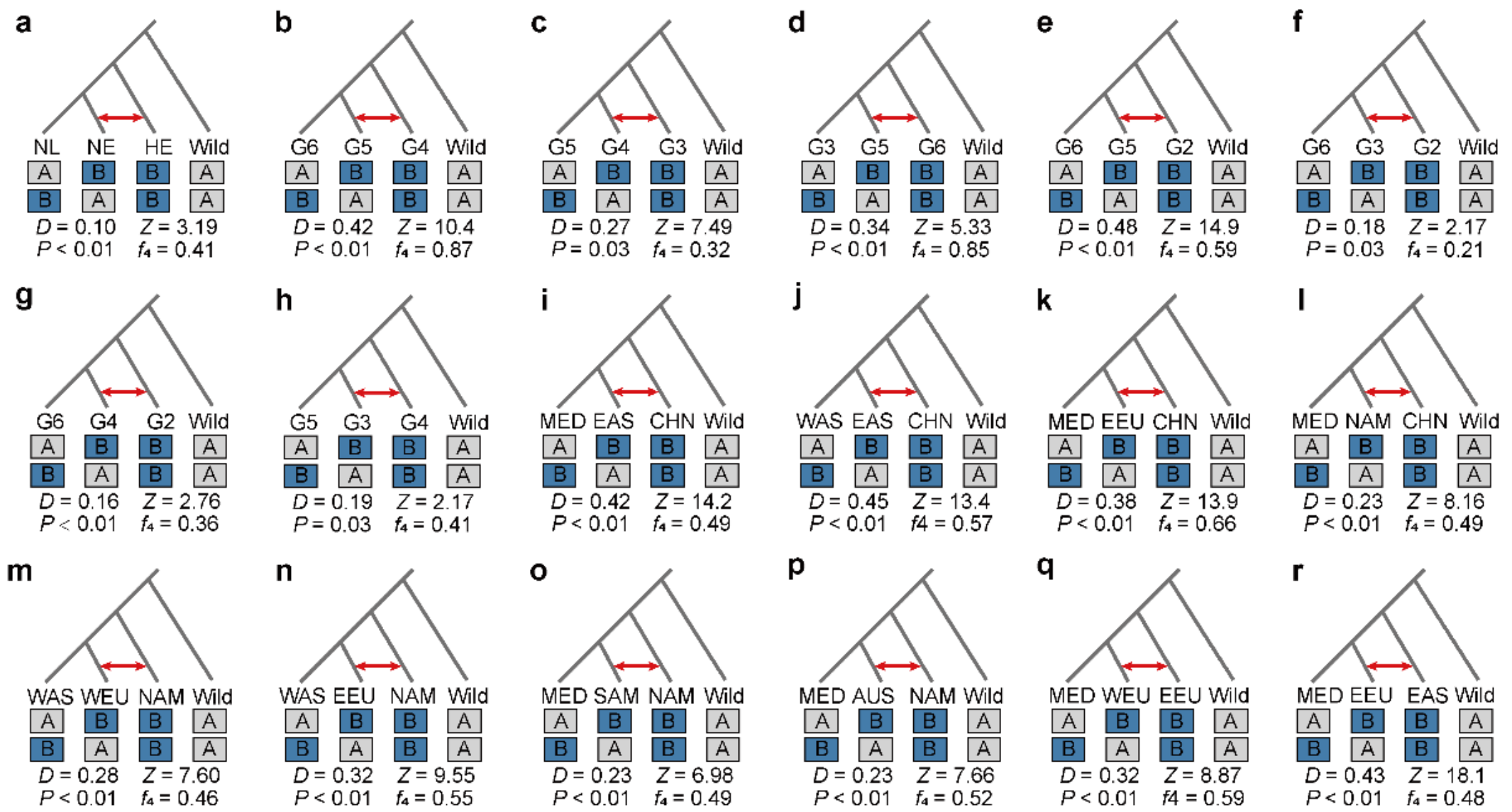

Supplementary Figure 6. *D*-test of gene flow events among hulled and naked oat accessions from different subpopulations and geographic regions based on the ABBA-BABA model.

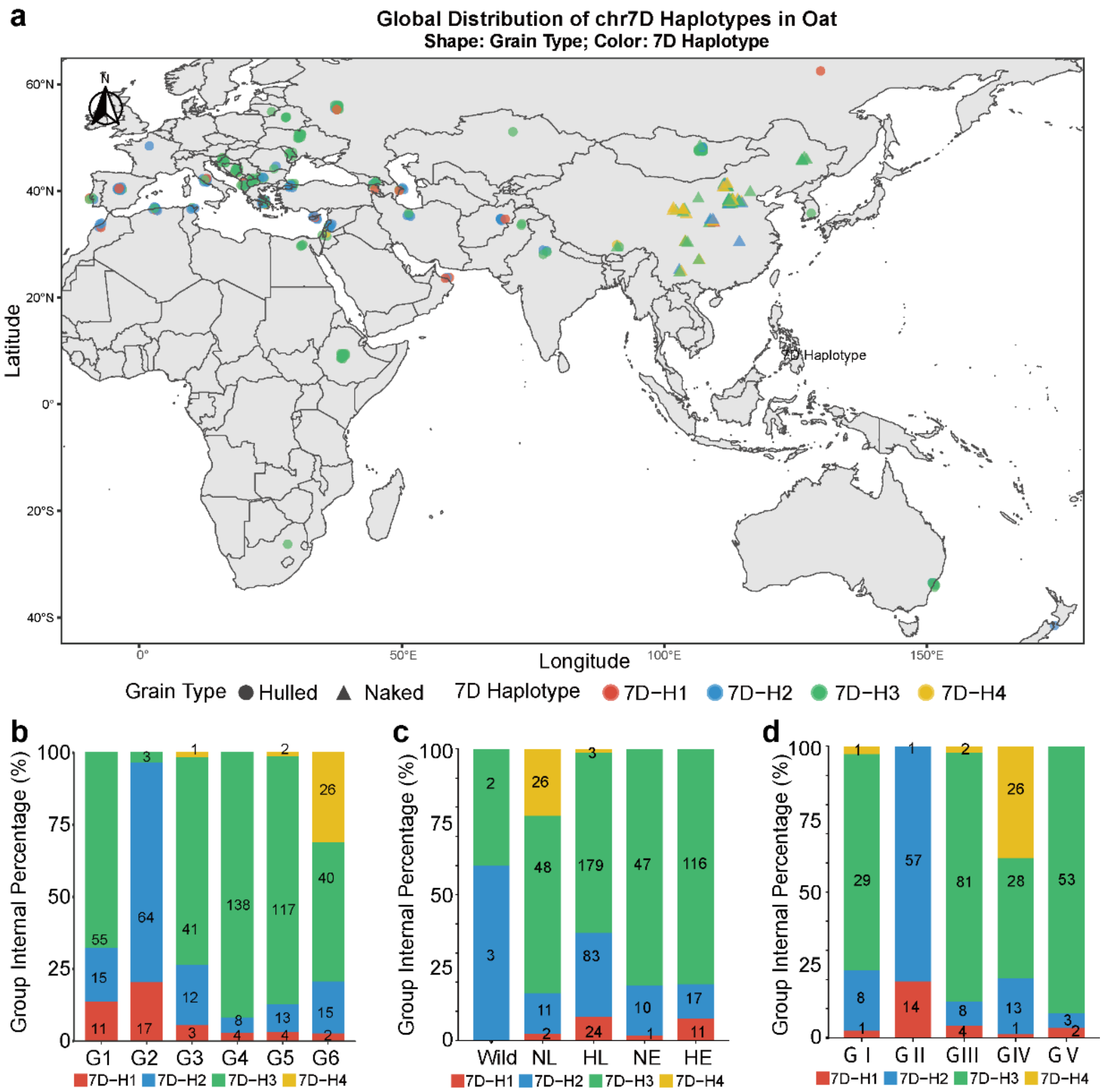

Supplementary Figure 7. Validation and characterization of known Chr7D inversion haplotypes in the present study. (a) Distribution of 7D inversion haplotypes among the 591 analyzed accessions; (b) Proportions of the four haplotypes across the six genetic clusters identified in the entire panel; (c) Haplotype composition in hulled versus naked oat accessions; (d) Haplotype proportions within the five genetic clusters defined among landrace accessions.

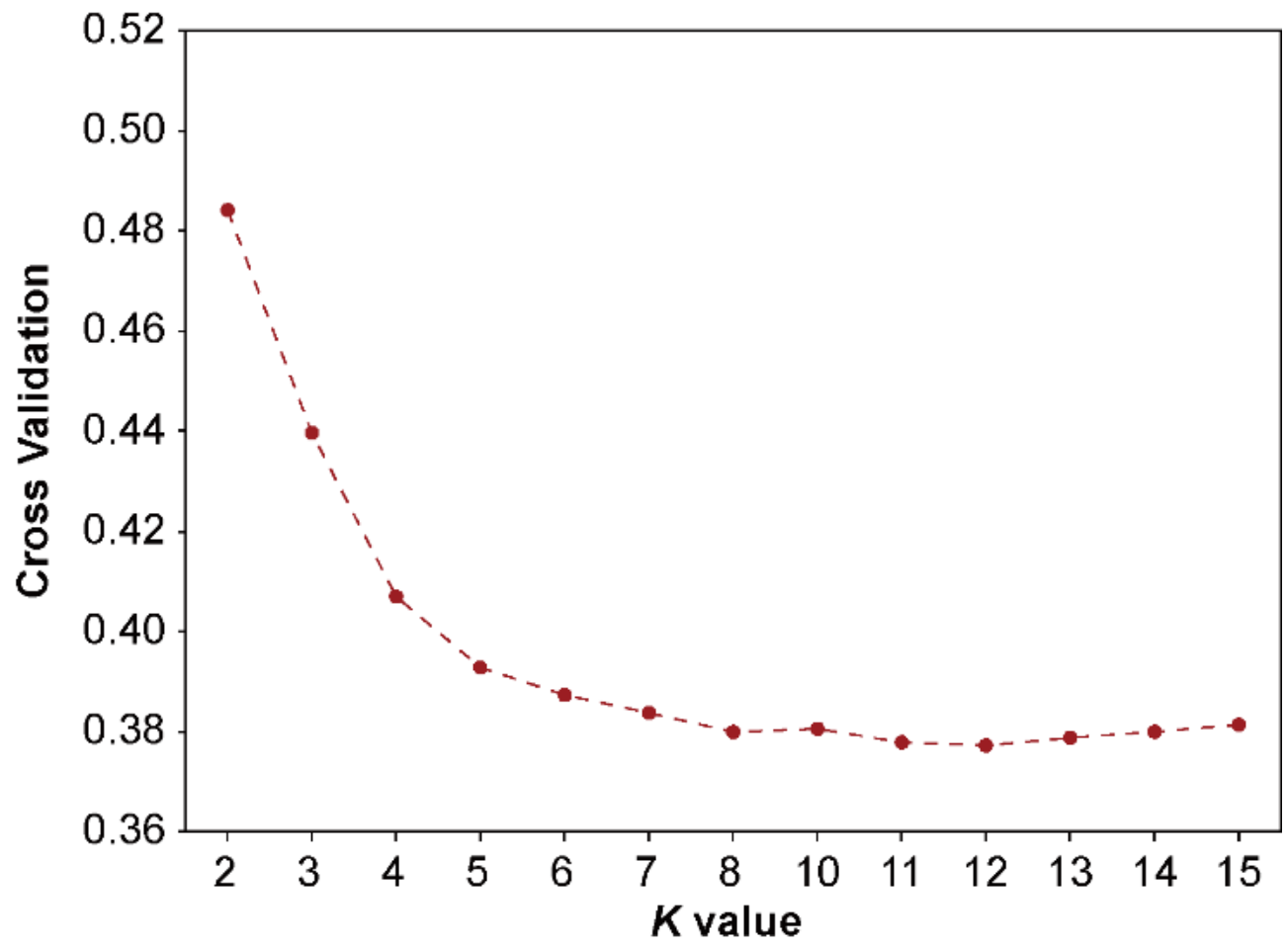

Supplementary Figure 8. Admixture cross-validation (CV) error plot for 366 landraces and 7 wild accessions of hexaploid oat (K = 2–15)

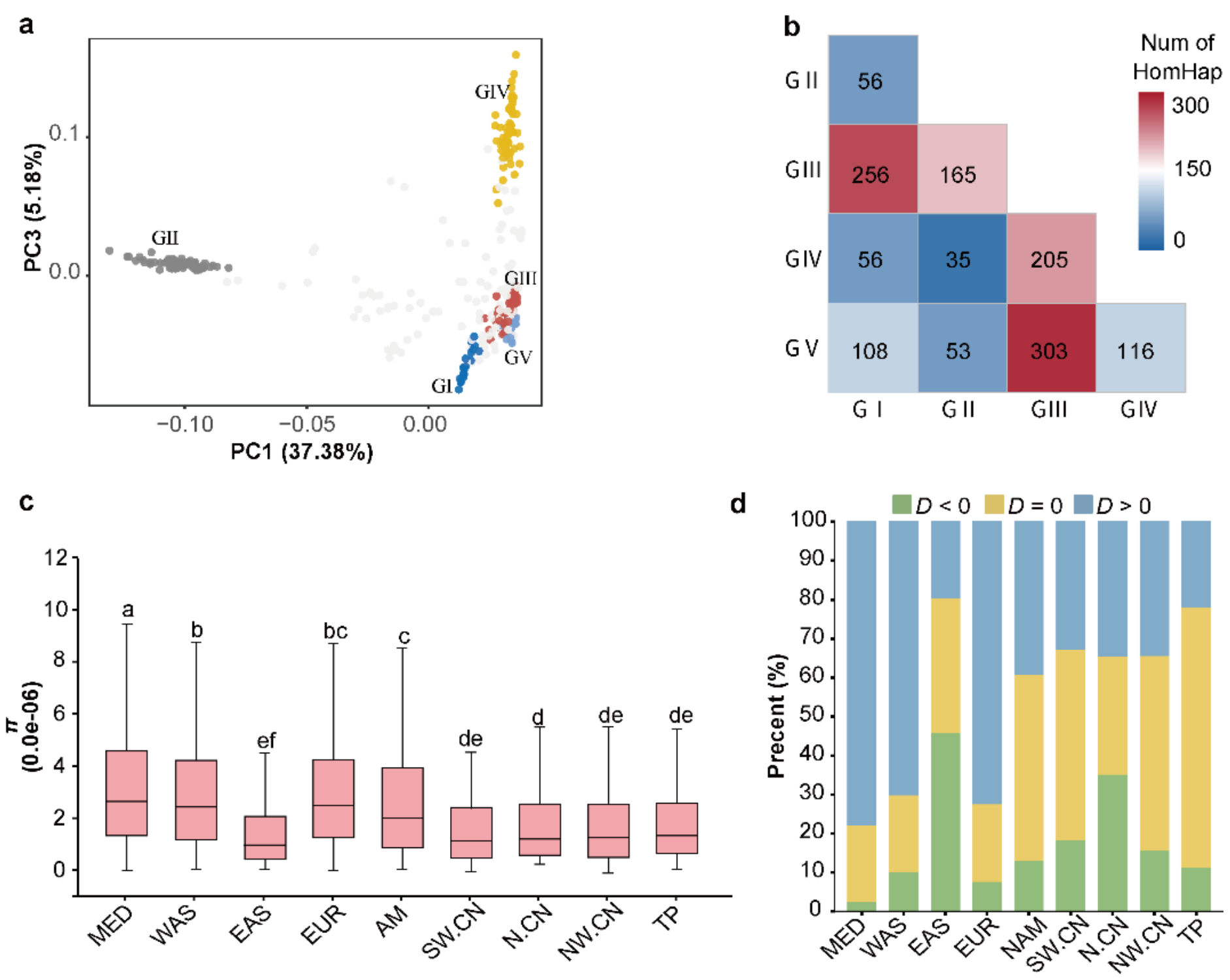

Supplementary Figure 9. Population structure and genetic diversity analyses of 366 landraces and 7 wild accessions of hexaploid oat. (a) Distribution of the 366 landrace and 7 wild accessions along PCA1 and PCA3; (b) Number of shared homozygous haplotypes (10kb–3 Mb) among subgroups; (c) Boxplot comparing average nucleotide diversity (π) among landraces from different geographic groups; (d) Stacked bar plot comparing Tajima's D values among landraces from different geographic groups. Note: SW.CN, Southwest China; NC, North China; NW.CN, Northwest China; TP, Tibetan Plateau.

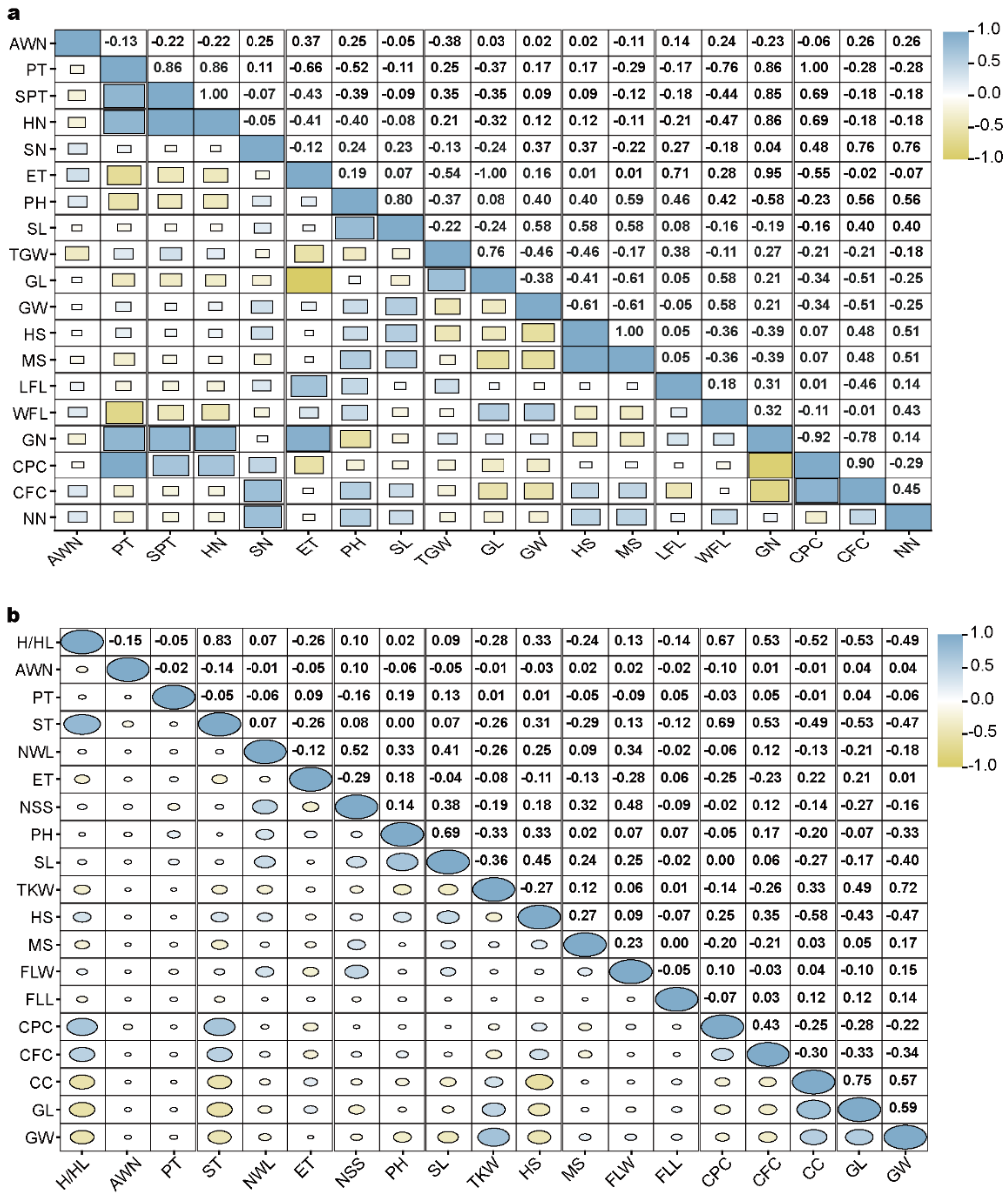

Supplementary Figure 10. Correlation heatmaps of 17 agronomic traits and 2 grain quality-related traits in oat. (a) Heatmap of genetic correlations among traits; (b) Heatmap of phenotypic Pearson correlations among traits.

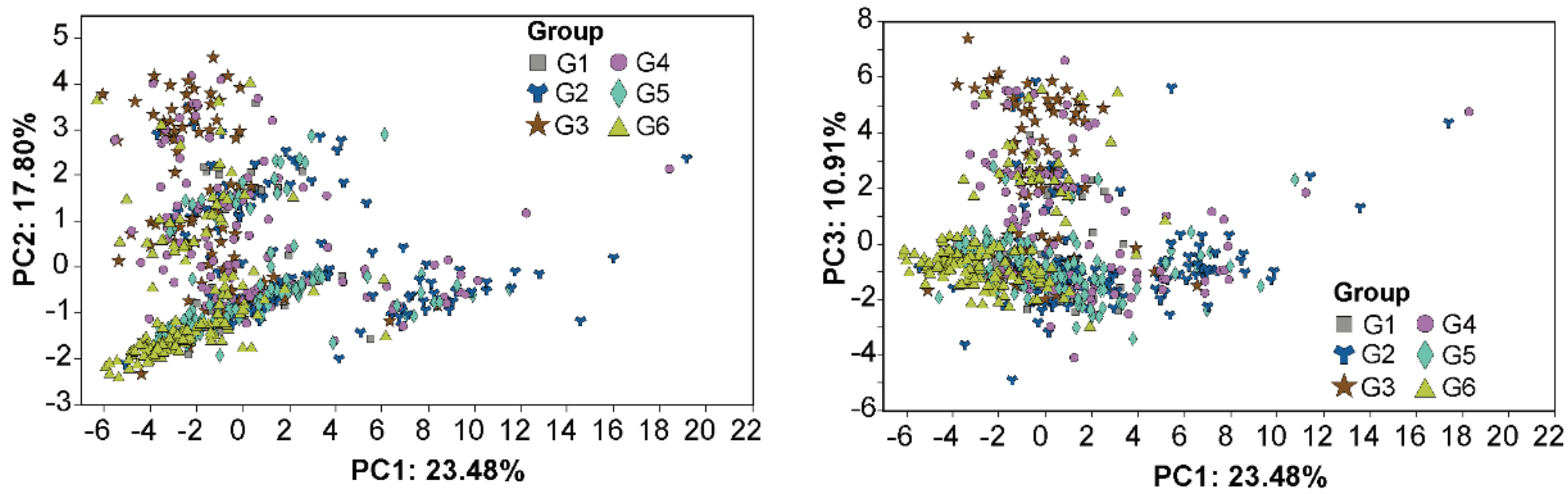

Supplementary Figure 11. Score plot of the first three principal components from PCA based on 19 phenotypic traits in 666 oat accessions.

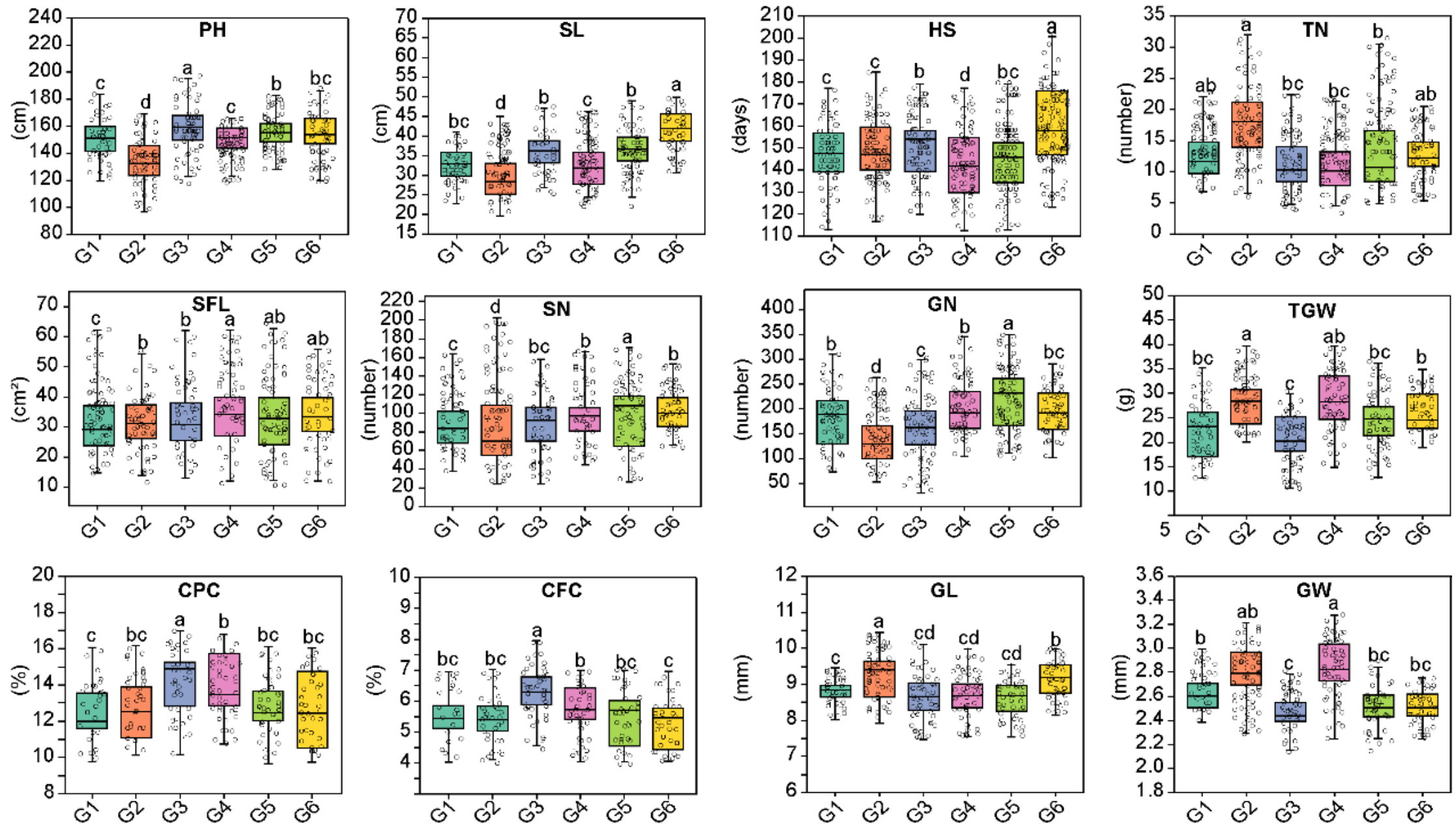

Supplementary Figure 12. Comparative analysis of core phenotypic traits across different genetic subpopulations of 666 oat accessions.

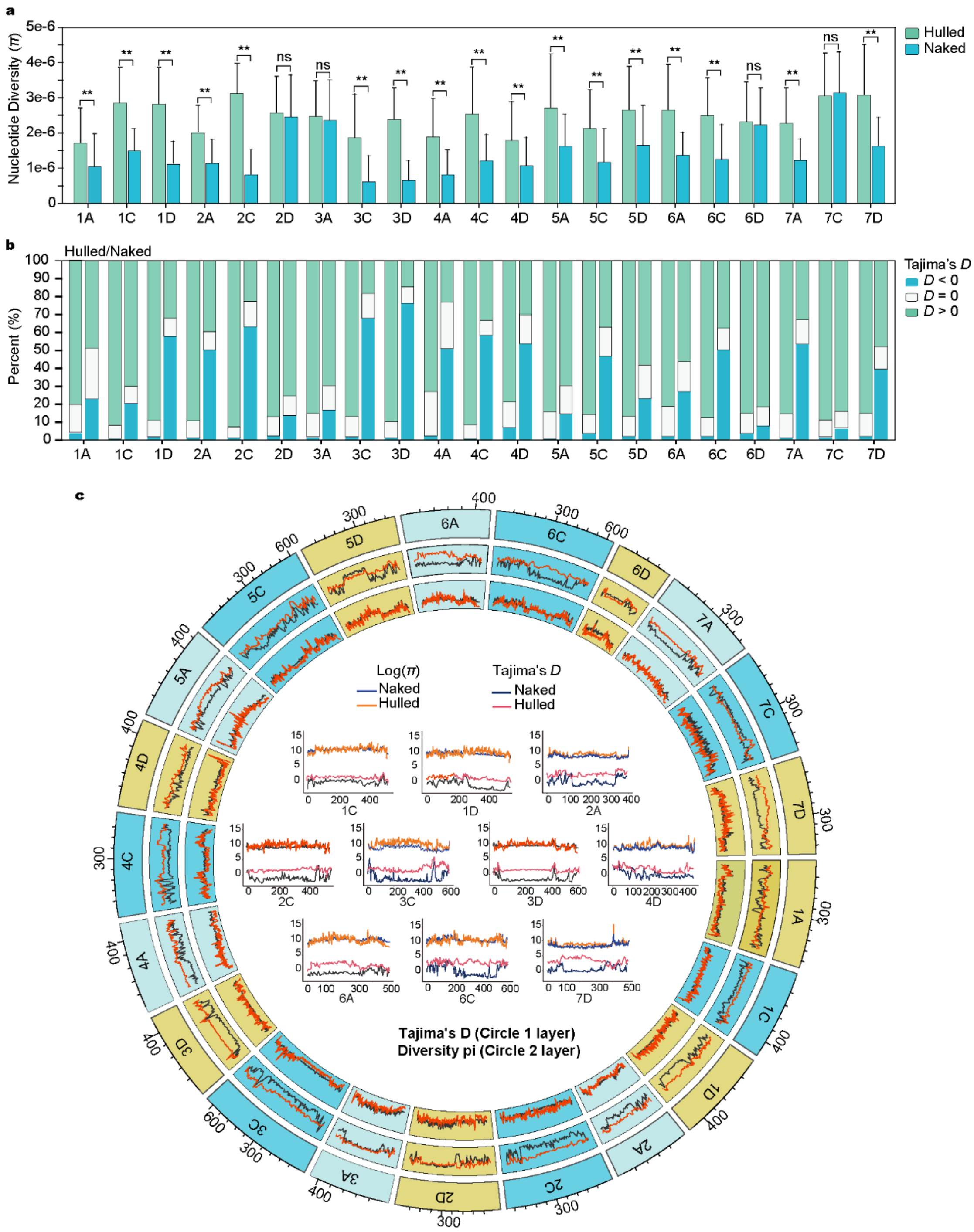

Supplementary Figure 13. Genetic differentiation across chromosomes between hulled and naked oats. (a) Boxplot of nucleotide diversity differences across 21 chromosomes between hulled and naked oats ($P < 0.001$); (b) Selection pressure analysis based on Tajima's D values across 21 chromosomes in hulled and naked oats; (c) Circular plot of nucleotide diversity differences and Tajima's D neutrality test values across 21 chromosomes between hulled and naked oat.

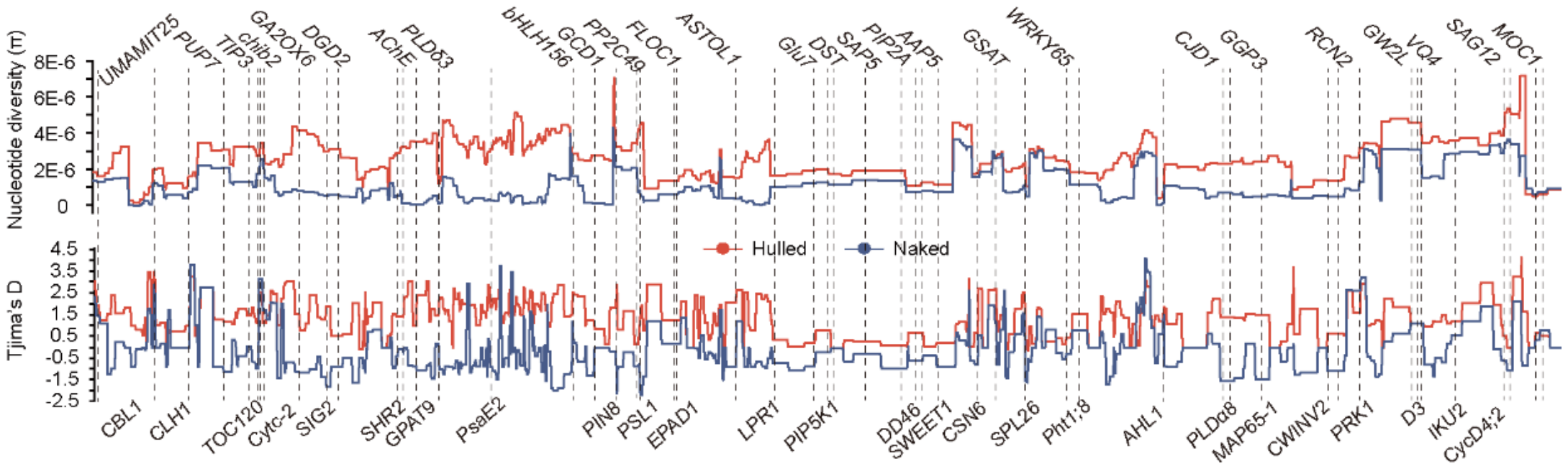

Supplementary Figure 14. Scatter line plot of nucleotide diversity (π) and Tajima's *D* neutrality test values in genomic regions harboring 1504 selected genes between hulled and naked oats.

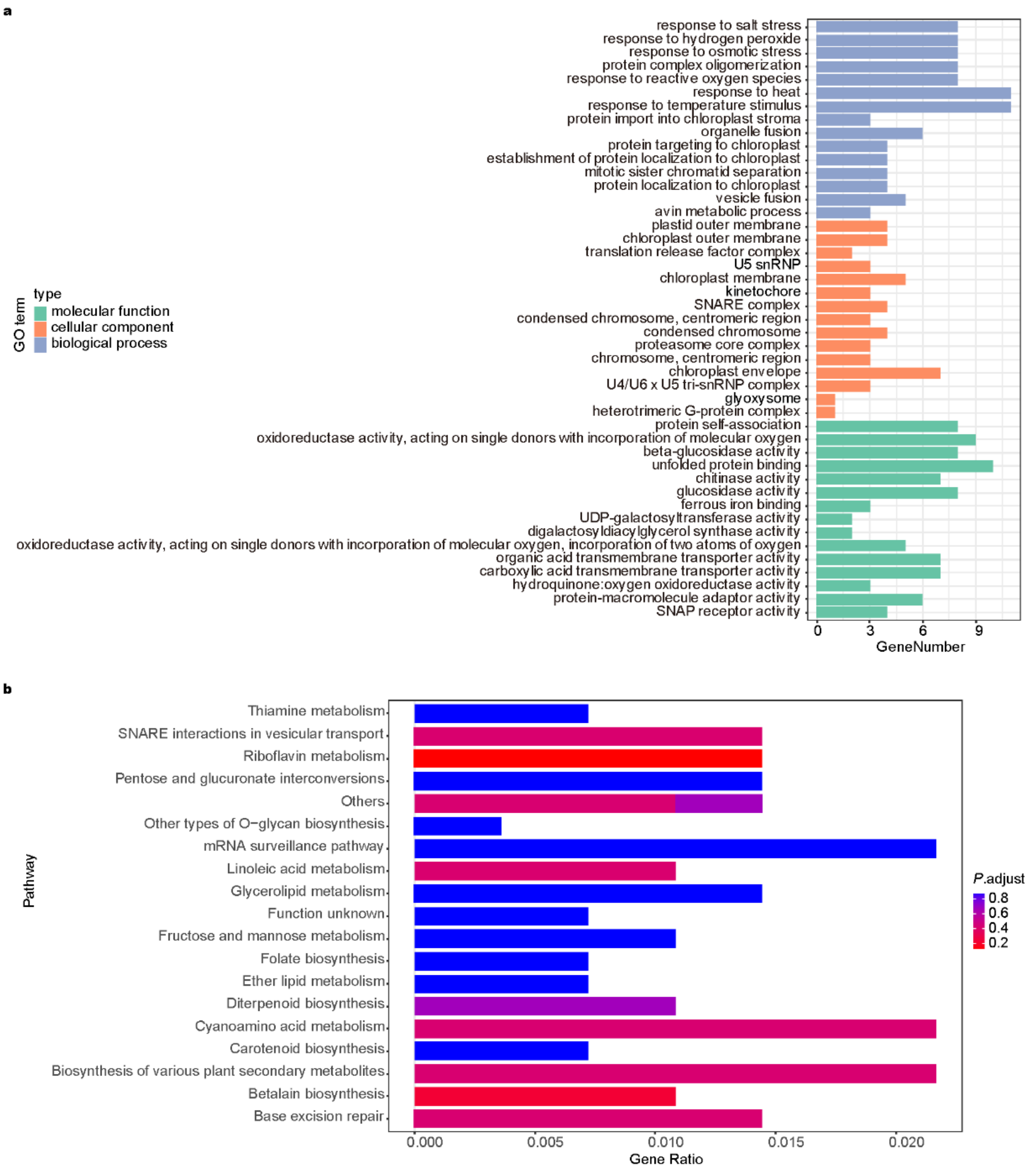

Supplementary Figure 15. Functional enrichment analysis of genes under directional selection during domestication and improvement in naked oats. (a) Top 15 enriched GO terms (Molecular Function, Cellular Component, Biological Process) for directional selection genes (q-value < 0.05); (b) Top 20 enriched KEGG pathways ranked by adjusted P-value.

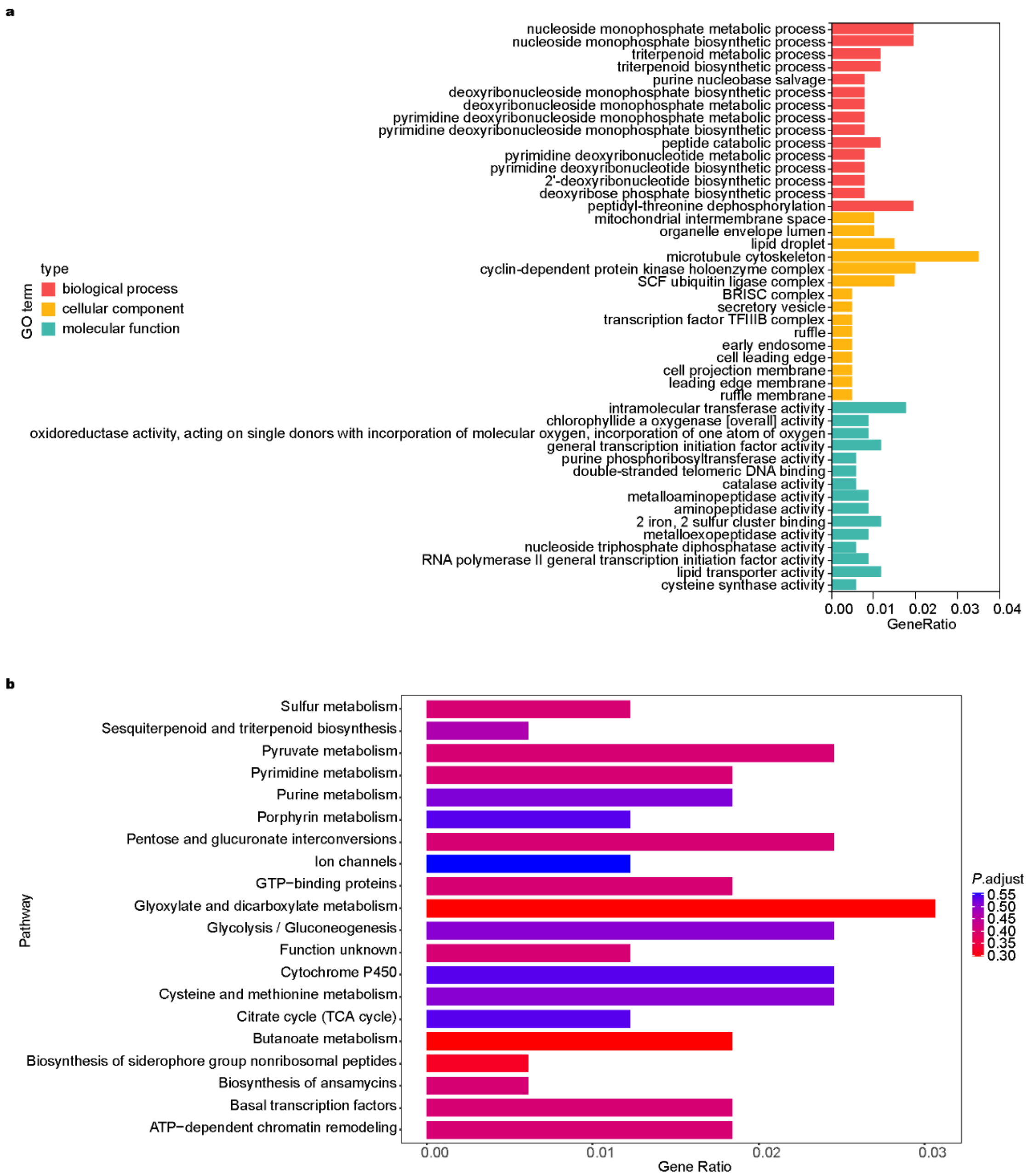

Supplementary Figure 16. Functional enrichment analysis of genes under balancing selection during domestication and improvement in naked oats. (a) Top 15 enriched GO terms (Molecular Function, Cellular Component, Biological Process) for genes under balancing selection (q-value < 0.05); (b) Top 20 enriched KEGG pathways for genes under balancing selection, ranked by adjusted P-value.

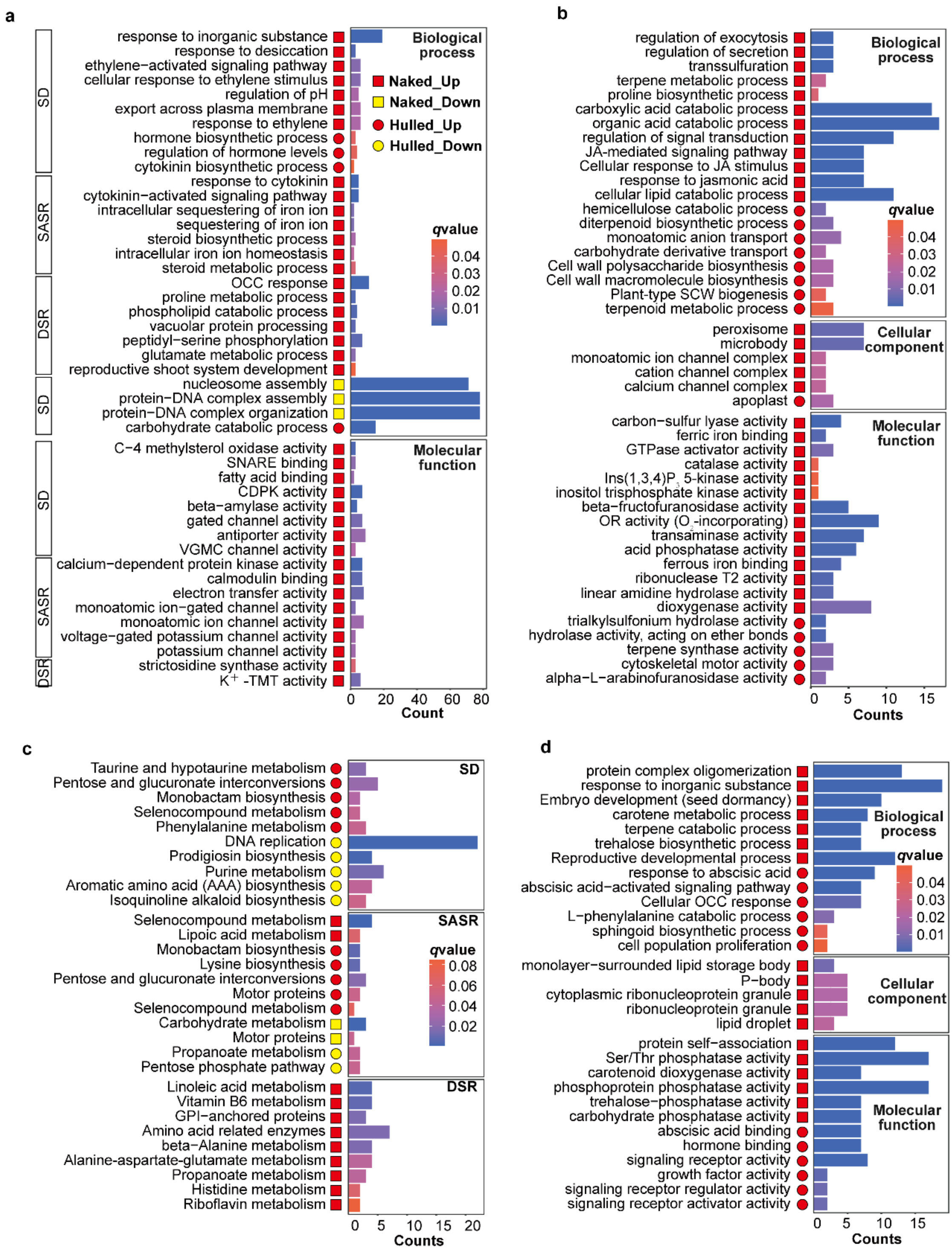

Supplementary Figure 17. Enrichment analysis of shared DEGs across transcriptome datasets and condition-specific upregulated genes in hulled and naked oats (*q*-value < 0.05). (a) Shared GO enrichments for differentially expressed genes identified in both pooled multi-tissue samples and condition-specific transcriptomes; (b) GO enrichment for genes specifically up-regulated during seed development in hulled or naked oats; (c) Shared KEGG enrichments for differentially expressed genes from pooled multi-tissue samples and condition-specific transcriptomes; (d) GO enrichment for genes specifically up-regulated in hulled or naked oats under salt-alkali or drought stress.

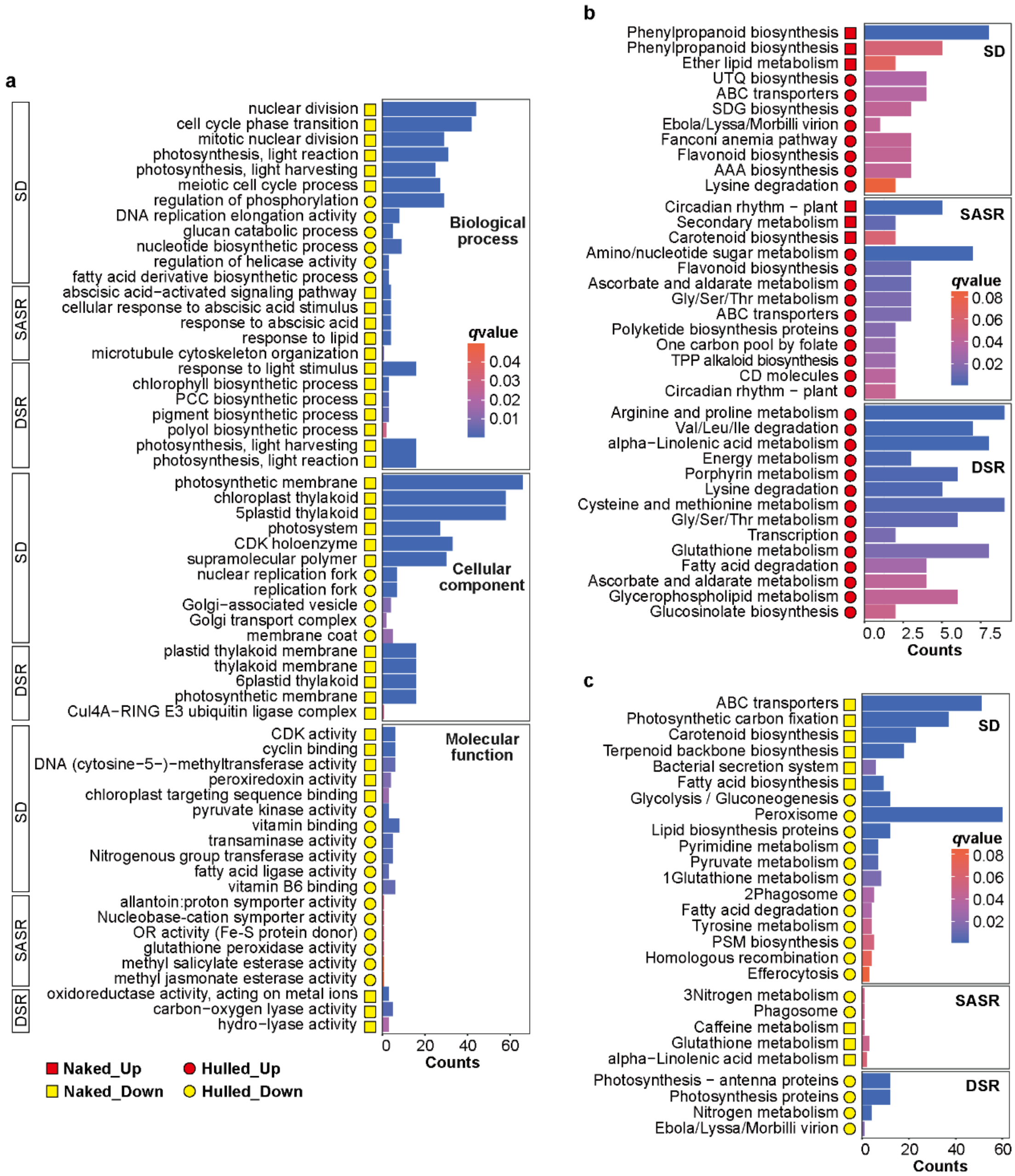

Supplementary Figure 18. Enrichment analysis of specific differentially expressed genes between hulled and naked oats based on transcriptome data of seed development and abiotic stress. (*q*-value < 0.05). (a) GO enrichment for genes specifically down-regulated in hulled or naked oats from condition-specific transcriptomes; (b) KEGG enrichment for genes specifically up-regulated during seed development in hulled or naked oats; (c) KEGG enrichment for genes specifically down-regulated in hulled or naked oats under salt-alkali or drought stress.

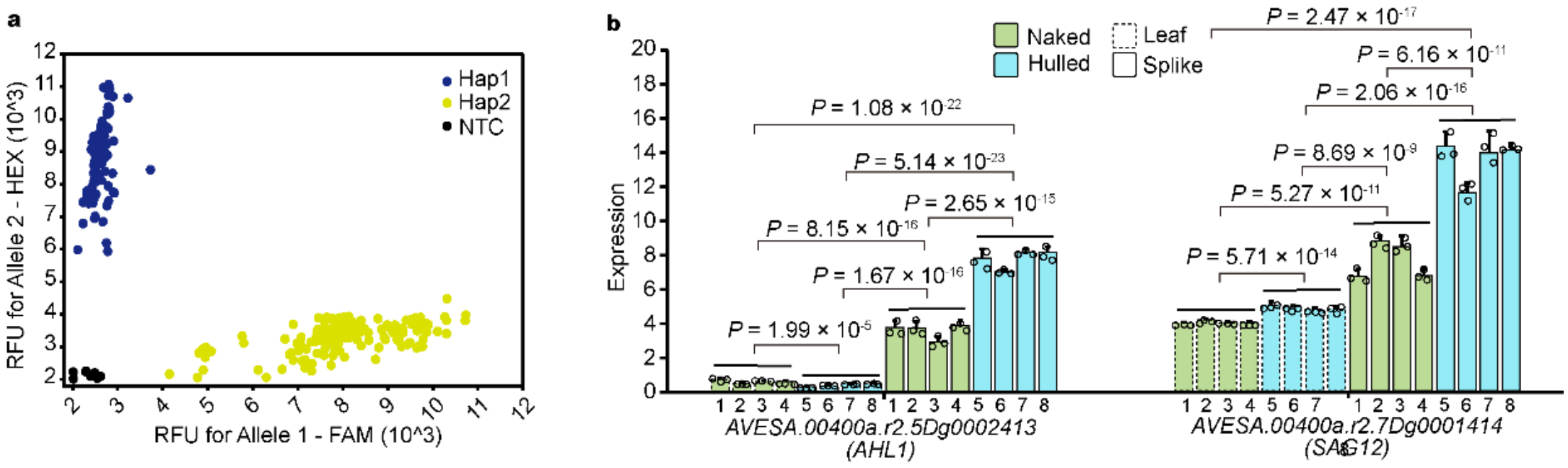

Supplementary Figure 19. Genetic analysis and validation of oat improvement-related genes identified in this study. (a) Genotyping results of a KASP marker, developed based on a SNP tightly linked to *AsUMAMIT25*, across 268 hexaploid cultivated oat accessions; (b) Expression patterns of *AsAHL1* and *AsSAG12* in leaves and panicles of hulled and naked oats during grain filling (samples 1–8 for qRT-PCR are detailed in Tab. S1.4).

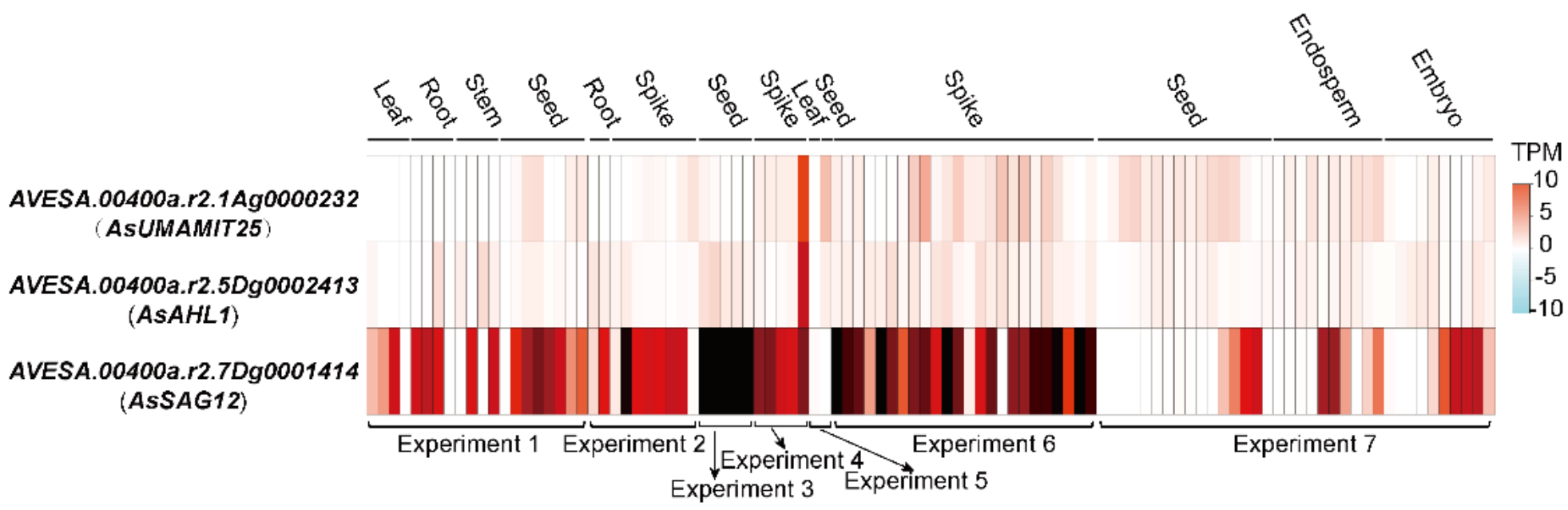

Supplementary Figure 20. Expression patterns of three selected QTL genes associated with yield and quality in naked oats, analyzed across publicly available panicle and seed transcriptome datasets.

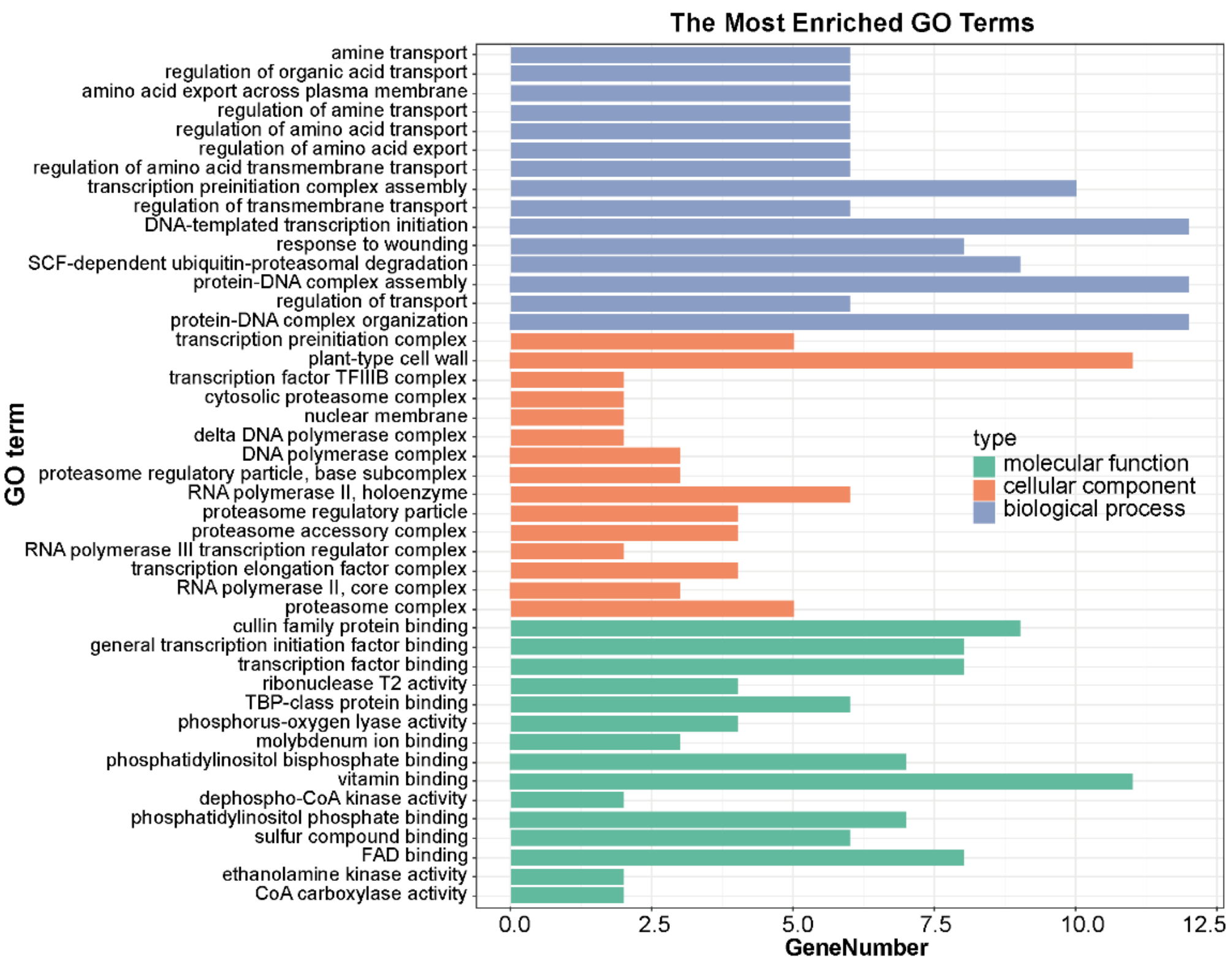

Supplementary Figure 21. GO enrichment results of selected genes in GIV (the core subgroup of landrace naked oats), showing the top 15 significantly enriched terms in each category of Molecular Function, Cellular Component and Biological Process (*P* < 0.05)

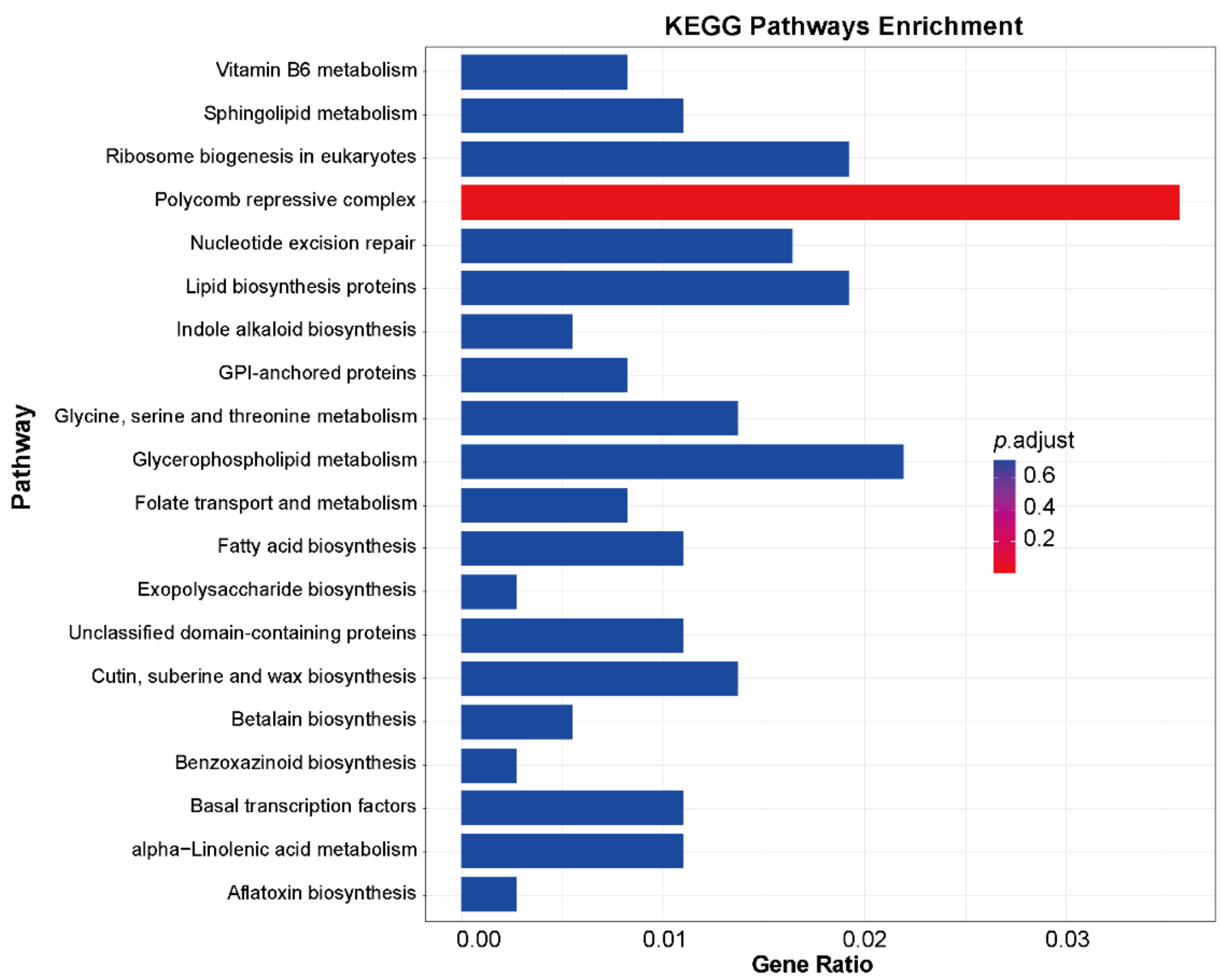

Supplementary Figure 22. KEGG enrichment results of genes under selection in GIV (the core subgroup of landrace naked oats), showing the top 20 enriched terms.

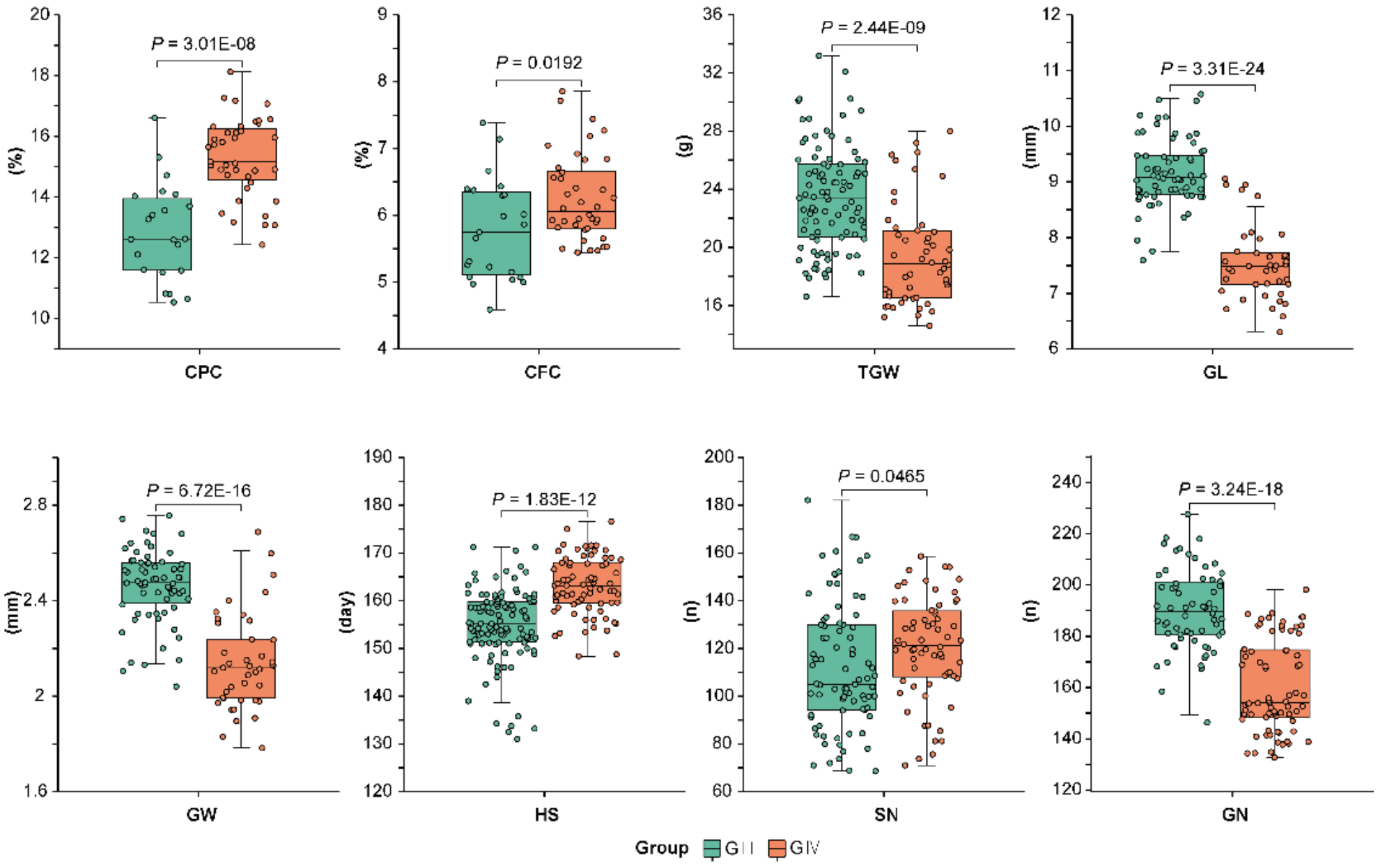

Supplementary Figure 23. Boxplots comparing phenotypic trait differences between subgroup GIV (core landrace naked oat subgroup) and subgroup GIII (hulled oats from the Central and Eastern Eurasia)
