# Supplementary material for: Investigation of naked oat (Avena sativa ssp. nuda) domestication provides insights into yield and nutritional gaps in oats
Source: Commun Biol. 2026 Jul 11;9:1453. doi: 10.1038/s42003-026-10626-w (PMC13737007; doi:10.1038/s42003-026-10626-w)
Supplement: Supplementary file 2 [file 42003_2026_10626_MOESM2_ESM.pdf]

Descriptions of Supplementary Files

1. Supplementary Figures.pdf

This file contains all supplementary figures of the manuscript:

Supplementary Figure 1: Genome-wide distribution density map of 127,563 SNPs, supporting all genotyping and population genetic analyses of this study.

Supplementary Figure 2: Admixture cross-validation (CV) error plot for 666 oats (K = 2–20)

Supplementary Figure 3: Genetic diversity analysis of the 666 tested oat accessions.

Supplementary Figure 4: Analysis of shared homozygous haplotypes between hulled and naked oats (10 kb–3000 kb).

Supplementary Figure 5: Analysis of gene flow and shared homozygous haplotypes among different clustered subgroups and accessions from diverse geographical origins (10 kb–3000 kb).

Supplementary Figure 6: *D*-test of gene flow events among hulled and naked oat accessions from different subpopulations and geographic regions based on the ABBA-BABA model.

Supplementary Figure 7: Validation of known Chr7D inversion haplotypes. High-resolution haplotype analysis combined with published data from Bekele et al. resolves naked oat domestication trajectory.

Supplementary Figure 8: Admixture cross-validation (CV) error plot for 366 landraces and 7 wild accessions of hexaploid oat (K = 2–15).

Supplementary Figure 9: Population structure and genetic diversity analyses of 366 landraces and 7 wild accessions of hexaploid oat.

Supplementary Figure 10: Correlation heatmaps of 17 agronomic traits and 2 grain quality-corresponding traits in oat.

Supplementary Figure 11: Score plot of the first three principal components from PCA based on 19 phenotypic traits in 666 oat accessions.

Supplementary Figure 12: Comparative analysis of core phenotypic traits across different genetic subpopulations of 666 oat accessions.

Supplementary Figure 13: Genetic diversity and differentiation across all

chromosomes between 504 hulled and 162 naked oat accessions.

Supplementary Figure 14: Scatter line plot of nucleotide diversity (π) and Tajima's *D* neutrality test values in genomic regions harboring 1504 selected genes between hulled and naked oats.

Supplementary Figure 15: Functional enrichment analysis of genes under directional selection during domestication and improvement in naked oats.

Supplementary Figure 16: Functional enrichment analysis of genes under balancing selection during domestication and improvement in naked oats.

Supplementary Figure 17: Enrichment analysis of shared DEGs across transcriptome datasets and condition-specific upregulated genes in hulled and naked oats.

Supplementary Figure 18: Enrichment analysis of specific differentially expressed genes between hulled and naked oats based on transcriptome data of seed development and abiotic stress.

Supplementary Figure 19: Genetic analysis and validation of oat improvement-associated genes identified in this study, including genotyping validation of the *AsUMAMIT25*-linked KASP marker and expression profiling of *AsAHL1* and *AsSAG12* in leaves and panicles of hulled and naked oats during grain filling.

Supplementary Figure 20: Expression patterns of three selected yield- and quality-associated QTL genes in naked oats, analyzed using publicly available panicle and seed transcriptome datasets from NCBI.

Supplementary Figure 21: GO enrichment results of selected genes in GIV (the core subgroup of landrace naked oats), showing the top 15 significantly enriched terms in each category of Molecular Function, Cellular Component and Biological Process.

Supplementary Figure 22: KEGG enrichment results of genes under selection in GIV (the core subgroup of landrace naked oats), showing the top 20 enriched terms.

Supplementary Figure 23: Boxplots comparing phenotypic trait differences between subgroup GIV (core landrace naked oat subgroup) and subgroup GIII (hulled oats from the Central and Eastern Eurasia).

2. Supplementary_Data_1.xlsx

This file compiles all summarized supplementary tables corresponding to the core analyses in the main text. Each worksheet contains collated statistical results and annotation information matched to the main figures. Detailed contents are as follows:

Table S1: Germplasm classification, geographic origin, and population structure assignment (Admixture, NJ phylogenetic clustering, PCA) for the 666 oat accessions used in this study.

Table S2: Statistics of genetic diversity, population differentiation index, and Tajima's *D* neutrality test across different germplasm types and subpopulations of the 666 tested oat accessions.

Table S3: Gene flow analysis results across different germplasm types and subpopulations, generated from shared homozygous haplotype profiling, TreeMix analysis, and ABBA-BABA tests.

Table S4: Population structure, genetic diversity, and gene flow analysis results for 366 oat landraces and 7 wild oat accessions, along with demographic inference of naked oat domestication history.

Table S5: Phenotypic divergence statistics between hulled and naked oats, based on multi-environment phenotypic data of 666 oat accessions collected from 2010 to 2021.

Table S6: Identification and functional enrichment of putative selective sweep regions and selected genes in naked oats using F_{st}, π ratio (hulled/naked) and XP-CLR with hulled oats as the reference group.

Table S7: Analysis results of transcriptional regulatory divergence between hulled and naked oats, and screening of key candidate genes associated with yield, quality, and stress tolerance, based on 38 transcriptome datasets spanning seed development, saline-alkali stress, and drought stress.

Table S8: Haplotype analysis, KASP marker genotyping, qRT-PCR validation, and breeding potential evaluation of yield-related candidate genes, including representative genes *AsUMAMIT25*, *AsAHL1*, and *AsSAG12*.

3. Supplementary_Data_2.xlsx

This file contains raw data from Admixture and nucleotide diversity analyses in this study, as well as multi-environment phenotypic data for 666 tested oat accessions:

Table S9: Raw ancestry component Q-matrix data from Admixture analysis: 666 tested oat accessions at $K = 2–7$, and 366 oat landraces plus 7 wild oat accessions at $K = 2–6$.

Table S10: Raw nucleotide diversity data for oat germplasms under different grouping schemes: 666 tested accessions (6 clustered subpopulations and 10 geographic populations), and 366 landraces plus 7 wild oat accessions (5 subpopulations).

Table S11: Curated multi-environment phenotypic data for the 666 tested oat accessions.
