# Supplementary material for: Investigation of naked oat (Avena sativa ssp. nuda) domestication provides insights into yield and nutritional gaps in oats
Source: Commun Biol. 2026 Jul 11;9:1453. doi: 10.1038/s42003-026-10626-w (PMC13737007; doi:10.1038/s42003-026-10626-w)
Supplement: Supplementary file 3 [file 42003_2026_10626_MOESM5_ESM.pdf]

nature portfolio

Corresponding author(s): Yuanying Peng, Changzhong Ren

Last updated by author(s): Jun 17, 2026

Reporting Summary

Nature Portfolio wishes to improve the reproducibility of the work that we publish. This form provides structure for consistency and transparency in reporting. For further information on Nature Portfolio policies, see our Editorial Policies and the Editorial Policy Checklist.

Statistics

For all statistical analyses, confirm that the following items are present in the figure legend, table legend, main text, or Methods section.

n/a	Confirmed	
☐	☒	The exact sample size (*n*) for each experimental group/condition, given as a discrete number and unit of measurement
☐	☒	A statement on whether measurements were taken from distinct samples or whether the same sample was measured repeatedly
☐	☒	The statistical test(s) used AND whether they are one- or two-sided<br>*Only common tests should be described solely by name; describe more complex techniques in the Methods section.*
☒	☐	A description of all covariates tested
☒	☐	A description of any assumptions or corrections, such as tests of normality and adjustment for multiple comparisons
☐	☒	A full description of the statistical parameters including central tendency (e.g. means) or other basic estimates (e.g. regression coefficient) AND variation (e.g. standard deviation) or associated estimates of uncertainty (e.g. confidence intervals)
☐	☒	For null hypothesis testing, the test statistic (e.g. *F*, *t*, *r*) with confidence intervals, effect sizes, degrees of freedom and *P* value noted<br>*Give P values as exact values whenever suitable.*
☒	☐	For Bayesian analysis, information on the choice of priors and Markov chain Monte Carlo settings
☒	☐	For hierarchical and complex designs, identification of the appropriate level for tests and full reporting of outcomes
☐	☒	Estimates of effect sizes (e.g. Cohen's *d*, Pearson's *r*), indicating how they were calculated

Our web collection on statistics for biologists contains articles on many of the points above.

Software and code

Policy information about availability of computer code

Data collection	Wanshen SC-G automatic seed analyzer, Dumas rapid nitrogen analyzer (Rapid N Exceed, Elementar, Germany), Soxhlet extraction method (GB/T 6433-2006), Nanophotometer (Thermo Fisher), Bio-Rad Image lab Touch software (version 2.1.0.35), Illumina HiSeq 2000, JLM qPCR AnalyzerSoft(QX400), Bio-Rad Real-Time System(CFX96).
Data analysis	Excel (version 2108), IBM SPSS Statistics 27.0, GCTA (v1.0), BWA-MEM (v0.7.8), GATK (v4.4.0), SAMtools (v1.16.1), ADMIXTURE (v1.3.0), MEGA X, VCFtools (v0.1.16), Salmon (v0.8.2),Kallisto2Matrix (v1.0.0), pixy (v1.2.7), Dsuite (v0.4), fastsimcoal2 (v2.7.0.9), edgeR (v1.6.15), FASTQ (v0.23.4), LDBlockShow, Treemix (v1.13), Primer3 Input (v0.4.0), OatBioDB (http://www.waooat.cn). The data analysis was performed following standard workflows of the respective software with optimized parameters. Detailed parameters are provided in the Materials and Methods section.

For manuscripts utilizing custom algorithms or software that are central to the research but not yet described in published literature, software must be made available to editors and reviewers. We strongly encourage code deposition in a community repository (e.g. GitHub). See the Nature Portfolio guidelines for submitting code & software for further information.

Data

Policy information about availability of data

All manuscripts must include a data availability statement. This statement should provide the following information, where applicable:

- Accession codes, unique identifiers, or web links for publicly available datasets
- A description of any restrictions on data availability
- For clinical datasets or third party data, please ensure that the statement adheres to our policy

The data that support the findings of this study are provided in the Article and its Supplementary Information. The genotyping-by-sequencing (GBS) data are available under NCBI BioProject accession PRJNA807126. The RNA-seq data are publicly available under accession PRJNA794002.

Research involving human participants, their data, or biological material

Policy information about studies with human participants or human data. See also policy information about sex, gender (identity/presentation), and sexual orientation and race, ethnicity and racism.

Reporting on sex and gender	No ethical approval was required for this study, as the materials used are oat crop plants, not humans or experimental animals. Written permission was obtained from all institutions that provided the germplasm resources. The collection and exchange of these materials complied with the Seed Law of the People's Republic of China and international conventions.
Reporting on race, ethnicity, or other socially relevant groupings	The concepts of race and ethnicity are not variables or subjects of analysis in this work.
Population characteristics	Our study does not involve population characteristics.
Recruitment	Our study does not involve the recruitment of human samples.
Ethics oversight	Our study does not require ethical oversight.

Note that full information on the approval of the study protocol must also be provided in the manuscript.

Field-specific reporting

Please select the one below that is the best fit for your research. If you are not sure, read the appropriate sections before making your selection.

☒ Life sciences ☐ Behavioural & social sciences ☐ Ecological, evolutionary & environmental sciences

For a reference copy of the document with all sections, see nature.com/documents/nr-reporting-summary-flat.pdf

Life sciences study design

All studies must disclose on these points even when the disclosure is negative.

Sample size	666
Data exclusions	We did not exclude the data.
Replication	We conducted a 12-year, multi-environment trial (2010-2021) to obtain robust phenotypic data. We called SNPs using a standard protocol and identified loci by intersecting results from multiple genome-wide scan methods. Finally, we validated these loci through molecular experiments (KASP, qRT-PCR), performing all assays with over three replicates.
Randomization	The analysis of all samples was conducted using pre-defined objective criteria to avoid researcher bias.
Blinding	Blinding was not necessary as no intervention was included in the study

Reporting for specific materials, systems and methods

We require information from authors about some types of materials, experimental systems and methods used in many studies. Here, indicate whether each material, system or method listed is relevant to your study. If you are not sure if a list item applies to your research, read the appropriate section before selecting a response.

Materials & experimental systems

n/a	Involved in the study	
☒	☐	Antibodies
☒	☐	Eukaryotic cell lines
☒	☐	Palaeontology and archaeology
☒	☐	Animals and other organisms
☒	☐	Clinical data
☒	☐	Dual use research of concern
☐	☒	Plants

Methods

n/a	Involved in the study	
☒	☐	ChIP-seq
☒	☐	Flow cytometry
☒	☐	MRI-based neuroimaging

Dual use research of concern

Policy information about dual use research of concern

Hazards

Could the accidental, deliberate or reckless misuse of agents or technologies generated in the work, or the application of information presented in the manuscript, pose a threat to:

No	Yes	
☒	☐	Public health
☒	☐	National security
☒	☐	Crops and/or livestock
☒	☐	Ecosystems
☒	☐	Any other significant area

Experiments of concern

Does the work involve any of these experiments of concern:

No	Yes	
☒	☐	Demonstrate how to render a vaccine ineffective
☒	☐	Confer resistance to therapeutically useful antibiotics or antiviral agents
☒	☐	Enhance the virulence of a pathogen or render a nonpathogen virulent
☒	☐	Increase transmissibility of a pathogen
☒	☐	Alter the host range of a pathogen
☒	☐	Enable evasion of diagnostic/detection modalities
☒	☐	Enable the weaponization of a biological agent or toxin
☒	☐	Any other potentially harmful combination of experiments and agents

Plants

Seed stocks	This study utilized a collection of Chinese naked oats, North American breeding lines, and diverse genotypes from the Canadian Plant Gene Resources Consortium (PGRC), with all corresponding catalog numbers provided in the supplementary materials.
Novel plant genotypes	This study does not involve novel plant genotypes.
Authentication	This study does not involve the authentication of novel plant genotypes.
