# Supplementary material for: Investigation of naked oat (Avena sativa ssp. nuda) domestication provides insights into yield and nutritional gaps in oats
Source: Commun Biol. 2026 Jul 11;9:1453. doi: 10.1038/s42003-026-10626-w (PMC13737007; doi:10.1038/s42003-026-10626-w)
Supplement: Supplementary file 4 [file 42003_2026_10626_MOESM6_ESM.pdf]

nature portfolio

Peer Review File

Domestication of naked oat (Avena sativa ssp. nuda) provides insights bridging yield and nutritional gaps in oats

Corresponding Author: Dr Yuanying Peng

This file contains all reviewer reports in order by version, followed by all author rebuttals in order by version.

Attachments originally included by the reviewers as part of their assessment can be found at the end of this file.

Version 0:

Reviewer comments:

Reviewer #1

(Remarks to the Author)
Review

In the manuscript "Bridging the Yield and Nutritional Gap in Oat: Insights from Naked Oat (Avena sativa ssp. nuda) Domestication", the authors Dong et al. evaluate the population structure, genetic diversity, and gene flow of 666 oat accessions from 65 countries using 127,563 SNPs obtained through GBS.
The main objective of this work was to assess the genetic origin and phenotypic divergence between hulled oat (Avena sativa) and naked oat (Avena sativa var. nuda). The panel consists of 293 bred varieties (227 hulled oat [H] and 66 naked oat [N]), 366 landraces (270 H and 96 N), and 7 wild materials (H).
The study used 19 agronomic and quality traits evaluated over 11 years and found that hulled oats generally have higher yield, while naked oats exhibit higher nutritional quality (e.g., crude protein and fat content). Genome-wide scans and RNA-seq analyses identified numerous genes and markers involved in metabolite synthesis and abiotic stress resistance in naked oats.

Major Comments

The authors fail to clearly explain, for readers not familiar with oat, the difference between hulled oat (A. sativa) and naked oat (A. sativa var. nuda). A comparison table summarizing the populations used throughout the manuscript is necessary. It is unclear whether the main goal was to compare hulled versus naked oats, or to evaluate differences among countries or improvement levels (wild materials, landraces, and cultivars).

Throughout the manuscript, numerical values for Fst and genetic diversity are missing. The authors instead use vague terms such as “weak genetic differentiation” or “low genetic diversity.” These quantitative results should be explicitly reported in tables.

Overall, the figures are difficult to interpret and appear unnecessarily complex. For instance, the Pearson correlation in Fig. S6a is unclear, and the Circos plots—especially those for Fst (Fig. 1f, Fig. S4b, and Fig. S4e)—are hard to follow. Fst values and other intra/inter-genetic metrics should be summarized in a table.

I have not access any supplementary tables, and many of the results described in the manuscript are missing. All supplementary materials should be deposited in Figshare or a similar public repository.

1. Population Structure

In the PCA (Fig. 1d), there is a clear separation of a cluster along PC1, but the authors do not provide any information about this group of accessions.

L116: What statistical test supports the claim of breeding introgression in GVI, which includes 13 Chinese naked oats and 61 hulled cultivars from Oceania and North America?

2. Domestication Pathway

Why did the authors not use the classical definition of gene flow, $N_m = (1 - Fst)/(4 \times Fst)$?

L135: The authors mention “subgroup divergence chronology shows Mediterranean hulled groups (G2/G3),” but do not provide collection or development dates for all accessions. What evidence supports this statement?

Of the 19 evaluated traits, 17 are agronomic and only two are quality traits (crude protein and crude fat content). Therefore, it is difficult to draw strong conclusions regarding quality.

L693: I suggest replacing Fig. S6b with a table showing the eigenvectors (Fig. 3d), since the current table is difficult to interpret and fails to capture genetic variance effectively.

3. Phenotypic Analysis

There is no multi-environment genetic modeling or other analyses that elucidate how divergent selection drives yield–quality trade-offs or environmental adaptation between hulled and naked oats.

The authors do not include analyses of broad-sense heritability or genetic correlations among traits.

L688: Agronomic traits should be grouped and discussed by yield and quality.

L222: “The results suggest that naked oats and hulled oats have developed distinct stress response traits through directional selection in their respective cultivation environments.” However, no stress conditions were tested, and the functional enrichment analysis is insufficient to support this conclusion.

L180: Why did the authors not compare improvement levels or countries directly, rather than relying solely on clustering? Groups GI–GX were generated from a Neighbor-Joining (NJ) analysis based on 127,563 markers, including both neutral and non-neutral variants, and each cluster contains mixed accession types.
What is the LD decay for the full dataset and for each population (naked vs. hulled)?
How was the threshold for Fst and Pi ratio chosen in Figure 4a?

4. Genome-Wide Scanning and RNA-seq

L187: Why did the authors perform a genome-wide selective sweep analysis rather than a genome-wide association study (GWAS)? Selection scans are generally more informative in advanced populations after several selection cycles where Fst changes within specific genomic regions.

L227: The RNA-seq analysis of 22 genetically and geographically diverse accessions is interesting, but why were tissues (root, stem, leaf, spike) pooled across different developmental stages? Using pooled tissues increases background noise and makes it difficult to identify differentially expressed genes (DEGs). The 22 accessions could have been sequenced separately with replicates instead.
If the goal was to identify DEGs in spikes and relate them to oat type, only spike tissue should have been used.

L247: It is unclear how the transcriptomic findings align with the genome-wide selection scans. How many associated markers were overlaping with DEG? and how to correlate the role of the 19 traits with the Genome-Wide Scanning and RNA-seq analysis?

L270: The rationale for using both KASP markers and qPCR is unclear. Typically, qPCR is used to validate DEGs.

Minor Comments

L100: Replace “Ancestral component analysis” with “ADMIXTURE analysis.”

Fig. 1 L664: “Ancestral component” refers to a structure analysis with multiple K. Include sample origin in the PCA (e.g., AFR for Africa). Please maintain consistent geographic abbreviations across figures—e.g., Fig. S5 uses “CHN” for China and “AF” for Africa, but other figures use different labels.
Panels (c) and (d) could be replotted using an interactive 3D Plotly visualization. Panel (f) (Circos plot) could be replaced with a table or matrix for easier comparison.

L686: “Spike type (ST)” and “Spikelet type (SLT)” are mislabeled in Fig. 3b. The figure also lacks axis labels.

L161: The morphological differences between hulled and naked oats (e.g., multiflowered spikelets and long awns) are not clearly visible in Fig. 3b.

Reviewer #2

(Remarks to the Author)
In this manuscript, Dong and colleagues systematically integrated population genetics, multi-environment phenotypes, selection and clearance scanning, transcriptomics and functional verification, and comprehensively analyzed the domestication pathway, yield-nutrient trade-off mechanism and key candidate genes of naked oat. The scale of the germplasm (666 accessions) and phenotyping (19 traits) is impressive. Despite the claim to have analyzed 666 "globally sourced" oat accessions, 90% of the naked oat samples (146 out of 162) originate from China, while critical regions implicated in the origin debate, such as Central Asia, Eastern Europe, Turkey, and Russia, are severely underrepresented. This 'pseudo-global' sampling strategy effectively only validates the genetic background of Chinese naked oats, and fails to rule out the possibility of independent domestication events occurring elsewhere. The paper overstates its causal claims, lacks mechanistic validation, and fails to address alternative evolutionary scenarios. I recommend major revision with a sharper focus on correlation rather than causation, and a much more cautious interpretation of domestication history. These issues need appropriate revisions and clarifications before this work can be endorsed for publication.

Major Comment:
1. Lines 77-80: The Introduction acknowledge that oats are allohexaploid, and that structural variations (SVs), such as the chr7D inversion (Bekele et al. 2024) or chr1A-chr1C translocations (Li et al. 2025), have been shown to drive domestication-related traits, and independent domestication hypothesis based on chr7D inversion, while this study derived Mediterranean hulled oat origins using solely SNP data. The authors should explicitly emphasize the differences and complementary nature between these two methodological approaches.

2. Lines 103-104: The '$F_{st} = 0.0701$' indicates 'weak differentiation', but fails to justify this threshold in the context of an allohexaploid crop. In polyploids, Fst values are often lower than in diploids — 0.07 may represent moderate or even strong differentiation. The authors must compare this value with published Fst distributions in oat or other polyploids (e.g., Li et al. 2025) and explicitly define why 0.07 is classified as 'weak'.

3.Line 123-126: The authors claim that "The 666 accessions displayed a reticulate structure, maintained by 18 inter-regional and 22 inter-subgroup gene flow events" based primarily on TreeMix analysis. While TreeMix can suggest historical gene flow, relying solely on this method provides limited evidence for a complex reticulate population structure. The manuscript does not provide independent validation of these events (e.g., f-branch statistics, D-statistics, or haplotype-based tests), and the connection between the numerical counts (18 inter-regional, 22 inter-subgroup) and the visualization in Fig. S4g, h is unclear.

4. In the "Domestication pathway of naked oats" section, the authors conclude that naked oats originated from a key divergence during the eastward dispersal of Mediterranean hulled oat landraces and delineate the domestication trajectory of naked oats. However, the current evidence-gene flow analyses, Fst differentiation, and π statistics-is largely correlative. These analyses establish genetic affinities but do not directly demonstrate the direction, geographic location, or uniqueness of the proposed domestication route. A more quantitative model-based comparison (e.g., through fastsimcoal2, or qpgraph topology testing) is required to distinguish among alternative origins, clarify the assumptions of the differentiation time model and to evaluate whether the Mediterranean-origin scenario is statistically favored over other plausible demographic models. The Discussion should more clearly distinguish supportive-but not decisive-evidence and avoid overinterpretation. Statements about the domestication center should be moderated unless supported by explicit demographic model testing.

5. Line 159-186: "The phenotypic divergence analysis" section, describes differences between naked and hulled oats in yield- and quality-related traits across multiple environments. However, 1) the causal links between these observed phenotypic differences and underlying genetic variation are not fully established. While PC analysis and multi-environment data reveal patterns of variation, these correlations do not clarify which specific loci or selection events drive the divergence. No genome-wide association (GWAS) or QTL analyses are integrated with the phenotypic data to directly link candidate genes or selection sweeps with observed trait differences. Such analyses would strengthen the inference of genetic determinants underlying the phenotypic divergence. 2) Environmental effects across years and locations are not explicitly accounted for in the analyses. Without proper correction for environmental and management variation, the observed phenotypic differences may be confounded, potentially affecting conclusions about trait divergence between oat types.

6. Line 195-197: The authors define the top 1% of windows based on Fst, π ratio (hulled/naked), and XP-CLR as candidate selected regions, identifying 1,504 putative selected genes. However, the rationale for choosing the top 1% threshold is not statistically justified, and no cross-validation is presented. Given the complex population structure and evidence of extensive gene flow, relying on a single XP-CLR or Fst metric may generate false positives. I recommend implementing multiple complementary methods or using simulation/resampling approaches to assess the robustness of candidate gene identification.

7. Line 197-199: Tajima's $D < 0$ as evidence of positive selection and $D \geq 0$ as negative or balancing selection. This interpretation is overly simplistic because Tajima's D is also sensitive to demographic history, gene flow, and genetic drift. The authors should explicitly discuss these confounding factors or consider more rigorous statistical approaches to distinguish true selection from demographic effects.

8. Line 200-224: The authors link enriched pathways and specific haplotypes (e.g., GPAT9, SAE2) to nutritional and yield traits. However, functional enrichment and frequency-based associations only indicate correlation, not causation. The manuscript should avoid overstating direct phenotypic effects and clarify that these are putative associations unless supported by GWAS or functional validation.

9. Line 226-229: "from 13 naked oat and 9 hulled oat accessions"-the sample size is relatively limited given the global diversity and complex population structure of Avena. The selected accessions may not adequately represent the full genetic breadth of hulled and naked oats, which restricts the generalizability of the inferred transcriptional differences.

10. Lines 250-257: The section claims that "naked oats prioritize expression of nutrient metabolism genes," yet the RNA-seq data were generated from pooled tissues (root, stem, leaf, and spike) — making it impossible to determine whether the observed expression differences are driven by grain-specific processes or by other tissues (e.g., root or stem). Moreover, among the 39 differentially expressed and selected genes highlighted in Fig. 5c, many are annotated as "cytoskeletal" or "transporter" genes - which are not directly involved in nutrient biosynthesis. This weakens the causal link between gene expression and grain nutritional traits. The authors must either supplement the analysis with tissue-specific RNA-seq (e.g., developing spikelets or seeds) or explicitly acknowledge that the expression patterns observed may not reflect grain-specific regulation. Without this, the inference that "naked oats prioritize nutrient synthesis at the transcriptional level" remains speculative.

11. Lines 325-337: The Results section completely overlooks the large-scale inversion on chromosome 7D (H4 haplotype), recently identified by Bekele et al. (2024) as a key structural variant (SV) strongly associated with naked grain development and domestication in oat. While the authors rely exclusively on SNP-based analyses, they fail to integrate or even mention this well-established SV, which has been shown to co-segregate with the naked phenotype and exhibit strong geographic and phenotypic correlations. This omission represents a significant gap in the genomic interpretation of domestication. The authors must supplement their analysis by examining the distribution of the chr7D inversion across their 10 genetic subgroups (e.g., GI-GX), and/or perform co-localization analysis to test whether their SNP-based selection signals (e.g., XP-CLR peaks) overlap with the inversion breakpoints or haplotype blocks. Without this, the study's conclusion on the genomic basis of naked oat domestication remains incomplete.

Minor Comments:
1. The population structure lacks explicit labeling of oat types (Fig. 1a). Consequently, the genetic composition of hulled versus naked oats within the inferred clusters is unclear, it is difficult to interpret whether the observed clustering truly reflects domestication or population structure patterns. Incorporating sample type annotations in the figures would clarify the correspondence between genetic clusters and phenotypic classifications.

2. In Fig. 1b, it is unclear why the wild oat groups were not used as an outgroup, but were instead included within the ingroup together with the cultivated oat lineages.

3. Lines 144-148: Gene flow analysis assumes directional migration but lacks statistical support for migration rates (e.g., Migrate-N or BayesAss). Provide confidence intervals for inferred events. The authors must add statistical support for each migration event (e.g., bootstrap support > 80% or LRT $P < 0.05$).

Reviewer #3

(Remarks to the Author)
In the present study, Dong et al. analyzed 666 hulled and naked oat accessions originating from different countries and representing wild samples, landraces, and bred varieties. Their goal was to address the genetic origin and phenotypic trait divergence between hulled and naked oats by integrating genotypic, phenotypic, and transcriptomic data. They propose that naked oats originated from a key divergence event during the eastward dispersal of Mediterranean hulled oat landraces, followed by independent domestication and diversification in China. Using multi-environment phenotypic data, they showed phenotypic differences between the two types, with hulled oats exhibiting higher yield and larger grain size, whereas naked oats had higher crude protein and fat content. They also incorporated a selection scan during naked oat domestication, confirming a trade-off between genes linked to quality and yield. By combining selection signatures with differential gene expression, they identified three genes associated with previously reported oat grain-yield QTLs.

Major comments:
While Dong et al. present a large and valuable dataset integrating genomic, phenotypic, and transcriptomic information from 666 hulled and naked oat accessions, several aspects of the work suffer from unclear presentation, overly dense descriptions, and insufficient explanation of key analysis.
A major issue is the lack of a clear, logical sequence in the population genetics and domestication sections. The authors report numerous subgroups, divergence events, and geographic transitions, but do not clearly explain how these analyses support their proposed evolutionary scenario. Although they argue that naked oats originated from hulled landraces during an eastward Mediterranean dispersal, the genomic evidence underlying this conclusion is only briefly referenced. As a result, the domestication narrative remains difficult to follow, and the claim of independent domestication in China is not convincingly supported.
The presentation of figures further complicates interpretation. Several figures and corresponding legends lack critical information, making it difficult to understand how the data support the authors' claims.
The selection-scan analyses are similarly problematic. The rationale for the specific methods, thresholds, or integration strategy is not explained. Without justification, it is unclear why these statistics were chosen or how the candidate regions were identified, weakening confidence in the reported selection signals.
Transcriptomic analyses also lack critical clarity. The manuscript does not clearly define which comparisons were performed (e.g., hulled vs. naked, tissues, or subgroups). Instead, the results list numbers of DEGs and enriched pathways without connecting them to the experimental design or explaining their biological relevance. This makes it difficult to interpret how expression differences relate to domestication or adaptive divergence.

Overall, the manuscript presents a rich dataset with potential insights into oat domestication, but the narrative is difficult to follow due to unclear explanations, insufficient justification of methods, and a focus on listing results rather than discussing them. I believe a more structured presentation with clear rationale for analytical choices and better integration of results would be essential for its publication.

Line-by-line comments:
L82 I would write the acronyms the first time you mention it.
L163-164 Fig. 3b is not properly explained and misses axis labels.
L170-172 Fig. 3c missies axis labels and it is not clear how the scale on the Y axis could refer to different units, e.g GCI, mm CFC & CPC, %, etc.
L178 I would avoid including the table as a figure, also it does not help supporting the claims in the text.
L189-190 I think it would be more appropriate talking about nucleotide diversity, not genetic divergence.
L193 Here it would be appropriate to calculate the loss of diversity statistic (Vigouroux et al. 2002).
L195-196 This approach to detect genes under selection should be much better explained.
L198 This interpretation of Tajima's D is oversimplified and not strictly correct. Tajima's D detects whether a population's allele frequency spectrum deviates from the neutral model, but it doesn't specify which evolutionary force caused the deviation.
L204 The p-values or p.adjust showed in the Fig. S9 and supplementary tables are not significant. Have any normalizations been applied that were not reported?
L207-on This integration of homology annotation and allele frequency shifts should be much better explained.
L229 You mention different accessions and tissues, but it is not clear what was used for the differential expression analysis.
L237. Fig 5a,b misses axis labels. In the Table S8 some FDR values are high and not significant, was that considered?
L252 The FPKM values you show in Fig. 5c cannot be negative. Have any normalizations been applied that were not reported? Also, you show expression values per accession, but you have RNA-seq from different tissues, how can this be possible?
L302 The hulled and naked genotypes are not indicated in the admixture plot. If they correspond to the blue and yellow at K2, I would say the opposite, since the yellow group is strongly conserved across all K values.
L305 There are no Fig. 4g,h.
L430 After the HaplotypeCaller, haven't you performed the joint genotyping? If not, how did you generate the single vcf file containing all the samples?
L453 Specify which software you used for the alignment.
L481 It is not clear how you computed the XP-CLR scores.

Version 1:

Reviewer comments:

Reviewer #1

(Remarks to the Author)
The authors have addressed all comments/questions, and the revised manuscript shows significant improvements in its flow, level of detail, and figures. I have no additional comments.

Reviewer #2

(Remarks to the Author)
This manuscript has been significantly improved compared to the previous version, in which the major concerns I raised earlier have been adequately addressed. However, I still have a few comments as outlined below:

1. In Fig. 1b, the authors arrange the ADMIXTURE results according to the topology of the phylogenetic tree. However, this approach is not particularly informative and raises methodological concerns. Phylogenetic trees derived from re-sequencing data are fundamentally constructed based on pairwise genetic distances and therefore reflect clustering of genetic relatedness rather than true evolutionary history. In addition, the ordering of samples along such trees is highly sensitive to SNP filtering parameters, including thresholds for missing data and minor allele frequency (MAF). Even relatively small adjustments to these parameters can result in noticeable changes in sample arrangement. As a consequence, the current visualization may lead to a situation where samples with similar genetic ancestry components are not grouped together, reducing the clarity of population structure patterns and credibility.
For these reasons, we recommend presenting the phylogeny as an unrooted tree. Meanwhile, for ADMIXTURE visualization, samples should be ordered according to their ancestry proportions, which would provide a clearer and more interpretable representation of the underlying population structure.

2. Fst = 0.0701 is described as indicating "weak differentiation" based on the study by Nan et al. (Gigascience, 2022; https://doi.org/10.1093/gigascience/giad061) on hexaploid oat populations. However, upon careful examination of this article, we did not find an explicit classification standard defining thresholds for weak, moderate, or significant genetic differentiation (e.g., Fst < 0.08, 0.08-0.15, > 0.15). Therefore, the use of this specific categorization appears to be unsupported by the cited reference and may require either clarification or citation of an appropriate source that explicitly defines these thresholds.

3. Lines 196-199: The authors state that subpopulation GIV diverged approximately 6,124 generations ago and underwent an extreme population bottleneck (Ne ≈ 187). While this provides a clear demographic timeframe, such a severe bottleneck is typically accompanied by intense genetic drift. The authors need to clarify whether the observed genetic signal is a result of active anthropogenic selection during domestication, or simply a result of genetic drift associated with a founder effect during geographic isolation. The authors are encouraged to supplement their analysis or discuss within the text whether there are distinct selective sweep regions in the GIV group. If it can be demonstrated that Chinese naked oats possess unique selection signals divergent from GIII—particularly those associated with key traits such as the naked grain phenotype, crude protein and fat content—the argument for North China as a 'secondary domestication center' would be significantly more compelling and robust.

4. Lines 202-206: The authors state that "GII's Ne (969) was markedly lower than the model-predicted value (1,860) ... These patterns collectively support GII as the ancestral lineage..." Under typical evolutionary scenarios, ancestral populations should have a higher effective population size (Ne) and greater genetic diversity. However, the authors identify GII as the ancestral group despite its observed Ne being significantly lower than the model-predicted value, which appears logically inconsistent with the 'ancestral diversity center' hypothesis. I suggest the authors briefly explain why the ancestral population GII has a lower Ne. Is it due to recent population contraction in the Mediterranean region, or insufficient sampling representativeness?

5. Lines 209-211: The authors conclude by inferring that: "...followed by secondary domestication in North China (highest π in naked oats)". Relying solely on the observation that 'naked oats have the highest diversity in China' is insufficient to designate China as the center of domestication. It is recommended that the authors supplement the Discussion section by addressing whether any archaeological or phenological evidence supports the presence of oat utilization in North China around the estimated timeframe (~6,124 generations ago). Aligning the genetic timeline with historical and archaeological records would provide a more cohesive and closed-loop argument for the proposed evolutionary scenario.

6. Line 264: The statistical approach used to calculate the P-values is not clearly described. We recommend that the authors explicitly specify the method used for significance testing, including the underlying assumptions and parameters, to ensure the robustness and reproducibility of these results.

Reviewer #3

(Remarks to the Author)
I appreciate the authors' efforts to improve the manuscript and the additional analyses provided in this revised version. The deeper exploration of evolutionary history is valuable, and the second part of the manuscript dedicated to phenotypic characterization and RNA-seq analysis has notably improved. This section is now clearer and provides more convincing support for the biological interpretation of the results.
However, several important issues remain in the first part of the manuscript, particularly concerning the population genetic framework and the interpretation of domestication pathways. A clearer explanation of the defined admixture groups is needed, including a more explicit justification of the admixture membership thresholds used to assign individuals to clusters. In particular, the attribution of hulled and naked genotypes should be carefully revised. In some figures, these classifications appear potentially mislabeled, which is a critical issue because these categories are central to the authors' conclusions. In addition, the authors should more clearly highlight the position of the wild accessions within the admixture analysis, as this information is essential to support the proposed domestication scenario.
The analytical order and presentation of results in the first section are still somewhat confusing, with population genetic metrics presented in a way that does not always clearly support the narrative. Improving the logical flow of analyses and ensuring that figures are consistently and correctly referenced would substantially strengthen the manuscript. At present, some figures are not optimally used to support the authors' claims, making it difficult for the reader to follow the argument.
Finally, the abstract would benefit from careful language revision, as some typos and phrasing issues reduce clarity.
Overall, while the second part of the manuscript has clearly improved, the first section still requires clarification and better structure in order to fully support the proposed evolutionary interpretation. Addressing these points would significantly enhance the clarity and impact of the study.
Line-by-line comments:
L115 it is not clear why you refer to figure 1f in this first paragraph.
L121 The different acronyms of Fig.1 and other, referring to the results of this paragraph, are not explained in the legends nor in the main text.
L124 Why did you choose best K=6, in this case?
L128-157 This section is really hard to follow, a reference to the figures showing the results reported for the different groups is needed (e.g. Fst, nucleotide diversity and Tajima's D). Moreover, form Fig. 1b it seems that the "G" groups are not properly assigned on the basis of the membership to the admixture groups (as it seems from the colors in the plot), comprising both pure and admixed individuals.
L159 How have you chosen these 366 lines?
L162 It is not clear why K=5 is now used instead of K=6. Also, in Fig 2a the color of GV is wrong in the legend.
L165 From the plot, the GIV yellow group is composed by hulled oats, not naked.
L174 Fig 2 h-j are referred but not described.
L182 The Fst values do not match the figure.
L213-219 A table here would be useful to follow these results.
L221 What do you mean by "genetic correlation"?
L228 There is no Fig.3

L239-257 PCA plots would be useful to follow these results.

Version 2:

Reviewer comments:

Reviewer #2

(Remarks to the Author)
The work is novel and of broad interest to the community, particularly because it advances our understanding of naked oat domestication and provides valuable genomic resources and analytical frameworks that will likely facilitate future research. Overall, I believe this study represents a significant contribution to the field and will be of interest to researchers working in related areas. The findings are likely to stimulate further investigations and may influence future studies in oat genomics, evolution, and trait improvement. I appreciate the authors' careful revisions and responses. I fully support publication of this manuscript in its current form.

Reviewer #3

(Remarks to the Author)
The revised manuscript has clearly improved, and the authors have addressed the comments raised in my previous review. Overall, I am satisfied with the revisions, and I do not have any further comments, except for a careful check of the figure references and captions throughout the manuscript. In particular, Figure 1a-c seems to be mislabeled, as the captions of panels a-c do not match the corresponding plots.

Open Access This Peer Review File is licensed under a Creative Commons Attribution 4.0 International License, which permits use, sharing, adaptation, distribution and reproduction in any medium or format, as long as you give appropriate credit to the original author(s) and the source, provide a link to the Creative Commons license, and indicate if changes were made.
In cases where reviewers are anonymous, credit should be given to 'Anonymous Referee' and the source.
The images or other third party material in this Peer Review File are included in the article's Creative Commons license, unless indicated otherwise in a credit line to the material. If material is not included in the article's Creative Commons license and your intended use is not permitted by statutory regulation or exceeds the permitted use, you will need to obtain permission directly from the copyright holder.
To view a copy of this license, visit https://creativecommons.org/licenses/by/4.0/

We appreciate the reviewers very much for the constructive comments and highlighting the contributions of our manuscript. Each suggested revision and comment, brought forward by the reviewers was accurately incorporated and considered. We have now revised the manuscript according to these valuable suggestions. We provide our point-by-point responses below. We have made every effort to ensure that our revisions and responses address all of the remaining concerns. We believe these revisions will significantly improve the manuscript's accessibility and logical flow.

Reviewers' Comments:

Reviewer #1 (Remarks to the Author):

Review

In the manuscript "Bridging the Yield and Nutritional Gap in Oat: Insights from Naked Oat (Avena sativa ssp. nuda) Domestication", the authors Dong et al. evaluate the population structure, genetic diversity, and gene flow of 666 oat accessions from 65 countries using 127,563 SNPs obtained through GBS.

The main objective of this work was to assess the genetic origin and phenotypic divergence between hulled oat (Avena sativa) and naked oat (Avena sativa var. nuda). The panel consists of 293 bred varieties (227 hulled oat [H] and 66 naked oat [N]), 366 landraces (270 H and 96 N), and 7 wild materials (H).

The study used 19 agronomic and quality traits evaluated over 11 years and found that hulled oats generally have higher yield, while naked oats exhibit higher nutritional quality (e.g., crude protein and fat content). Genome-wide scans and RNA-seq analyses identified numerous genes and markers involved in metabolite synthesis and abiotic stress resistance in naked oats.

RESPONSE: We are deeply grateful for this excellent and comprehensive summary of our manuscript. Specific revisions and responses to each comment are provided in detail below.

Major Comments

The authors fail to clearly explain, for readers not familiar with oat, the difference between hulled oat (A. sativa) and naked oat (A. sativa var. nuda). A comparison table summarizing the populations used throughout the manuscript is necessary. It is unclear whether the main goal was to compare hulled versus naked oats, or to evaluate differences among countries or improvement levels (wild materials, landraces, and cultivars).

RESPONSE: Thank you for your careful reading and constructive feedback. We appreciate your suggestions to improve the clarity and focus of our manuscript. We will address all three points in the revised version.

1) Clarifying the difference between hulled and naked oats

We agree that this fundamental distinction should be explained more clearly for a general audience. In the revised manuscript, we have added a dedicated paragraph in the Introduction with a concise explanation in the revised manuscript [L65-L70], and we have included a simple schematic figure to visually illustrate the key morphological and practical differences [Fig. R1 below; also Revised Manuscript Fig. 3a] as follows:

"Oats diverge into globally dominant hulled oats and China-predominant naked oats. In hulled oats, the caryopsis is tightly enclosed by a rigid lemma that remains attached after threshing, providing physical protection but necessitating dehulling prior to utilization. By contrast, in naked oats, the lemma detaches freely from the mature grain, as in wheat, permitting direct utilization but rendering the grain more susceptible to mechanical damage (*New Phytologist* 1996 https://doi.org/10.1111/j.1469-8137.1996.tb01141.x)."

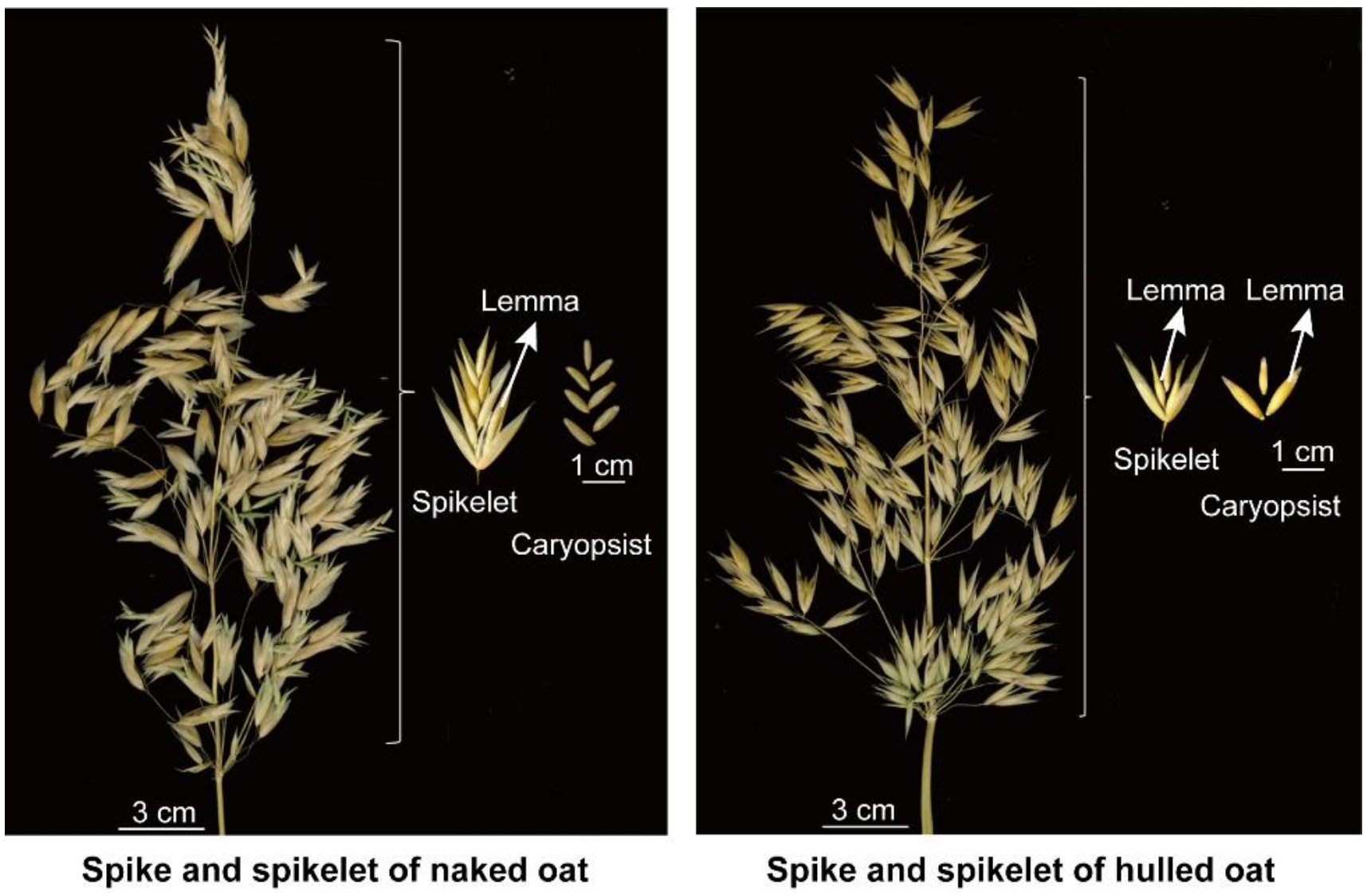

Fig. R1. Comparison of panicle and spikelet morphology between hulled and naked oats.

2) Adding a summary table of the population

This is an excellent suggestion. We will create a new Table (Revised Supplementary Tab. S1-1) in the Materials and Methods section, titled "Summary of the oat panel used in this study."

The table have clearly presented:

Accession: Numbers of oats in the seed database

Category: Wild, Landrace, Cultivar.

Subtype: Number of accessions for Hulled (H) and Naked (N) within each category.

Growth_habit: Spring habit oats or winter habit oats.

Geographic Origin: Summary of counts by major region (e.g., East Asia, Europe, Americas).

3) Clarifying the main research goal

We apologize for any lack of clarity. The primary and explicit goal is to compare hulled versus naked oats at the genetic and phenotypic levels. The analysis of geographic origin (65 countries) serves to demonstrate the global representativeness and diversity of our panel, providing context for the population structure analysis. It is not intended for direct country-to-country comparisons. The analysis by improvement level (landrace, cultivar) is crucial as it allows us to trace the domestication and improvement history of the hulled/naked trait and to understand how modern breeding has shaped their respective gene pools. This framework supports the primary hulled vs. naked comparison. We have revised the Abstract and Introduction to state this hierarchy of objectives more explicitly. The relevant revisions are as follows: [L97-L102]

“To address the genetic origin and phenotypic trait divergence between hulled and naked oats, this study leverages 127,563 high-quality genome-wide SNPs to systematically dissect the population structure, genetic differentiation, and domestication history of 666 hexaploid oat accessions from 65 countries. By comparing population differences among various subpopulations, particularly between accessions from China (the center of diversity for naked oats) and those from other geographical regions, we revealed the differentiation characteristics of hulled and naked oats with respect to genetic structure, diversity distribution, gene flow events, and domestication pathways. Furthermore, we integrate phenotypic datasets of 19 agronomic and grain quality traits evaluated across multiple environments from 2010-2021 to clarify phenotypic divergence and breeding selection preferences between hulled and naked oats. By combining selection signatures during naked oat improvement with RNA-seq data from globally representative accessions, we aimed to elucidate the genetic basis of the nutrition-yield trade-off, identify candidate genes, and establish a foundation for nutrition-oriented oat breeding.”

Throughout the manuscript, numerical values for Fst and genetic diversity are missing. The authors instead use vague terms such as “weak genetic differentiation” or “low genetic diversity.” These quantitative results should be explicitly reported in tables.

RESPONSE: We sincerely appreciate your valuable suggestion to supplement the conclusion-supporting data. Your comments precisely identify key optimization points in our research finding presentation. We have revised ambiguous conclusions (e.g., "low genetic differentiation" and "low genetic diversity") throughout the manuscript for precise, quantifiable expression, with specific values or clear ranges added to all corresponding arguments. Additionally, we have tabulated detailed supporting data on population genetic diversity (π), F_{st} values of population differentiation and Tajima’s D values as Supplementary for reference (Revised Supplementary Tab. S2). The relevant revisions are as follows: [L111-157]

“Based on 127,563 high-quality bi-allelic SNPs (MAF $\geq$ 0.05, miss $\leq$ 0.1), this study detected moderate introgression from cultivated oat to naked oat using the ABBA-BABA test and TreeMix analysis (D = 0.35, $P < 0.01$), persistent gene flow between hulled and naked oats resulted in weak differentiation (F_{st} = 0.0701) and significant genetic overlap (Figs. 1f and 2a; Tab. S2). Genetic diversity analysis revealed that hulled oat exhibited significantly higher nucleotide diversity ($\pi = 2.79 \times 10^{-6}$) and faster linkage disequilibrium decay (1.34 Mb) than naked oat ($\pi = 1.64 \times 10^{-6}$; 2.2 Mb). Together with the significantly higher proportion of long runs of homozygosity (ROH) (1–3 Mb) in naked oat (14.4%) than in hulled oat (9.5%), these results demonstrate that naked oat underwent a stronger genetic bottleneck and greater selection pressure during domestication (Figs. 1d, h; S6a, d; Tab. S2). Furthermore, by integrating Admixture cross-validation (K=1-20) with neighbor-joining (NJ) phylogenetic analysis (rooted with wild hexaploids), we classified 666 oat accessions from 65 countries and regions into 6 genetic subgroups (G1-G6) (Figs. 1a, b and S1-S3; Tab. S1). While the core germplasms of each subpopulation had clear origins, genetic admixture between hulled and naked oats and cross-regional gene flow were prevalent, with no strict geographic-genetic correspondence observed (Figs. 1and S3-S6; Tabs. S1-S3). Specifically, G1 represented African-Asian convergence, with 57 Ethiopian-Egyptian hulled oats, 7 wild relatives, and a Chinese naked landrace from Hebei. G2, a hulled oat landrace core subpopulation from the Mediterranean and West Asia, exhibited the weakest domestication selection (Tajima’s $D < 0$: 1.4%-4.8%) and

highest nucleotide diversity ($\pi = 3.31 \times 10^{-6}$). Its genetic components introgressed into other subpopulations at 7%-70%, with Gene flow analysis further confirmed moderate-to-high introgression from G2 into other subpopulations and across geographic regions (D = 0.18-0.48), underscoring G2 as a key genetic donor in the global oat germplasm network. Despite such widespread gene flow, G2 remained strongly differentiated from these subpopulations ($F_{st} > 0.15$) due to geographic isolation and divergent selection pressures. G3 is a subpopulation dominated by Chinese naked oats, which has experienced strong domestication selective pressure, with the proportion of loci showing Tajima's $D < 0$ being 11.7 to 16.3 times that of G2, and exhibits significantly reduced genetic diversity. G4 reflected trans-Pacific gene flow with 43 North American hulled oats and 41 Chinese naked oats from northern domestication hubs. Gene flow analysis revealed bidirectional introgression between North American cultivated hulled oats and lineages from East Asia, the Mediterranean, and Europe. Frequent recent gene flow was detected with Mediterranean and European hulled oats (shared ROH >1 Mb: 26.2%-37.8%), indicating that G4 serves as a genetic fusion hub for global modern breeding germplasm. Driven by such extensive cross-regional gene exchange, G4 maintains relatively high nucleotide diversity ($\pi = 2.07 \times 10^{-6}$). G5 is a Sino-Eurasian mosaic which comprising 53 European hulled cultivars and 13 Chinese naked oat landraces from Shanxi-Hebei-Inner Mongolia, exhibits the lowest nucleotide diversity ($\pi = 1.26 \times 10^{-6}$) due to strong breeding and domestication pressures. G6 is a typical geographically adjacent admixed subpopulation, comprising 54 Mediterranean hulled oat landraces, 28 European hulled oat germplasms, and 55 accessions from Asia (including 28 Chinese naked oats). Principal component analysis (PCA) supports this structure and overall genetic admixture: only G2 shows independent clustering on PC1, and G3 (naked oat core) concentrates on PC3; otherwise, extensive overlap by origin and type confirms global admixture (Fig. 1c; Tabs. S1-3)."

Overall, the figures are difficult to interpret and appear unnecessarily complex. For instance, the Pearson correlation in Fig. S6a is unclear, and the Circos plots—especially those for Fst (Fig. 1f, Fig. S4b, and Fig. S4e)—are hard to follow. Fst values and other intra/inter-genetic metrics should be summarized in a table.

RESPONSE: We thank you for your suggestions to improve the presentation and readability of several figures. We fully acknowledge the limitations of the original formats and have systematically revised and improved them, as detailed below:

1) Given the suboptimal clarity of Pearson's correlation coefficients in Supplementary Fig. S6a, we have reorganized the figure (Fig. R2 below; also Revised Manuscript Fig. S9b) and provided the corresponding detailed data in the supplementary file (Revised Supplementary Tab. S5-2).

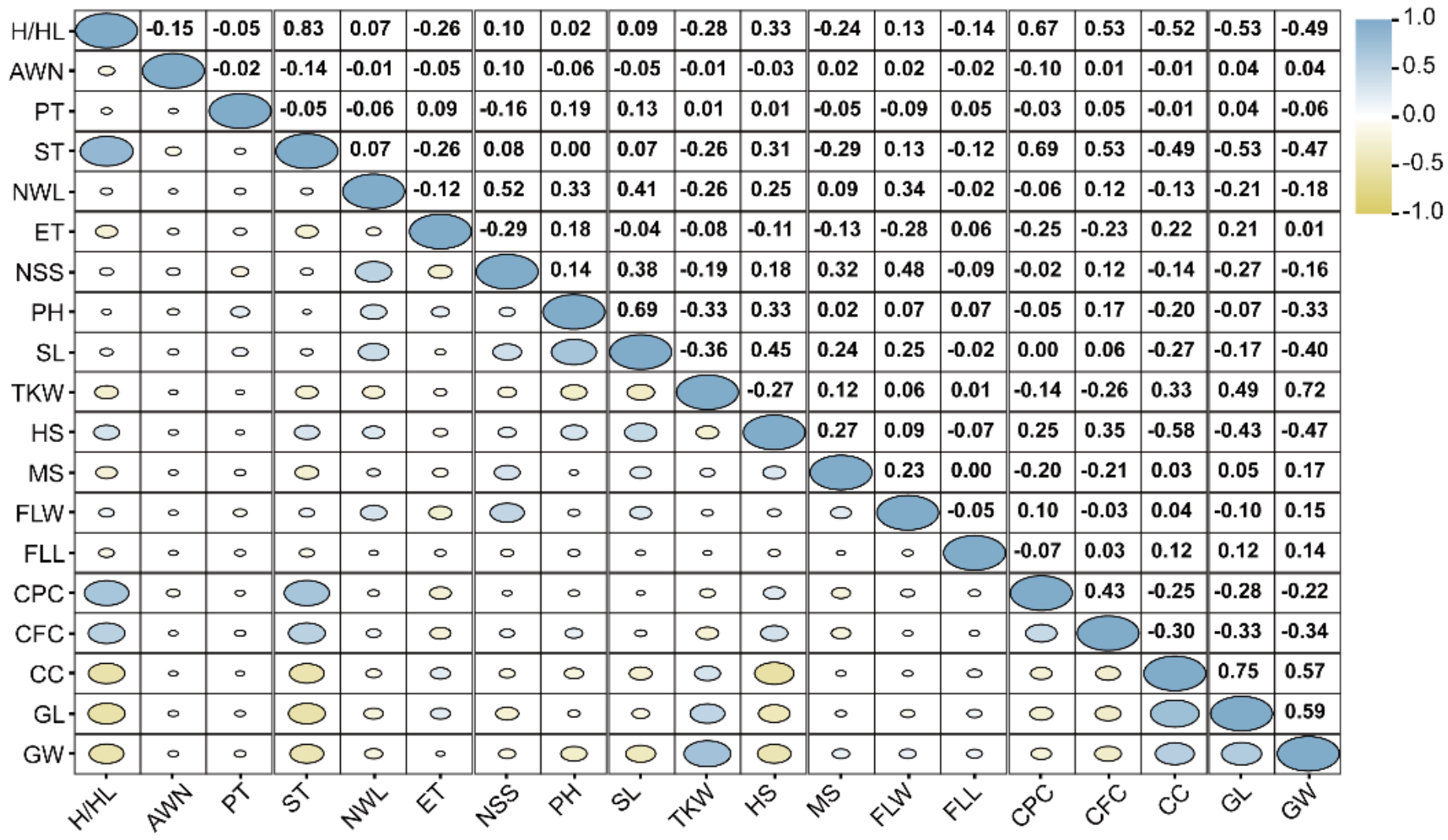

Fig. R2 Pearson correlation analysis heatmap of 17 agronomic traits and 2 grain quality-related traits in oat. The traits and their corresponding abbreviations are listed as follows: heading stage (HS), growth period (GP), spike type (ST), spikelet type (SLT), effective tiller number (ET), plant height (PH), spike length (PL), flag leaf length (LFL), flag leaf width (WFL), stem diameter (SD), node number (NN), awn presence (AWN), spikelet number (SN), Hulled/Naked (H/N), grains per spike (GN), thousand-grain weight (TGW), grain length (GL), grain width (GW), grain circumference index (GCI); crude protein content (CPC), crude fat content (CFC).

2) Given the limited interpretability of the circular plots in the original manuscript, particularly for presenting F_{st} values in Fig. 1f and Supplementary Figs. S4b and S4e, we have redesigned these figures (Fig. R3 below, also Revised Manuscript Figs. 1h, i and S6a). The corresponding detailed results have also been compiled into a supplementary table for reference (Revised Supplementary Tab. S2).

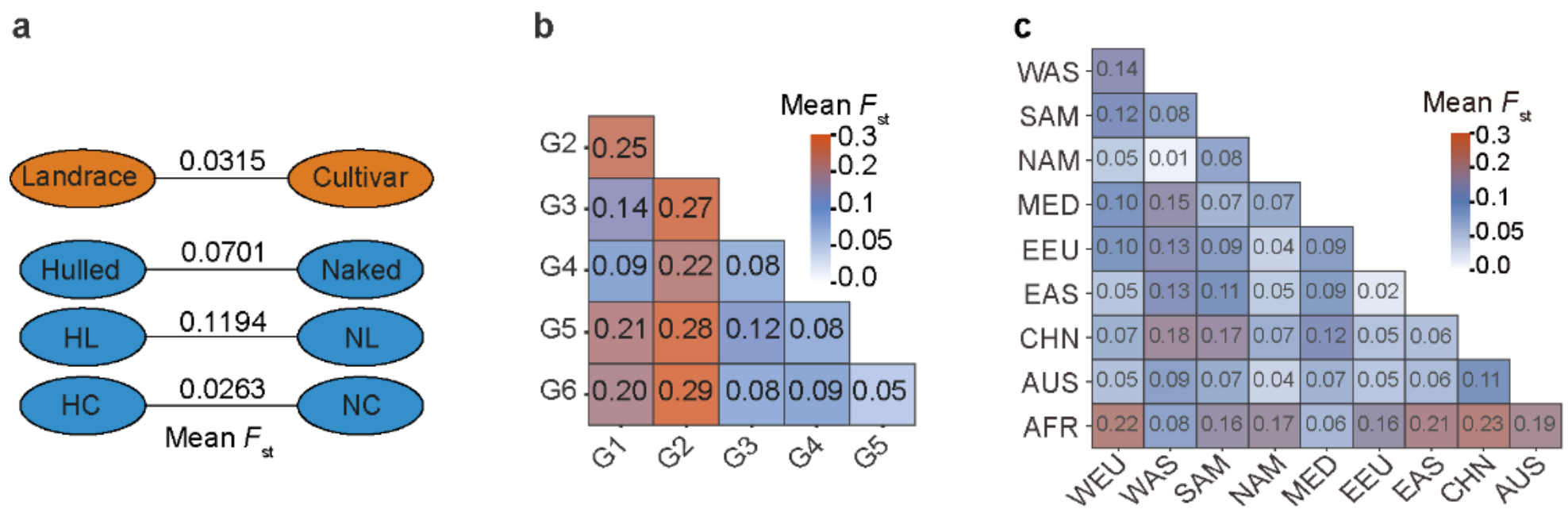

Fig. R3 Genetic differentiation index (F_{st}) analysis. (a) Genetic differentiation between landraces and bred cultivars, hulled/naked oat landraces, and hulled/naked oat bred cultivars (Revised manuscript Fig. 1h); (b) Genetic differentiation among 6 genetic subpopulations delineated by NJ clustering (Revised manuscript Fig. 1i); (c) Genetic differentiation among nine geographic subpopulations classified by geographic origin (Revised manuscript Fig. S6a).

3) Additionally, in addition to adjusting the presentation of the aforementioned figures to enhance their readability, we have systematically revised the presentation of all figures throughout the manuscript to reduce complexity and more effectively convey the core findings of the study, which are available for your review in the revised manuscript.

I have not access any supplementary tables, and many of the results described in the manuscript are missing. All supplementary materials should be deposited in Figshare or a similar public repository.

RESPONSE: We apologize for not providing the supplementary materials in detail earlier. All supplementary figures and tables have now been compiled and submitted with the revised manuscript. For ease of reference, the location of each supplementary item is indicated directly following the relevant conclusion in the main text.

1. Population Structure

In the PCA (Fig. 1d), there is a clear separation of a cluster along PC1, but the authors do not provide any information about this group of accessions.

RESPONSE: We greatly appreciate your profound insights into this scientific issue, which have been pivotal to clarifying the oat population structure. In the revised manuscript, we redrew the 3D-PCA plot for the first three principal components (Fig. R4; also Revised Manuscript Fig. 1c). We also elaborated on the geographic origins, germplasm types (L154-157) and phenotypic differentiation characteristics (L245-252) of accessions forming independent clusters in the PCA results. The relevant details are as follows:

L154-L157: "Principal component analysis (PCA) supports this structure and overall genetic admixture: only G2 shows independent clustering on PC1, and G3 (naked oat core) concentrates on PC3; otherwise, extensive overlap by origin and type confirms global admixture (Fig. 1c; Tabs. S1-3)."

L245-L252: "Phenotypic PCA revealed population differentiation consistent with marker-based analysis, reflecting the breeding characteristics of distinct genetic backgrounds and yield-quality divergence between hulled and naked oats. Consistent with this observation, G3, dominated by naked oats, showed the highest crude protein (15.06%) and crude fat (6.22%) contents. G2, composed mainly of Mediterranean and West Asian hulled landraces, exhibited superior thousand-kernel weight (26.48 g) and kernel length (9.45 mm), reflecting a large-kernel, high-yield phenotype distinct from G3."

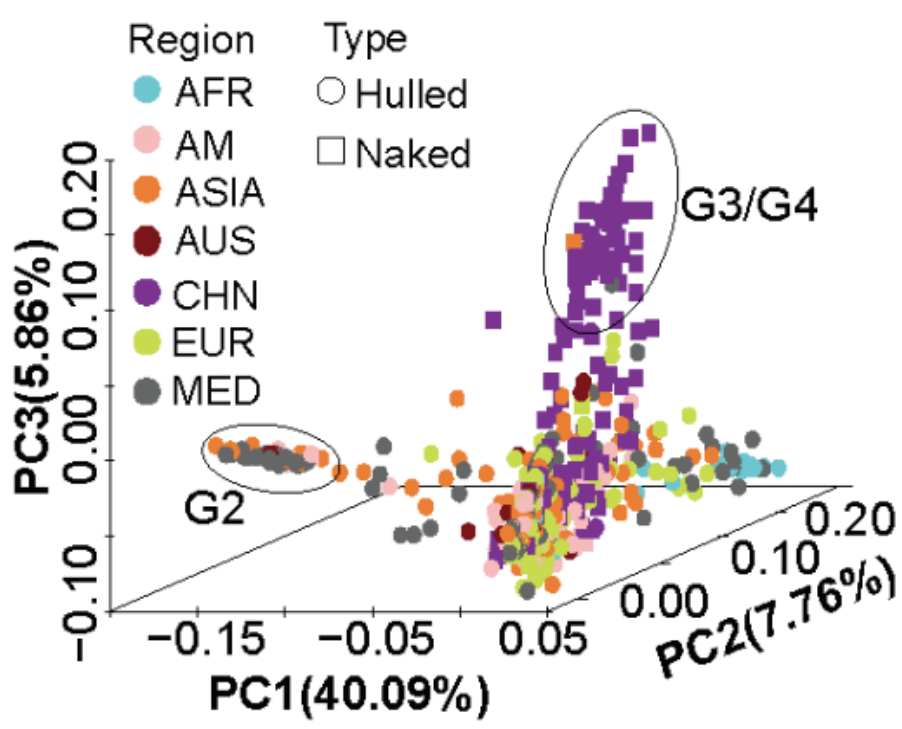

Fig. R4. 3D-PCA plot of 666 oat accessions based on 127,563 high-quality SNP markers (Revised Manuscript Fig. 1c).

L116: What statistical test supports the claim of breeding introgression in GVI, which includes 13 Chinese naked oats and 61 hulled cultivars from Oceania and North America?

RESPONSE: We appreciate your pointing out this issue and sincerely apologize for the insufficient evidence and imprecise description previously provided to support the relevant conclusion. Regarding the conclusion that the original GVI (13 Chinese naked oat accessions and 61 cultivar hulled oats from Oceania/North America) underwent breeding introgression, we reconstructed the NJ phylogenetic tree of the tested accessions with wild hexaploid oats as the outgroup and optimized the population structure analysis, which revealed that the original GVI was clearly corresponding to G3 (Chinese naked oats) and G4 (North American cultivars). In the revised manuscript, we verified the genetic introgression between the two groups through multiple statistical tests: Admixture analysis (K = 2-7) showed shared genetic components between Group 3 and Group 4 (Fig. R5 below; also Revised Manuscript Fig. S3); Treemix gene flow analysis detected significant gene flow signals between them (Fig. R6 below; also Revised Manuscript Fig. 1j and S6g); the ABBA-BABA test ($D > 0$ and $Z > 3$) confirmed the statistical reliability of genetic introgression (Fig. R8 below; also Revised Manuscript Fig. S5b, c); and shared haplotype analysis identified high-frequency shared fragments between the two groups, further supporting the introgression characteristics (Fig. R7 below; also Revised Manuscript Fig. S4b, e). These consistent results from multi-method cross-validation fully confirm the breeding introgression between G3 (Chinese naked oats) and G4 (North American cultivars), thus supplementing the statistical evidence for the original conclusion. We have described these findings in the Results section (L141-148) and further discussed in the Discussion section (L408-411) of the revised manuscript. The relevant details are as follows:

L141-L148: “G4 reflected trans-Pacific gene flow with 43 North American hulled oats and 41 Chinese naked oats from northern domestication hubs. Gene flow analysis revealed bidirectional introgression between North American cultivated hulled oats and lineages from East Asia, the Mediterranean, and Europe. Frequent recent gene flow was detected with Mediterranean and European hulled oats (shared ROH >1 Mb: 26.2%-37.8%), indicating that G4 serves as a genetic fusion hub for global modern breeding germplasm. Driven by such extensive cross-regional gene exchange, G4 maintains relatively high nucleotide diversity ($\pi = 2.07 \times 10^{-6}$).”

L408-L411: “In addition, modern breeding breaking geographic barriers through introductions/hybridizations, with G4 (North American hulled cultivars) serving as a "bridge population" that maintained diversity ($\pi = 2.07 \times 10^{-6}$) while potentially accumulating favorable alleles for yield and adaptability (Figs. 1f, S10; Tab. S5-5).”

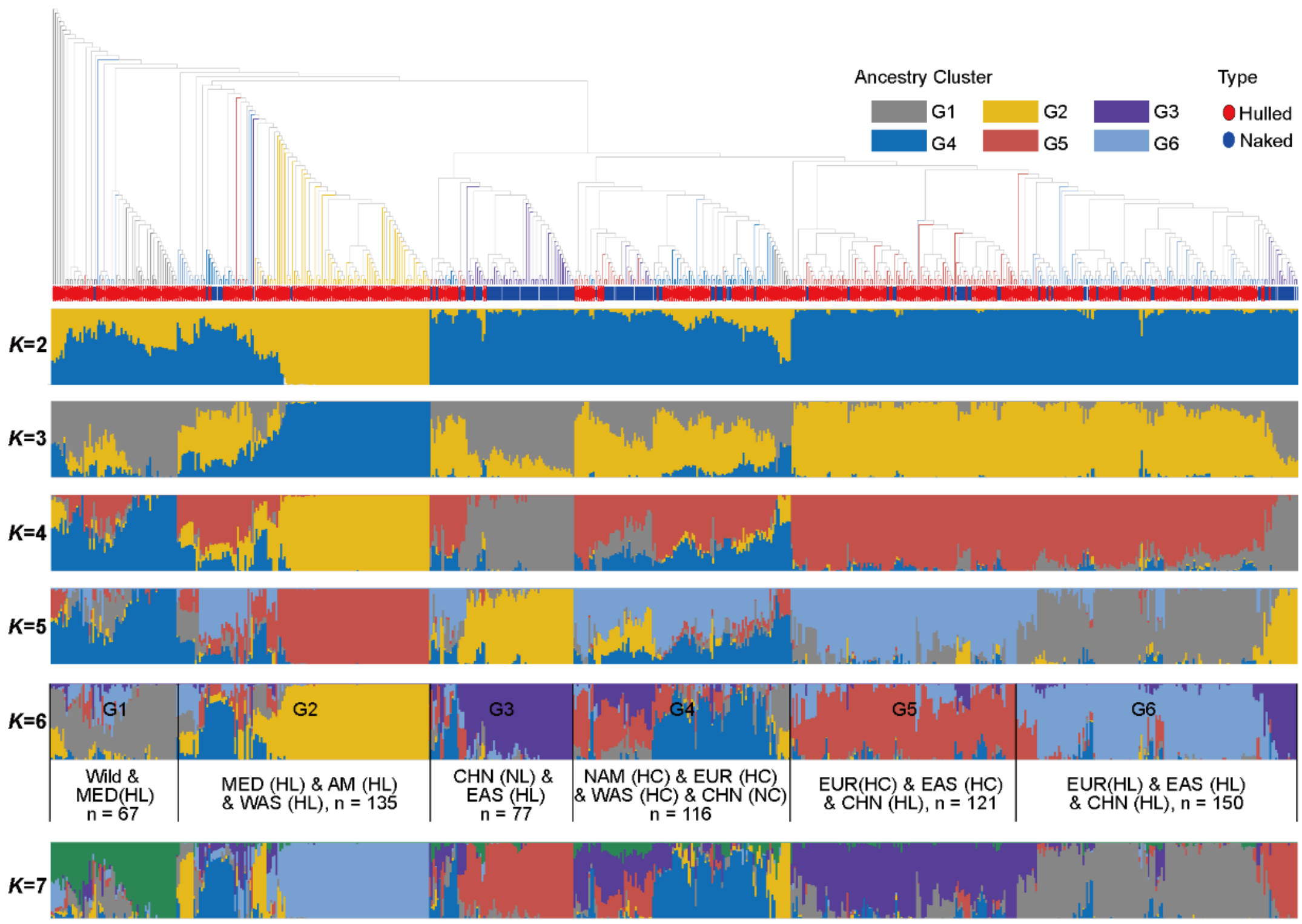

Fig. R5. Rooted NJ tree and Admixture analysis (*K*=1-7) of 666 oat accessions based on 127,563 high-quality SNPs (Revised Manuscript Fig. S3).

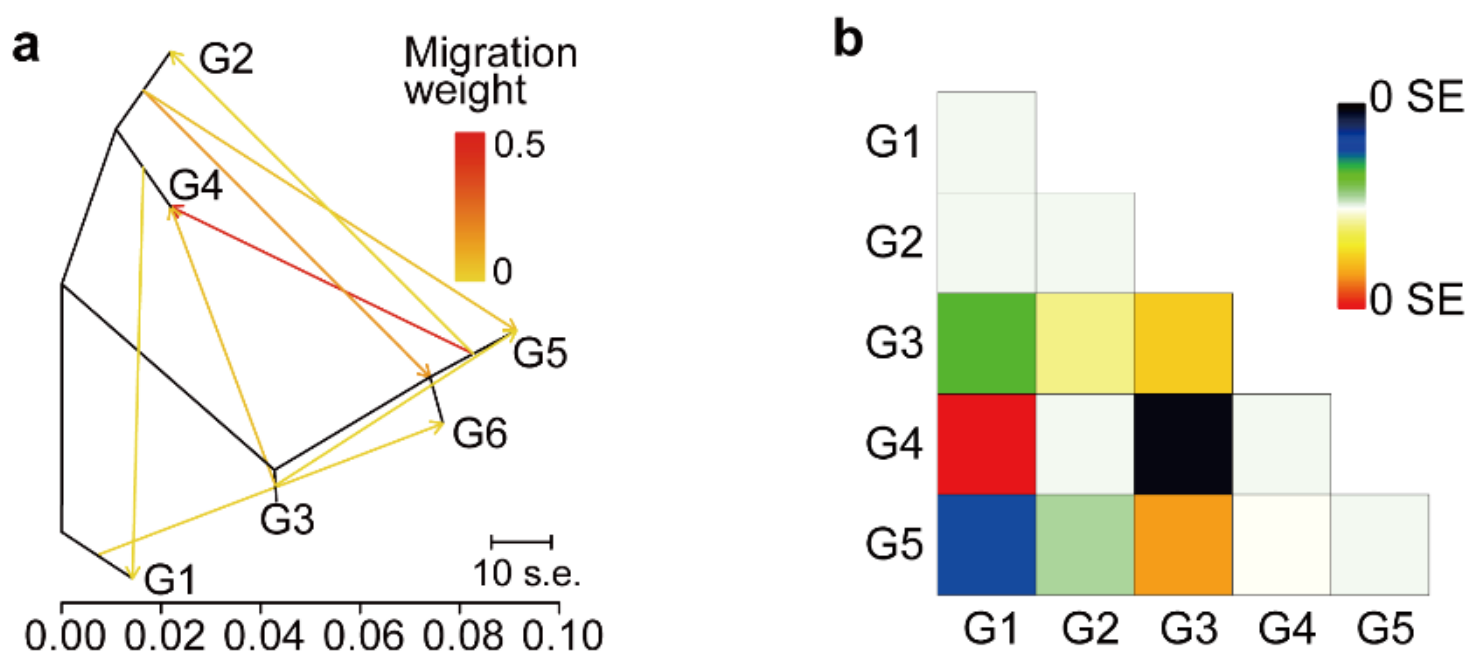

Fig. R6 Gene flow analysis based on NJ clustering subpopulations. (a) Schematic of phylogenetic topology and gene flow events (red arrows indicate the direction; Revised Manuscript Fig. 1j). (b) Residual plot after fitting eight gene flow events (Revised Manuscript Fig. S6g).

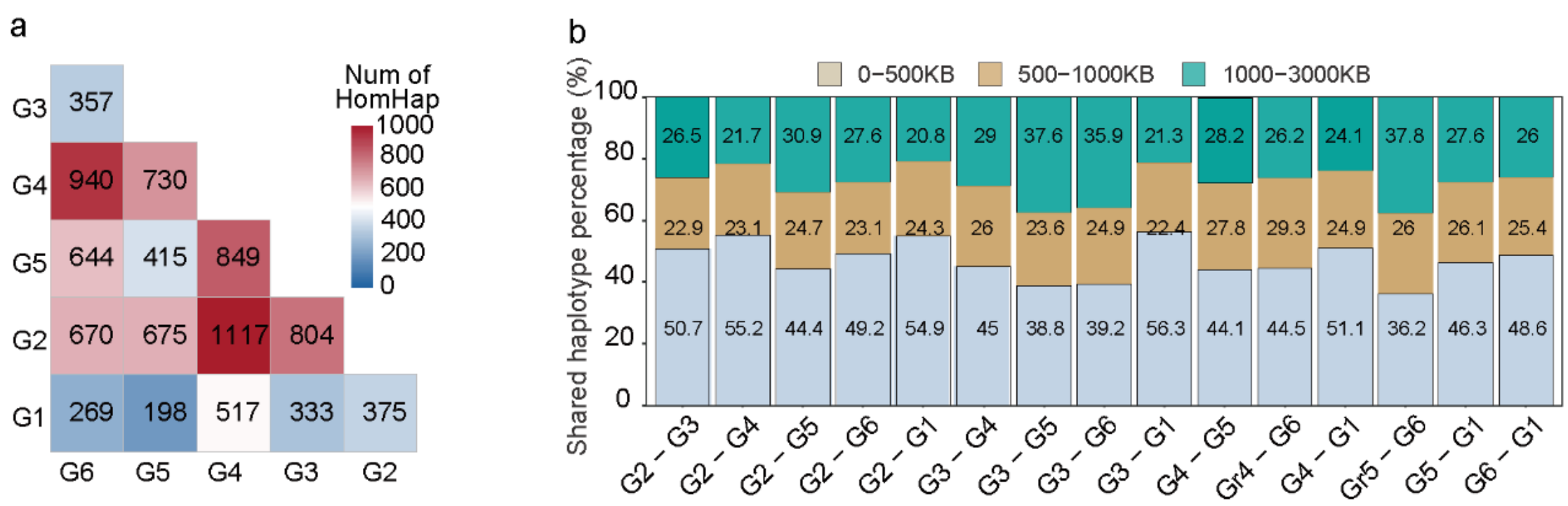

Fig. R7 Statistical analysis of shared homozygous haplotypes among 6 genetic subpopulations (Revised Manuscript Fig. S4). (a) Heatmap of shared haplotypes among subpopulations (Revised Manuscript Fig. S4b). (b) Stacked proportion plot of shared haplotypes with different lengths in each subpopulation combination (Revised Manuscript Fig. S4e).

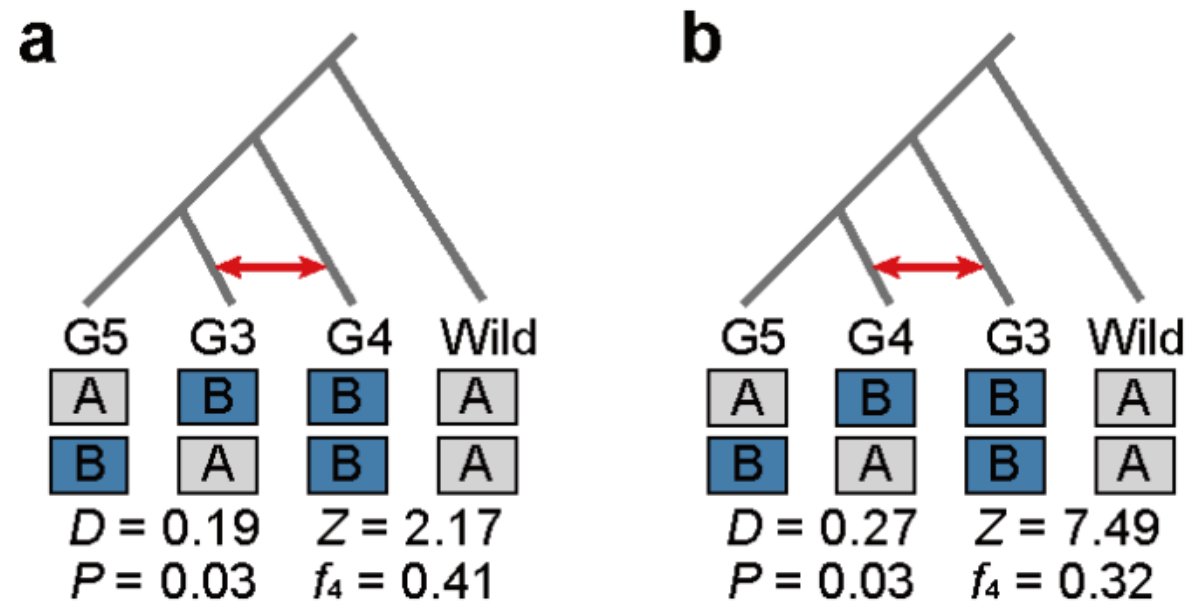

Fig. R8 Gene flow test between G3 and G4 subpopulations based on the ABBA-BABA model (Revised Manuscript Fig. S5). (a) *D* statistic results for testing gene flow between G4 and the sister groups of G3 and G5 (Revised Manuscript Fig. S5b). (b) *D* statistic results for testing gene flow between G3 and the sister groups of G4 and G5 (Revised Manuscript Fig. S5c).

2. Domestication Pathway

Why did the authors not use the classical definition of gene flow, Nm = (1 – Fst)/(4 × Fst)?

RESPONSE: We greatly appreciate your valuable suggestions regarding the gene flow calculation method. This study did not adopt the classic formula $Nm=(1-F_{st})/(4\times F_{st})$ derived from F_{st}, the core reason being that this method relies on stringent theoretical assumptions including the infinite island model and population migration-drift equilibrium (*Evolution* 1984 https://doi.org/10.1111/j.1558-5646.1984.tb05657.x). However, the oat populations in this study exhibit characteristics of high admixture, multidirectional gene flow and intense artificial selection, which violate the aforementioned assumptions; blind application of the formula would thus lead to biased results. After fully considering your comments, we used Treemix to clarify the direction of gene flow, supplemented the *D*-statistic (ABBA-BABA) to verify genetic introgression signals, and combined shared haplotype analysis to confirm the characteristics of genetic material sharing. These methods are more suitable for population analysis scenarios related to crop domestication and breeding, and multi-dimensional cross-validation ensures the reliability of our results.

L135: The authors mention "subgroup divergence chronology shows Mediterranean hulled groups (G2/G3)," but do not provide collection or development dates for all accessions. What evidence supports this statement?

RESPONSE: We sincerely appreciate your constructive suggestion. To support the conclusion of the subpopulation differentiation timeline, we have supplemented the collection locations and database accession numbers for all tested germplasm in the Supporting Information (Tab. S1-2). These detailed passport data provide essential geographic and resource information for evaluating the evolutionary and differentiation patterns among different oat groups, and can be referred to for further verification.

Of the 19 evaluated traits, 17 are agronomic and only two are quality traits (crude protein and crude fat content). Therefore, it is difficult to draw strong conclusions regarding quality.

RESPONSE: We greatly appreciate your valuable comments. We fully acknowledge that assaying only two quality traits (crude protein and crude fat content) cannot support robust systematic conclusions on comprehensive oat quality, and apologize for the

imprecise wording in the original manuscript. Comprehensive phenotypic characterization of full oat quality traits is beyond the core scope of this study, which focuses on the population genetic structure, domestication history and genomic basis of agronomic trait differentiation in oats. The two selected quality traits are core nutritional quality indicators recognized in prior studies, and our analysis was performed based on our available validated phenotypic dataset, which can reflect basic nutritional quality differences among tested accessions to a certain extent. In the revised manuscript, we have rigorously revised all relevant expressions to eliminate over-interpretation and avoid ambiguity.

L693: I suggest replacing Fig. S6b with a table showing the eigenvectors (Fig. 3d), since the current table is difficult to interpret and fails to capture genetic variance effectively.

RESPONSE: We greatly appreciate your valuable suggestions, which have been highly helpful for the clear interpretation of our principal component analysis (PCA) results based on phenotypic data. We have deleted this figure and replaced it with a more readable eigenvector table (Table R1 below; Revised supplementary Tab. S5-4).

Tab R1 Top 10 principal component scores of PCA based on 19 morphological markers (Revised supplementary Tab. S5-4)

Trieats	PC1	PC2	PC3	PC4	PC5	PC6	PC7	PC8	PC9	PC10
GW	0.886	-0.406	0.145	0.066	0.028	-0.05	-0.057	-0.041	0.038	0.012
GC	0.884	-0.407	0.158	0.069	0.033	-0.051	-0.063	-0.051	0.042	0.006
GL	0.882	-0.407	0.165	0.068	0.036	-0.05	-0.065	-0.053	0.042	0.006
CFC	0.864	-0.362	0.144	0.082	0.007	-0.048	-0.022	-0.06	0.031	0.002
PH	0.312	0.703	0.12	0.099	0.205	-0.305	-0.064	-0.179	0.034	-0.076
SL	0.241	0.67	-0.032	0.306	0.215	-0.405	-0.046	-0.201	-0.007	-0.058
HS	0.526	0.629	0.038	-0.362	-0.114	0.244	-0.052	0.034	0.034	-0.245
NWL	0.352	0.616	0.156	0.337	-0.169	0.208	0.035	0.251	-0.104	0.044
FLW	0.518	0.589	0.036	-0.213	-0.162	0.354	-0.098	0.109	-0.069	-0.248
TKW	-0.127	-0.485	0.349	0.101	-0.034	0.173	-0.377	0.417	-0.074	-0.02
FLL	0.284	0.449	0.126	-0.443	0.044	0.232	0.129	0.117	-0.005	0.104
MS	-0.21	0.094	0.728	0.14	-0.322	-0.238	0.22	0.204	0.035	0.024
H/HL	0.373	-0.044	-0.72	-0.059	-0.217	-0.092	0.095	0.253	0.266	0.11
ST	0.363	-0.018	-0.628	0.013	-0.276	-0.221	0.48	0.063	0.28	-0.069
ET	-0.018	0.063	0.581	-0.263	-0.326	-0.206	0.047	0.121	-0.174	0.2
NSS	0.126	0.372	-0.052	0.748	-0.185	0.1	0.624	0.08	-0.51	0.045
PT	0.042	0.275	0.105	-0.178	0.583	-0.273	-0.066	0.607	0.15	0.041
CPC	0.326	-0.149	0.038	-0.121	0.363	0.137	0.285	-0.054	0.568	-0.053
AWN	-0.092	-0.037	0.053	0.429	0.343	0.507	-0.108	-0.139	0.107	0.628
CE rate%	23.47	41.278	52.185	60.086	65.98	71.743	76.827	81.395	85.746	88.919

3. Phenotypic Analysis
Table 5-4b. Scores of the top ten principal components from the PCA dimensionality reduction analysis of 19 morphological markers
There is no multi-environment genetic modeling or other analyses that elucidate how divergent selection drives yield–quality trade-offs or environmental adaptation between hulled and naked oats.

The authors do not include analyses of broad-sense heritability or genetic correlations among traits.

RESPONSE: We greatly appreciate the two aforementioned revision suggestions. In response to these issues, we have completed a series of supplementary analyses, and further refined content interpretation and conclusion synthesis in the revised manuscript. The detailed revisions are provided as follows:

1）Phenotypic variations of 12 growth and development traits, 5 grain yield-related traits and 2 grain quality traits measured on test materials across multiple environments were fitted with a mixed linear model: $y = X\beta + Z_g g + Z_{ge} ge + \varepsilon$. The likelihood ratio test (LRT_P) and Bayesian information criterion (BIC) were used to screen the optimal genetic model: LRT_P verified the significance of random effects by calculating $LRT = -2(\log L_{\text{simplified model}} - \log L_{\text{full model}})$, while BIC was applied to select the optimal non-nested model via the formula $BIC = -2\log L + k\log n$ (where k is the number of parameters and n is the sample size) (*Genetics and analysis of quantitative traits* 1998 https://doi.org/10.1017/S0016672398219732; *Introduction to quantitative genetics* 1996 https://doi.org/10.1016/0168-9525(96)81458-2). Furthermore, based on the genotypic variance (V_g), genotype × environment interaction variance (V_{ge}) and residual variance (V_ε) estimated by the optimal genetic model, the broad-sense heritability (H^2) of each trait was calculated using the formula $H^2 = V_g/(V_g + V_{ge}/E + V_\varepsilon/(R \times E))$. Detailed methods are as follows: [L492-508]

“Phenotypic variations of 12 growth and development traits, 5 grain yield-related traits and 2 grain quality traits measured on test materials across multiple environments were fitted with a mixed linear model: $y = X\beta + Z_g g + Z_{ge} ge + \varepsilon$. Here, y represents the vector of phenotypic observed values, β denotes the fixed effects including environment and experimental replication, g is the random genotypic effect, ge refers to the random genotype × environment interaction effect, and ε is the random residual effect; X, Z_g and Z_{ge} are the corresponding design matrices. The likelihood ratio test (LRT_P) and Bayesian information criterion (BIC) were used to screen the optimal genetic model: LRT_P verified the significance of random effects by calculating $LRT = -2(\log L_{\text{simplified model}} - \log L_{\text{full model}})$, while BIC was applied to select the optimal non-nested model via the formula $BIC = -2\log L + k\log n$ (where k is the number of parameters and n is the sample size) (*Genetics and analysis of quantitative traits* 1998 https://doi.org/10.1017/S0016672398219732; *Introduction to quantitative genetics* 1996 https://doi.org/10.1016/0168-9525(96)81458-2). Furthermore, based on the genotypic variance (V_g), genotype × environment interaction variance (V_{ge}) and residual variance (V_ε) estimated by the optimal genetic model, the broad-sense heritability (H^2) of each trait was calculated using the formula $H^2 = V_g/(V_g + V_{ge}/E + V_\varepsilon/(R \times E))$, in which E is the number of environments and R is the number of replications in a single environment.”

2) Using genotypic and phenotypic data of the tested materials, we employed GCTA (*American Journal of Human Genetics* 2011 https://doi.org/10.1016/j.ajhg.2010.11.011) with the bivariate GREML model. Genome-wide SNPs were used to construct the GRM, and additive genetic variances and covariances between two traits were estimated to calculate genetic correlations, which reflect phenotypic associations caused by shared genetic factors. The heatmap of genetic correlations among traits is shown in Fig. R9 below (also Revised Manuscript Fig. S9a).

Tab. R2 G×E interaction effects and broad-sense heritability for 19 agronomic traits (Revised Supplementary Tab. S5-1).

Trait	Best_Model	Var_Line	Var_Year	Var_Loc	Var_Res	H2	LRT_*P*	BIC
H/N	ID	0.18	0	0	0.05	0.96	0.03	-18184.35
AWN	ID+YEAR	1.11	0	0	0.1	0.78	0.0001	4822.54
ST	ID+YEAR	0.63	0.01	0	0.06	0.95	0.0001	3812.9
SLT	ID	0.18	0	0	0.02	0.95	0.0001	-5345.56
PH	ID+YEAR+LOC	239.71	40.46	625.05	20	0.41	4.30E-195	31936.24
PL	ID+YEAR+LOC	47.61	0	8.43	5	0.81	4.68E-206	19009.41
HS	ID+YEAR+LOC	63.79	42.21	64.2	6	0.53	7.11E-162	23778.96
GP	ID+YEAR+LOC	70.33	46.54	70.77	7	0.53	7.11E-162	24082.63
LFL	ID+YEAR+LOC	8.18	0	15.71	1.3	0.55	4.91E-46	12224.3
WLF	ID+YEAR+LOC	0.07	0.12	0.02	0.03	0.56	1.11E-61	2367.57
ET	ID+YEAR+LOC	18.64	3.4	0	1.8	0.62	2.29E-36	31179.7
NN	ID+YEAR+LOC	0.37	0	0	0.03	0.8	8.39E-115	8329.62
SN	ID+YEAR+LOC	164.84	24.04	0	36	0.71	2.29E-86	25354.43
GN	ID+YEAR+LOC	1846.1	698.95	949.94	180	0.78	6.69E-41	11937.03
TGW	ID+YEAR+LOC	17.38	1.49	2.48	2	0.75	8.80E-48	9431.61
GL	ID+YEAR	0.59	0.31	0	0.06	0.79	0.587424	8786.27
GW	ID+YEAR+LOC	0.07	0.01	0	0.01	0.9	1.94E-203	-317.13
CPC	ID+YEAR	0.02	0	0	0	0.75	0.000123	1398.82
CFC	ID+YEAR	0	0.01	0	0	0.69	0.00001	-3206.07

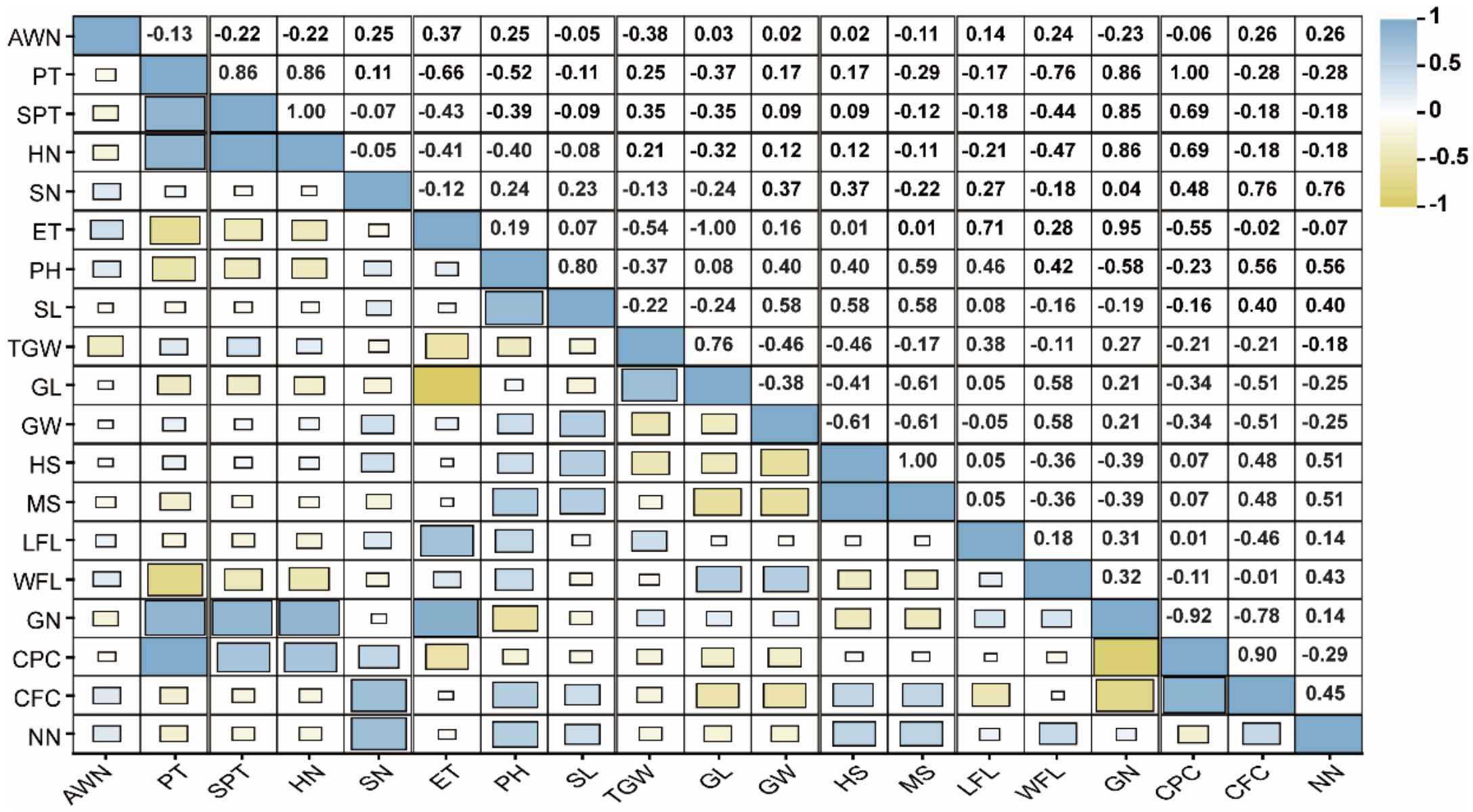

Fig. R9 Genetic correlation heatmap of 17 agronomic traits and 2 grain quality-related traits in oat (Revised Manuscript Fig. S9a).

3) We sincerely appreciate your identification of this study's limitation in elucidating how directional selection drives trait divergence between hulled and naked oats. We have thoroughly restructured the logical flow of the Results section in the revised manuscript to clarify the analytical workflow. To further delineate the role of directional selection in mediating the yield-quality trade-off and divergence in environmental adaptability, we supplemented multi-dimensional omics integrative analyses as follows: First, we performed colocalization of genome-wide selection scan results with published oat GWAS datasets, systematically characterized genetic variation within selected genomic regions, and linked these variations to yield, quality, and adaptive phenotypes. Second, we integrated multiple public transcriptome datasets related to oat grain development and abiotic stress, and by incorporating phenotypic divergence traits

of hulled and naked oats, developed an analytical framework of "selection divergence -expression divergence- phenotypic association", thereby systematically delineating the evolutionary patterns of trait divergence mediated by directional selection. All these supplementary analyses and related content are fully presented in the Results section of the revised manuscript (L258-355).

L688: Agronomic traits should be grouped and discussed by yield and quality.

RESPONSE: We sincerely appreciate your revision suggestions. To address these concerns, we first refined the overall figure presentation of the Phenotypic Divergence Analysis section in the revised manuscript, presenting trait divergences between hulled and naked oats individually (Figs. R10-11 below; also Revised Manuscript Fig. 3), with logical reorganization of the analytical results. The detailed revisions are as follows: [L226-238]

"Multi-environment evaluation of the above traits in 504 hulled and 162 naked oat accessions revealed significant domestication-driven phenotypic divergence (Fig. 3). For growth and development traits, naked oats exhibited 44.9% multiflowered spikelets and long-awned phenotypes (20.2% in hulled oats), indicating divergent awn trait selection; both hulled and naked oats favored umbellate spikes to optimize light interception and lodging resistance (Fig. 3b; Tab. S5-3). In terms of phenological and yield-related traits, hulled oats exhibited earlier heading and extended filling periods (11 days longer), which facilitated photosynthetic accumulation and superior yield traits (thousand-kernel weight increased by 21.5%; grain length increased by 15.2%). Critically, naked oats showed 23.2% fewer grains per spikelet despite higher spikelet counts, implicating seed-setting efficiency as the yield-limiting bottleneck. In contrast, naked oats achieved nutritional superiority, with crude protein increasing by 25.5% ($P < 0.001$) and crude fat increasing by 16.9% ($P < 0.001$) (Fig. 3c; Tab. S5-3)."

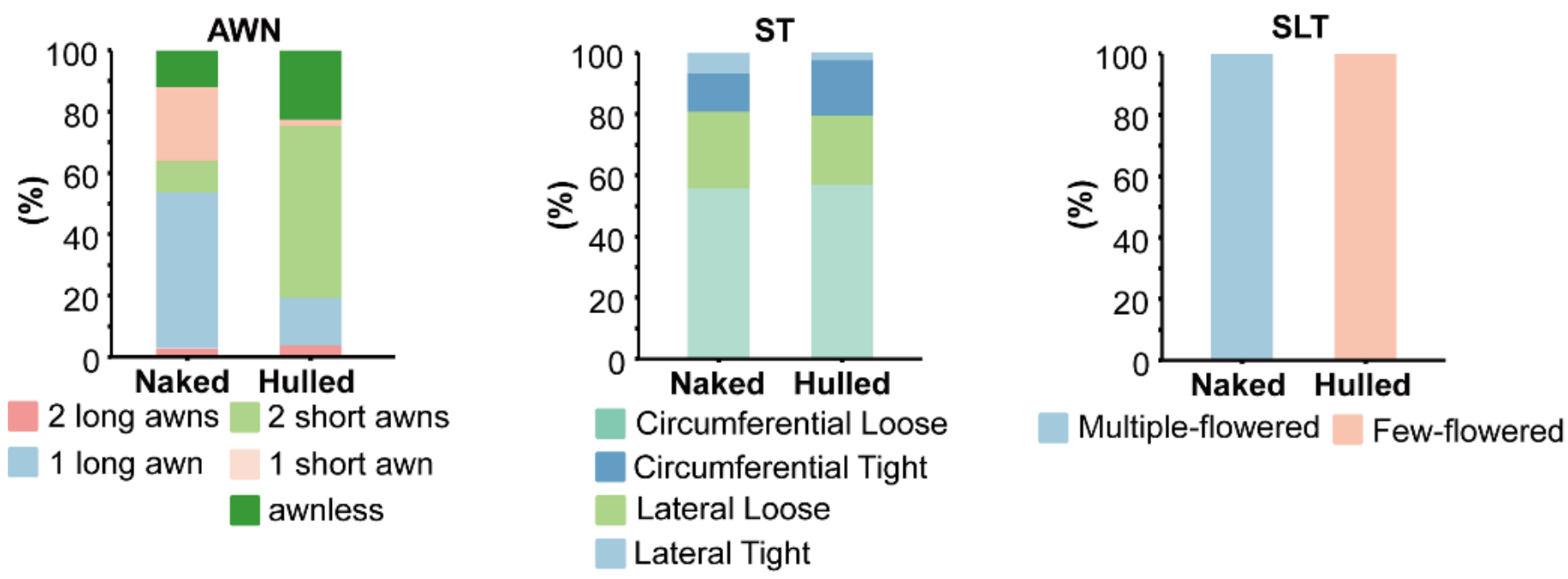

Fig. R10 Proportion of accessions with different awn types, panicle types and spikelet morphologies in hulled and naked oats (Revised Manuscript Fig. 3b)

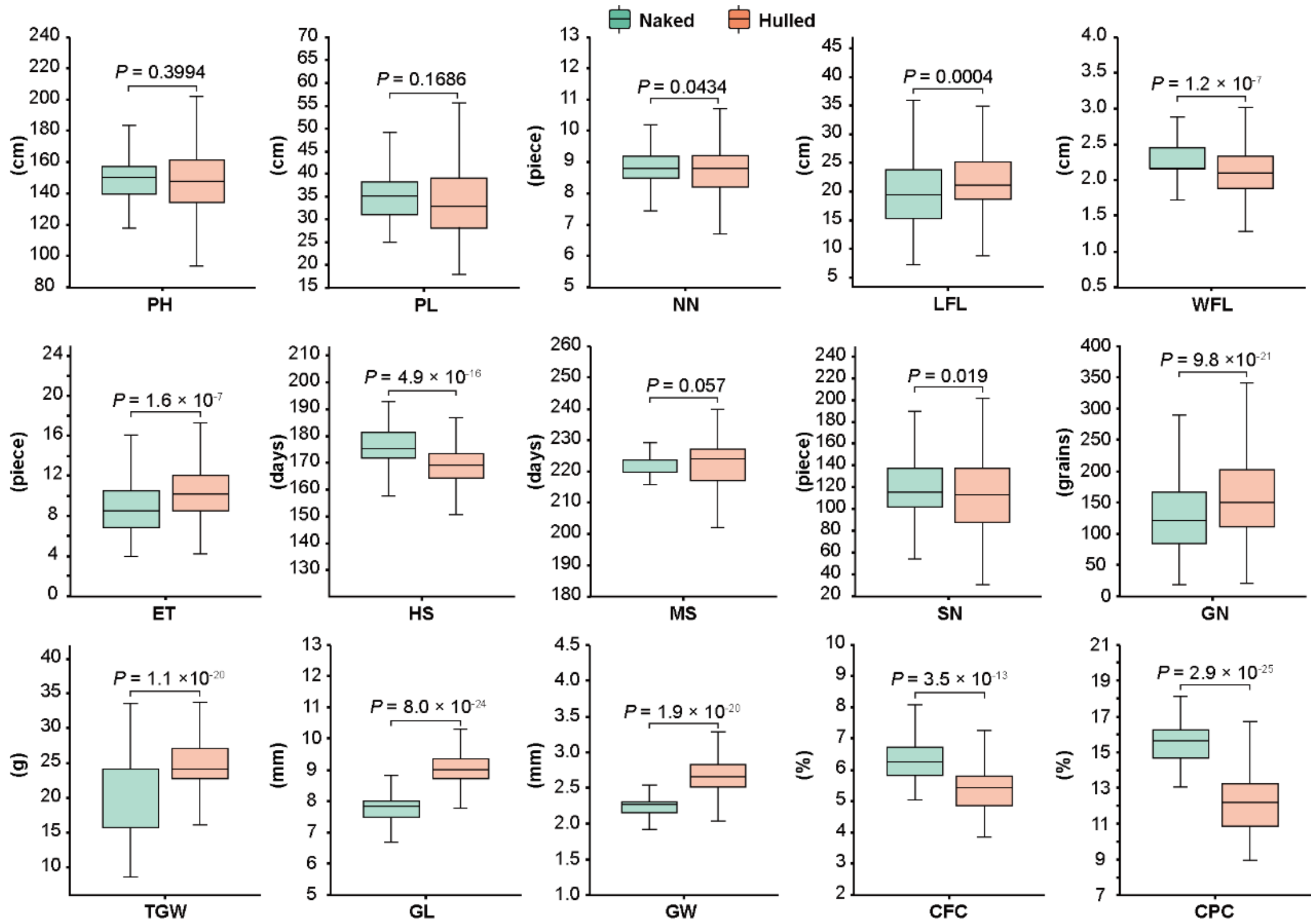

Fig. R11 Box plots showing phenotypic differences between hulled and naked oats for 8 growth-related traits (PH, PL, NN, LFL, WFL, ET, HS, MS), 5 grain-yield traits (SN, GN, TGW, GL, GW), and 2 grain-quality traits (CFC, CPC) (Revised Manuscrip Fig. 3c).

L222: "The results suggest that naked oats and hulled oats have developed distinct stress response traits through directional selection in their respective cultivation environments." However, no stress conditions were tested, and the functional enrichment analysis is insufficient to support this conclusion.

RESPONSE: We sincerely thank you for identifying this issue and acknowledge that the original claim regarding hulled and naked oats developing distinct stress response traits through directional selection was overstated, particularly in the absence of direct evidence from stress treatment experiments. To address this, we have supplemented our analysis with multidimensional omics data and revised the manuscript accordingly.

1) We integrated published oat GWAS data to perform colocalization analysis between selective sweep regions and abiotic stress associated QTLs (Fig. R12 below; also Revised Manuscrip Fig. 4b). Among 178 genes linked to five drought stress QTLs, 117 showed signals of directional selection in naked oats and were significantly enriched in stress response pathways, including drought, high temperature, and salt alkali tolerance (GO:0009651, GO:0009408, GO:0009266, ko00999; $P < 0.01$) (Fig. 4b; Tab. S6 6), suggesting naked oats may have accumulated adaptive genetic variants related to stress survival. The detailed revisions are as follows: [L292-L294)

"Moreover, 117 of 178 genes linked to five drought-responsive QTLs showed directional selection (Fig. 4b; Tab. S6-6), implying enhanced adaptation to specific ecological niches."

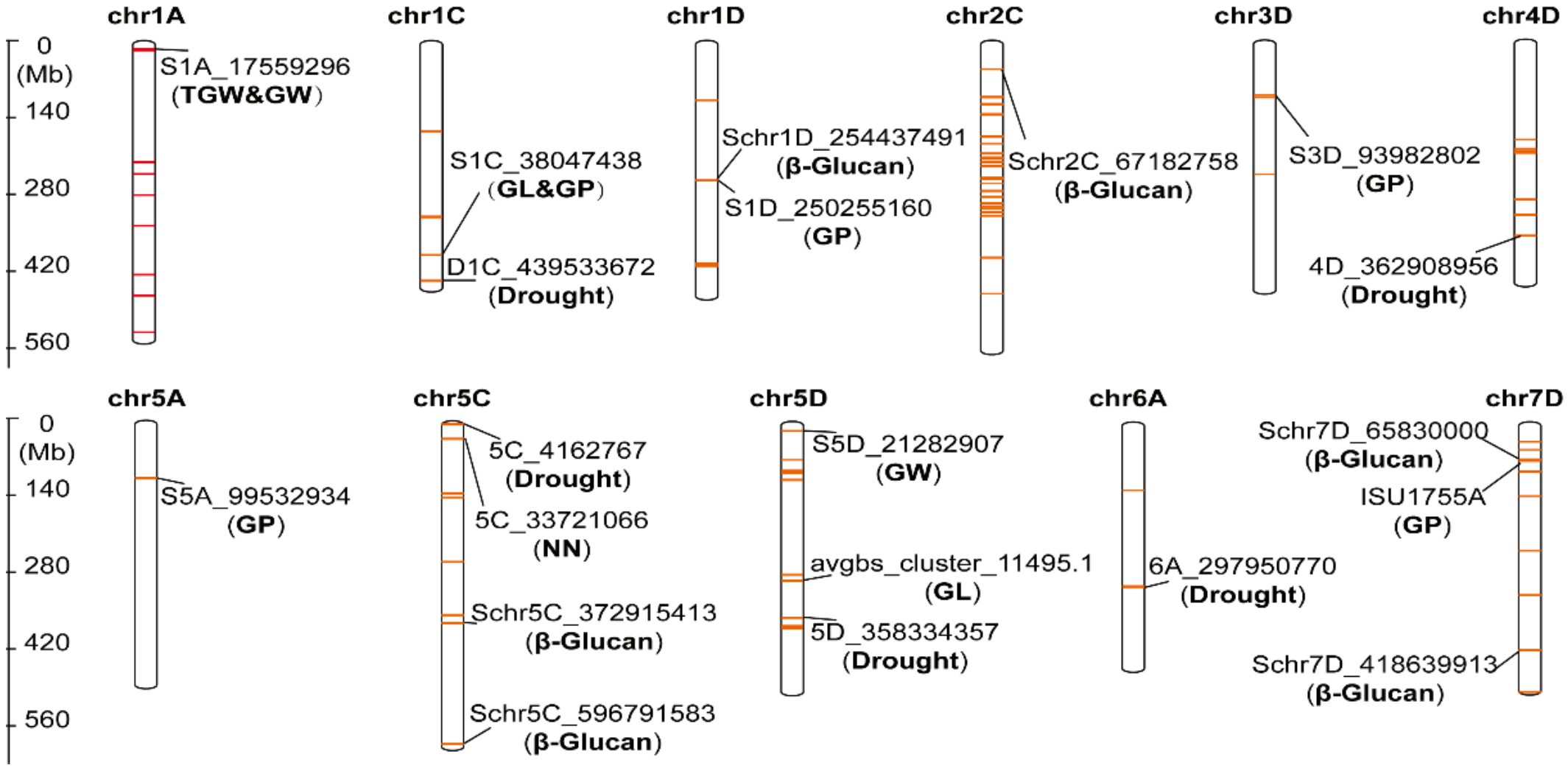

Fig. R12. Genome-wide colocalization analysis of selected gene loci and published QTLs in naked oats. (Red horizontal lines indicate the chromosomal positions of selected genes) (Revised Manuscript Fig. 4b)

2) Additionally, we incorporated 7 salt alkali and 2 drought stress response related transcriptome datasets; pathway enrichment analysis of specifically upregulated genes in hulled and naked oats showed that differentially expressed genes (DEGs) in naked oats were enriched in ion homeostasis maintenance, stress signal transduction, and secondary metabolic pathways (e.g., proline and carotenoid synthesis; GO:0006561, GO:0017157, ko99988, ko00906), whereas those in hulled oats were predominantly enriched in cell wall remodeling, terpenoid biosynthesis, and substance transport pathways mediated by ABC transporters (GO:0009834, GO:0016102, GO:0006721, ko02010; q value < 0.05). This intrinsic divergence in regulatory pathways may underpin a potential transcriptional basis for the distinct stress adaptation strategies of the two oat accessions (Fig. R13 below; also Revised Manuscrip Fig. S15). The detailed revisions are as follows: [L325-331]

"Under abiotic stress, naked oats upregulated genes involved in ion homeostasis, stress signal transduction, and specific secondary metabolism (GO:0006561, GO:0017157, ko99988, ko00906), whereas hulled oats exhibited enrichment in pathways for cell wall remodeling, terpenoid biosynthesis, and ABC transporter-mediated transport (GO:0009834, GO:0016102, GO:0006721, ko02010; *q*value < 0.05), highlighting distinct transcriptional bases underlying their differential stress adaptation strategies (Figs. S15, S16; Tab. S7)."

3) These complementary genomic and transcriptomic analyses collectively reveal inherent differences in abiotic stress response mechanisms between hulled and naked oats. It is important to note that none of these analyses are supported by direct evidence from functional validation assays a key limitation of this study. Accordingly, we have rigorously revised all relevant descriptions in the Results section, with all conclusions grounded in correlation analyses and overstatements eliminated. We have also explicitly stated the study's limitations in experimentally validating stress response mechanisms in the Discussion section to uphold the rigor of research conclusions. The detailed revisions are as follows: [L470-472]

"We acknowledge that broader developmental sampling and experimental validation are needed to fully elucidate gene functions and their regulatory networks."

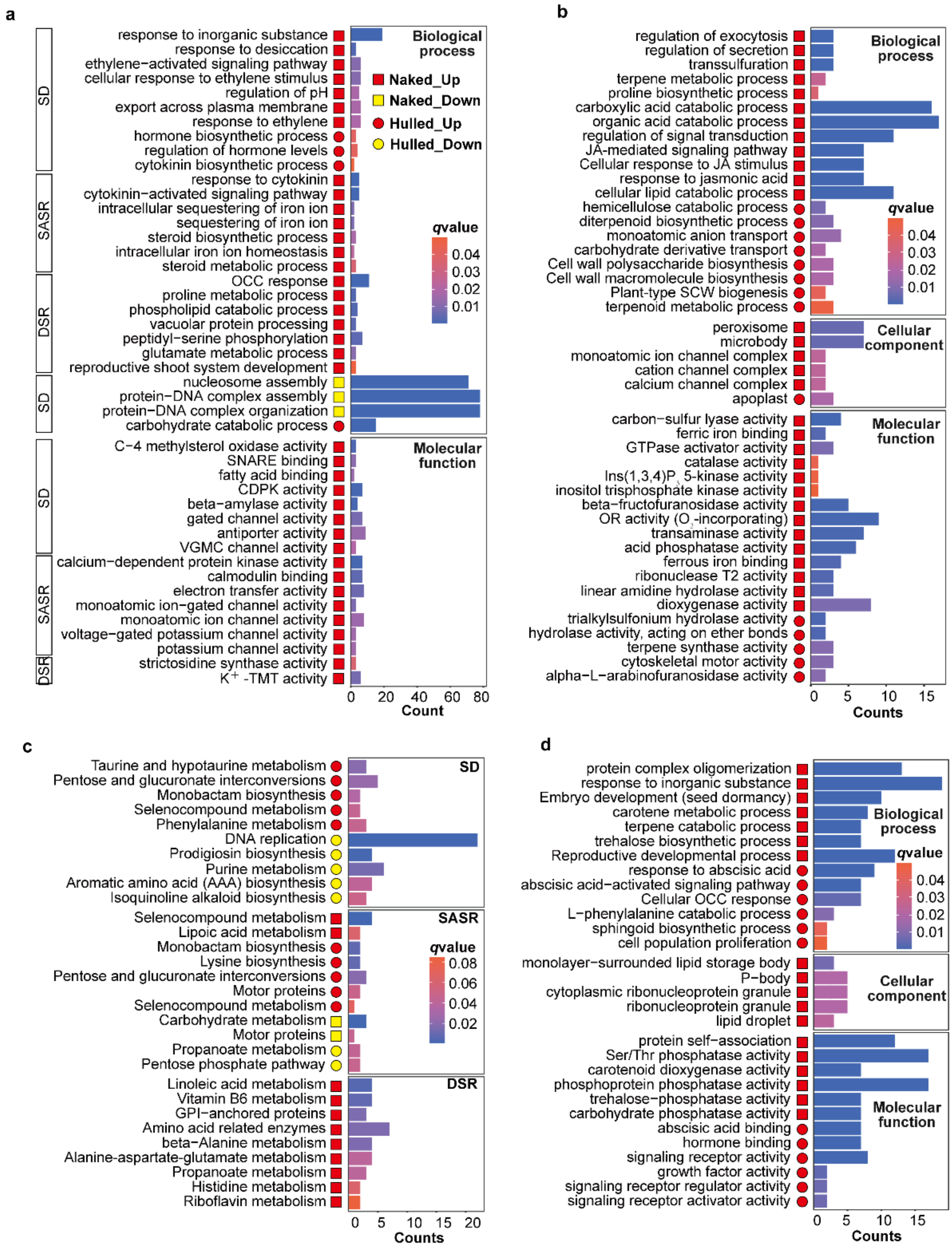

Fig. R13 Functional enrichment analysis of differentially expressed genes (DEGs) in hulled/naked oats across transcriptome analyses of basal physiology, seed development, and abiotic stress. (Abbreviations for each transcriptome project: SASR: Salt-alkali stress treatment; SD: Seed development; DSR: Drought stress treatment. Pooled multi-tissue samples refer to mixed samples of oat spikelets, leaves, and stems at the grain filling stage) (Revised Manuscrip Fig. S15). (a) Shared GO term enrichment analysis for DEGs commonly identified in pooled multi-tissue samples and condition-specific transcriptomes (SASR, SD, DSR); (b) GO term enrichment analysis for DEGs specifically up-regulated in hulled/naked oats during the seed development (SD) stage; (c) Shared KEGG pathway enrichment analysis for DEGs in pooled multi-tissue samples and condition-specific transcriptomes (SASR, SD, DSR); (d) GO term enrichment analysis for DEGs specifically up-regulated in hulled/naked oats under abiotic stress (SASR, DSR).

L180: Why did the authors not compare improvement levels or countries directly, rather than relying solely on clustering? Groups GI–GX were generated from a Neighbor-Joining (NJ) analysis based on 127,563 markers, including both neutral and non-neutral variants, and each cluster contains mixed accession types.

RESPONSE: We sincerely appreciate your insightful question. Regarding the rationale for using genetic clustering (G1-G6) for phenotypic analyses rather than directly grouping accessions by geographic origin, we explain as follows: The G1-G6 clusters used in this study were generated using the Neighbor-Joining (NJ) method with 127,563 molecular markers covering both neutral and non-neutral variations, a dataset that more objectively captures the true genetic relationships among oat accessions. Given the extensive cross-regional gene flow in global oat accessions and substantial genetic admixture between hulled and naked oats, grouping accessions solely by geographic origin would likely introduce analytical biases, including marked genetic divergence within a single geographic origin and shared genetic backgrounds across distinct geographic origins due to genetic introgression. This would hinder the accurate dissection of the independent contributions of genetic versus geographic factors to phenotypic variation. Thus, to precisely elucidate the genetic basis of phenotypic variation, we performed phenotypic divergence analyses in this study based on genetic clustering results.

What is the LD decay for the full dataset and for each population (naked vs. hulled)? How was the threshold for Fst and Pi ratio chosen in Figure 4a?

RESPONSE: We sincerely appreciate your valuable suggestions regarding the detailed specification of LD decay analysis and genome-wide selection scan methods. In light of your comments, we have added relevant descriptions in the Methods section and provided further elaboration in the Results section to more clearly demonstrate the reliability of population genetic background characterization and selection signal identification. The relevant details are presented as follows:

1）The LD half-decay distance was derived from 127,563 high-quality SNPs using r2. Analysis involved: (1) filtering SNPs (MAF ≥ 0.05, missing ≤ 0.2); (2) calculating intra-chromosomal pairwise r2 with PLINK (*American Journal of Human Genetics* 2007https://doi.org/10.1086/519795); (3) grouping r2 by distance and fitting an exponential decay model. The half-decay distance was defined as the point where r2 fell to 50% of its peak value. Based on the methods described above, we estimated and characterized linkage disequilibrium (LD) decay in hulled and naked oat populations (Fig. R14 below; also Revised Manuscript Figs. 1c and S6j).

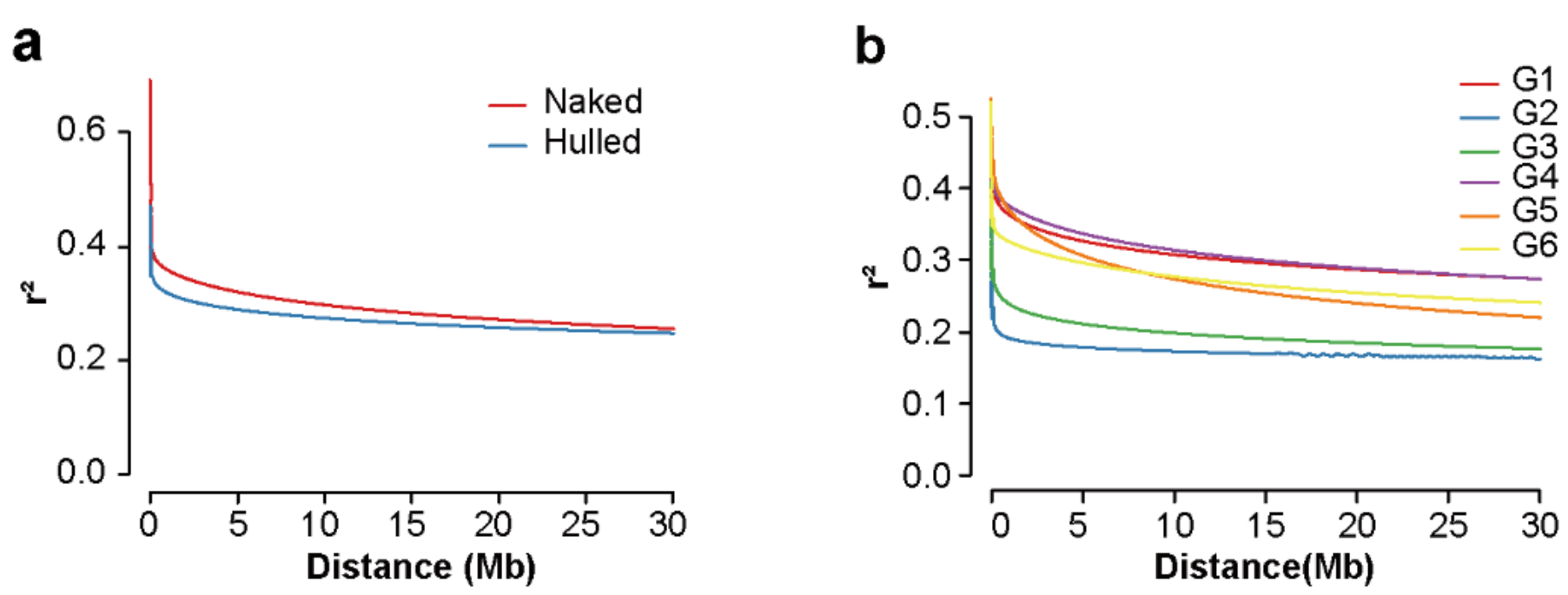

Fig. R14 Analysis of linkage disequilibrium half-decay characteristics between hulled and naked oats and each genetically clustered subpopulation (Revised Manuscrip Figs. 1c and S6j). (a) Plot of linkage disequilibrium half-decay curves for hulled oats and naked oats (Revised

Manuscript Fig. S1c). (b) Plot of linkage disequilibrium half-decay curves for 6 clustered subpopulations (Revised Manuscript Fig. S6j).

2) For the genome-wide selection scan, we used the high-quality VCF file generated for all tested accessions in this study to extract and reformat VCF subfiles corresponding to the hulled oat (504 accessions) and naked oat (162 accessions) subgroups with BCFtools (*Bioinformatics* 2016 https://doi.org/10.1093/bioinformatics/btw044). Subsequently, we employed VCFtools (*Bioinformatics* 2011 https://doi.org/10.1093/bioinformatics/btr330) to calculate nucleotide diversity (π) and fixation index (F_{st}) for the two oat genomes by applying a 500 kb sliding window with a 50 kb step size. Following these calculations, we designated genome-wide loci with a LogF_{st} value > 2.7 ($P < 0.001$) between hulled and naked oats as potential selection targets in naked oats. In addition, we computed the nucleotide diversity ratio (π ratio) at orthologous genomic loci between the two subgroups, and further identified the top 1% of these loci ($\pi_{hulled}/\pi_{naked} > 59.07$) as potential selection targets in naked oats via the percentile method (*Nature genetics* 2012 https://doi.org/10.1038/ng.2309; *The Plant cell* 2023 https://doi.org/10.1093/plcell/koad229).

4. Genome-Wide Scanning and RNA-seq

L187: Why did the authors perform a genome-wide selective sweep analysis rather than a genome-wide association study (GWAS)? Selection scans are generally more informative in advanced populations after several selection cycles where Fst changes within specific genomic regions.

RESPONSE: We sincerely appreciate your valuable suggestions. Our study prioritized genome-wide selective sweep analysis due to its strong alignment with our core research objectives: we focused on genomic selection divergence between hulled and naked oats during domestication and genetic improvement, a method that directly detects candidate selected genomic regions shaped by long-term directional selection and accurately captures signals of marked allele frequency shifts driven by selection during the divergence of these two oat groups, thus aligning perfectly with our core scientific inquiry into improvement-driven genomic divergence.

Your insightful suggestions also highlighted the untapped value of genome-wide association studies (GWAS) for this research. To validate the biological relevance of candidate regions identified via selective sweep analysis, we accordingly integrated published oat phenotype-associated QTL datasets and conducted colocalization analysis and cross-validation of these candidates selected regions with GWAS-mapped trait-associated loci (Fig. R12 above; also Revised Manuscript Fig. 4b). This combined analysis not only elucidates patterns of selected genomic divergence between hulled and naked oats during genetic improvement but also clarifies their potential links to divergence in key agronomic traits (e.g., yield and quality), thereby establishing a robust link between selection signals and phenotypic effects. The revised findings are presented below: [L283-294]

"To investigate the association between selection signals and phenotypic differentiation, we colocalized selected genes in naked oats with published QTLs. We identified selected genes associated with 20 known QTLs across 11 chromosomes, and their selection patterns were highly consistent with phenotypic differentiation between

hulled and naked oats. By trait category, only 11 of 51 genes linked to yield-related QTLs (e.g., thousand-kernel weight, grain size) showed directional selection. In contrast, 38 of 50 genes associated with crude protein content and 86 of 113 genes for β-glucan content exhibited signatures of directional selection (Fig. 4b; Tab. S6-2), indicating stronger selection on quality-related genes during naked oat domestication. Moreover, 117 of 178 genes linked to 5 drought-responsive QTLs showed directional selection (Fig. 4b; Tab. S6-6), implying enhanced adaptation to specific ecological niches."

L227: The RNA-seq analysis of 22 genetically and geographically diverse accessions is interesting, but why were tissues (root, stem, leaf, spike) pooled across different developmental stages? Using pooled tissues increases background noise and makes it difficult to identify differentially expressed genes (DEGs). The 22 accessions could have been sequenced separately with replicates instead. If the goal was to identify DEGs in spikes and relate them to oat type, only spike tissue should have been used.

RESPONSE: We sincerely appreciate your constructive comments; your insights on the transcriptome sequencing strategy offer critical guidance in refining our experimental design and enhancing the reliability of our findings. We selected 9 hulled oat and 13 naked oat accessions for analysis, encompassing representative germplasm with diverse genetic backgrounds for both types and thereby providing a sound basis for transcriptional divergence analysis. Mixed-tissue RNA-seq of roots, stems, leaves, and panicles was a practical decision driven by sequencing cost constraints, intended to efficiently capture shared expression differences across tissues, rapidly generate a broad transcriptome profile across developmental stages, and initially identify core differential trends in global transcription between the two oat types.

We fully acknowledge and agree that mixed-tissue sampling introduces background noise and compromises the accurate identification of differentially expressed genes (DEGs). To mitigate this limitation to the greatest extent possible, we integrated public targeted transcriptome datasets, including 7 seed development, 7 salt-alkali stress, and 2 drought response datasets in oats (Revised Supplementary Tab. S7-2). Combined differential expression analysis across these datasets effectively improved the reliability of core gene identification while preserving the study's exploratory breadth. In the revised manuscript, we have placed greater analytical emphasis on genes associated with seed development and salt-alkali stress responses, and clearly elaborated on the study's limitations in the Discussion section (Fig. R13 above; also Revised Manuscrip Fig. S15) . The detailed revisions are as follows: [L308-331]

"To investigate transcriptional regulatory divergence between hulled and naked oats, we conducted RNA-seq analysis on pooled root, stem, leaf, and spike tissues from 13 naked oat and 9 hulled oat accessions with geographically and genetically diverse backgrounds, and further integrated 16 public transcriptome datasets (7 for seed development, 9 for salinity and drought stress) for comprehensive multi-dimensional comparisons (Tab. S7-2). Enrichment analysis of differentially expressed genes (DEGs) during seed development revealed that upregulated genes in naked oats were primarily associated with pathways related to nutritional quality accumulation, including developmental signaling, sterol-lipid synthesis, and ion channel activity (e.g.,

GO:0009873, GO:0009867, GO:0000149, ko00906, ko00543; *q*value < 0.05).This suggests their transcriptional regulation tends to favor nutrient synthesis and accumulation in the grain. In contrast, upregulated genes in hulled oats during seed development were primarily enriched in pathways for cell wall organization, transmembrane transport, and basal metabolism (e.g., GO:0009834, GO:0070592, ko02010, ko00430; *q*value < 0.05), potentially linking to nutrient transport efficiency and growth regulation. Under abiotic stress, naked oats upregulated genes involved in ion homeostasis, stress signal transduction, and specific secondary metabolism (GO:0006561, GO:0017157, ko99988, ko00906). Under abiotic stress, naked oats upregulated genes involved in ion homeostasis, stress signal transduction, and specific secondary metabolism (GO:0006561, GO:0017157, ko99988, ko00906), whereas hulled oats exhibited enrichment in pathways for cell wall remodeling, terpenoid biosynthesis, and ABC transporter-mediated transport (GO:0009834, GO:0016102, GO:0006721, ko02010; *q*value < 0.05), highlighting distinct transcriptional bases underlying their differential stress adaptation strategies (Figs. S15, S16; Tab. S7)."

L247: It is unclear how the transcriptomic findings align with the genome-wide selection scans. How many associated markers were overlaping with DEG? and how to correlate the role of the 19 traits with the Genome-Wide Scanning and RNA-seq analysis?

RESPONSE: We sincerely appreciate your insightful comment regarding the need to strengthen the association between genomic changes and phenotypic divergence in naked oats during genetic improvement, which has greatly helped enhance the manuscript's overall logical coherence and the study's scientific merit. In the revised manuscript, we performed an overlap analysis by integrating selected genes from genome-wide scans with our transcriptome data, and we additionally incorporated published oat QTL datasets into this analysis. Through this combined analytical approach, we identified a core gene set and derived systematic conclusions that unify selection signals, transcriptional expression profiles, and phenotypic associations, thereby reinforcing the key association between genomic variation and phenotypic divergence (Fig. R15 below; also Revised Manuscript Fig. 5). The detailed revisions are as follows: [L332-355]

"To identify key genes underlying hulled-naked oat differentiation, we integrated 1,504 candidate selected genes in naked oats with multi-context transcriptome data to construct a combined selection signal-expression association profile. Our results revealed that during core physiological processes, we identified 39 selected genes exhibiting differential expression between hulled and naked oats, comprising 31 upregulated and 8 downregulated genes (e.g., *UMAMIT25*, *AHL1*, *SAG12*, *ygl8*, *WOX13*). Among these, 22 genes under directional selection and upregulated expression were significantly enriched in pathways such as lipid metabolism and post-translational protein modification (Fig. 5a; Tab. S7-6). During the seed development stage, a total of 31 selected genes were identified. Among them, upregulated genes (e.g., *PARP1*, *RAB28*, *PP2C30*, *HSP18.2*) may be involved in embryonic cell division and differentiation and ABA signal response, whereas genes under balancing selection and downregulated expression (e.g., *MPK3*, *CycB1;5*, *RSW3*) were enriched in cell cycle regulation pathways (*q*value < 0.05) (Fig. 5b; Tab. S7-6). In contrast, the 32 differentially expressed genes related to seed development in hulled oats exhibited a

pattern of cooperatively upregulating energy metabolism and signal transduction while downregulating redundant hormone pathways (Fig. 5c; Tab. S7-6). Furthermore, in response to abiotic stress, 25 selected genes specifically expressed in naked oats (e.g., *PP2C09*, *FEI1*, *PME18*, *PIP3I*) generally displayed signatures of directional selection and upregulated expression, suggesting their potential functions in stress adaptation (Fig. 5c-f; Tab. S7-6). Further analysis revealed that 33 of the aforementioned 127 associated genes co-localized with published QTLs for oat yield and quality traits (Fig. 5; Tab. S7-5, highlighted in red), significantly enhancing the credibility of these candidate genes in trait differentiation."

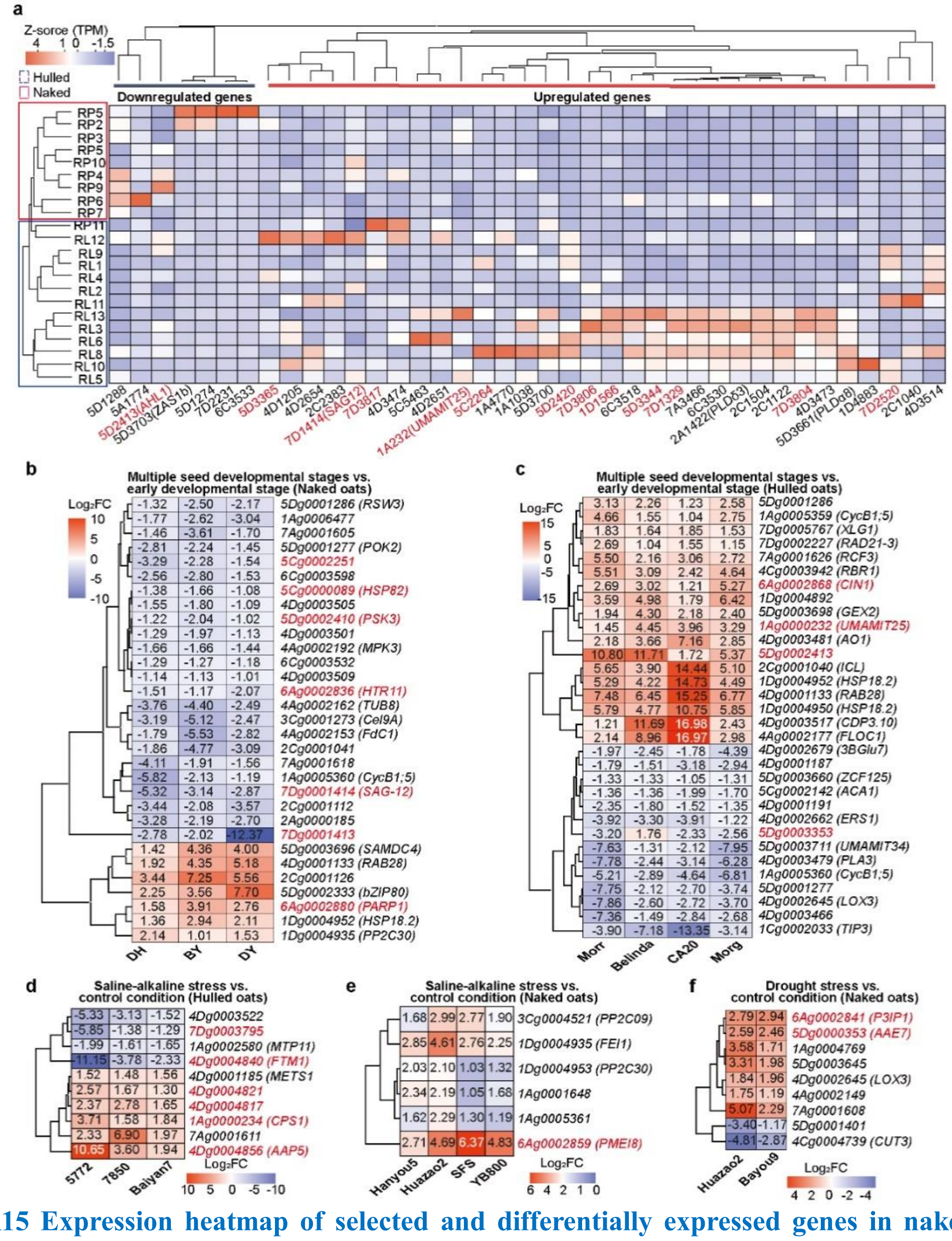

Fig. R15 Expression heatmap of selected and differentially expressed genes in naked oats based on Log_2FC values (Genes marked in red are associated with previously published oat QTLs) (Revised Manuscrip Fig. S5). (a) Intersection of DEGs between hulled and naked oats from bulk RNA-seq and selected genes in naked oats; (b) Intersection of DEGs from seed development transcriptome of naked oats and selected genes in naked oats; (c) Intersection of DEGs from seed development transcriptome of hulled oats and selected genes in naked oats; (d) Intersection of DEGs from saline-alkali stress transcriptome of naked oats and selected genes in naked oats; (e) Intersection of DEGs from drought stress transcriptome of naked oats and selected genes in naked oats.

L270: The rationale for using both KASP markers and qPCR is unclear. Typically, qPCR is used to validate DEGs.

RESPONSE: We sincerely apologize for the unclear wording in the original manuscript, which may have hindered your reading comprehension. In this study, KASP markers and qPCR were mainly used to verify the reliability of the associations between candidate genes and oat grain yield/quality-related traits, as well as their breeding potential. These two methods constitute a progressive and complementary validation strategy: we speculate that changes in the expression level of the gene may lead to changes in its function. Therefore, qPCR was mainly used to verify the expression specificity of candidate genes in panicles at the grain filling stage and the expression differences between hulled oats and naked oats. Furthermore, we developed KASP markers for functional SNPs in the upstream region of *UMAMIT25* and validated them in a population consisting of 268 hexaploid oat accessions. This validation not only confirmed the genotyping accuracy of haplotypes associated with grain size and crude protein content, but also developed these markers into practical tools for marker-assisted selection (MAS). These two assays were used as follow-up validations for the key genes identified in the preliminary study through a combination of selective sweep, GWAS-QTL association, transcriptome differential expression, and haplotype analysis. Collectively, these results strongly support the regulatory significance and practical breeding potential of the candidate genes.

Minor Comments

L100: Replace “Ancestral component analysis” with “ADMIXTURE analysis.”
RESPONSE: We sincerely appreciate your revision suggestions. We have revised the term "Ancestral component analysis" to "ADMIXTURE analysis" in the revised manuscript, which makes the nomenclature more aligned with our study results and avoids overstatement.

Fig. 1 L664: “Ancestral component” refers to a structure analysis with multiple K. Include sample origin in the PCA (e.g., AFR for Africa). Please maintain consistent geographic abbreviations across figures—e.g., Fig. S5 uses “CHN” for China and “AF” for Africa, but other figures use different labels.

RESPONSE: We sincerely thank you for your revision suggestions on the aforementioned issues in our manuscript! Your valuable input has greatly enhanced the standardization and readability of the figures and tables, and we have fully implemented all revisions in strict accordance with your requirements:

1) In the population structure analysis module, we reconstructed the NJ phylogenetic tree using hexaploid wild oats as the outgroup, and partitioned the entire germplasm panel into six subpopulations based on Admixture analysis results (K=1-20). For data visualization, we integrated Admixture results at K=6 with the NJ phylogenetic tree for combined visualization, and annotated the key geographic information for germplasm accessions in each subpopulation (Fig. R16 below; also Revised Manuscript Fig. 1b). Furthermore, we explicitly annotated the geographic origins of all samples on the principal component analysis (PCA) plot (Fig. R4 above; also Revised Manuscript Fig. 1c).

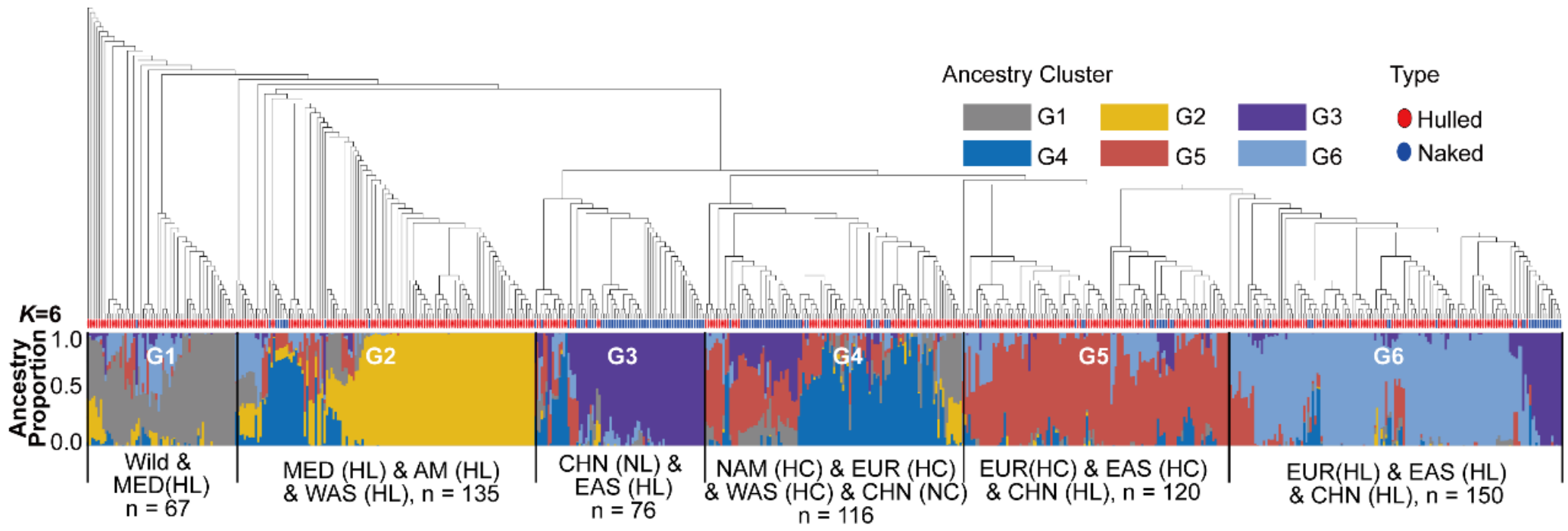

Fig. R16 Genetic analysis of 666 oat accessions using 127,563 high-quality SNP markers, presenting the rooted neighbor-joining tree and population structure Admixture plot at *K*=6 (Revised Manuscript Fig. 1b).

2) The abbreviations for geographic origins have been standardized across all panels: Western Asia (WAS), South America (SAM), North America (NAM), Mediterranean Region (MED), Eastern Europe (EEU), East Asia (EAS), China (CHN), Australia (AUS) and Africa (AFR).

Panels (c) and (d) could be replotted using an interactive 3D Plotly visualization. Panel (f) (Circos plot) could be replaced with a table or matrix for easier comparison.

RESPONSE: We greatly appreciate your valuable suggestions to enhance the readability of the figures in our manuscript. We have redrawn Figs. 1c and 1d as interactive 3D Plotly plots (Fig. R4 above; also Revised Manuscript Fig. 1c), and replaced the circular plot of Fig. 1f with a more intuitive and highly readable matrix heatmap (Fig. R3 above; also Revised Manuscript Figs. 1h-j).

L686: "Spike type (ST)" and "Spikelet type (SLT)" are mislabeled in Fig. 3b. The figure also lacks axis labels.

RESPONSE: We thank you for your valuable comments. We have comprehensively revised Fig. 3b as follows: Firstly, we corrected and standardized the labels for panicle type (ST) and spikelet type (SLT). Secondly, we optimized the original figure to a stacked plot to more clearly illustrate the distribution differences in panicle type, spikelet type, and awn traits between hulled and naked oats. Furthermore, we added complete x- and y-axis labels to ensure the figure information is accurate and readily interpretable (Fig. R10 above; also Revised Manuscript Fig. 3b).

L161: The morphological differences between hulled and naked oats (e.g., multiflowered spikelets and long awns) are not clearly visible in Fig. 3b.

RESPONSE: We greatly appreciate your meticulous comments. We have rephotographed and prepared a high-resolution comparative figure of panicle morphology between hulled and naked oats (Fig. R1 above; also Revised Manuscript Fig. 3a), and added textual labels to the spikelets and lemmas with significant morphological differences between the two oat types for a more intuitive visual comparison for readers.

Reviewer #2 (Remarks to the Author):

In this manuscript, Dong and colleagues systematically integrated population genetics, multi-environment phenotypes, selection and clearance scanning, transcriptomics and functional verification, and comprehensively analyzed the domestication pathway, yield-nutrient trade-off mechanism and key candidate genes of naked oat. The scale of the germplasm (666 accessions) and phenotyping (19 traits) is impressive. Despite the claim to have analyzed 666 "globally sourced" oat accessions, 90% of the naked oat samples (146 out of 162) originate from China, while critical regions implicated in the origin debate, such as Central Asia, Eastern Europe, Turkey, and Russia, are severely underrepresented. This 'pseudo-global' sampling strategy effectively only validates the genetic background of Chinese naked oats, and fails to rule out the possibility of independent domestication events occurring elsewhere. The paper overstates its causal claims, lacks mechanistic validation, and fails to address alternative evolutionary scenarios. I recommend major revision with a sharper focus on correlation rather than causation, and a much more cautious interpretation of domestication history. These issues need appropriate revisions and clarifications before this work can be endorsed for publication.

RESPONSE: We thank the reviewer for the thorough and insightful comments. We fully agree with the critiques regarding the geographic limitations of our germplasm sampling, the overstated conclusions, the insufficient discussion of evolutionary hypotheses, and the lack of mechanistic validation. These points are invaluable for improving our manuscript. Regarding the concern about a "pseudo-global" sampling design, we would like to clarify the following based on our actual materials: first, no published studies to date have reported the presence of naked oat landraces outside China and Mongolia, and our 146 Chinese naked oat accessions were collected from 14 provinces, representing substantial geographic diversity within China, with their concentrated distribution reflecting the natural distribution of naked oat landraces; second, of the 666 accessions included in this study, 504 are hulled oat, 36.7% (185 accessions) of which originated from Central Asia, Eastern Europe, Türkiye, and Russia-key regions relevant to the origin of naked oat-and 74% (137 accessions) of these are landraces with relatively ancient genetic backgrounds. Given this context, we believe our overall germplasm panel retains reasonable global representativeness. We fully take your comments seriously and will revise the manuscript rigorously as recommended: we will strengthen the discussion of germplasm background and clearly document the geographic distribution of all accessions, adjust the framing of our conclusions to focus on correlational analyses and interpret the domestication history of naked oat more cautiously, supplement discussion of alternative evolutionary hypotheses, and enhance cross-validation and integrated analyses to address remaining limitations. We will refine the study with strict scientific objectivity to improve its reliability and robustness.

Major Comment:

1. Lines 77-80: The Introduction acknowledge that oats are allohexaploid, and that structural variations (SVs), such as the chr7D inversion (Bekele et al. 2024) or chr1A-chr1C translocations (Li et al. 2025), have been shown to drive domestication-related traits, and independent domestication hypothesis based on chr7D inversion, while this study derived Mediterranean hulled oat origins using solely SNP data. The authors

should explicitly emphasize the differences and complementary nature between these two methodological approaches.

RESPONSE: We thank you for the constructive suggestions on interpreting oat domestication history in relation to prior studies. Bekele et al. (*Nature communications* 2025 https://doi.org/10.1038/s41467-025-57895-3) and Li et al. (*Cell reports* 2025 https://doi.org/10.1093/gigascience/giad061) emphasized chromosomal structural variations, such as the 7D inversion, to propose independent domestication events. Our study instead employed genome-wide SNP analysis. While this approach does not capture structural variants, it provides high genome coverage, precise estimates of genetic distance and relatedness, and scalability for large and diverse panels-features well suited for tracing dispersal routes and resolving hierarchical population structure.

To address the limitations of a single analytical method, we performed cross-validation using complementary approaches, including population structure inference, gene flow detection, F_{st} and D_{XY} estimation, and demographic modeling with fastsimcoal2. This integrated framework consistently supports our conclusions (Fig. R1 below; also Revised Manuscript Fig. 2). We further evaluated the 7D inversion hypothesis by reanalyzing the NC_GBS data (*Nature communications* 2025 https://doi.org/10.1038/s41467-025-57895-3) using a 31-mer SNP based haplotype approach across 591 accessions. Chinese naked oats predominantly carried H1-H3 haplotypes, with H3 being the most frequent (55%). This pattern closely aligns with the dominant haplotype in Eurasian hulled oats, suggesting that the main genetic component of Chinese naked oats derives from the major hulled domestication lineage (the P05 ancestor marked by H3). The H4 haplotype, though present in a few Chinese naked oat accessions, was found in only one Turkish hulled landrace (1/23). Its highly uneven distribution between populations is inconsistent with a common independent origin but fits a founder effect model. Together, the genome-wide SNP evidence—population structure, gene flow patterns, differentiation metrics, demographic modeling, and targeted 7D haplotype analysis-converges on an evolutionary scenario of Mediterranean origin, eastward dispersal, and subsequent redomestication in China(Fig. R2 below; also Revised Manuscript Fig. S7). These revisions have been incorporated into the Results (L159-211) and Discussion (L411-443). The detailed revisions are as follows:

1) L159-211: "To minimize confounding gene flow from modern breeding, we selected 366 landraces with conserved ancient genetic backgrounds for gene flow and population evolutionary analyses. First, phylogenetic reconstruction of 366 landraces reveals hulled oats dominate transcontinental clusters with continuous distribution, comprising the Northeast African and West Asian group (GI: 45 accessions), the Mediterranean Basin group (GII: 77 accessions), and the Central and Eastern Eurasian group (GIII: 110 accessions). In contrast, over 81% of Chinese naked oats segregate into isolated clades GIV. The GV subpopulation consists of 63 admixed accessions of hulled and naked oats from multiple geographic origins (Figs. 2a-c and S8a, b; Tab. S4). Notably, although most Chinese naked oats formed a single subpopulation, 26.8% of accessions showed genetic admixture with other subpopulations and geographically diverse hulled oats (Tab. S4). Further gene flow analysis revealed 7.5%-16.8% shared haplotypes across subpopulations and significant multidirectional introgression in most pairs (D > 0.45, Z > 9, P < 0.001), indicating sustained cross-regional gene flow between hulled and naked oat landraces even after excluding cultivars, and supporting an ancient evolutionary origin for their genetic exchange (Fig. 2g-j; Tab. S4,). Gene flow analyses also delineate the evolutionary succession: first from wild hexaploid oats

to hulled oat landraces, followed by naked oat landraces from hulled oat landrace and finally hulled and naked cultivars derived from their respective landraces (Fig. S6e), Therefore, the genetic admixture between hulled and naked oats is the result of both shared ancestral polymorphism and ancient gene flow, rather than being a product of recent breeding alone.

Genetic differentiation analysis identifies the Mediterranean Basin group (GII) as the most divergent (F_{st} = 0.18-0.31) and genetically diverse ($\pi = 2.75 \times 10^{-6}$) subpopulation, suggesting it is an ancestral diversity center retaining ancient landrace variation (Fig. 2d-e; Tab. S4-2). In contrast, extensive gene flow (D = 0.45, Z = 9.3, $P < 0.001$), minimal differentiation (F_{st} = 0.09-0.12), and the highest proportion of shared long ROH (1-3 Mb; 44.7%) occur between the Central and Eastern Eurasian group (GIII) and Chinese naked oats (GIV), positioning GIII as the most direct genetic source for GIV (Fig. 2f-h; Tab. S4-2). Phylogenetic analysis based on D_{xy} quantifies these relationships: GIV shows the smallest D_{xy} with GIII (0.231), confirming their close affinity, whereas GII exhibits the largest D_{xy} with all other groups (0.279-0.305), consistent with its early divergence (Fig. 2i). Demographic modeling with fastsimcoal2 further supported these evolutionary relationships. The no-migration model was rejected across all pairwise comparisons among GII, GIII, and GIV, verifying extensive post-divergence gene flow. The optimal three-population model (Model 9, AIC = 293574.92) identified GIV as a recent derivative of the GIII lineage. It diverged from the GIII ancestral pool ~6124 generations ago and initiated secondary population contact ~4940 generations ago, after an early isolation period of 1184 generations. During early divergence, GIV experienced an extreme founder bottleneck (Ne ≈ 187), with strong genetic drift accelerating the fixation of unique traits. GIV initiated recent population expansion at 1772 generations ago, earlier than GIII (1572 generations ago), consistent with the rapid adaptation and diffusion typical of derived lineages post-domestication. Furthermore, GII's *Ne* (969) was markedly lower than the model-predicted value (1,860), while GIII's *Ne* (3,625) exceeded expectations (1,572). GIV's *Ne* (1,338) closely matched the predicted value (1,772). These patterns collectively support GII as the ancestral lineage and GIII as the most direct genetic source of GIV(Fig. 2j; Tab. S4-3). Collectively, synthesizing population structure and diversity patterns, we propose the primary domestication is hulled oats from wild species in the Mediterranean Basin region. The dual dispersal are southeastward to East Africa and Eastward to Central and Eastern Eurasia/China. During this eastward dispersal, naked oats diverged from hulled oat lineages, followed by secondary domestication in North China (highest π in naked oats) (Fig. S8c-d)."

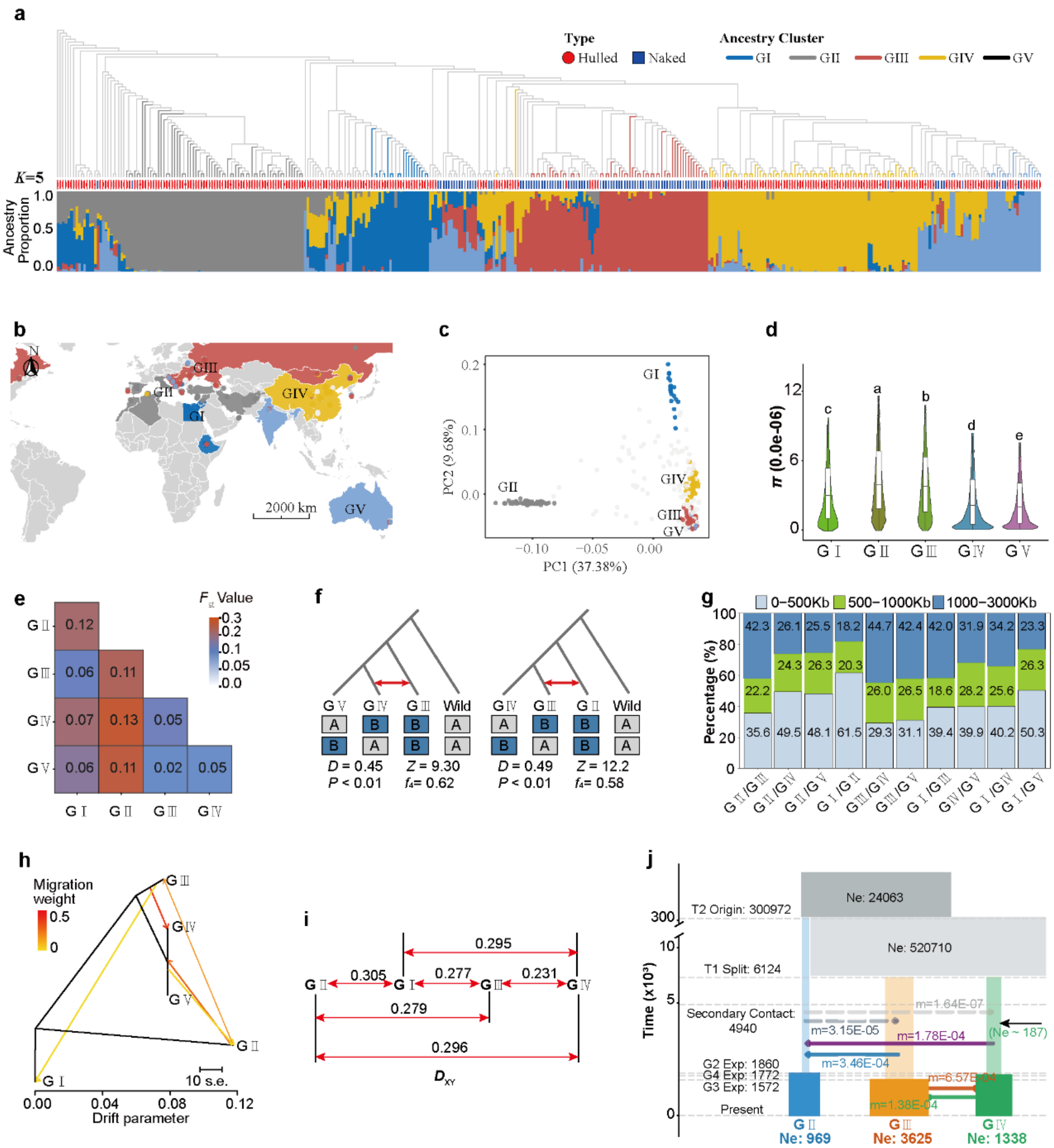

Fig. R1 Analysis of the domestication pathway of naked oats based on 366 landrace accessions (Revised Manuscript Fig. 2). (a) Combined plot of Admixture analysis (K = 5) and neighbor-joining (NJ) phylogenetic tree (rooted with wild accessions) for 366 landrace accessions; (b) Global distribution of accessions in each subgroup; (c) Population clustering of 366 landrace accessions based on the first two principal components (PC1 and PC2); (d) Nucleotide diversity levels among different subgroups (P < 0.05); (e) Fixation index (F_{st}) values among different subgroups; (f) ABBA-BABA test revealing significant genetic flow between GII and GIII-GIV, and between GIII and GIV-GV subgroups; (g) Proportions of shared homozygous haplotypes of different lengths (10 kb–3 Mb) among subgroups; (h) Phylogenetic topology and genetic flow direction among clustered subgroups with five fitted migration events; (i) D_{xy}-based phylogenetic tree showing genetic diversity differentiation among GI-GIV subgroups; (j) Demographic history reconstruction of GII, GIII, and GIV using fastsimcoal2.

2) L416-449: "Furthermore, integrated analysis of population structure, gene flow, and genetic differentiation reveals that naked oats originated from a key divergence during the eastward dispersal of Mediterranean hulled oat landraces, undergoing

independent domestication and diversification in China. This conclusion supported by multiple genetic lines of evidence from globally representative samples. First, 26.8% of Chinese naked oat landraces show admixture with Eurasian hulled oats (Fig. 1a, 2a; Tab. S4), and ancient gene flow plus long shared haplotypes between GIV and hulled subpopulations (Fig. 2g-i; Tab. S4) indicate non-isolated differentiation. Second, GII (Mediterranean hulled oats) is the ancestral lineage with highest diversity (Fig. 2d, e). Significant gene flow occurs from GII to GIII (Central/Eastern Eurasian hulled) and then to GIV (Chinese naked) (Fig. 2b, i; Tab. S4-3), with D_{XY} and fastsimcoal2 confirming closest GIII-GIV affinity (Fig. 2f; Tab. S4-3). These findings delineate a continuous differentiation gradient from the Mediterranean to Central/Eastern Eurasia and then to Chinese naked oats (Fig. 2f). These findings align with Loskutov's which posits that naked oats arose from mutations in cultivated hulled oats and were later domesticated in China[25], but contrast with Nan et al.[26] which suggested Chinese naked oats may have diverged from wild hulled oats prior to domestication, followed by independent domestication. Bekele et al.[27] inferred from 7D-H4 haplotype distribution that Turkish hulled oats and Chinese naked oat landraces likely originated independently from weedy species such as *A. fatua*. Fine-scale chr7D haplotype analysis with NC_GBS data [27] supports naked oat evolution in the present study. First, the 7D-H3 haplotype dominates Chinese naked oats (55%), matching Eurasian hulled oat patterns [27], indicating derivation from the mainstream hulled lineage characterized by the 7D-H3 haplotype. Second, the 7D-H4 haplotype, despite high frequency in North China naked landraces (30%), appears in only 1 in 23 Turkish hulled landraces, arguing against common independent origin. Furthermore, 7D-H4 accessions show coordinated phenotypes: tall (152.23 cm), late heading (~10 d), increased protein (19.6%) and fat (6%) (Fig. S7; Tabs. S2-2, S4-1, S4-5), this combination of "tall stature, late maturity, and high nutritional quality" aligns with the requirements of traditional grain-forage dual-purpose systems in Northern China. Additionally, selection signals in the chr7D inversion (256.6-412.1 Mbp) in naked oats (Fig. 4a,b; Tab. S6-2) suggest local adaptation roles. Notably, naked grain loci reside on 4D [14,15,21,26]. Thus, the chr7D inversion is more likely associates with adaptive traits, not the naked phenotype directly."

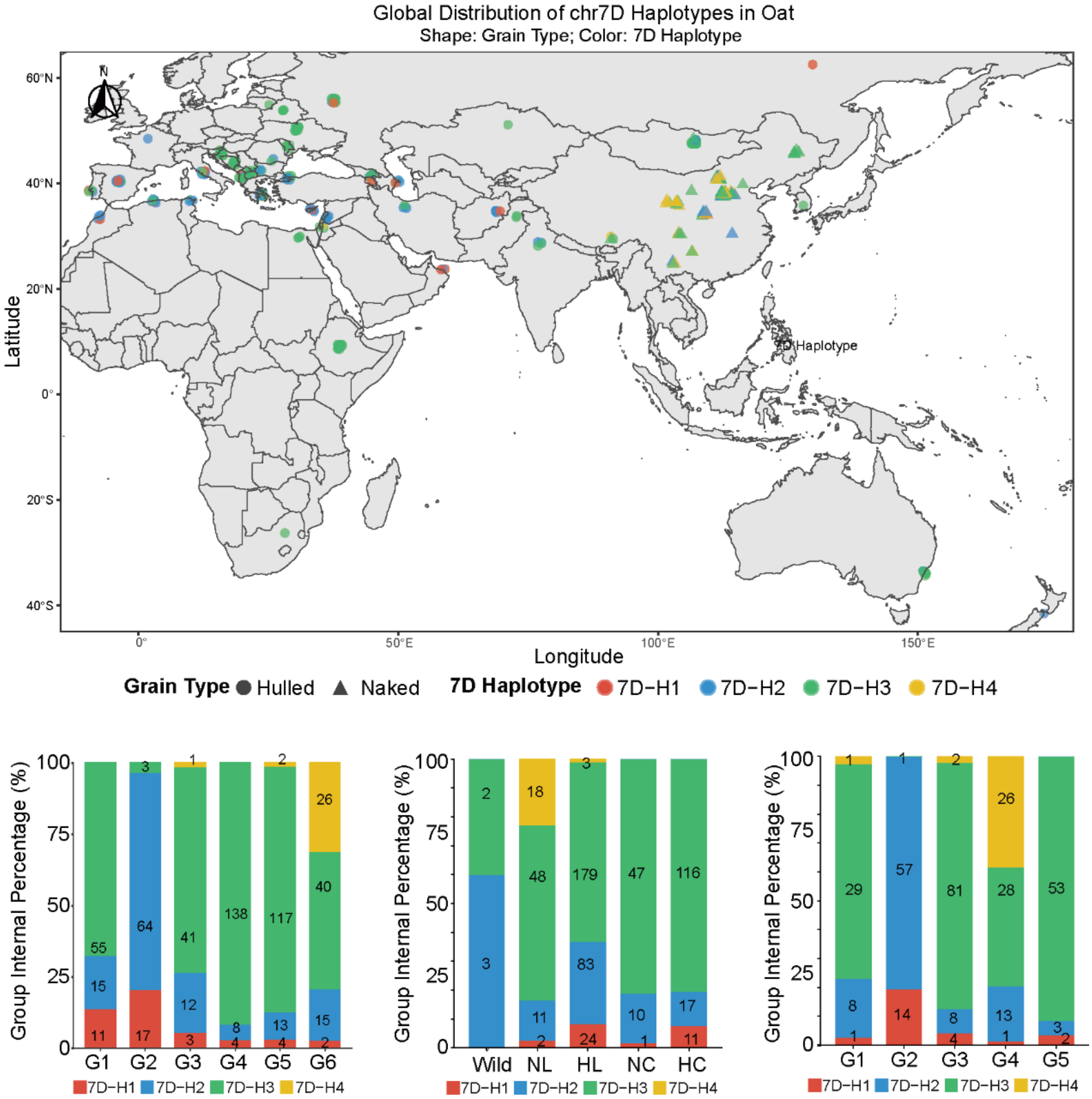

Fig. R2 Validation and characterization of known chr7D inversion haplotypes in the present study (Revised Manuscript Fig. S7). (a) Distribution of 7D inversion haplotypes among the 591 analyzed accessions; (b) Proportions of the four haplotypes across the six genetic clusters identified in the entire panel; (c) Haplotype composition in hulled versus naked oat accessions; (d) Haplotype proportions within the five genetic clusters defined among landrace accessions.

2. Lines 103-104: The 'Fst = 0.0701' indicates 'weak differentiation', but fails to justify this threshold in the context of an allohexaploid crop. In polyploids, Fst values are often lower than in diploids — 0.07 may represent moderate or even strong differentiation. The authors must compare this value with published Fst distributions in oat or other polyploids (e.g., Li et al. 2025) and explicitly define why 0.07 is classified as 'weak'.

RESPONSE: We thank you for pointing out this issue. Previously, we did not elaborate on justifying the F_{st} threshold in the context of allopolyploid crop characteristics, and we now provide supplementary evidence to clearly clarify this rationale: in this study, F_{st} = 0.0701 is defined as indicating "weak differentiation", based on the findings of Nan et al. (*Gigascience* 2022 https://doi.org/10.1093/gigascience/giad061) from their study on hexaploid oat populations. This study clearly classified genetic differentiation levels in hexaploid oats as follows: weak differentiation for $F_{st} < 0.08$, moderate differentiation for $0.08 \leq F_{st} \leq 0.15$, and significant differentiation for $F_{st} > 0.15$. This

criterion is also consistent with that reported in studies on allopolyploid wheat (*Plant Biotechnology Journa* 2022 https://doi.org/10.1186/s12864-020-6536-x; *Nature Communications* 2022 https://doi.org/10.1038/s41467-022-28453-y). Therefore, a value of 0.0701 is consistent with the characteristics of weak differentiation in hexaploid oats.

3.Line 123-126: The authors claim that "The 666 accessions displayed a reticulate structure, maintained by 18 inter-regional and 22 inter-subgroup gene flow events" based primarily on TreeMix analysis. While TreeMix can suggest historical gene flow, relying solely on this method provides limited evidence for a complex reticulate population structure. The manuscript does not provide independent validation of these events (e.g., f-branch statistics, D-statistics, or haplotype-based tests), and the connection between the numerical counts (18 inter-regional, 22 inter-subgroup) and the visualization in Fig. S4g, h is unclear.

RESPONSE: We thank you for pointing out that our sole reliance on TreeMix analysis previously was insufficient to fully support the conclusion that the 666 accessions exhibit a complex reticulate population structure and inter-subpopulation gene flow events. We attach great importance to this comment and have performed comprehensive supplementary validation analyses.

To determine the optimal number of clusters, we first performed Admixture analysis with cross-validation for K values ranging from 1 to 20. Integrating Admixture, PCA, and a neighbor-joining phylogenetic tree rooted with wild hexaploid oat consistently supported the classification of 6 subpopulations (G1-G6) (Fig. R3 below; also Revised Manuscript Fig. S3). Hulled oat accessions from the Mediterranean and West Asia emerged as a genetic hub, showing admixture across all subpopulations and extensive genetic exchange between hulled and naked oats as well as across geographic regions, indicating a reticulate population structure. We further examined haplotype sharing among the six subpopulations (Fig. R4 below; also Revised Manuscript Fig. S4). Subpopulations, particularly those from adjacent regions, shared more homozygous haplotypes (10-3000 kb) and exhibited a higher proportion of long shared segments (>1000 kb), directly reflecting frequent genetic exchange. To quantify gene flow magnitude and direction, we performed ABBA-BABA (*D*-test) and TreeMix analyses (Figs. R5, R6 below; also Revised Manuscript Fig. S5, S6). These revealed a multidirectional gene flow network: from hulled to naked oats, from Mediterranean and West Asian subpopulations to other cultivated groups, and across intercontinental regions. The detailed revisions are as follows: [L111-157]

"Based on 127,563 high-quality bi-allelic SNPs (MAF $\geq$ 0.05, miss $\leq$ 0.1), this study detected moderate introgression from cultivated oat to naked oat using the ABBA-BABA test and TreeMix analysis (D = 0.35, P < 0.01), persistent gene flow between hulled and naked oats resulted in weak differentiation (F_{st} = 0.0701) and significant genetic overlap (Figs. 1f and 2a; Tab. S2). Genetic diversity analysis revealed that hulled oat exhibited significantly higher nucleotide diversity ($\pi = 2.79 \times 10^{-6}$) and faster linkage disequilibrium decay (1.34 Mb) than naked oat ($\pi = 1.64 \times 10^{-6}$; 2.2 Mb). Together with the significantly higher proportion of long runs of homozygosity (ROH) (1-3 Mb) in naked oat (14.4%) than in hulled oat (9.5%), these results demonstrate that naked oat underwent a stronger genetic bottleneck and greater selection pressure during domestication (Figs. 1d, h; S6a, d; Tab. S2). Furthermore, by integrating Admixture cross-validation (K=1-20) with neighbor-joining (NJ)

phylogenetic analysis (rooted with wild hexaploids), we classified 666 oat accessions from 65 countries and regions into 6 genetic subgroups (G1-G6) (Figs. 1a, b and S1-S3; Tab. S1). While the core germplasms of each subpopulation had clear origins, genetic admixture between hulled and naked oats and cross-regional gene flow were prevalent, with no strict geographic-genetic correspondence observed (Figs. 1, S3-S6; Tabs. S1-S3). Specifically, G1 represented African-Asian convergence, with 57 Ethiopian-Egyptian hulled oats, 7 wild relatives, and a Chinese naked landrace from Hebei. G2, a hulled oat landrace core subpopulation from the Mediterranean and West Asia, exhibited the weakest domestication selection (Tajima's $D < 0$: 1.4%-4.8%) and highest nucleotide diversity ($\pi = 3.31 \times 10^{-6}$). Its genetic components introgressed into other subpopulations at 7%-70%, with Gene flow analysis further confirmed moderate-to-high introgression from G2 into other subpopulations and across geographic regions (D = 0.18-0.48), underscoring G2 as a key genetic donor in the global oat germplasm network. Despite such widespread gene flow, G2 remained strongly differentiated from these subpopulations ($F_{st} > 0.15$) due to geographic isolation and divergent selection pressures. G3 is a subpopulation dominated by Chinese naked oats, which has experienced strong domestication selective pressure, with the proportion of loci showing Tajima's $D < 0$ being 11.7 to 16.3 times that of G2, and exhibits significantly reduced genetic diversity. G4 reflected trans-Pacific gene flow with 43 North American hulled oats and 41 Chinese naked oats from northern domestication hubs. Gene flow analysis revealed bidirectional introgression between North American cultivated hulled oats and lineages from East Asia, the Mediterranean, and Europe. Frequent recent gene flow was detected with Mediterranean and European hulled oats (shared ROH >1 Mb: 26.2%-37.8%), indicating that G4 serves as a genetic fusion hub for global modern breeding germplasm. Driven by such extensive cross-regional gene exchange, G4 maintains relatively high nucleotide diversity ($\pi = 2.07 \times 10^{-6}$). G5 is a Sino-Eurasian mosaic which comprising 53 European hulled cultivars and 13 Chinese naked oat landraces from Shanxi-Hebei-Inner Mongolia, exhibits the lowest nucleotide diversity ($\pi = 1.26 \times 10^{-6}$) due to strong breeding and domestication pressures. G6 is a typical geographically adjacent admixed subpopulation, comprising 54 Mediterranean hulled oat landraces, 28 European hulled oat germplasms, and 55 accessions from Asia (including 28 Chinese naked oats). Principal component analysis (PCA) supports this structure and overall genetic admixture: only G2 shows independent clustering on PC1, and G3 (naked oat core) concentrates on PC3; otherwise, extensive overlap by origin and type confirms global admixture (Fig. 1c; Tabs. S1-3)."

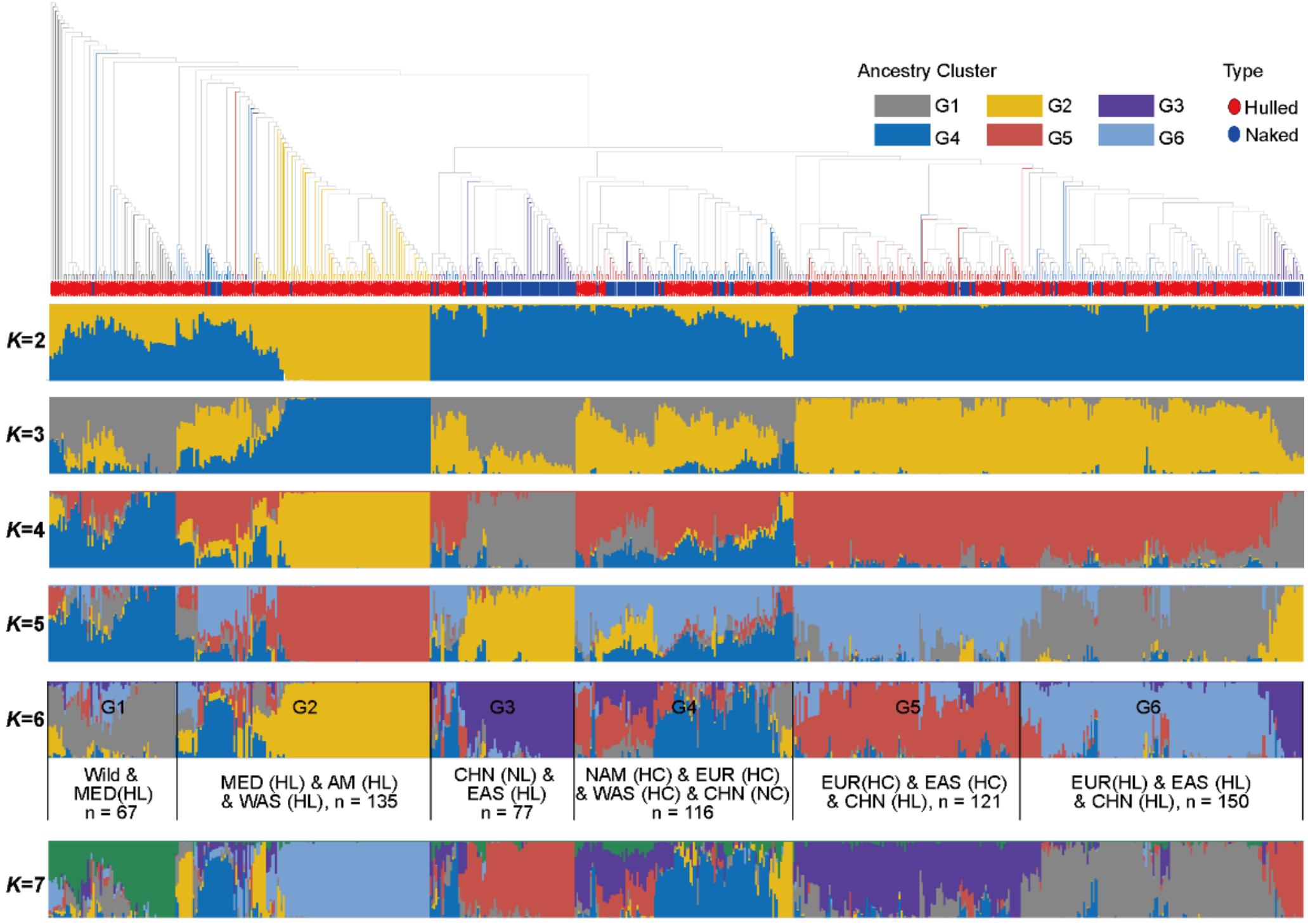

Fig. R3 shows the rooted NJ phylogenetic tree and Admixture plots at *K*=1 to 7 for 666 oat accessions, constructed with 127,563 high-quality SNP markers (Revised Manuscript Fig. S3).

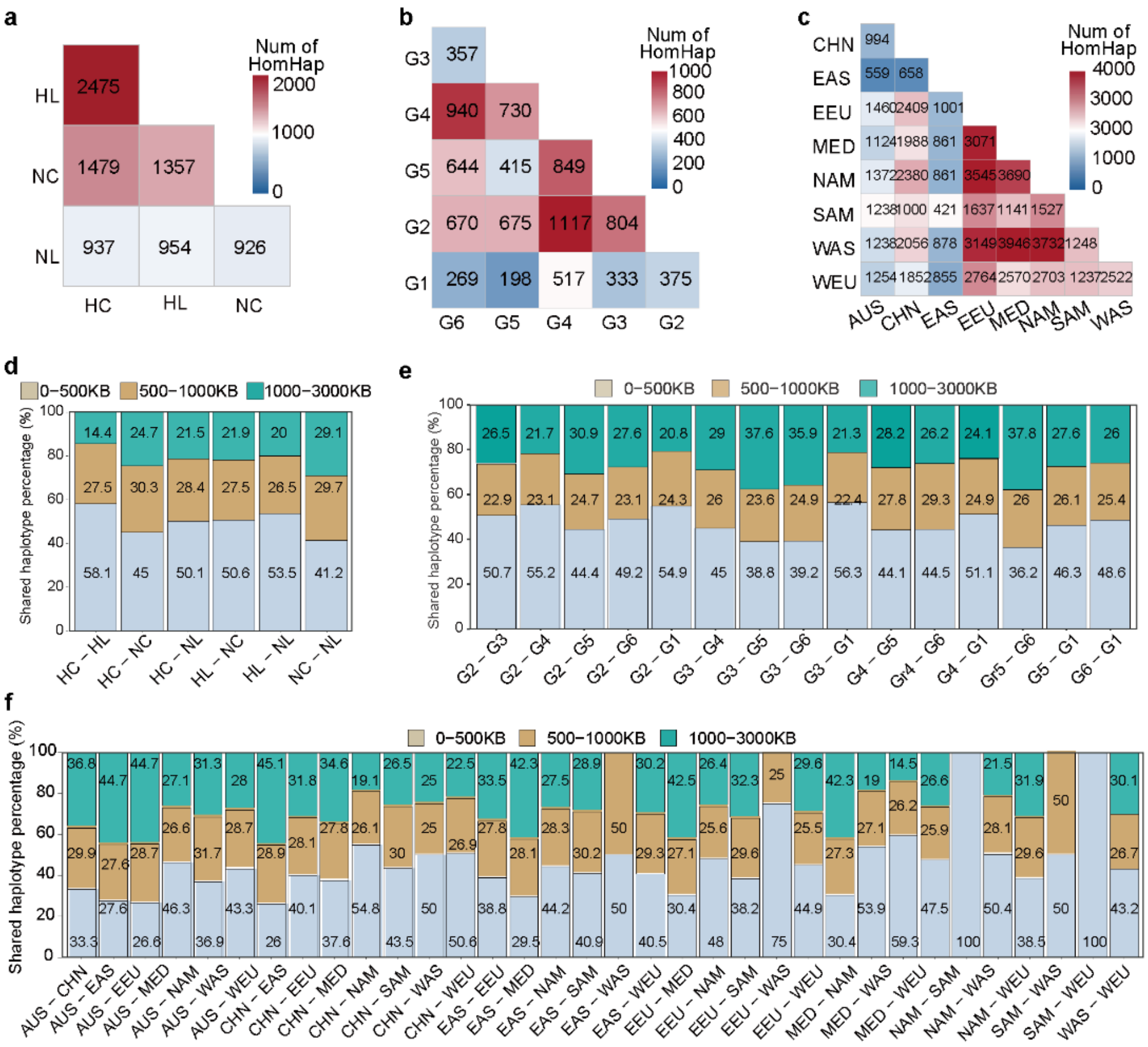

Fig. R4 presents results of shared homozygous haplotype analysis among different groups of the tested oat accessions (Length : 10 kb-3000 kb; Revised Manuscript Fig. S4). (a, d) Display the number of shared haplotypes and proportional distribution of haplotypes across length categories

for hulled and naked oat landraces and cultivars; (b, e) Display the same metrics among accessions from distinct clustered genetic subgroups; (c, f) Display the same metrics among accessions from distinct geographical regions.

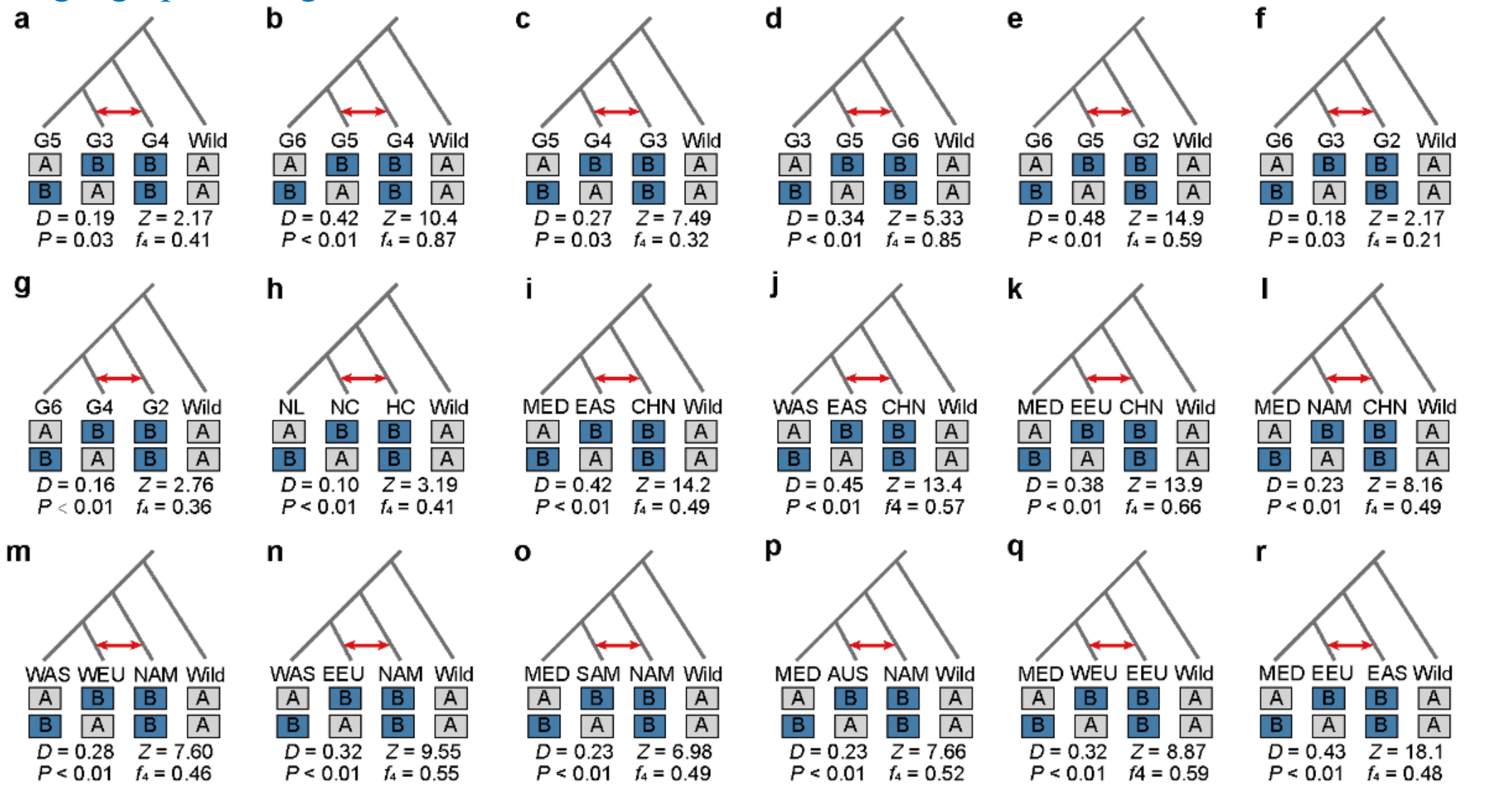

Fig. R5 presents *D*-statistic tests of gene flow events among distinct groups of the tested oat accessions (hulled vs. naked oats, distinct genetic subgroups, and distinct geographical regions) based on the ABBA-BABA model (Revised Manuscript Fig. S5).

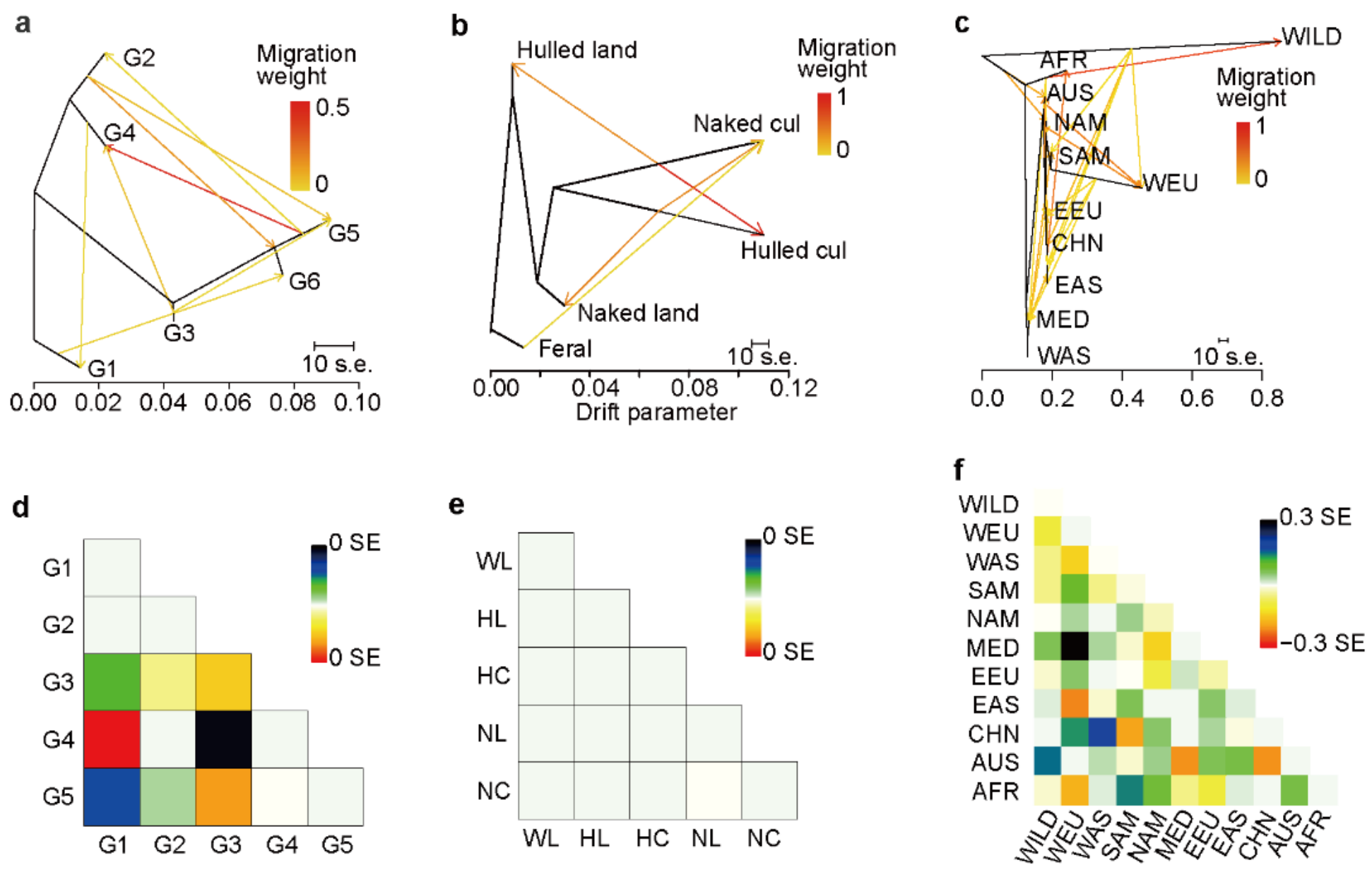

Fig. R6 presents gene flow analyses based on Treemix software (Revised Manuscript Fig. S6). (a-c) show the topology of maximum likelihood trees and the direction of inferred gene flow (indicated by arrows) under different numbers of gene flow events (m); (d-f) show the corresponding residual heatmaps for (a-c), used to assess model fit-lower residuals (lighter colors) indicate a more robust explanation of genetic relationships among the tested populations.

4. In the "Domestication pathway of naked oats" section, the authors conclude that naked oats originated from a key divergence during the eastward dispersal of Mediterranean hulled oat landraces and delineate the domestication trajectory of naked oats. However, the current evidence-gene flow analyses, Fst differentiation, and π statistics-is largely correlative. These analyses establish genetic affinities but do not directly demonstrate the direction, geographic location, or uniqueness of the proposed domestication route. A more quantitative model-based comparison (e.g., through

fastsimcoal2, or qpgraph topology testing) is required to distinguish among alternative origins, clarify the assumptions of the differentiation time model and to evaluate whether the Mediterranean-origin scenario is statistically favored over other plausible demographic models. The Discussion should more clearly distinguish supportive-but not decisive-evidence and avoid overinterpretation. Statements about the domestication center should be moderated unless supported by explicit demographic model testing.

RESPONSE: We thank you for the constructive comments on our conclusions regarding naked oat domestication. Your suggestions on distinguishing types of evidence and validating with quantitative models have helped us substantially improve the rigor of this study. We have accordingly refined our analytical logic and conclusion statements.

Population structure analyses based on both the full panel and landrace only accessions consistently revealed extensive genetic admixture between hulled and naked oats, indicating that naked oat landraces share a close evolutionary relationship with hulled landraces rather than being independently derived. The multidirectional intercontinental gene flow detected in this study aligns closely with patterns of genetic diversity and differentiation among subpopulations. The Mediterranean hulled oat subpopulation, which exhibits the highest diversity and strongest differentiation, served as a core genetic source with substantial gene flow to other groups. The low differentiation between the central eastern Eurasian hulled oat subpopulation and Chinese naked oat subpopulation similarly reflects their gene flow pattern, providing consistent evolutionary evidence for inferring the domestication route. We agree that analyses such as gene flow, F_{st}, and π represent indirect evidence: while effective for revealing genetic relatedness, they cannot alone define the direction and uniqueness of domestication routes. To address this, we first integrated these indirect lines of evidence to construct a logically consistent evolutionary framework. We then clarified hierarchical relationships among subpopulations using absolute nucleotide divergence (D_{XY}) and performed quantitative testing of multiple evolutionary models with fastsimcoal2 (Fig. R1 above; also Revised Manuscript Fig. 2). After removing highly admixed accessions, we statistically validated the proposed route in which Mediterranean hulled oat spread eastward and differentiated into central eastern Eurasian hulled oat, which subsequently gave rise to Chinese naked oat. This model outperformed alternative origin hypotheses, providing direct quantitative support for our conclusions. Within the Discussion section, we made a clear distinction between supportive and conclusive lines of evidence to mitigate the risk of overinterpretation regarding the oat domestication center and its dispersal routes. Our findings were contextualized and discussed alongside prior results from oat domestication studies, particularly those analyzing SNP and structural variations, to ensure the overall rigor and robustness of our conclusions.

5. Line 159-186: “The phenotypic divergence analysis” section, describes differences between naked and hulled oats in yield- and quality-related traits across multiple environments. However, 1) the causal links between these observed phenotypic differences and underlying genetic variation are not fully established. While PC analysis and multi-environment data reveal patterns of variation, these correlations do not clarify which specific loci or selection events drive the divergence. No genome-wide association (GWAS) or QTL analyses are integrated with the phenotypic data to directly link candidate genes or selection sweeps with observed trait differences. Such analyses would strengthen the inference of genetic determinants underlying the

phenotypic divergence. 2) Environmental effects across years and locations are not explicitly accounted for in the analyses. Without proper correction for environmental and management variation, the observed phenotypic differences may be confounded, potentially affecting conclusions about trait divergence between oat types.

RESPONSE: We thank you for noting the weak link between phenotypic differentiation and genomic evidence in our original manuscript. We agree that the analysis did not establish a clear causal relationship between genetic variation and phenotypic differences in hulled and naked oats, nor did it adequately account for environmental effects such as year and location. In response, we have performed targeted supplementary analyses, as detailed below:

1) To establish genetic links between phenotypic differentiation and genomic variation, we followed your suggestions to integrate three core datasets: (I) selective sweeps and 1,504 candidate genes identified through genome-wide scans (F_{st}, π ratio, XP-CLR); (II) previously reported QTLs associated with yield, quality, and stress resistance in oat (Fig. R7 below; also Revised Manuscript Fig. 4b); (III) transcriptome data from seed development and stress response experiments in hulled and naked oats (Fig. R8 below; also Revised Manuscript Fig. 5). We constructed an integrated framework combining selection signals, QTL colocalization, and differential expression. Through haplotype–phenotype association analysis, we further established direct links between selected regions and phenotypic variation. Details of these supplementary analyses are provided below: [L283-294]

"To investigate the association between selection signals and phenotypic differentiation, we colocalized selected genes in naked oats with published QTLs. We identified selected genes associated with 20 known QTLs across 11 chromosomes, and their selection patterns were highly consistent with phenotypic differentiation between hulled and naked oats. By trait category, only 11 of 51 genes linked to yield-related QTLs (e.g., thousand-kernel weight, grain size) showed directional selection. In contrast, 38 of 50 genes associated with crude protein content and 86 of 113 genes for β-glucan content exhibited signatures of directional selection (Fig. 4b; Tab. S6-2), indicating stronger selection on quality-related genes during naked oat domestication. Moreover, 117 of 178 genes linked to 5 drought-responsive QTLs showed directional selection (Fig. 4b; Tab. S6-6), implying enhanced adaptation to specific ecological niches."

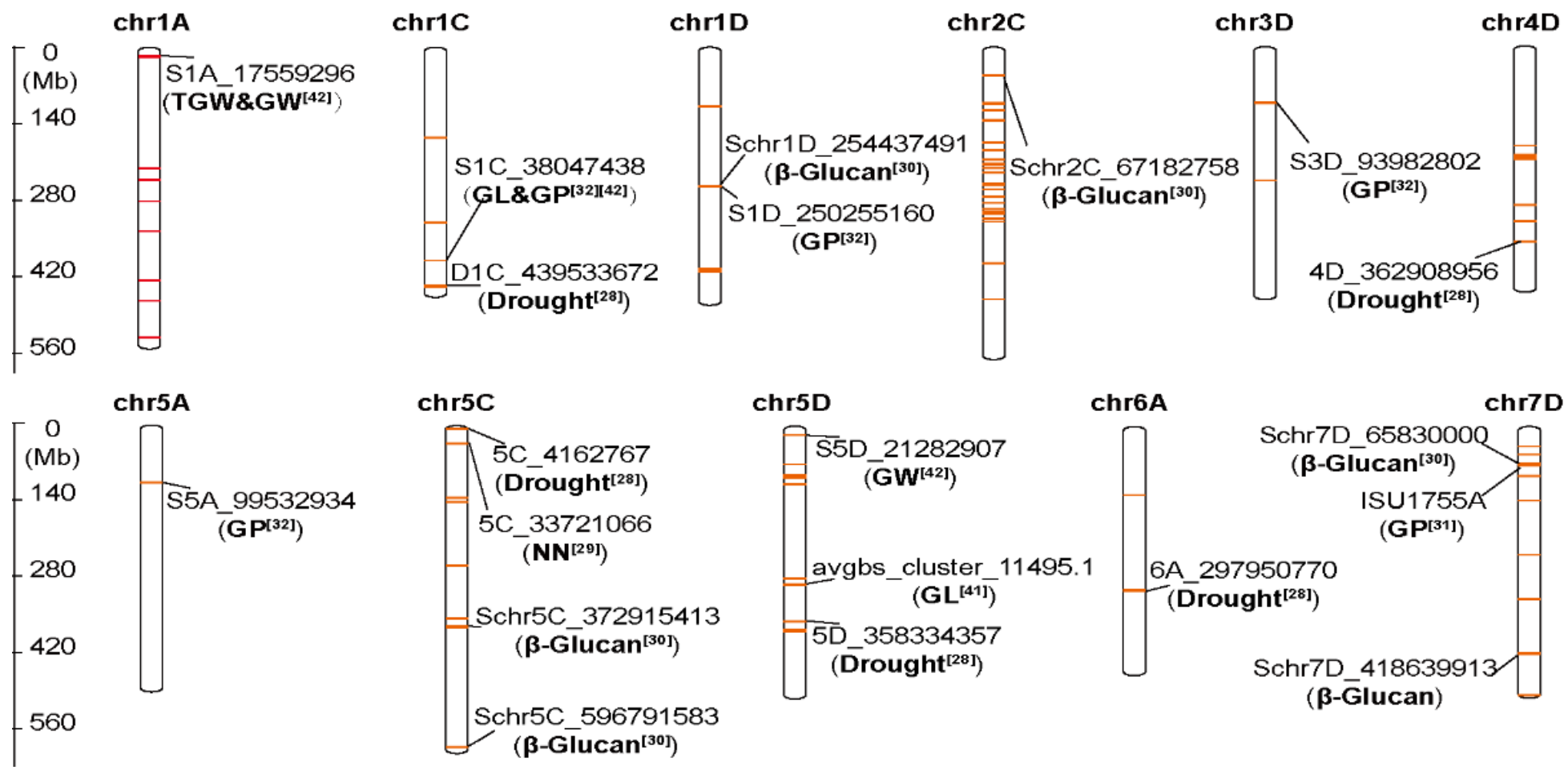

Fig. R7 Genome-wide colocalization analysis of selected gene loci and published QTLs in naked oats. (Red horizontal lines indicate the chromosomal positions of selected genes) (Revised Manuscript Fig. 4b)

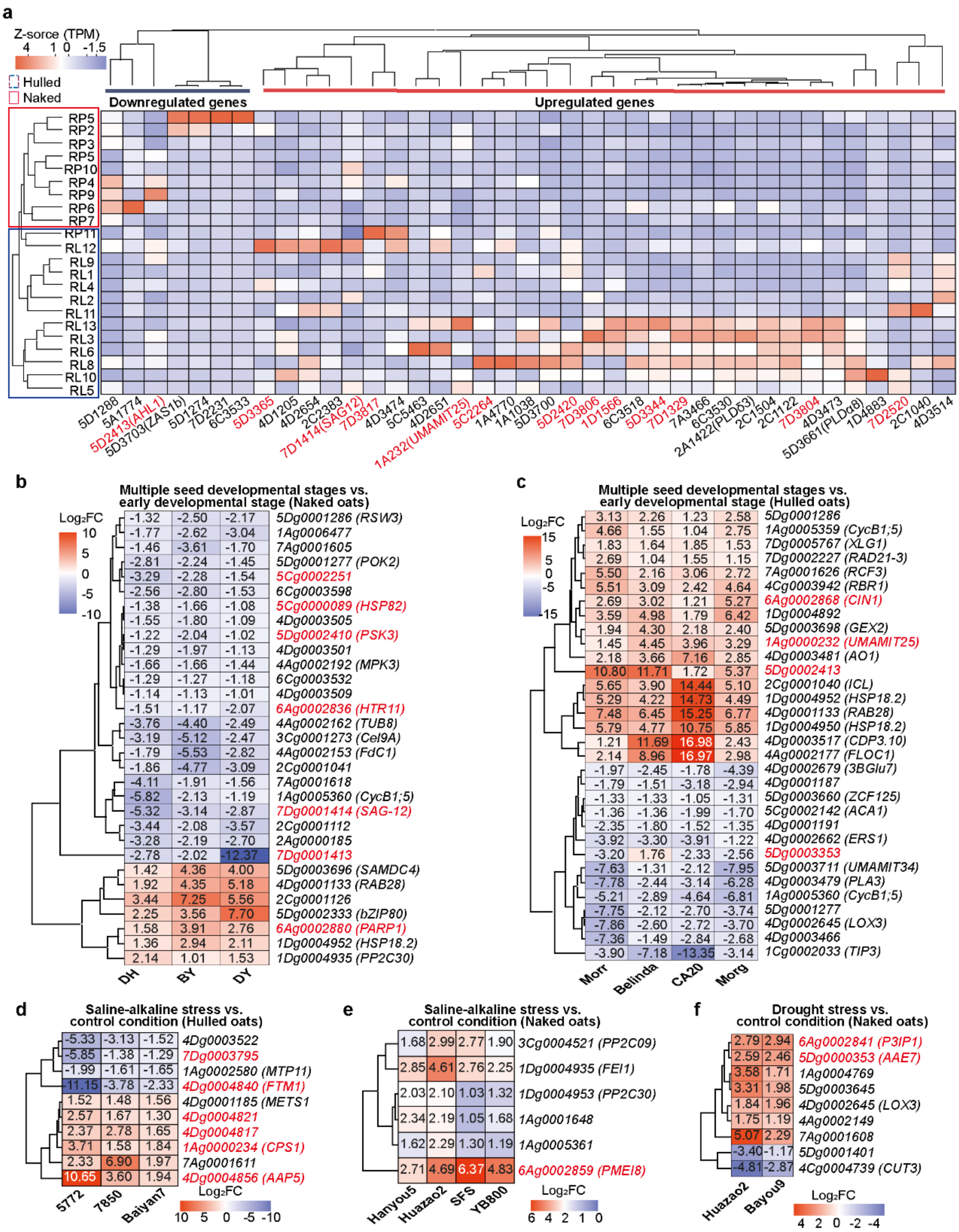

Fig. R8 Expression heatmap of selected and differentially expressed genes in naked oats based on Log₂FC values (Genes marked in red are associated with previously published oat QTLs) (Revised Manuscript Fig. 5). (a) Intersection of DEGs between hulled and naked oats from bulk RNA-seq and selected genes in naked oats; (b) Intersection of DEGs from seed development transcriptome of naked oats and selected genes in naked oats; (c) Intersection of DEGs from seed development transcriptome of hulled oats and selected genes in naked oats; (d) Intersection of DEGs from saline-alkali stress transcriptome of naked oats and selected genes in naked oats; (e) Intersection of DEGs from drought stress transcriptome of naked oats and selected genes in naked oats.

2) Phenotypic variations of 12 growth and development traits, 5 grain yield-related traits and 2 grain quality traits measured on test materials across multiple environments

were fitted with a mixed linear model: $y = X\beta + Z_g g + Z_{ge} ge + \varepsilon$. The likelihood ratio test (LRT_P) and Bayesian information criterion (BIC) were used to screen the optimal genetic model: LRT_P verified the significance of random effects by calculating $LRT = -2(\log L_{\text{simplified model}} - \log L_{\text{full model}})$, while BIC was applied to select the optimal non-nested model via the formula $BIC = -2\log L + k\log n$ (where k is the number of parameters and n is the sample size) (*Genetics and analysis of quantitative traits* 1998 https://doi.org/10.1017/S0016672398219732; *Introduction to quantitative genetics* 1996 https://doi.org/10.1016/0168-9525(96)81458-2). Furthermore, based on the genotypic variance (V_g), genotype × environment interaction variance (V_{ge}) and residual variance (V_ε) estimated by the optimal genetic model, the broad-sense heritability (H^2) of each trait was calculated using the formula $H^2 = V_g/(V_g + V_{ge}/E + V_\varepsilon/(R \times E))$.

Using genotypic and phenotypic data of the tested materials, we employed GCTA (*American Journal of Human Genetics* 2011 https://doi.org/10.1016/j.ajhg.2010.11.011) with the bivariate GREML model. Genome-wide SNPs were used to construct the GRM, and additive genetic variances and covariances between two traits were estimated to calculate genetic correlations, which reflect phenotypic associations caused by shared genetic factors (see Materials and Methods, Revised Manuscript L519-525). The detailed revisions are as follows: (Tab. R1 and R9 below; also Revised supplementary Tab. S5-1and Fig. S9; L213-226):

"Building on population structure analysis, we dissected the genetic basis of 19 oat agronomic traits using multi-environment phenotypic data (Tab. S6). Optimal genetic models and broad-sense heritability (H^2) were determined via LRT_P and BIC. Model analysis showed hulled/naked trait and spikelet type were regulated solely by variety main effects; awn presence, panicle type, grain width, crude protein, and crude fat were influenced by variety and variety × year interactions; most other traits were environmentally regulated. Heritability estimates were moderate for PH, HS, and ET (H^2 = 0.41–0.53) and high for all other traits ($H^2 \geq 0.6$), indicating predominant genetic control (Tab. S5-1). Genetic correlation analysis revealed significant negative correlations between yield-related traits (TGW, GL, GW) and quality traits (CFC, CPC) (r = –0.22 to –0.34, $P < 0.05$). Hulled traits correlated positively with yield traits (r = 0.28–0.49, $P < 0.01$), while naked traits correlated positively with quality traits (r = 0.53–0.67, $P < 0.01$), reflecting domestication-driven phenotypic specialization (Fig. S9; Tab. S5-2)."

Tab R1 G×E interaction effects and broad-sense heritability for 19 agronomic traits. (Revised Supplementary Tab. S5-1)

Trait	Best_Model	Var_ Line	Var_ Year	Var_ Loc	Var_ Res	H^2	LRT_*P*	BIC
H/N	ID	0.18	0	0	0.05	0.96	0.03	-18184.35
AWN	ID+YEAR	1.11	0	0	0.1	0.78	0.0001	4822.54
ST	ID+YEAR	0.63	0.01	0	0.06	0.95	0.0001	3812.9
SLT	ID	0.18	0	0	0.02	0.95	0.0001	-5345.56
PH	ID+YEAR+LO	239.71	40.46	625.05	20	0.41	4.30E-195	31936.24
PL	ID+YEAR+LO	47.61	0	8.43	5	0.81	4.68E-206	19009.41
HS	ID+YEAR+LO	63.79	42.21	64.2	6	0.53	7.11E-162	23778.96
GP	ID+YEAR+LO	70.33	46.54	70.77	7	0.53	7.11E-162	24082.63
LFL	ID+YEAR+LO	8.18	0	15.71	1.3	0.55	4.91E-46	12224.3
WLF	ID+YEAR+LO	0.07	0.12	0.02	0.03	0.56	1.11E-61	2367.57
ET	ID+YEAR+LO	18.64	3.4	0	1.8	0.62	2.29E-36	31179.7

NN	ID+YEAR+LO	0.37	0	0	0.03	0.8	8.39E-115	8329.62
SN	ID+YEAR+LO	164.84	24.04	0	36	0.71	2.29E-86	25354.43
GN	ID+YEAR+LO	1846.1	698.95	949.94	180	0.78	6.69E-41	11937.03
TGW	ID+YEAR+LO	17.38	1.49	2.48	2	0.75	8.80E-48	9431.61
GL	ID+YEAR	0.59	0.31	0	0.06	0.79	0.587424	8786.27
GW	ID+YEAR+LO	0.07	0.01	0	0.01	0.9	1.94E-203	-317.13
CPC	ID+YEAR	0.02	0	0	0	0.75	0.000123	1398.82
CFC	ID+YEAR	0	0.01	0	0	0.69	0.00001	-3206.07

Notes: These traits and their abbreviations are as follows: heading stage (HS), growth period (GP), spike type (ST), spikelet type (SLT), effective tiller number (ET), plant height (PH), spike length (PL), flag leaf length (LFL), flag leaf width (WFL), stem diameter (SD), node of number (NN), awn presence (AWN), spikelet number (SN), Hulled/Naked (H/N), grains per spike (GN), thousand-grain weight (TGW), grain length (GL), grain width (GW), and grain circumference index (GCI); crude protein content (CFC), crude fat content (CPC).

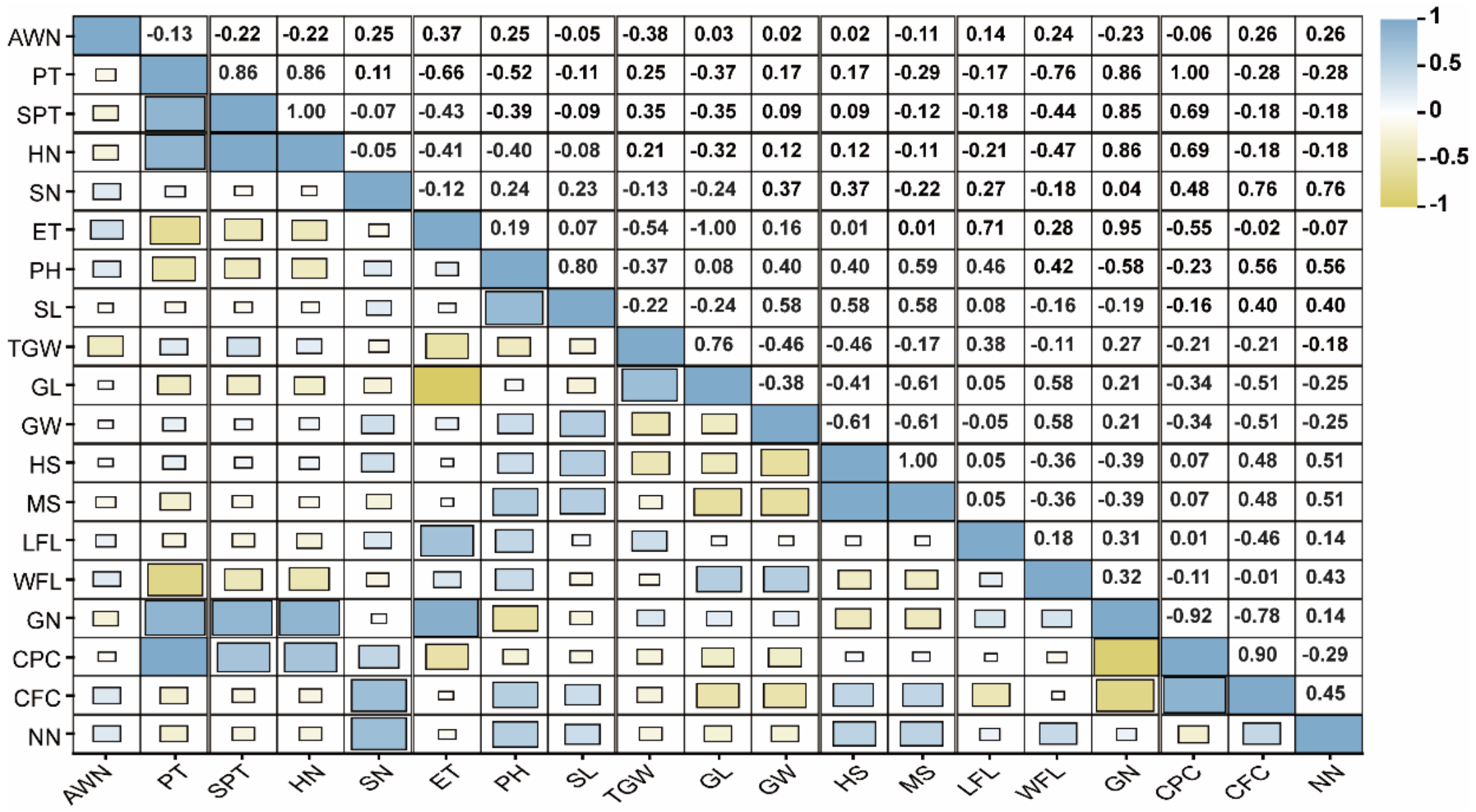

Fig. R9 Genetic correlation heatmap of 17 agronomic traits and 2 grain quality-related traits in oat (Revised Manuscript Fig. S9).

6. Line 195-197: The authors define the top 1% of windows based on Fst, π ratio (hulled/naked), and XP-CLR as candidate selected regions, identifying 1,504 putative selected genes. However, the rationale for choosing the top 1% threshold is not statistically justified, and no cross-validation is presented. Given the complex population structure and evidence of extensive gene flow, relying on a single XP-CLR or Fst metric may generate false positives. I recommend implementing multiple complementary methods or using simulation/resampling approaches to assess the robustness of candidate gene identification.

RESPONSE: We sincerely thank you for identifying the lack of statistical justification for threshold setting and the absence of cross-validation in the genome-wide selection scan. We take this feedback seriously and have supplemented and refined the relevant supporting evidence, as detailed below: For the genome-wide selection scan, we first extracted and reformatted VCF subfiles for 504 hulled oats and 162 naked oats using BCFtools (*Bioinformatics* 2016 https://doi.org/10.1093/bioinformatics/btw044). leveraging the high-quality VCF file of all tested accessions. Subsequently, we calculated the nucleotide diversity (π) and fixation index (F_{st}) across the genomes of

these two groups using VCFtools (*Bioinformatics* 2011 https://doi.org/10.1093/bioinformatics/btr330), with a 500 kb window and 50 kb step size (Revised Manuscript L649-663). Based on these analysis results, we established screening thresholds for putative selected loci in naked oats: the core threshold was set as a genome-wide LogF_{st} (differentiation index) > 2.7 ($P < 0.001$) between hulled and naked oats. Additionally, we calculated the nucleotide diversity ratio (π) at the same genomic loci between the two groups and combined these results with XP-CLR analysis outcomes. For both analyses, the top 1% of loci ($\pi > 59.07$) were selected as auxiliary screening thresholds using the percentile method. This threshold-setting approach is consistent with standard practices in crop population genomics research (*Nature genetics* 2012 https://doi.org/10.1038/ng.2309; *The Plant cell* 2023 https://doi.org/10.1093/plcell/koad229) and supported by robust methodological rationale. To mitigate false positives potentially caused by complex population structure and gene flow, we implemented a multi-faceted validation strategy integrating QTL co-localization (LD half-decay distance = 2 Mb), intersections of transcriptome-derived differentially expressed genes, and haplotype-phenotype association analysis. This strategy allowed us to focus on core candidate genes and effectively eliminate false positive signals, thereby enhancing the reliability of the 1504 candidate selected genes.

7. Line 197-199: Tajima's D <0 as evidence of positive selection and D ≥ 0 as negative or balancing selection. This interpretation is overly simplistic because Tajima's D is also sensitive to demographic history, gene flow, and genetic drift. The authors should explicitly discuss these confounding factors or consider more rigorous statistical approaches to distinguish true selection from demographic effects.

RESPONSE: We sincerely thank you for your rigorous feedback! We employed a multi-criteria joint validation approach integrating XP-CLR, F_{st} values, and *π* ratios, which has eliminated interference from confounding factors such as single-indicator bias, genetic drift, and gene flow-thus ensuring the reliability of selected loci. On this basis, we used Tajima's *D* statistic combined with nucleotide diversity (*π*) to infer selection modes (Fig. R10 below; also Revised Manuscript Fig. S12). The core logic is: Tajima's D reflects the degree to which allele frequencies deviate from neutral evolution ($D < 0$ indicates enrichment of low-frequency alleles, and $D \geq 0$ indicates enrichment of intermediate-frequency alleles), while π quantifies population polymorphism levels. If a genomic region meets three criteria simultaneously: naked oats have Tajima's $D < 0$ and significantly lower π relative to hulled oats (the average π reduction rate across the 17 chromosomes is 37.75% to 73.92%), and hulled oats have Tajima's $D > 0$ at the same locus (with enrichment of intermediate-frequency alleles and high polymorphism), we can infer, based on crop domestication principles, that directional selection occurred in this region during naked oat domestication and improvement-characterized by enrichment of advantageous alleles and loss of overall polymorphism.

We fully agree with your comment that Tajima's *D* is influenced by factors such as population evolutionary history, and that Tajima's *D*, when combined with *π*, is more suitable for inferring directional and balancing selection than for directly defining positive or negative selection, as interpreting a single indicator has inherent limitations. To improve the rigor of the manuscript, we have revised relevant terminology, replacing "positive/negative selection" with the more objective "tendency for directional or balancing selection", and reorganized the logical flow of the results section. The detailed revisions are as follows: [L259-294]

“The pronounced phenotypic differences between hulled and naked oats in growth, yield, and quality traits indicate substantial genomic differentiation between the two oat types. Building upon established population structure and phenotypic differentiation between hulled and naked oats, we quantified chromosomal-level genetic divergence during oat domestication using Tajima's D and nucleotide diversity (π). The results showed that except for chromosomes 2D, 3A, 6D, and 7C (π differences , $P > 0.05$; Tajima's $D \geq 0$ spanning >83%), the remaining 17 chromosomes, naked oats showed significantly reduced π (loss rate: 37.75%–73.92%, $P < 0.001$) and elevated selection pressure ($D < 0$; 29-fold increase) relative to hulled oats(Fig. S11; Tab. S6), indicating that naked oats underwent a stronger genetic bottleneck and more intensive artificial selection during domestication, thereby laying the genomic foundation for their distinct phenotypic differentiation. Integrating the population differentiation index (F_{st}), π ratio (hulled/naked), and XP-CLR, We screened for potential selective sweeps in naked oats （LogF_{st} > 2.80, $P < 0.001$, and the top 1% of windows identified by π ratio and XP-CLR analyses） we identified 1,504 putative selected genes (Fig. 4a; Tab. S6). Among these, 883 exhibited directional selection ($D < 0$), and 621 tended toward balancing selection ($D \geq 0$) (Fig. S12, Tab. S6-4). Functional enrichment analyses showed selected genes retain core cellular metabolism but diverge markedly in domestication pathways. Directional selected genes significantly enriched lipid, amino acid, and vitamin biosynthesis pathways (GO:0009110, GO:0019375, ko00591), directly correlating with enhanced nutrient accumulation in naked oats (Fig. S13； Tab. S6-4). In contrast, directional selected genes clustered in chlorophyll a oxidase activity (GO:0016049, ko00195) and butanoate metabolism (ko00650), suggesting compromised photosynthetic efficiency and growth regulation in naked oats (Fig. S14； Tab. S6-5).

To investigate the association between selection signals and phenotypic differentiation, we colocalized selected genes in naked oats with published QTLs. We identified selected genes associated with 20 known QTLs across 11 chromosomes, and their selection patterns were highly consistent with phenotypic differentiation between hulled and naked oats. By trait category, only 11 of 51 genes linked to yield-related QTLs (e.g., thousand-kernel weight, grain size) showed directional selection. In contrast, 38 of 50 genes associated with crude protein content and 86 of 113 genes for β-glucan content exhibited signatures of directional selection (Fig. 4b; Tab. S6-2), indicating stronger selection on quality-related genes during naked oat domestication. Moreover, 117 of 178 genes linked to five drought-responsive QTLs showed directional selection (Fig. 4b; Tab. S6-6), implying enhanced adaptation to specific ecological niches.”

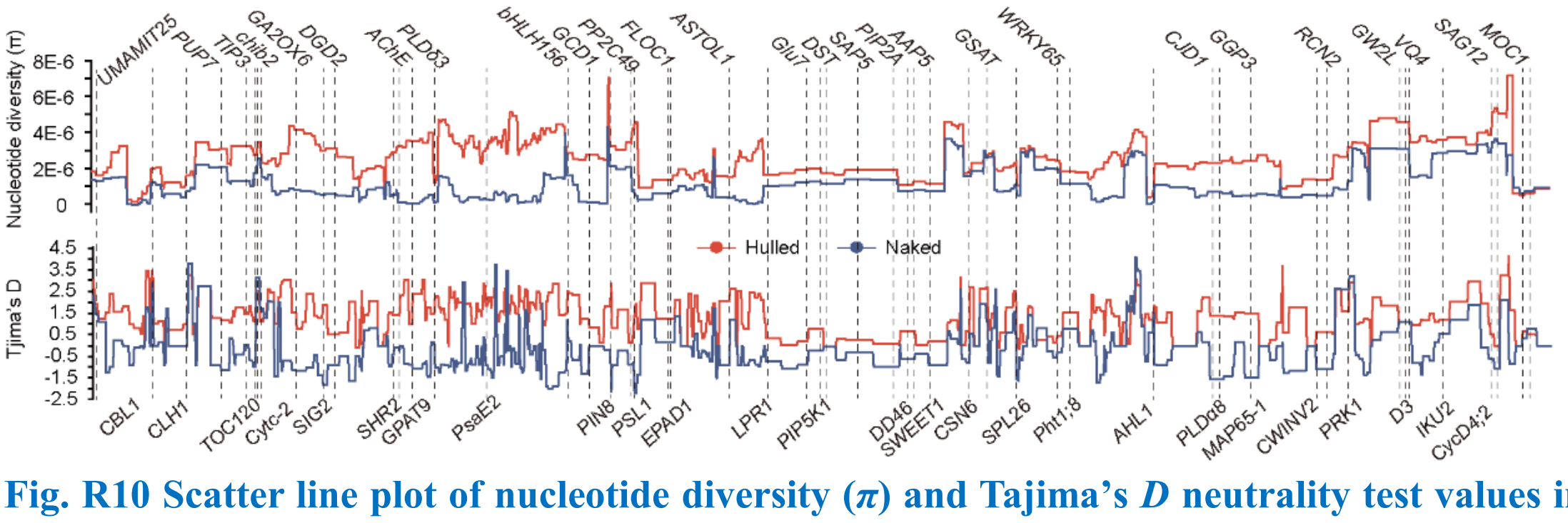

Fig. R10 Scatter line plot of nucleotide diversity (π) and Tajima’s D neutrality test values in genomic regions harboring 1504 selected genes between hulled and naked oats (Revised Manuscript Fig. S12).

8. Line 200-224: The authors link enriched pathways and specific haplotypes (e.g., GPAT9, SAE2) to nutritional and yield traits. However, functional enrichment and frequency-based associations only indicate correlation, not causation. The manuscript should avoid overstating direct phenotypic effects and clarify that these are putative associations unless supported by GWAS or functional validation.

RESPONSE: We apologize for overinterpreting statistical correlations as causal relationships in the analysis of associations between selected genes and phenotypic traits. In line with your suggestions, we have adjusted the narrative logic in the revised manuscript to downplay direct causal claims: all definitive phrasing (e.g., "directly correlated", "closely correlated", "significantly associated") in the original text has been replaced with neutral terminology including "exhibit consistency", "show consistent trends", "correspond with", and "have a corresponding relationship". We have also explicitly noted the speculative nature of these conclusions and the need for subsequent functional validation to ensure rigorous, accurate presentation of our results. The detailed revisions are as follows: [L294-306]

"Homology annotation and allele frequency analysis further identified multiple selected genes linked to phenotypic divergence between hulled and naked oats. For example, accumulation of beneficial haplotypes of glyceride synthesis gene *GPAT9* and seed ovule development gene *SAE2* in naked oats exhibit consistency with higher grain crude protein and fat. Conversely, selective sweeps of favorable haplotypes in grain development regulator *GSA1* and aquaporin gene *TIP3* exhibit consistency with reduced naked oat yield traits. Furthermore, selective sweep of the Hap2 haplotype in photodamage repair gene *CLH1* exhibit consistency with shorter spikes and fewer tillers in naked oats. Similarly, Hap2 sweep in photoperiod regulator *EL1* likely underlies delayed flowering. Haplotype–phenotype associations for these selected genes confirmed the link between genome-wide selection signals and phenotypic divergence, providing valuable candidate targets for marker-assisted selection (MAS) in oat breeding (Fig. 4c； Tab. S8-2)."

9. Line 226-229: "from 13 naked oat and 9 hulled oat accessions"-the sample size is relatively limited given the global diversity and complex population structure of Avena. The selected accessions may not adequately represent the full genetic breadth of hulled and naked oats, which restricts the generalizability of the inferred transcriptional differences.

RESPONSE: We sincerely appreciate your insightful comments on the sample size for transcriptome analysis. We fully agree that the 9 hulled oat and 13 naked oat accessions used in this study are limited in representing the extensive genetic diversity and complex population structure of global oat germplasm, which may compromise the generalizability of our conclusions. In response, we have made targeted supplements and objective clarifications to both the experimental design and the manuscript.

In the experimental design, the selected accessions were prioritized for maximum representativeness in geographical origin and genetic background. Given limited sequencing costs, we adopted a mixed sampling strategy for root, stem, leaf and panicle tissues to generate transcriptome data-a design intended to rapidly capture the basal, broad transcriptional regulatory differences underlying physiological variations between hulled and naked oats, thus comprehensively characterizing transcriptional regulation across key plant organs. To mitigate the limitations of a single sample set, we further integrated 7 independent transcriptome datasets for seed development, 7 for

salt-alkali stress, and 2 for drought response, establishing a joint analysis framework across multiple physiological scenarios to identify conserved regulatory patterns. On this basis, we performed systematic differential expression and functional enrichment analyses, and combined these with cross-validation via the integration of selected genes, differentially expressed genes and QTL co-localization-yielding 117 core associated genes that provide internal validation for our conclusions (Figs. R11 below; also Revised Manuscript Fig. S15. Figs. R8 above; also Revised Manuscript Fig. 5)

We fully acknowledge this limitation and have therefore rigorously framed the relevant conclusions as: The transcriptional regulatory differences revealed in this study provide a basis for identifying core molecular clues underlying phenotypic divergence between hulled and naked oats. We have also explicitly noted this limitation-insufficient sample size preventing full capture of the genetic diversity of global oat germplasm-in the discussion section, and proposed specific future research directions including expanding the sample size and incorporating germplasm from more geographically diverse origins (Revised Manuscript L470-472). These adjustments respect the objective boundaries of the study and ensure that core conclusions based on available data remain robust and rigorous. The detailed revisions are as follows: [L308-355]

"To investigate transcriptional regulatory divergence between hulled and naked oats, we conducted RNA-seq analysis on pooled root, stem, leaf, and spike tissues from 13 naked oat and 9 hulled oat accessions with geographically and genetically diverse backgrounds, and further integrated 16 public transcriptome datasets (7 for seed development, 9 for salinity and drought stress) for comprehensive multi-dimensional comparisons (Tab. S7-2). Enrichment analysis of differentially expressed genes (DEGs) during seed development revealed that upregulated genes in naked oats were primarily associated with pathways related to nutritional quality accumulation, including developmental signaling, sterol-lipid synthesis, and ion channel activity (e.g., GO:0009873, GO:0009867, GO:0000149, ko00906, ko00543; *q*value < 0.05).This suggests their transcriptional regulation tends to favor nutrient synthesis and accumulation in the grain. In contrast, upregulated genes in hulled oats during seed development were primarily enriched in pathways for cell wall organization, transmembrane transport, and basal metabolism (e.g., GO:0009834, GO:0070592, ko02010, ko00430; *q*value < 0.05), potentially linking to nutrient transport efficiency and growth regulation. Under abiotic stress, naked oats upregulated genes involved in ion homeostasis, stress signal transduction, and specific secondary metabolism (GO:0006561, GO:0017157, ko99988, ko00906). Under abiotic stress, naked oats upregulated genes involved in ion homeostasis, stress signal transduction, and specific secondary metabolism (GO:0006561, GO:0017157, ko99988, ko00906), whereas hulled oats exhibited enrichment in pathways for cell wall remodeling, terpenoid biosynthesis, and ABC transporter-mediated transport (GO:0009834, GO:0016102, GO:0006721, ko02010; *q*value < 0.05), highlighting distinct transcriptional bases underlying their differential stress adaptation strategies (Figs. S15, S16; Tab. S7).

To identify key genes underlying hulled-naked oat differentiation, we integrated 1,504 candidate selected genes in naked oats with multi-context transcriptome data to construct a combined selection signal-expression association profile. Our results revealed that during core physiological processes, we identified 39 selected genes

exhibiting differential expression between hulled and naked oats, comprising 31 upregulated and 8 downregulated genes (e.g., *UMAMIT25*, *AHL1, SAG12*, *ygl8*, *WOX13*). Among these, 22 genes under directional selection and upregulated expression were significantly enriched in pathways such as lipid metabolism and post-translational protein modification (Fig. 5a; Tab. S7-6). During the seed development stage, a total of 31 selected genes were identified. Among them, upregulated genes (e.g., *PARP1*, *RAB28*, *PP2C30*, *HSP18.2*) may be involved in embryonic cell division and differentiation and ABA signal response, whereas genes under balancing selection and downregulated expression (e.g., *MPK3*, *CycB1;5*, *RSW3*) were enriched in cell cycle regulation pathways (*q*value < 0.05) (Fig. 5b; Tab. S7-6). In contrast, the 32 differentially expressed genes related to seed development in hulled oats exhibited a pattern of cooperatively upregulating energy metabolism and signal transduction while downregulating redundant hormone pathways (Fig. 5c; Tab. S7-6). Furthermore, in response to abiotic stress, 25 selected genes specifically expressed in naked oats (e.g., *PP2C09*, *FEI1*, *PME18*, *PIP3I*) generally displayed signatures of directional selection and upregulated expression, suggesting their potential functions in stress adaptation (Fig. 5c-f; Tab. S7-6). Further analysis revealed that 33 of the aforementioned 127 associated genes co-localized with published QTLs for oat yield and quality traits (Fig. 5; Tab. S7-5, highlighted in red), significantly enhancing the credibility of these candidate genes in trait differentiation.”

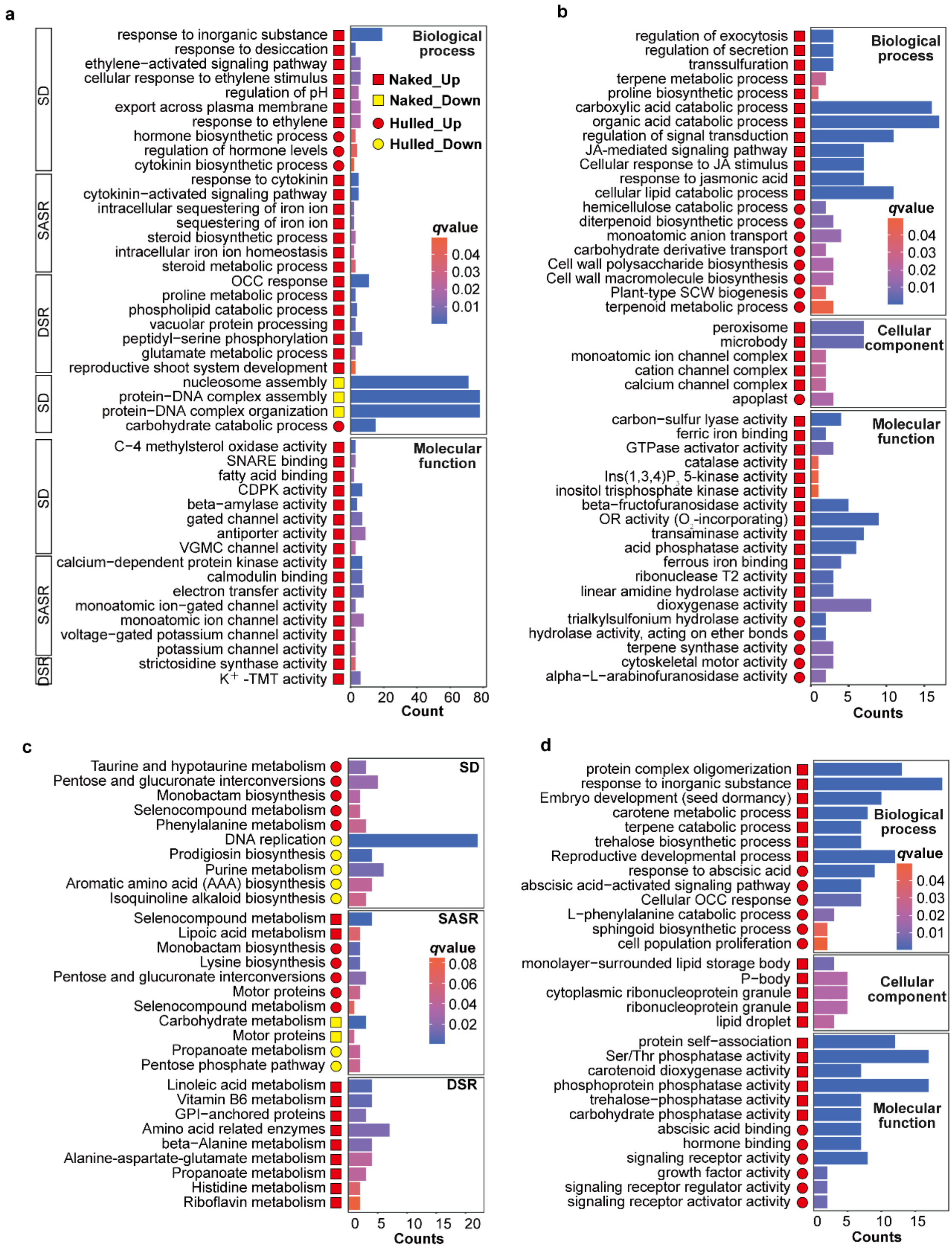

Fig. R11 Functional enrichment analysis of differentially expressed genes (DEGs) in hulled/naked oats across transcriptome analyses of basal physiology, seed development, and abiotic stress. (Abbreviations for each transcriptome project: SASR: Salt-alkali stress treatment; SD: Seed development; DSR: Drought stress treatment. Pooled multi-tissue samples refer to mixed samples of oat spikelets, leaves, and stems at the grain filling stage) (Revised Manuscrip Fig. S15). (a) Shared GO term enrichment analysis for DEGs commonly identified in pooled multi-tissue samples and condition-specific transcriptomes (SASR, SD, DSR); (b) GO term enrichment analysis for DEGs specifically up-regulated in hulled/naked oats during the seed development (SD) stage; (c) Shared KEGG pathway enrichment analysis for DEGs in pooled multi-tissue samples and condition-specific transcriptomes (SASR, SD, DSR); (d) GO term enrichment analysis for DEGs specifically up-regulated in hulled/naked oats under abiotic stress (SASR, DSR).

10. Lines 250-257: The section claims that "naked oats prioritize expression of nutrient metabolism genes," yet the RNA-seq data were generated from pooled tissues (root, stem, leaf, and spike) — making it impossible to determine whether the observed expression differences are driven by grain-specific processes or by other tissues (e.g., root or stem). Moreover, among the 39 differentially expressed and selected genes highlighted in Fig. 5c, many are annotated as "cytoskeletal" or "transporter" genes - which are not directly involved in nutrient biosynthesis. This weakens the causal link between gene expression and grain nutritional traits. The authors must either supplement the analysis with tissue-specific RNA-seq (e.g., developing spikelets or seeds) or explicitly acknowledge that the expression patterns observed may not reflect grain-specific regulation. Without this, the inference that "naked oats prioritize nutrient synthesis at the transcriptional level" remains speculative.

RESPONSE: We thank the reviewer for pointing out that our previous mixed-tissue analysis could not distinguish grain-specific expression and that the association between naked oats and nutrient-metabolism-related genes lacked sufficient support. To address this, we obtained and analyzed 7 targeted transcriptome datasets of oat grain development (3 hulled oats and three naked oats) from public databases. Pathway enrichment analysis ($q < 0.05$) performed on the consistently up-regulated differentially expressed genes (DEGs) from these grain-specific datasets, cross-validated with the original mixed-tissue results, revealed significant enrichment of pathways related to nutrient accumulation in naked oat grains, including steroid biosynthesis, fatty acid binding, and cellular lipid metabolism (Fig. R11b, c; also Revised Manuscript Fig. S15). This confirms that the expression patterns detected in our study originate from grain-specific regulatory mechanisms.

To further strengthen the evidence, we intersected the grain-specific DEGs with the 1,504 selected genes in naked oats, identifying 31 core overlapping genes associated with grain development (Fig. R7b, c above; also Revised Manuscript Figs. 5b, c). Among these, 22 genes showed both up-regulation and signals of directional selection in naked oats, and they were significantly enriched in pathways directly linked to nutrient accumulation ($q < 0.05$). Together, these results construct a systematic evidence chain comprising grain-specific transcriptional enrichment, selection-signal validation, and phenotypic association. All relevant descriptions, figures, and interpretive analyses have been added to the corresponding sections of the revised manuscript. The specific revision content can be found in the previous response. [L308-350]

"To identify key genes underlying hulled-naked oat differentiation, we integrated 1,504 candidate selected genes in naked oats with multi-context transcriptome data to construct a combined selection signal-expression association profile. Our results revealed that during core physiological processes, we identified 39 selected genes exhibiting differential expression between hulled and naked oats, comprising 31 upregulated and 8 downregulated genes (e.g., *UMAMIT25*, *AHL1, SAG12*, *ygl8*, *WOX13*). Among these, 22 genes under directional selection and upregulated expression were significantly enriched in pathways such as lipid metabolism and post-translational protein modification (Fig. 5a; Tab. S7-6). During the seed development stage, a total of 31 selected genes were identified. Among them, upregulated genes (e.g., *PARP1*, *RAB28*, *PP2C30*, *HSP18.2*) may be involved in embryonic cell division and differentiation and ABA signal response, whereas genes under balancing selection and

downregulated expression (e.g., *MPK3*, *CycB1;5*, *RSW3*) were enriched in cell cycle regulation pathways (*q*value < 0.05) (Fig. 5b; Tab. S7-6). In contrast, the 32 differentially expressed genes related to seed development in hulled oats exhibited a pattern of cooperatively upregulating energy metabolism and signal transduction while downregulating redundant hormone pathways (Fig. 5c; Tab. S7-6). Furthermore, in response to abiotic stress, 25 selected genes specifically expressed in naked oats (e.g., *PP2C09*, *FEI1*, *PME18*, *PIP31*) generally displayed signatures of directional selection and upregulated expression, suggesting their potential functions in stress adaptation (Fig. 5c-f; Tab. S7-6). Further analysis revealed that 33 of the aforementioned 127 associated genes co-localized with published QTLs for oat yield and quality traits (Fig. 5; Tab. S7-5, highlighted in red), significantly enhancing the credibility of these candidate genes in trait differentiation."

11. Lines 325-337: The Results section completely overlooks the large-scale inversion on chromosome 7D (H4 haplotype), recently identified by Bekele et al. (2024) as a key structural variant (SV) strongly associated with naked grain development and domestication in oat. While the authors rely exclusively on SNP-based analyses, they fail to integrate or even mention this well-established SV, which has been shown to co-segregate with the naked phenotype and exhibit strong geographic and phenotypic correlations. This omission represents a significant gap in the genomic interpretation of domestication. The authors must supplement their analysis by examining the distribution of the chr7D inversion across their 10 genetic subgroups (e.g., GI-GX), and/or perform co-localization analysis to test whether their SNP-based selection signals (e.g., XP-CLR peaks) overlap with the inversion breakpoints or haplotype blocks. Without this, the study's conclusion on the genomic basis of naked oat domestication remains incomplete.

RESPONSE: We thank you for this critical suggestion. In the Discussion section of the revised manuscript, we referred to the core 7D inversion interval reported by Bekele et al. (*Nature communications* 2025 https://doi.org/10.1038/s41467-025-57895-3) performed SNP-centered 31-mer analysis using the same NC_GBS data, and finely characterized the haplotype distribution of chr7D across 591 accessions (Fig. R2 above; also Revised Manuscript Fig. S7):

Fine-scale chr7D haplotype analysis with NC_GBS data supports naked oat evolution in the present study. First, the 7D-H3 haplotype dominates Chinese naked oats (55%), matching Eurasian hulled oat patterns (*Nature communications* 2025 https://doi.org/10.1038/s41467-025-57895-3) indicating derivation from the mainstream hulled lineage characterized by the 7D-H3 haplotype. Second, the 7D-H4 haplotype, despite high frequency in North China naked landraces (30%), appears in only 1 out of 23 Turkish hulled landraces, arguing against common independent origin. Furthermore, 7D-H4 accessions show coordinated phenotypes: tall (152.23 cm), late heading (~10 d), increased protein (19.6%) and fat (6%) (Fig. S7; Tabs. S2-2, S4-1, S4-5), this combination of "tall stature, late maturity, and high nutritional quality" aligns with the requirements of traditional grain-forage dual-purpose systems in Northern China. Additionally, selection signals in the chr7D inversion (256.6-412.1 Mbp) in naked oats (Fig. 4a, b; Tab. S6-2) suggest local adaptation roles. Notably, naked grain loci reside on 4D (*Theoretical and Applied Genetics* 2020 https://doi.org/10.1007/s00122-020-03674-1; *Nature genetics* 2022

https://doi.org/10.1038/s41588-022-01127-7; *Gigascience* 2022 https://doi.org/10.1093/gigascience/giad061; *Cell reports* 2025 https://doi.org/10.1093/gigascience/giad061). Thus, the chr7D inversion is more likely associates with adaptive traits, not the naked phenotype directly.

Minor Comments:

1. The population structure lacks explicit labeling of oat types (Fig. 1a). Consequently, the genetic composition of hulled versus naked oats within the inferred clusters is unclear, it is difficult to interpret whether the observed clustering truly reflects domestication or population structure patterns. Incorporating sample type annotations in the figures would clarify the correspondence between genetic clusters and phenotypic classifications.

RESPONSE: We sincerely thank you for your thorough review. We agree that the point you raised-unclear sample type annotations in the original Fig. 1a-is valid, as this would hinder intuitive interpretation of the genetic distribution patterns of hulled and naked oats. In response to your suggestion, we have added sample type annotations to the relevant panels (Fig. R1 above and Figs. R12-13 below; also Revised Manuscript Figs. S3a, 1a, and 1c). We thank you again for your valuable feedback.

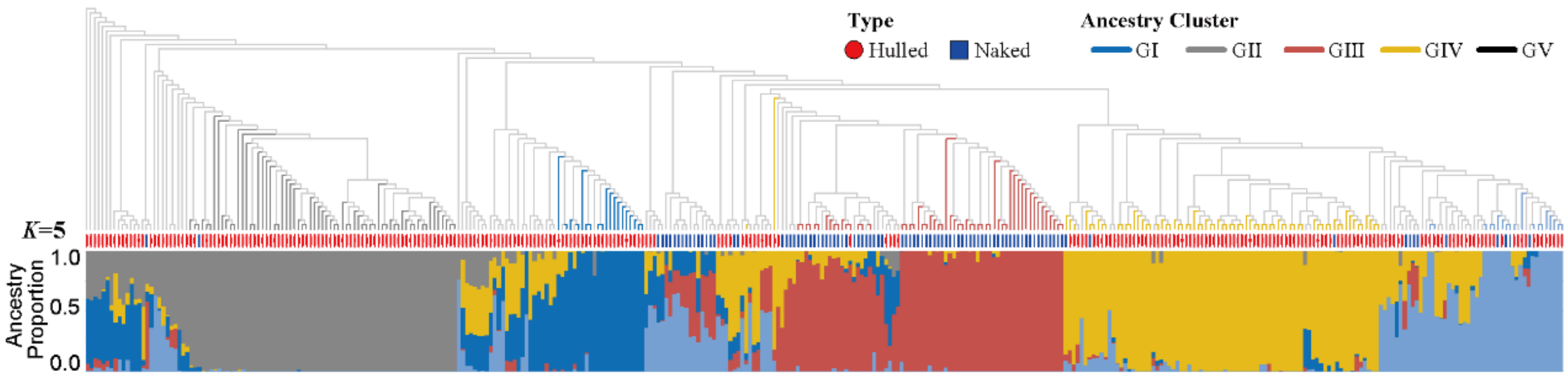

Fig. R12 Genetic analysis of 666 oat accessions using 127,563 high-quality SNP markers, presenting the rooted neighbor-joining tree and population structure Admixture plot at *K*=5 (Revised Manuscript Fig. 1b).

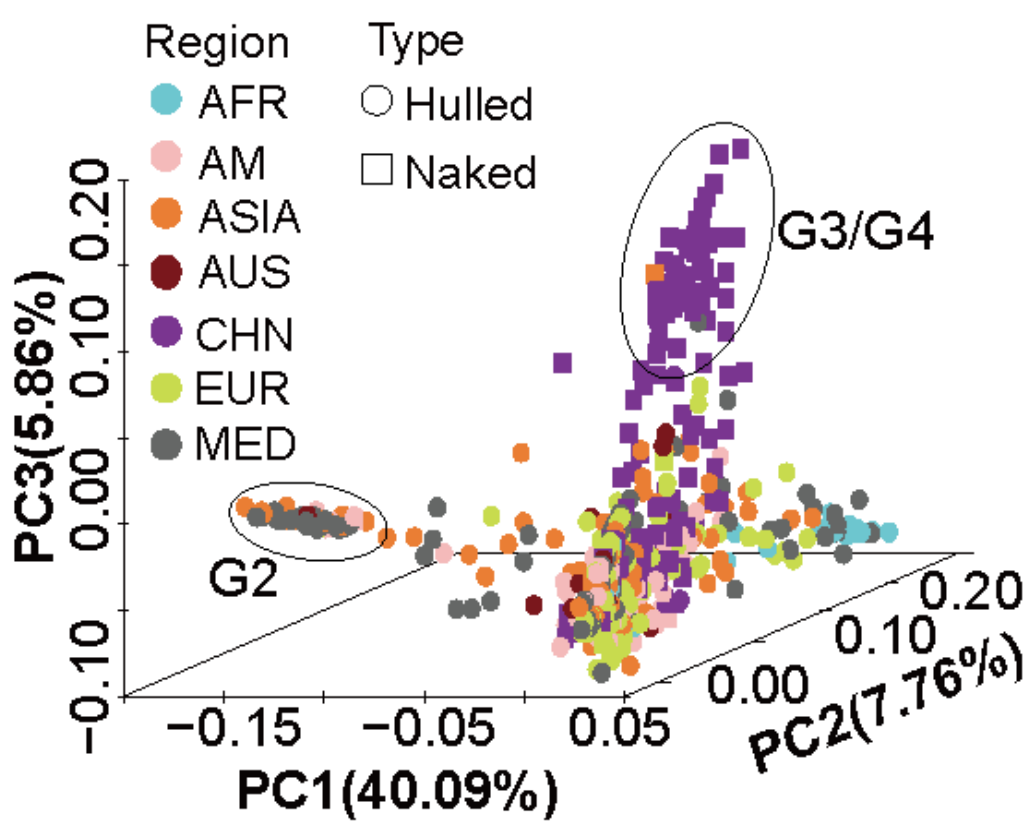

Fig. R13 3D-PCA plot of 666 oat accessions based on 127,563 high-quality SNP markers (Revised Manuscript Fig. 1c).

2. In Fig. 1b, it is unclear why the wild oat groups were not used as an outgroup, but were instead included within the ingroup together with the cultivated oat lineages.

RESPONSE: We sincerely appreciate your helpful suggestion. In response, we have reconstructed the original unrooted tree into a rooted tree using wild hexaploid accessions as the outgroup (Fig. R12 above, also Revised Manuscript Fig. 1b). Unlike an unrooted tree, which only reflects topological relationships among clades, a rooted tree clarifies the shared ancestor and evolutionary direction of the taxa. This modification better aligns with our goal of elucidating the population evolutionary history of oats and renders the results clearer and more intuitive. We thank you again for your insightful guidance.

3. Lines 144-148: Gene flow analysis assumes directional migration but lacks statistical support for migration rates (e.g., Migrate-N or BayesAss). Provide confidence intervals for inferred events. The authors must add statistical support for each migration event (e.g., bootstrap support > 80% or LRT P <0.05).

RESPONSE: We sincerely appreciate your rigorous feedback regarding the reliability of our gene flow analyses, and we fully understand your well-founded request that explicit statistical support be provided for the inferred migration events. To this end, we have refined our gene flow analyses and added robust statistical validation for each inferred migration event, as shown below (Tabs. R2-5 below; also Revised Supplementary Tab. S3-3).

Tab.R2 Key Statistical Metrics for Gene Flow Events Between Hulled and Naked Oats (Revised Supplementary Tab. S3-3a)

DS	RS	Weight	Variance	SE	95% CI	Z-value
HC	NC	0.471098	0.0034869	0.05905	(0.3554,0.5868)	7.98
HC	NL	0.417519	0.001921	0.04383	(0.3316,0.5034)	9.53
NC	HC	0.170872	0.00349	0.05908	(0.0551,0.2867)	2.89
NC	HL	0.07547	0.002169	0.04657	(0,0.1668)	1.62
Wild	NC	0.059186	0.00136	0.03688	(0,0.1315)	1.61

Note: Donor Subpopulation (DS),Recipient Subpopulation (RS)。

Tab.R3 Model Selection Statistics of Treemix Across Varying Migration Event Counts of hulled and Naked oats (Revised Supplementary Tab. S3-3b)

M	LL	LRT	AIC
0	58.0339	-	-116.0678
1	78.233	40.3982	-154.466
2	92.5139	68.96	-181.0278
3	97.6274	79.187	-189.2548
4	98.1004	80.133	-188.2008
5	98.19017	80.31254	-186.38034

Note: Migration events (M)，Log-Likelihood (LL)，LRT (Likelihood Ratio Test Statistic)，AIC (Akaike Information Criterion)

Tab.R4 Key Statistical Metrics for Gene Flow Events Between Different Clustered Subgroups Subpopulations (Revised Supplementary Tab. S3-3c)

DS	RS	Weight	Variance	SE	95% CI	Z-value
G5	G4	0.4516	0.00300255	0.0548	(0.3442,0.5590)	8.24
G2	G5/G6	0.2659	0.00213777	0.0462	(0.1753,0.3565)	5.76
G2	G5	0.1544	0.00204777	0.0453	(0.0657,0.2431)	3.41
G5	G2	0.1423	0.00204777	0.0453	(0.0536,0.2310)	3.15
G3	G4	0.1398	0.00047103	0.0217	(0.0973,0.1823)	6.44
G3	G5	0.11618	0.00016631	0.0129	(0.0909,0.1415)	9
G4	G1	0.10943	0.00053629	0.0232	(0.0640,0.1548)	4.72
G1	G6	0.0883591	0.00013091	0.0114	(0.0660,0.1108)	7.75

Note: Donor Subpopulation (DS),Recipient Subpopulation (RS)。

Tab. R5 Model Selection Statistics of Treemix Across Varying Migration Event Counts Among Different Clustered Subgroups (Revised Supplementary Tab. S3-3d)

M	LL	LRT	AIC
0	63.4371	-	-126.8742
4	112.327	97.7798	-216.654
5	115.2169	103.5596	-220.4338
6	120.1068	113.3394	-228.2136
7	122.406	117.9378	-229.85
8	122.925	118.9758	-230.812

Note: Migration events (M)，Log-Likelihood (LL)，LRT (Likelihood Ratio Test Statistic)，AIC (Akaike Information Criterion)

Reviewer #3 (Remarks to the Author):

In the present study, Dong et al. analyzed 666 hulled and naked oat accessions originating from different countries and representing wild samples, landraces, and bred varieties. Their goal was to address the genetic origin and phenotypic trait divergence between hulled and naked oats by integrating genotypic, phenotypic, and transcriptomic data. They propose that naked oats originated from a key divergence event during the eastward dispersal of Mediterranean hulled oat landraces, followed by independent domestication and diversification in China. Using multi-environment phenotypic data, they showed phenotypic differences between the two types, with hulled oats exhibiting higher yield and larger grain size, whereas naked oats had higher crude protein and fat content. They also incorporated a selection scan during naked oat domestication, confirming a trade-off between genes linked to quality and yield. By combining selection signatures with differential gene expression, they identified three genes associated with previously reported oat grain-yield QTLs.

Major comments:
While Dong et al. present a large and valuable dataset integrating genomic, phenotypic, and transcriptomic information from 666 hulled and naked oat accessions, several aspects of the work suffer from unclear presentation, overly dense descriptions, and insufficient explanation of key analysis.

RESPONSE: We sincerely appreciate your comprehensive and insightful comments. The issues you raised-including unclear presentation, verbose content, and insufficient elaboration of key analytical methods-have offered critical guidance to enhance the clarity, focus, and methodological rigor of the manuscript. In this revision, we have systematically streamlined and refined the manuscript by focusing on our primary objective: elucidating the genetic origin of naked oats and the phenotypic and genomic divergence mechanisms between naked and hulled oats. First, We have strengthened the manuscript's logical thread, reorganized it into a progressive three-module framework (population genetic structure and domestication history analysis, genomic basis dissection of phenotypic differentiation, core gene identification via integrated GWAS and gene expression analysis), and streamlined the full text to focus strictly on core results. The Discussion section is centered on the genetic divergence patterns between naked and hulled oats, the genetic basis of nutritional-yield trait trade-offs, and core gene set identification, ensuring a coherent scientific narrative and highlighting our key findings. Second, we have expanded and clarified the details of key analytical methods, supplementing the Materials and Methods section with detailed statistical rationale, parameter configurations, and validation protocols for population analysis, selection sweep analysis, and gene flow testing, to guarantee the transparency and reproducibility of analytical processes. All targeted revisions, supplementary data, and analytical details have been integrated into the revised manuscript and are highlighted through section references, updated figures and tables. We believe this revision has significantly enhanced the manuscript's rigor and readability. We sincerely thank you again for your invaluable guidance and kindly request your further review of the revision.

A major issue is the lack of a clear, logical sequence in the population genetics and domestication sections. The authors report numerous subgroups, divergence events, and geographic transitions, but do not clearly explain how these analyses support their

proposed evolutionary scenario. Although they argue that naked oats originated from hulled landraces during an eastward Mediterranean dispersal, the genomic evidence underlying this conclusion is only briefly referenced. As a result, the domestication narrative remains difficult to follow, and the claim of independent domestication in China is not convincingly supported.

RESPONSE: We thank the reviewer for pointing out the issues in our population genetics and domestication sections, including unclear logical flow, weak connections between results and evolutionary hypotheses, and insufficient support for the conclusion regarding independent domestication of naked oat in China. These suggestions are constructive, and we have fully revised the relevant sections accordingly. We have also added robust genetic evidence to clarify the domestication process of naked oat. The revised content is presented below (Fig. R1 below; also Revised Manuscript Fig. 2 and; L158-211):

"To minimize confounding gene flow from modern breeding, we selected 366 landraces with conserved ancient genetic backgrounds for gene flow and population evolutionary analyses. First, phylogenetic reconstruction of 366 landraces reveals hulled oats dominate transcontinental clusters with continuous distribution, comprising the Northeast African and West Asian group (GI: 45 accessions), the Mediterranean Basin group (GII: 77 accessions), and the Central and Eastern Eurasian group (GIII: 110 accessions). In contrast, over 81% of Chinese naked oats segregate into isolated clades GIV. The GV subpopulation consists of 63 admixed accessions of hulled and naked oats from multiple geographic origins (Figs. 2a-c and S8a, b; Tab. S4). Notably, although most Chinese naked oats formed a single subpopulation, 26.8% of accessions showed genetic admixture with other subpopulations and geographically diverse hulled oats (Tab. S4). Further gene flow analysis revealed 7.5%-16.8% shared haplotypes across subpopulations and significant multidirectional introgression in most pairs ($D > 0.45$, $Z > 9$, $P < 0.001$), indicating sustained cross-regional gene flow between hulled and naked oat landraces even after excluding cultivars, and supporting an ancient evolutionary origin for their genetic exchange (Fig. 2g-j; Tab. S4). Gene flow analyses also delineate the evolutionary succession: first from wild hexaploid oats to hulled oat landraces, followed by naked oat landraces from hulled oat landrace and finally hulled and naked cultivars derived from their respective landraces (Fig. S6e), Therefore, the genetic admixture between hulled and naked oats is the result of both shared ancestral polymorphism and ancient gene flow, rather than being a product of recent breeding alone.

Genetic differentiation analysis identifies the Mediterranean Basin group (GII) as the most divergent ($F_{st} = 0.18$-0.31) and genetically diverse ($\pi = 2.75 \times 10^{-6}$) subpopulation, suggesting it is an ancestral diversity center retaining ancient landrace variation (Fig. 2d-e; Tab. S4-2). In contrast, extensive gene flow ($D = 0.45$, $Z = 9.3$, $P < 0.001$), minimal differentiation ($F_{st} = 0.09$-0.12), and the highest proportion of shared long ROH (1-3 Mb; 44.7%) occur between the Central and Eastern Eurasian group (GIII) and Chinese naked oats (GIV), positioning GIII as the most direct genetic source for GIV (Fig. 2f-h; Tab. S4-2). Phylogenetic analysis based on D_{xy} quantifies these relationships: GIV shows the smallest D_{xy} with GIII (0.231), confirming their close affinity, whereas GII exhibits the largest D_{xy} with all other groups (0.279-0.305), consistent with its early divergence (Fig. 2i). Demographic modeling with fastsimcoal2 further supported these evolutionary relationships. The no-migration model was rejected across all pairwise comparisons among GII, GIII, and GIV, verifying extensive

post-divergence gene flow. The optimal three-population model (Model 9, AIC = 293574.92) identified GIV as a recent derivative of the GIII lineage. It diverged from the GIII ancestral pool ~6124 generations ago and initiated secondary population contact ~4940 generations ago, after an early isolation period of 1184 generations. During early divergence, GIV experienced an extreme founder bottleneck (Ne ≈ 187), with strong genetic drift accelerating the fixation of unique traits. GIV initiated recent population expansion at 1772 generations ago, earlier than GIII (1572 generations ago), consistent with the rapid adaptation and diffusion typical of derived lineages post-domestication. Furthermore, GII's *Ne* (969) was markedly lower than the model-predicted value (1,860), while GIII's *Ne* (3,625) exceeded expectations (1,572). GIV's *Ne* (1,338) closely matched the predicted value (1,772). These patterns collectively support GII as the ancestral lineage and GIII as the most direct genetic source of GIV (Fig. 2j; Tab. S4-3). Collectively, synthesizing population structure and diversity patterns, we propose the primary domestication is hulled oats from wild species in the Mediterranean Basin region. The dual dispersal are southeastward to East Africa and Eastward to Central and Eastern Eurasia/China. During this eastward dispersal, naked oats diverged from hulled oat lineages, followed by secondary domestication in North China (highest π in naked oats) (Fig. S8c-d)."

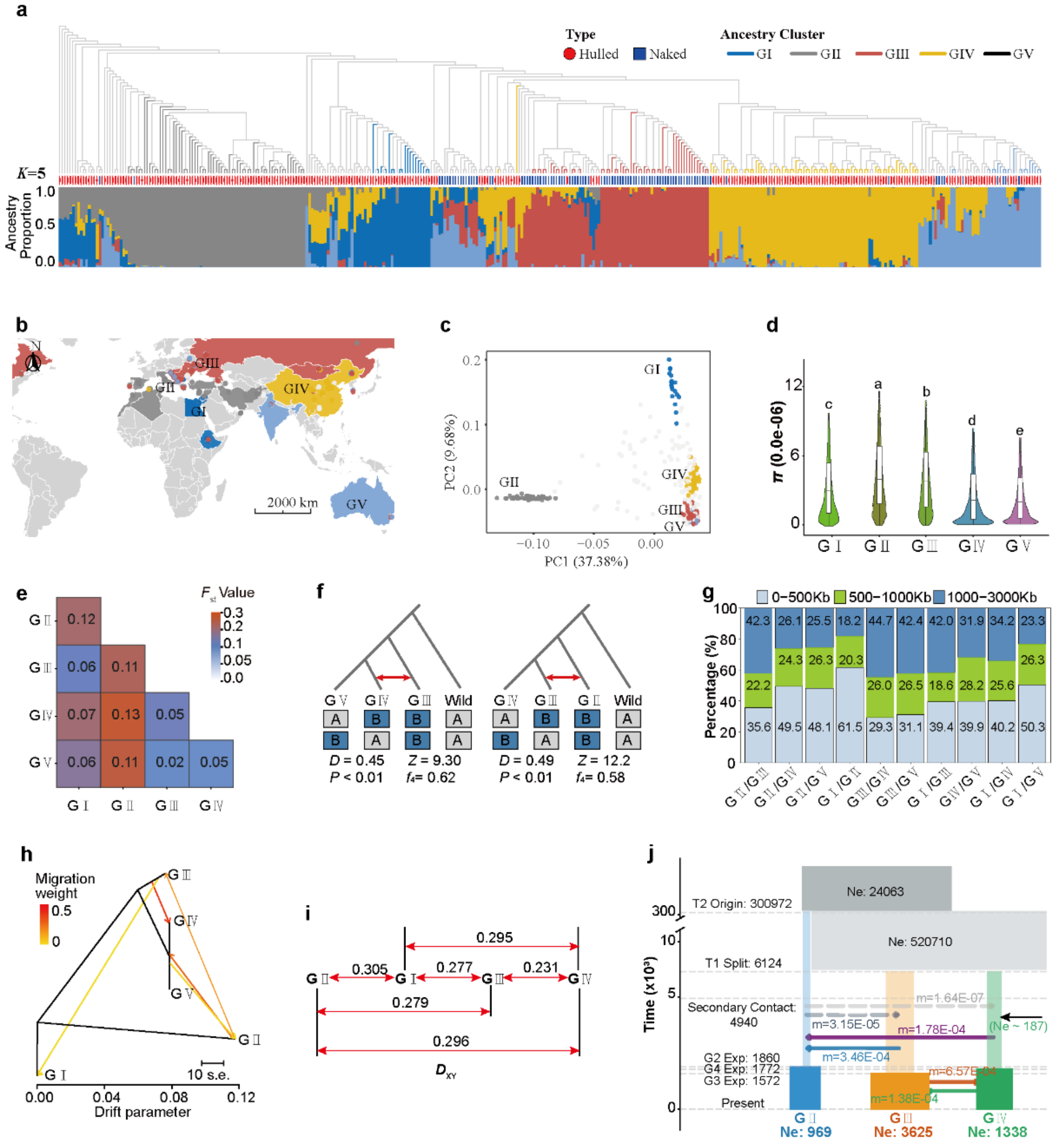

Fig. R1 Analysis of the domestication pathway of naked oats based on 366 landrace accessions

(Revised Manuscrip Fig. 2). (a) Combined plot of Admixture analysis ($K = 5$) and neighbor-joining (NJ) phylogenetic tree (rooted with wild accessions) for 366 landrace accessions; (b) Global distribution of accessions in each subgroup; (c) Population clustering of 366 landrace accessions based on the first two principal components (PC1 and PC2); (d) Nucleotide diversity levels among different subgroups ($P < 0.05$); (e) Fixation index (F_{st}) values among different subgroups; (f) ABBA-BABA test revealing significant genetic flow between GII and GIII-GIV, and between GIII and GIV-GV subgroups; (g) Proportions of shared homozygous haplotypes of different lengths (10 kb-3 Mb) among subgroups; (h) Phylogenetic topology and genetic flow direction among clustered subgroups with five fitted migration events; (i) D_{xy}-based phylogenetic tree showing genetic diversity differentiation among GI-GIV subgroups; (j) Demographic history reconstruction of GII, GIII, and GIV using fastsimcoal2.

The presentation of figures further complicates interpretation. Several figures and corresponding legends lack critical information, making it difficult to understand how the data support the authors' claims.

RESPONSE: We have revised all figures and tables in line with your comments, enhancing the legends with essential details such as data descriptions, statistical methods, and sample sizes, and refining all annotations. These improvements have substantially increased the clarity and readability of the visual presentation, allowing the figures and tables to more effectively support the manuscript's conclusions. We are grateful for your valuable suggestions. The updated versions are now included in the main text and supplementary materials of the revised manuscript.

The selection-scan analyses are similarly problematic. The rationale for the specific methods, thresholds, or integration strategy is not explained. Without justification, it is unclear why these statistics were chosen or how the candidate regions were identified, weakening confidence in the reported selection signals.

RESPONSE: We thank the reviewer for the insightful comments concerning the rigor of the selection sweep analysis and the coherence of its presentation. In response to the points raised about the need for clearer justification of the methods, thresholds, and integration strategy, we have systematically addressed these aspects in the revised manuscript: For the genome-wide selection scan, we first extracted and reformatted VCF subfiles for 504 hulled oats and 162 naked oats using BCFtools (*Bioinformatics* 2016 https://doi.org/10.1093/bioinformatics/btw044) leveraging the high-quality VCF file of all tested accessions. Subsequently, we calculated the nucleotide diversity (π) and fixation index (F_{st}) across the genomes of these two groups using VCFtools (*Bioinformatics* 2011 https://doi.org/10.1093/bioinformatics/btr330) with a 500 kb window and 50 kb step size (Revised Manuscript L609-620). Based on these analysis results, we established screening thresholds for putative selected loci in naked oats: the core threshold was set as a genome-wide LogF_{st} (differentiation index) > 2.7 ($P < 0.001$) between hulled and naked oats. Additionally, we calculated the nucleotide diversity ratio (π) at the same genomic loci between the two groups and combined these results with XP-CLR analysis outcomes. For both analyses, the top 1% of loci ($\pi > 59.07$) were selected as auxiliary screening thresholds using the percentile method. This threshold-setting approach is consistent with standard practices in crop population genomics research (*Nature genetics* 2012 https://doi.org/10.1038/ng.2309; *The Plant cell* 2023 https://doi.org/10.1093/plcell/koad229), and supported by robust methodological rationale. To mitigate false positives potentially caused by complex population structure

and gene flow, we implemented a multi-faceted validation strategy integrating QTL co-localization (LD half-decay distance = 2 Mb), intersections of transcriptome-derived differentially expressed genes, and haplotype-phenotype association analysis. This strategy allowed us to focus on core candidate genes and effectively eliminate false positive signals (Revised Manuscript L283-294), thereby enhancing the reliability of the 1504 candidate selected genes.

Transcriptomic analyses also lack critical clarity. The manuscript does not clearly define which comparisons were performed (e.g., hulled vs. naked, tissues, or subgroups). Instead, the results list numbers of DEGs and enriched pathways without connecting them to the experimental design or explaining their biological relevance. This makes it difficult to interpret how expression differences relate to domestication or adaptive divergence.

RESPONSE: We sincerely appreciate your identifying the lack of clarity in our transcriptome analysis. We fully concur with your feedback that we should clearly define comparison groups, strengthen the link between results and experimental design, and elaborate on biological significance, all of which are critical to enhancing analytical logical coherence and conclusion persuasiveness. In the revised manuscript, we have systematically addressed these issues.

1)We clearly established a core comparative framework, designating 9 geographically diverse hulled oat accessions as the control group and 13 genetically distinct naked oat accessions as the test group. From mixed root, stem, leaf, and panicle tissue transcriptome data, we identified differentially expressed genes (DEGs) using edgeR (*Bioinformatics* 2009 https://doi.org/10.1093/bioinformatics/btp616) with unified filtering criteria: FDR-adjusted *P*-value < 0.05, $|\log_2\text{FoldChange}| > 1.5$, and TPM > 1.To further elucidate specific biological processes, we added targeted analyses of seed development, saline-alkali stress, and drought response. For these, we used blank samples or early developmental stage samples as controls. After identifying DEGs under each condition, we compared intersections of DEGs between hulled and naked oat data and excluded genes with consistent expression trends in both groups. This ultimately yielded gene sets specifically expressed in either hulled or naked oats, thus ensuring the specificity of all subsequent analyses. The detailed revisions are as follows: [L578-597]

“We integrated 16 public transcriptome datasets from NCBI and 22 in-house mixed-sample datasets. Public datasets included 6 seed development datasets (4 hulled, 3 naked oats), 7 saline-alkali stress datasets (3 naked, 4 hulled oats), and 2 drought stress datasets (naked oats). All datasets underwent quality control (QC) using FASTQ (*iMeta* 2003 https://doi.org/10.1002/imt2.107), ensuring normal distribution of sequencing quality scores (average Q30 ≥80%, error rate ≤0.1%) and GC content, plus removal of adapter-contaminated reads. Post-QC clean reads were aligned to the hexaploid oat reference genome "Sanfensan" using HISAT2 (*Nature Biotechnology* 2019 https://doi.org/10.1038/s41587-019-0201-4) with default parameters. Differentially expressed genes (DEGs) were screened via edgeR (*Bioinformatics* 2009 https://doi.org/10.1093/bioinformatics/btp616) with FDR-corrected p-value < 0.05 and |log2Fold Change| > 1.5. Only genes with TPM > 1 (used for expression quantification) were considered for DEG selection. The identification pipeline for DEG sets across all projects was as follows: for bulk RNA-seq data, hulled oats were designated as the

control group and naked oats as the treatment group to identify DEGs between the two types. For single-project RNA-seq data, blank controls or early developmental stages were used as references, with DEGs first identified for all other developmental stages and treatment conditions. Subsequently, for the same project, DEGs from multiple single-project datasets of hulled oats and naked oats were intersected separately; genes with consistent differential expression (e.g., both upregulated or downregulated during seed development) in both oat types were then excluded. Ultimately, the hulled oat-specific or naked oat-specific DEG sets were obtained for subsequent analyses."

2) We performed separate GO and KEGG enrichment analyses on DEGs from both mixed-tissue and targeted scenario datasets. We adopted a strategy of intersecting significantly enriched pathways across individual datasets and conducting joint validation across multiple datasets to screen for core biological pathways. Most importantly, we pinpointed 117 core associated genes through a cross-validation approach that integrates selected genes, DEGs, and QTL co-localization. This approach directly links enriched pathways, including those related to nutrient accumulation and stress response, to the phenotypic differences between hulled and naked oats in nutrient accumulation, yield performance, and abiotic stress response. It also clarifies the intrinsic relationship between gene expression variation, domestication divergence, and adaptive trait formation, avoids isolated data presentation, and significantly enhances the biological significance of our results. The detailed revisions are as follows: [L332-355]

"To identify key genes underlying hulled-naked oat differentiation, we integrated 1,504 candidate selected genes in naked oats with multi-context transcriptome data to construct a combined selection signal-expression association profile. Our results revealed that during core physiological processes, we identified 39 selected genes exhibiting differential expression between hulled and naked oats, comprising 31 upregulated and 8 downregulated genes (e.g., *UMAMIT25*, *AHL1, SAG12*, *ygl8*, *WOX13*). Among these, 22 genes under directional selection and upregulated expression were significantly enriched in pathways such as lipid metabolism and post-translational protein modification (Fig. 5a; Tab. S7-6). During the seed development stage, a total of 31 selected genes were identified. Among them, upregulated genes (e.g., *PARP1*, *RAB28*, *PP2C30*, *HSP18.2*) may be involved in embryonic cell division and differentiation and ABA signal response, whereas genes under balancing selection and downregulated expression (e.g., *MPK3*, *CycB1;5*, *RSW3*) were enriched in cell cycle regulation pathways ($q$value < 0.05) (Fig. 5b; Tab. S7-6). In contrast, the 32 differentially expressed genes related to seed development in hulled oats exhibited a pattern of cooperatively upregulating energy metabolism and signal transduction while downregulating redundant hormone pathways (Fig. 5c; Tab. S7-6). Furthermore, in response to abiotic stress, 25 selected genes specifically expressed in naked oats (e.g., *PP2C09*, *FEI1*, *PME18*, *PIP3I*) generally displayed signatures of directional selection and upregulated expression, suggesting their potential functions in stress adaptation (Fig. 5c-f; Tab. S7-6). Further analysis revealed that 33 of the aforementioned 127 associated genes co-localized with published QTLs for oat yield and quality traits (Fig. 5; Tab. S7-5, highlighted in red), significantly enhancing the credibility of these candidate genes in trait differentiation."

Overall, the manuscript presents a rich dataset with potential insights into oat domestication, but the narrative is difficult to follow due to unclear explanations, insufficient justification of methods, and a focus on listing results rather than discussing them. I believe a more structured presentation with clear rationale for analytical choices and better integration of results would be essential for its publication.

RESPONSE: We sincerely appreciate your thorough review and constructive comments. The issues you pointed out, including unclear logical reasoning, insufficient explanation of analytical methods, and excessive presentation of separate results without integrated discussion, clearly identify the main weaknesses of the original manuscript and provide valuable guidance for improving its quality. We have carefully followed your suggestions and revised the manuscript systematically. We restructured the entire logical framework based on germplasm characteristics, population structure analysis, and domestication pathway interpretation. We clarified the relationships among genomic selection, differential expression, phenotypic differentiation, gene identification, and breeding application to improve narrative coherence and practical value. We added complete justification for key methods including population structure, gene flow, selective sweep detection and transcriptome analysis, with clear explanation of their appropriateness and rationality of parameter settings. We strengthened result integration and in-depth discussion by establishing internal connections among multi-dimensional evidence and interpreting the biological significance of our findings in combination with previous studies on oat domestication. The revised manuscript is logically consistent, clearly structured and scientifically rigorous, with all major limitations effectively addressed.

Line-by-line comments:
L82 I would write the acronyms the first time you mention it.

RESPONSE: We thank the reviewer for noting the need for greater rigor in the manuscript's presentation. As requested, we have now ensured that all abbreviations (including "SNPs" and "SVs" at the original Line 82 and others in the Results) are spelled out in full at first use. We appreciate this suggestion, which has enhanced the clarity and professionalism of the text.

L163-164 Fig. 3b is not properly explained and misses axis labels.

RESPONSE: We sincerely appreciate your meticulous feedback. We have fully revised Fig. 3b with the following improvements to address the noted issues: first, we corrected the mislabeling of spike type (ST) and spikelet type (SLT) and standardized all labels in line with academic norms; second, we reformatted the figure as a stacked chart (Fig. R2 below; also Revised Manuscript Fig. 3b), which more clearly illustrates the distribution patterns in spike type, spikelet type, and awn trait between hulled and naked oats; third, we added complete x- and y-axis labels, ensuring the figure’s information is accurate and clear.

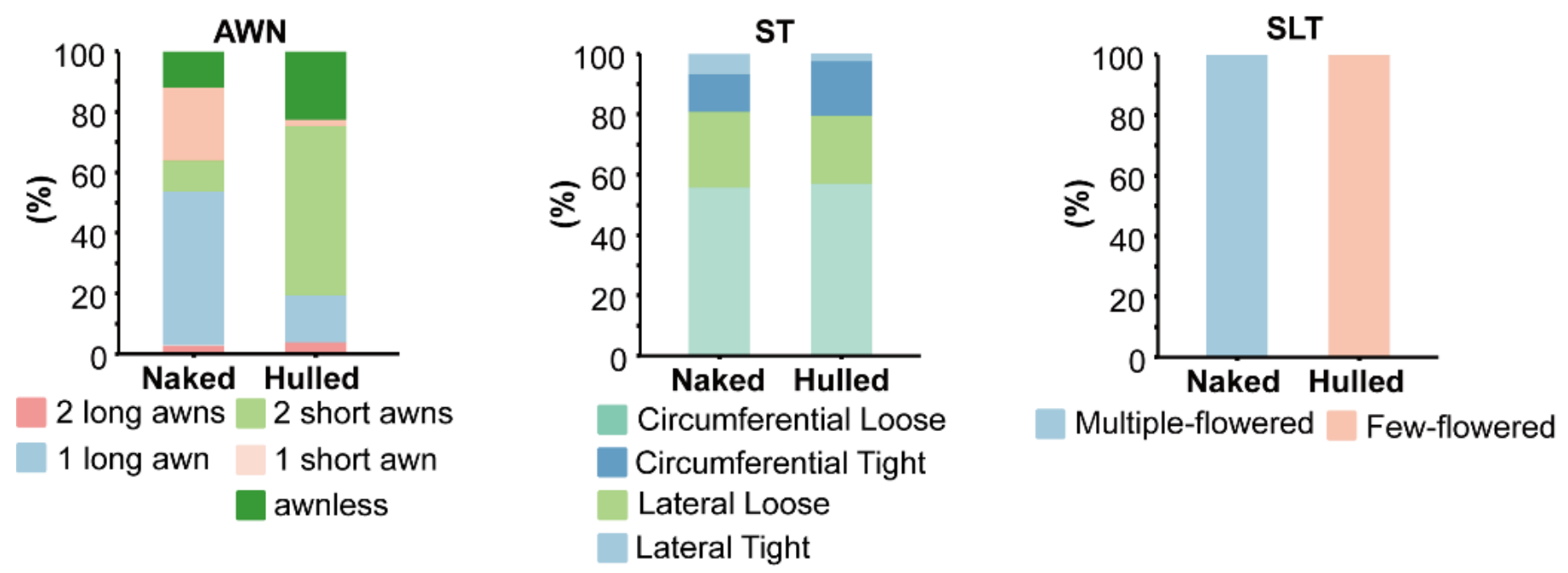

Fig. R2 Proportion of accessions with different awn types, panicle types and spikelet morphologies in hulled and naked oats (Revised Manuscript Fig. 3b).

L170-172 Fig. 3c missies axis labels and it is not clear how the scale on the Y axis could refer to different units, e.g GCI, mm CFC & CPC, %, etc.

RESPONSE: We sincerely appreciate your identifying the inadequacies and errors in our figure presentation. In response to these issues, we first revised the overall figure presentation in the "Phenotypic divergence analysis" section of the revised manuscript: we separately presented phenotypic differences for each trait between hulled and naked oats (Fig. R3 below; also Revised Manuscript Fig. 3c) and undertook a thorough recheck and correction of the units for all relevant metrics.

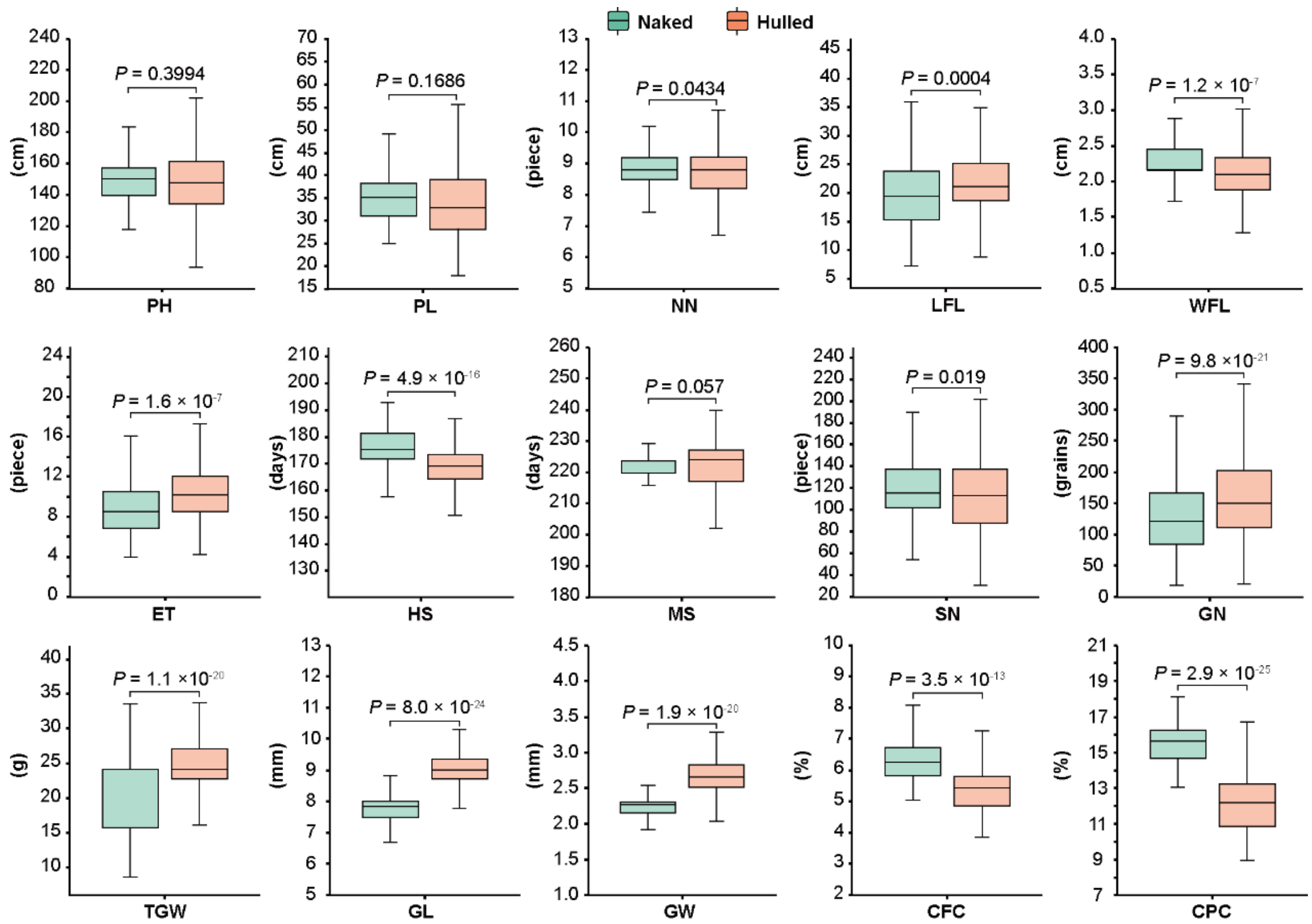

Fig. R3 Box plots showing phenotypic differences between hulled and naked oats for 8 growth-related traits (PH, PL, NN, LFL, WFL, ET, HS, MS), 5 grain-yield traits (SN, GN, TGW, GL, GW), and 2 grain-quality traits (CFC, CPC) (Revised manuscrip Fig. 3c).

L178 I would avoid including the table as a figure, also it does not help supporting the claims in the text.

RESPONSE: We sincerely appreciate your valuable suggestion, which we have adopted. We have thus removed the original figure and replaced it with a more reader-friendly eigenvector table (Table R1 below; also Revised Supplementary Tab. S5-4).

Table R1 Top 10 principal component scores of PCA based on 19 morphological markers (Revised supplementary Tab. S5-4)

Trieats	PC1	PC2	PC3	PC4	PC5	PC6	PC7	PC8	PC9	PC10
GW	0.886	-0.406	0.145	0.066	0.028	-0.05	-0.057	-0.041	0.038	0.012
GC	0.884	-0.407	0.158	0.069	0.033	-0.051	-0.063	-0.051	0.042	0.006
GL	0.882	-0.407	0.165	0.068	0.036	-0.05	-0.065	-0.053	0.042	0.006
CFC	0.864	-0.362	0.144	0.082	0.007	-0.048	-0.022	-0.06	0.031	0.002
PH	0.312	0.703	0.12	0.099	0.205	-0.305	-0.064	-0.179	0.034	-0.076
SL	0.241	0.67	-0.032	0.306	0.215	-0.405	-0.046	-0.201	-0.007	-0.058
HS	0.526	0.629	0.038	-0.362	-0.114	0.244	-0.052	0.034	0.034	-0.245
NWL	0.352	0.616	0.156	0.337	-0.169	0.208	0.035	0.251	-0.104	0.044
FLW	0.518	0.589	0.036	-0.213	-0.162	0.354	-0.098	0.109	-0.069	-0.248
TKW	-0.127	-0.485	0.349	0.101	-0.034	0.173	-0.377	0.417	-0.074	-0.02
FLL	0.284	0.449	0.126	-0.443	0.044	0.232	0.129	0.117	-0.005	0.104
MS	-0.21	0.094	0.728	0.14	-0.322	-0.238	0.22	0.204	0.035	0.024
H/HL	0.373	-0.044	-0.72	-0.059	-0.217	-0.092	0.095	0.253	0.266	0.11
ST	0.363	-0.018	-0.628	0.013	-0.276	-0.221	0.48	0.063	0.28	-0.069
ET	-0.018	0.063	0.581	-0.263	-0.326	-0.206	0.047	0.121	-0.174	0.2
NSS	0.126	0.372	-0.052	0.748	-0.185	0.1	0.624	0.08	-0.51	0.045
PT	0.042	0.275	0.105	-0.178	0.583	-0.273	-0.066	0.607	0.15	0.041
CPC	0.326	-0.149	0.038	-0.121	0.363	0.137	0.285	-0.054	0.568	-0.053
AWN	-0.092	-0.037	0.053	0.429	0.343	0.507	-0.108	-0.139	0.107	0.628
CE rate%	23.47	41.278	52.185	60.086	65.98	71.743	76.827	81.395	85.746	88.919

L189-190 I think it would be more appropriate talking about nucleotide diversity, not genetic divergence.

RESPONSE: We sincerely appreciate your revision suggestions for this key content. Describing this with nucleotide diversity is indeed more accurate than the term "genetic differentiation", and we have made the corresponding revisions.

L193 Here it would be appropriate to calculate the loss of diversity statistic (Vigouroux et al. 2002).

RESPONSE: We sincerely appreciate your suggestions for revising the comparison metrics of genetic diversity divergence between hulled and naked oats at the chromosomal level. Genetic diversity loss is a metric that quantifies the reduction in genetic diversity among related populations due to domestication, breeding selection, or genetic bottlenecks, and serves as a key basis for assessing chromosomal divergence between hulled and naked oats. In the revised manuscript, we used the classic calculation method from Vigouroux (*PNAs* 2002 https://doi.org/10.1073/pnas.112324299) to quantify genetic diversity loss in naked oats relative to hulled oats for each of the 21 chromosomes and at the genome-wide level (Table R2 below, Revised supplementary Tab. S2-7). We also revised the relevant descriptions and analyses in the results section of the revised manuscript. The detailed revisions are as follows: [L261-270]

"Building upon established population structure and phenotypic differentiation between hulled and naked oats, we quantified chromosomal-level genetic divergence during oat domestication using Tajima's D and nucleotide diversity (π). The results showed that except for chromosomes 2D, 3A, 6D, and 7C (π differences , $P > 0.05$; Tajima's $D \geq 0$ spanning >83%), the remaining 17 chromosomes, naked oats showed significantly reduced π (loss rate: 37.75%–73.92%, $P < 0.001$) and elevated selection pressure ($D < 0$; 29-fold increase) relative to hulled oats(Fig. S11; Tab. S6), indicating that naked oats underwent a stronger genetic bottleneck and more intensive artificial selection during domestication, thereby laying the genomic foundation for their distinct phenotypic differentiation."

Tab. R2 Average genetic diversity loss rate in naked oats compared to hulled oats across 21 chromosomes

Chrom	Nucleotide diversity (Hulled)	Nucleotide diversity	Diversity loss
chr1A	1.7E-06	1.1E-06	39.2907
chr1C	2.9E-06	1.5E-06	47.2927
chr1D	2.8E-06	1.1E-06	60.1188
chr2A	2E-06	1.1E-06	43.454
chr2C	3.1E-06	8.1E-07	73.9156
chr2D	2.6E-06	2.3E-06	10.2431
chr3A	2.5E-06	2.1E-06	13.5087
chr3C	1.9E-06	6.3E-07	66.1573
chr3D	2.4E-06	6.6E-07	72.5649
chr4A	1.9E-06	8.3E-07	56.0321
chr4C	2.5E-06	1.2E-06	51.9979
chr4D	1.8E-06	1.1E-06	40.1063
chr5A	2.7E-06	1.6E-06	40.4531
chr5C	2.1E-06	1.2E-06	44.6685
chr5D	2.7E-06	1.7E-06	37.7533
chr6A	2.7E-06	1.4E-06	48.2882
chr6C	2.5E-06	1.3E-06	49.5634
chr6D	2.3E-06	2.2E-06	4.11625
chr7A	2.3E-06	1.2E-06	46.1589
chr7C	3.1E-06	3.1E-06	-3.0862
chr7D	3.1E-06	1.6E-06	47.4614

L195-196 This approach to detect genes under selection should be much better explained.

RESPONSE: We sincerely appreciate your suggestion that we elaborate on the research methods for the key results section of the manuscript. For the identification of selected genes, our detailed methodologies are provided as follows: [L650-663]

"The cross-population composite likelihood ratio test (XP-CLR) (*Genome research* 2010 https://doi.org/10.1101/gr.100545.109) quantifies genomic allele frequency differentiation between target and reference populations to detect selection signals by fitting a skewed model of population allele frequencies. In this study, hulled oats served as the reference and naked oats as the target population for XP-CLR score calculation, with parameters set to -window 500 kb, -step 50 kb, -minsnps 20, and others default. Meanwhile, BCFtools (*Bioinformatics* 2016 https://doi.org/10.1093/bioinformatics/btw044) extracted and reformatted VCF subfiles

for 504 hulled and 162 naked oat accessions from the high-quality VCF file; VCFtools (*Bioinformatics* 2011 https://doi.org/10.1093/bioinformatics/btr330) (500 kb window, 50 kb step) then calculated their nucleotide diversity (π) and fixation index (F_{st}). Based on these results, potential selection loci were the top 1% of XP-CLR scores and π ratio (π_{hulled}/π_{naked}) via the percentile method; for F_{st}, loci with LogF_{st} >2.7 (P<0.001) were the threshold. Subsequently, candidate genes were defined as those within 2 Mb upstream/downstream of each potential selection locus (LD half-decay distance: 2 Mb). Finally, genes detected by at least two methods were identified as the naked oat selected gene set for subsequent analysis."

L198 This interpretation of Tajima's D is oversimplified and not strictly correct. Tajima's D detects whether a population's allele frequency spectrum deviates from the neutral model, but it doesn't specify which evolutionary force caused the deviation.

RESPONSE: We sincerely thank you for your rigorous feedback! We employed a multi-criteria joint validation approach integrating XP-CLR, F_{st} values, and π ratios, which has eliminated interference from confounding factors such as single-indicator bias, genetic drift, and gene flow-thus ensuring the reliability of selected loci. On this basis, we used Tajima's *D* statistic combined with nucleotide diversity (π) to infer selection modes (Fig. R4 below; also Revised Manuscript Fig. S12). The core logic is: Tajima's *D* reflects the degree to which allele frequencies deviate from neutral evolution ($D < 0$ indicates enrichment of low-frequency alleles, and $D \geq 0$ indicates enrichment of intermediate-frequency alleles), while π quantifies population polymorphism levels. If a genomic region meets three criteria simultaneously: naked oats have Tajima's $D < 0$ and significantly lower π relative to hulled oats (the average π reduction rate across the 17 chromosomes is 37.75% to 73.92%), and hulled oats have Tajima's $D > 0$ at the same locus (with enrichment of intermediate-frequency alleles and high polymorphism), we can infer, based on crop domestication principles, that directional selection occurred in this region during naked oat domestication and improvement-characterized by enrichment of advantageous alleles and loss of overall polymorphism.

We fully agree with your comment. Tajima's *D* values are influenced by population demographic history and other evolutionary factors. When combined with π values, they are more appropriate for inferring directional selection and balancing selection, rather than directly defining positive or negative selection. The interpretation of this metric in the original manuscript was therefore limited. To improve the rigor of our presentation, we have revised the relevant terminology in the revised manuscript by replacing "positive/negative selection" with the more objective "tendency toward directional or balancing selection", and have reorganized the logical flow of the corresponding results section. The detailed revisions are as follows: [L261-275]

"Building upon established population structure and phenotypic differentiation between hulled and naked oats, we quantified chromosomal-level genetic divergence during oat domestication using Tajima's *D* and nucleotide diversity (π). The results showed that except for chromosomes 2D, 3A, 6D, and 7C (π differences , $P > 0.05$; Tajima's $D \geq 0$ spanning >83%), the remaining 17 chromosomes, naked oats showed significantly reduced π (loss rate: 37.75%–73.92%, $P < 0.001$) and elevated selection pressure ($D < 0$; 29-fold increase) relative to hulled oats(Fig. S11; Tab. S6), indicating that naked oats underwent a stronger genetic bottleneck and more intensive artificial selection during domestication, thereby laying the genomic foundation for their distinct phenotypic differentiation. Integrating the population differentiation index (F_{st}), π ratio

(hulled/naked), and XP-CLR, We screened for potential selective sweeps in naked oats （LogF_{st} > 2.80, P < 0.001, and the top 1% of windows identified by π ratio and XP-CLR analyses）we identified 1,504 putative selected genes (Fig. 4a; Tab. S6). Among these, 883 exhibited directional selection (D < 0), and 621 tended toward balancing selection ($D \geq 0$) (Fig. S12, Tab. S6-4).”

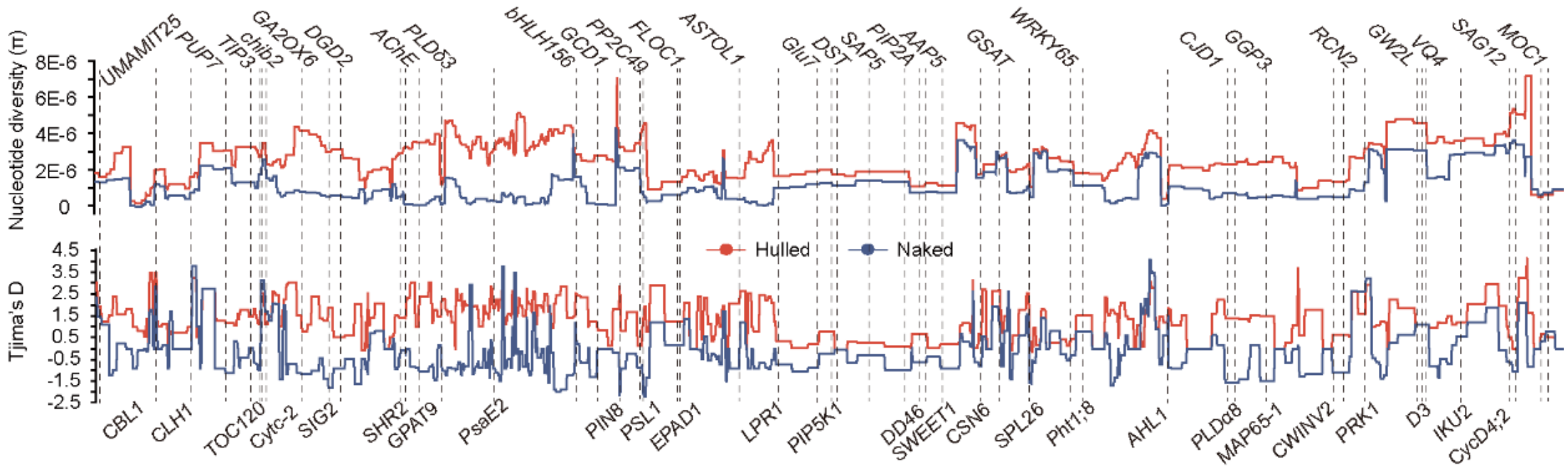

Fig. R4 Scatter line plot of nucleotide diversity (π) and Tajima's D neutrality test values in genomic regions harboring 1504 selected genes between hulled and naked oats (Revised Manuscrip Fig. S12).

L204 The p-values or p.adjust showed in the Fig. S9 and supplementary tables are not significant. Have any normalizations been applied that were not reported?

RESPONSE: We sincerely apologize for the confusion caused by P-value statistics in selected gene enrichment analysis and greatly appreciate your meticulous correction. We fully agree with the conclusion that the adjusted P-values (p.adjust) from KEGG enrichment analysis of downregulated genes in Supplementary Fig. S9 and supplementary tables do not meet the statistical significance threshold. The KEGG enrichment analysis was conducted strictly following the default pipeline and parameters of clusterProfiler software (*A Journal of Integrative Biology* 2012 https://doi.org/10.1089/omi.2011.0118), with no unreported additional standardization. This is common in non-model crop complex trait studies, mainly due to inherent limitations in crop KEGG pathway annotation and the scattered distribution of complex trait-associated differentially expressed genes across multiple pathways; similar observations have been reported in wheat (*The Plant cell* 2024 https://doi.org/10.1093/plcell/koad286; *BMC plant biology* 2015 https://doi.org/10.1186/s12870-015-0511-8). To ensure conclusion rigor and reliability, we systematically revised relevant analyses in the revised manuscript: first, we clearly labeled non-statistically significant enrichment results as the top 20 pathways ranked by statistical P-value; second, we refocused our analysis by integrating multi-tissue mixed transcriptomes with specialized datasets on seed development, salt-alkali stress, and drought stress, only elaborating on GO/KEGG pathways consistently and significantly enriched across all datasets while enhancing individual transcriptomic project enrichment analyses to emphasize statistically significant core findings (Fig. R5 below, also Revised Manuscript Fig. S15). The detailed revisions are as follows: [L307-355]

“To investigate transcriptional regulatory divergence between hulled and naked oats, we conducted RNA-seq analysis on pooled root, stem, leaf, and spike tissues from 13 naked oat and 9 hulled oat accessions with geographically and genetically diverse backgrounds, and further integrated 16 public transcriptome datasets (7 for seed development, 9 for salinity and drought stress) for comprehensive multi-dimensional

comparisons (Tab. S7-2). Enrichment analysis of differentially expressed genes (DEGs) during seed development revealed that upregulated genes in naked oats were primarily associated with pathways related to nutritional quality accumulation, including developmental signaling, sterol-lipid synthesis, and ion channel activity (e.g., GO:0009873, GO:0009867, GO:0000149, ko00906, ko00543; *q*value < 0.05).This suggests their transcriptional regulation tends to favor nutrient synthesis and accumulation in the grain. In contrast, upregulated genes in hulled oats during seed development were primarily enriched in pathways for cell wall organization, transmembrane transport, and basal metabolism (e.g., GO:0009834, GO:0070592, ko02010, ko00430; *q*value < 0.05), potentially linking to nutrient transport efficiency and growth regulation. Under abiotic stress, naked oats upregulated genes involved in ion homeostasis, stress signal transduction, and specific secondary metabolism (GO:0006561, GO:0017157, ko99988, ko00906). Under abiotic stress, naked oats upregulated genes involved in ion homeostasis, stress signal transduction, and specific secondary metabolism (GO:0006561, GO:0017157, ko99988, ko00906), whereas hulled oats exhibited enrichment in pathways for cell wall remodeling, terpenoid biosynthesis, and ABC transporter-mediated transport (GO:0009834, GO:0016102, GO:0006721, ko02010; *q*value < 0.05), highlighting distinct transcriptional bases underlying their differential stress adaptation strategies (Figs. S15, S16; Tab. S7).

To identify key genes underlying hulled-naked oat differentiation, we integrated 1,504 candidate selected genes in naked oats with multi-context transcriptome data to construct a combined selection signal-expression association profile. Our results revealed that during core physiological processes, we identified 39 selected genes exhibiting differential expression between hulled and naked oats, comprising 31 upregulated and 8 downregulated genes (e.g., *UMAMIT25*, *AHL1, SAG12*, *ygl8*, *WOX13*). Among these, 22 genes under directional selection and upregulated expression were significantly enriched in pathways such as lipid metabolism and post-translational protein modification (Fig. 5a; Tab. S7-6). During the seed development stage, a total of 31 selected genes were identified. Among them, upregulated genes (e.g., *PARP1*, *RAB28*, *PP2C30*, *HSP18.2*) may be involved in embryonic cell division and differentiation and ABA signal response, whereas genes under balancing selection and downregulated expression (e.g., *MPK3*, *CycB1;5*, *RSW3*) were enriched in cell cycle regulation pathways (*q*value < 0.05) (Fig. 5b; Tab. S7-6). In contrast, the 32 differentially expressed genes related to seed development in hulled oats exhibited a pattern of cooperatively upregulating energy metabolism and signal transduction while downregulating redundant hormone pathways (Fig. 5c; Tab. S7-6). Furthermore, in response to abiotic stress, 25 selected genes specifically expressed in naked oats (e.g., *PP2C09*, *FEI1*, *PME18*, *PIP3I*) generally displayed signatures of directional selection and upregulated expression, suggesting their potential functions in stress adaptation (Fig. 5c-f; Tab. S7-6). Further analysis revealed that 33 of the aforementioned 127 associated genes co-localized with published QTLs for oat yield and quality traits (Fig. 5; Tab. S7-5, highlighted in red), significantly enhancing the credibility of these candidate genes in trait differentiation."

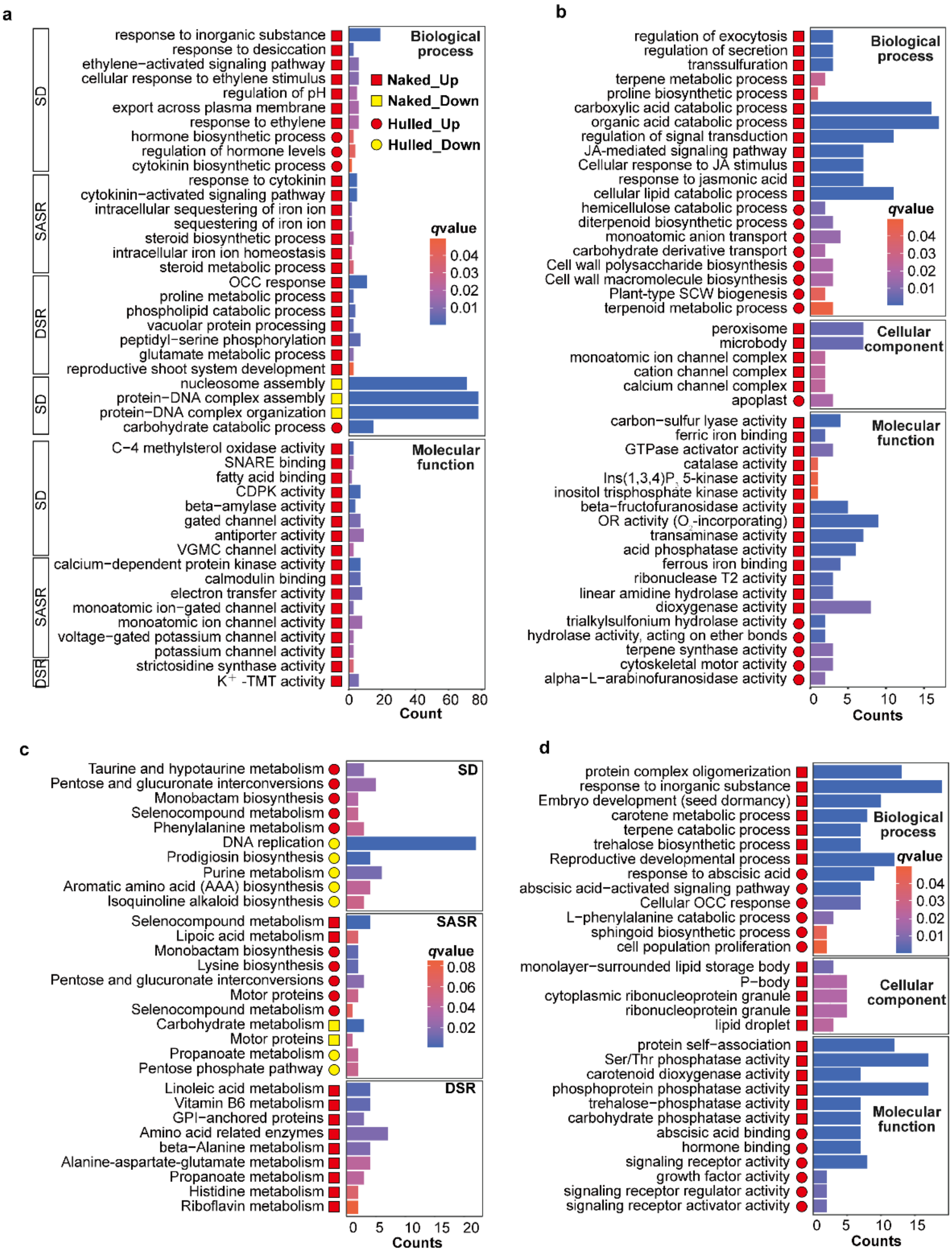

Fig. R5 Functional enrichment analysis of differentially expressed genes (DEGs) in hulled/nakedo ats across transcriptome analyses of basal physiology, seed development, and abiotic stress. (Abbreviations for each transcriptome project: SASR: Salt-alkali stress treatment; SD: Seed development; DSR: Drought stress treatment. Pooled multi-tissue samples refer to mixed samples of oat spikelets, leaves, and stems at the grain filling stage) (Revised Manuscrip Fig. S15). (a) Shared GO term enrichment analysis for DEGs commonly identified in pooled multi-tissue samples and condition-specific transcriptomes (SASR, SD, DSR); (b) GO term enrichment analysis for DEGs specifically up-regulated in hulled/naked oats during the seed development (SD) stage; (c) Shared KEGG pathway enrichment analysis for DEGs in pooled multi-tissue samples and condition-specific transcriptomes (SASR, SD, DSR); (d) GO term enrichment analysis for DEGs specifically up-regulated in hulled/naked oats under abiotic stress (SASR, DSR).

L207-on This integration of homology annotation and allele frequency shifts should be much better explained.

RESPONSE: We sincerely appreciate your supplementary suggestions on the integrated analytical approach for homologous annotation and allelic frequency variation in selected genes. In this study, we first systematically collated the screened and identified genes, then performed homology alignment and homologous gene retrieval on the Oat Genomics Online Database (http://www.waooat.cn). The homologous gene annotation pipeline for this resource is implemented following the standard workflow of OrthoFinder v2.5.4 (*Genome biology* 2019 https://doi.org/10.1186/s13059-019-1832-y). For the analysis of allelic frequencies at these target genes, we used the VCF file containing 127,563 high-quality SNP markers from 666 oat accessions as our primary dataset. We screened and extracted valid SNP loci within the physical intervals of candidate genes via VCFtools, further classified distinct alleles at each locus, and quantified the number of oat accessions associated with each allelic variant.

L229 You mention different accessions and tissues, but it is not clear what was used for the differential expression analysis.

RESPONSE: We greatly appreciate your rigorous feedback. We acknowledge an oversight in the original manuscript: we did not clearly specify the materials used for differential expression analysis. For experiments validating expression patterns of genes related to oat grain yield and crude protein content, we have added key details in the revised manuscript (Fig. R6 below; also Revised Manuscript Figs. 5d and S18b). We have clearly documented the characteristics and information of the germplasm accessions used, and provided these details in the supplementary materials (Tab. R3 below; also Revised Supplementary Tab. S2) for traceability.

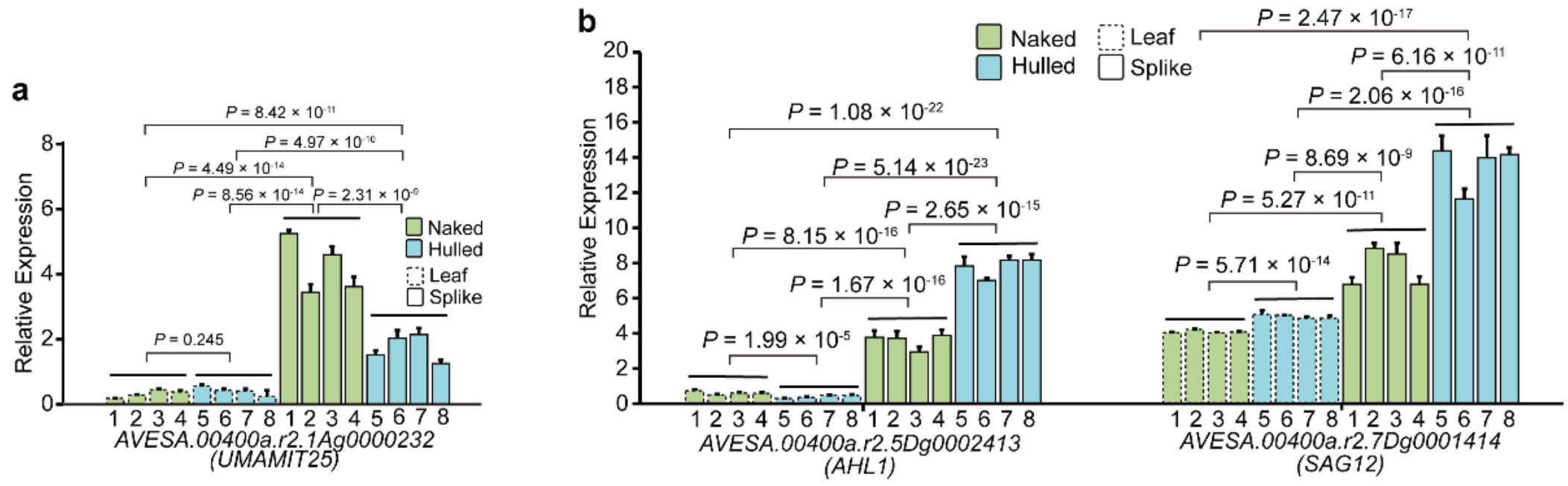

Fig. R6 Relative expression levels of *UMAMIT25*, *AHL1* and *SAG12* in leaves and spikelets during grain filling of hulled and naked oats (Revised Manuscript Fig. 5d and Fig. S18b). (a) *UMAMIT25*; (b) *AHL1* and *SAG12*. (X-axis labels 1-4 represent naked oat accessions RL3, RL8, RL10 and RL12; labels 5–8 represent hulled oat accessions RP3, RP5, RP6 and RP10. Detailed information about these accessions is provided in Tab. S1-4.)

Tab. R3 Information of hulled and naked oat accessions used for RT-PCR validation of the relative expression levels of three grain yield-related genes (Revised supplementary Tab. S1-S4).

ID	Biosample_name	Accession	Origin	Improvement	Grain_type
1	RL10	ZY00152	Hebei, China	Cultivar	naked
2	RL12	ZY00228	Shanxi, China	Landrace	naked
3	RL3	ZY00150	Hebei, China	Cultivar	naked
4	RL8	ZY00027	Inner Mongolia,	Cultivar	naked
5	RP10	Fulghum	Georgia, United	Cultivar	hulled
6	RP3	Ogle	Illinois, United	Cultivar	hulled
7	RP5	Xuekang	Tibet, China	Landrace	hulled
8	RP6	Maldwyn	Wales, United	Cultivar	hulled

L237. Fig 5a, b misses axis labels. In the Table S8 some FDR values are high and not significant, was that considered?

RESPONSE: We appreciate your comments and concerns on the identification of differentially expressed genes (DEGs). Below we clarify our screening criteria, quality control approaches, and revised analytical focus:

DEGs were identified using thresholds of p-value < 0.01 and $|log_2FC| \geq 1.5$, with no FDR correction for multiple testing. We acknowledge that the original manuscript failed to clearly state the rationale and potential limitations of this approach, which was insufficiently rigorous. This choice was driven by the low expression abundance of many key oat domestication-related genes, which are easily discarded by the stringent FDR threshold and risk the loss of core differential signals (false negatives). We thus applied this combined threshold to prioritize the detection of genes functioning in core biological processes including domestication and stress resistance.

To mitigate false positives, we introduced three key revisions in the revised manuscript: First, we cross-validated using targeted transcriptomes from seed development and salt-alkali stress, and only retained pathways consistently enriched across pooled multi-tissue and individual transcriptome datasets. Second, we enhanced enrichment analyses for individual transcriptomes to emphasize statistically significant core results (Fig. R5 above; also Revised Manuscript Fig. S15). Third, we performed multi-level cross-validation among DEGs, selected genes, and trait-associated QTLs to validate biological function at the transcriptional, domestication, and phenotypic levels, removing non-functional false positives (Fig. R7 below; also Revised Manuscript Fig. 5).

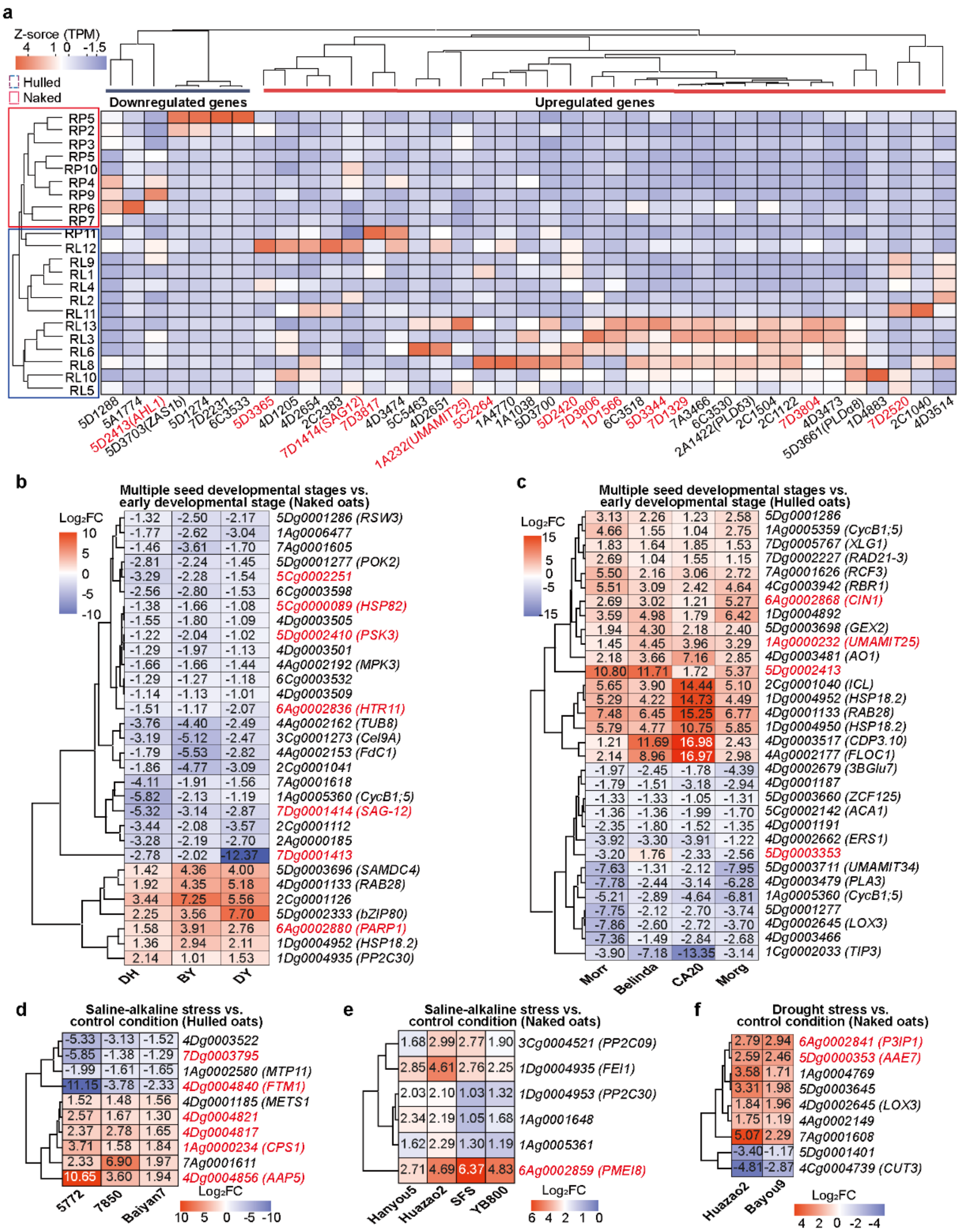

Fig. R7 Expression heatmap of selected and differentially expressed genes in naked oats based on Log₂FC values (Genes marked in red are associated with previously published oat QTLs) (Revised Manuscrip Fig. 5). (a) Intersection of DEGs between hulled and naked oats from bulk RNA-seq and selected genes in naked oats; (b) Intersection of DEGs from seed development transcriptome of naked oats and selected genes in naked oats; (c) Intersection of DEGs from seed development transcriptome of hulled oats and selected genes in naked oats; (d) Intersection of DEGs from saline-alkali stress transcriptome of naked oats and selected genes in naked oats; (e) Intersection of DEGs from drought stress transcriptome of naked oats and selected genes in naked oats.

L252 The FPKM values you show in Fig. 5c cannot be negative. Have any normalizations been applied that were not reported? Also, you show expression values per accession, but you have RNA-seq from different tissues, how can this be possible?

RESPONSE: We greatly appreciate your rigorous comments. We acknowledge minor inconsistencies between the result presentation and method description for Figure 5c in the original manuscript, and detail these issues and corresponding revisions below:

1) The negative values in the original FPKM-based heatmap arose from Z-score normalization. Further analysis revealed that FPKM values exhibit poor expression comparability across samples or genes due to the absence of global normalization. We therefore replaced FPKM with TPM and regenerated the expression heatmap for the 39 candidate genes (intersection of differentially expressed and selected genes). TPM corrects for both gene length and sequencing library size, enabling direct quantitative comparison of expression across germplasms and genes, and is more accurate than FPKM (*Nature methods* 2017 https://doi.org/10.1038/nmeth.4197). We applied Z-score normalization to TPM values; the resulting negative values are a normal post-standardization distribution, eliminating magnitude differences in raw gene expression and clearly highlighting expression clustering patterns across hulled and naked oat germplasms.

2) Regarding the correspondence between mixed-tissue samples and germplasm-specific expression levels: RNA was extracted from equal root, stem, leaf, and spike tissue mixtures of 22 hulled and naked oat accessions for transcriptome library construction, and the figure shows whole-tissue average expression per accession. While this sampling strategy lacks spatiotemporal specificity of gene expression, it directly reflects overall target gene expression differences between hulled and naked oats, consistent with our study's analytical objectives. Furthermore, key gene identification was not limited to transcriptomic data, but supported by a multi-dimensional validation system encompassing qPCR validation, genome-wide selection signal detection (π ratio, Fst, XP-CLR), and trait QTL co-localization, ensuring conclusion reliability.

In the revised manuscript, we have redrawn the relevant figure (Fig. R7a above; also Revised Manuscript Fig. 5a) and enhanced the results description. The detailed revisions are as follows: [L335-340]

"We identified 39 selected genes exhibiting differential expression between hulled and naked oats, comprising 31 upregulated and 8 downregulated genes (e.g., *UMAMIT25*, *AHL1, SAG12*, *ygl8*, *WOX13*). Among these, 22 genes under directional selection and upregulated expression were significantly enriched in pathways such as lipid metabolism and post-translational protein modification (Fig. 5a; Tab. S7-6)."

L302 The hulled and naked genotypes are not indicated in the admixture plot. If they correspond to the blue and yellow at K2, I would say the opposite, since the yellow group is strongly conserved across all K values.

RESPONSE: We greatly appreciate your rigorous comments. We acknowledge two oversights in the ancestral component analysis plot of the original manuscript: (1) the genotypes of hulled and naked oats were unlabeled, and (2) the correspondence between population colors and material types was unclear. We apologize for any inconvenience this may have caused during your review.

To resolve this, we integrated and optimized the population structure analysis results by jointly presenting the NJ phylogenetic tree (constructed with wild hexaploid oat as the outgroup) and the ancestral component plot from Admixture analysis (K=1-

7). We also clearly labeled the hulled/naked status of each accession and the core characteristics of each subpopulation in the figure (Fig. R8 below; also Revised Manuscript Fig. S3). This optimized figure intuitively illustrates the population differentiation pattern of hulled and naked oats, strongly supporting the core conclusion: hulled and naked oats do not form completely independent lineages, but exhibit extensive genetic admixture.

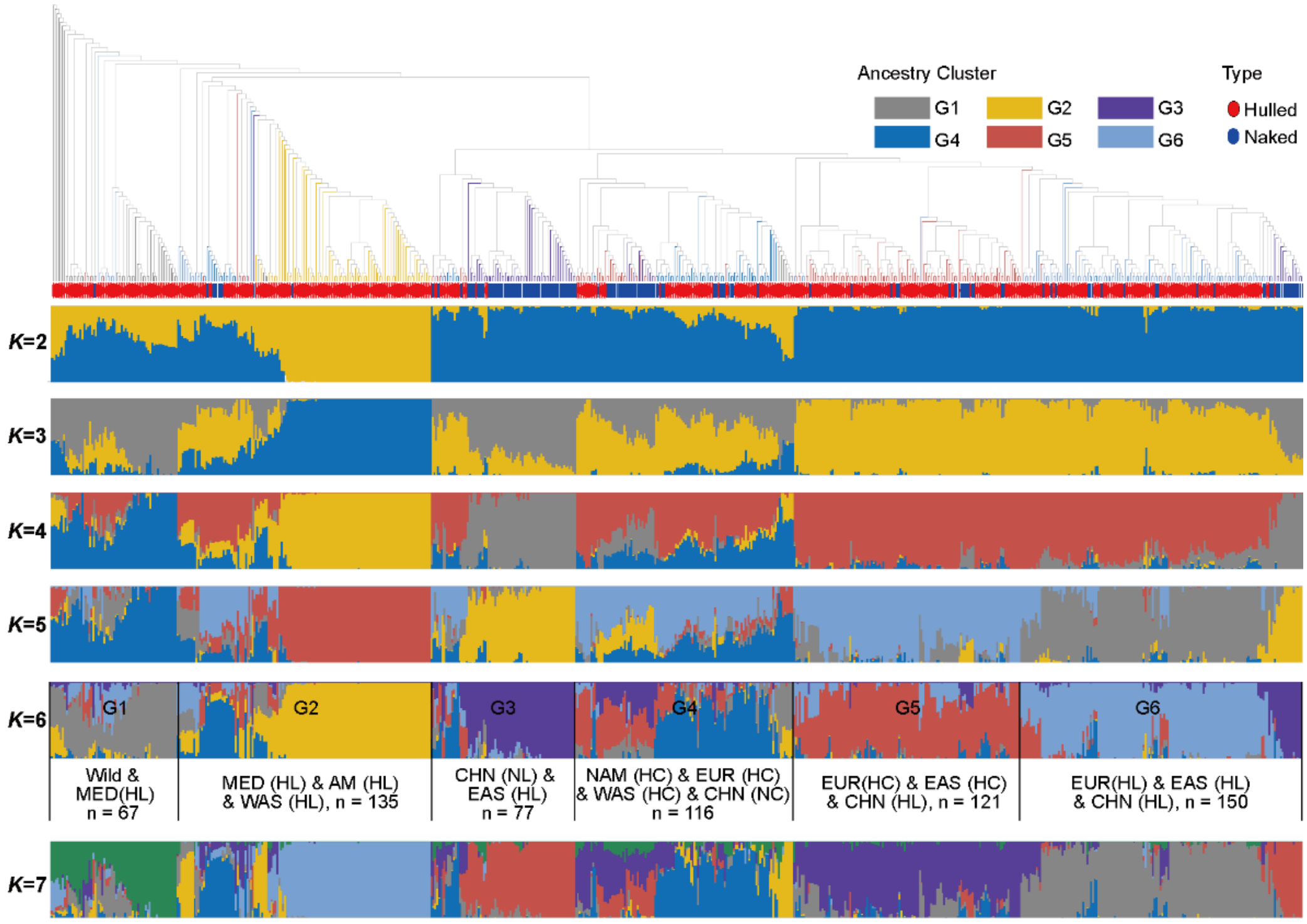

Fig. R8 shows the rooted NJ phylogenetic tree and Admixture plots at K=1 to 7 for 666 oat accessions, constructed with 127,563 high-quality SNP markers (Revised Manuscript Fig. S3).

L305 There are no Fig. 4g,h.

RESPONSE: We greatly appreciate your careful review and feedback. We sincerely apologize for this oversight, which led to erroneous references to the non-existent Fig. 4g, h. We have immediately corrected this issue by removing all references to Fig. 4g, h, and verified the consistency of all figure numbering and in-text citations throughout the manuscript to ensure full alignment between the text and figures.

L430 After the HaplotypeCaller, haven't you performed the joint genotyping? If not, how did you generate the single vcf file containing all the samples?

RESPONSE: We greatly appreciate your valuable suggestion to elaborate on the method for VCF file generation. We apologize for omitting this critical step in the Methods section of the original manuscript. We have now supplemented the relevant details as shown below : [L528-537]

"We performed variant calling with GATK (*Current protocols in bioinformatics* 2013 https://doi.org/10.1002/0471250953.bi1110s43). The pipeline involved HaplotypeCaller to generate per-sample gVCFs, followed by GenomicsDBImport to merge them, and culminated in joint genotyping across all samples using GenotypeGVCFs, yielding the raw VCF file. The filtering criteria for SNP markers were as follows: loci with base quality below 10 and sequencing depth less than 30

were excluded, as were loci with more than two alleles (retaining only biallelic loci). Additionally, loci with a minor allele frequency (MAF) ≤ 0.05 and greater than 10% missing data were removed. Considering oats are strictly self-pollinating, loci with heterozygosity exceeding 10% were also excluded. The remaining variant loci were used for further analysis."

L453 Specify which software you used for the alignment.

RESPONSE: We greatly appreciate your rigorous comments. In this study, clean RNA-seq reads were aligned to the hexaploid oat reference genome "SanfenSan" using HISAT2 (*Nature Biotechnology* 2019 https://doi.org/10.1038/s41587-019-0201-4). This tool provides efficient splice-site recognition, making it highly suitable for accurate transcriptome alignment in the complex hexaploid oat genome. We have added details of this software and its parameters to the Materials and Methods section (L578-580) to ensure a complete and traceable experimental workflow.

L481 It is not clear how you computed the XP-CLR scores.

RESPONSE: We greatly appreciate your rigorous comments. The original manuscript lacked a detailed description of the XP-CLR calculation method. We have supplemented this in the Materials and Methods section by clarifying the population settings, core principles, and parameter definitions to ensure a clear, reproducible, and traceable workflow. The detailed method is provided below: [650-655]

"The cross-population composite likelihood ratio test (XP-CLR) (*Genome research* 2010 https://doi.org/10.1101/gr.100545.109) quantifies genomic allele frequency differentiation between target and reference populations to detect selection signals by fitting a skewed model of population allele frequencies. In this study, hulled oats served as the reference and naked oats as the target population for XP-CLR score calculation, with parameters set to -window 500 kb, -step 50 kb, -minsnps 20, and others default."

We sincerely thank the reviewers for their professional and constructive comments, as well as their recognition of the research value of our study. We have carefully examined and fully addressed all comments and suggestions raised by Reviewers 2 and 3. The manuscript has been comprehensively revised according to these valuable suggestions, and our point-by-point responses are presented below. We have made every effort to resolve all remaining concerns through revisions and detailed explanations.

Reviewers' Comments:

Reviewer #1 (Remarks to the Author):
The authors have addressed all comments/questions, and the revised manuscript shows significant improvements in its flow, level of detail, and figures. I have no additional comments.
RESPONSE: Thank you very much for approving our revised manuscript which has been further improved with your help. We appreciate your great contribution to our revision.

Reviewer #2 (Remarks to the Author):

Major Comments
This manuscript has been significantly improved compared to the previous version, in which the major concerns I raised earlier have been adequately addressed. However, I still have a few comments as outlined below:

1. In Fig. 1b, the authors arrange the ADMIXTURE results according to the topology of the phylogenetic tree. However, this approach is not particularly informative and raises methodological concerns. Phylogenetic trees derived from re-sequencing data are fundamentally constructed based on pairwise genetic distances and therefore reflect clustering of genetic relatedness rather than true evolutionary history. In addition, the ordering of samples along such trees is highly sensitive to SNP filtering parameters, including thresholds for missing data and minor allele frequency (MAF). Even relatively small adjustments to these parameters can result in noticeable changes in sample arrangement. As a consequence, the current visualization may lead to a situation where samples with similar genetic ancestry components are not grouped together, reducing the clarity of population structure patterns and credibility.
For these reasons, we recommend presenting the phylogeny as an unrooted tree. Meanwhile, for ADMIXTURE visualization, samples should be ordered according to their ancestry proportions, which would provide a clearer and more interpretable representation of the underlying population structure.
RESPONS: We appreciate the reviewers' thoughtful comments and constructive suggestions. The advice concerning the visual layout of Figure 1b has substantially improves the rigor and interpretability of our results.

In the revised manuscript, we have fully separated the ADMIXTURE ancestry bar plot from phylogenetic tree topology. As noted by the reviewer, tree-based sample ordering is susceptible to biases introduced by SNP filtering criteria. Accordingly, we have reordered all individuals in the ADMIXTURE plot solely according to their individual ancestry component proportions (Fig. R1a below; also Revised Manuscript Fig. 1b). This adjustment clusters samples with closely related genetic backgrounds and homogeneous ancestry profiles together, which eliminates the artefactual ordering bias

in our original display. Population structure patterns are now more clearly resolved, and our presentation follows standard conventions for ADMIXTURE visualization in population genetics.

In addition, we have reconstructed and presented the phylogenetic tree as an unrooted tree (Fig. R1b below; also Revised Manuscript Fig. 1c) in accordance with your recommendation. This unrooted configuration objectively illustrates genetic relationships among accessions without imposing directional evolutionary assumptions, thus avoiding potential methodological misinterpretations associated with the previous rooted layout and further strengthening the robustness of our analyses.

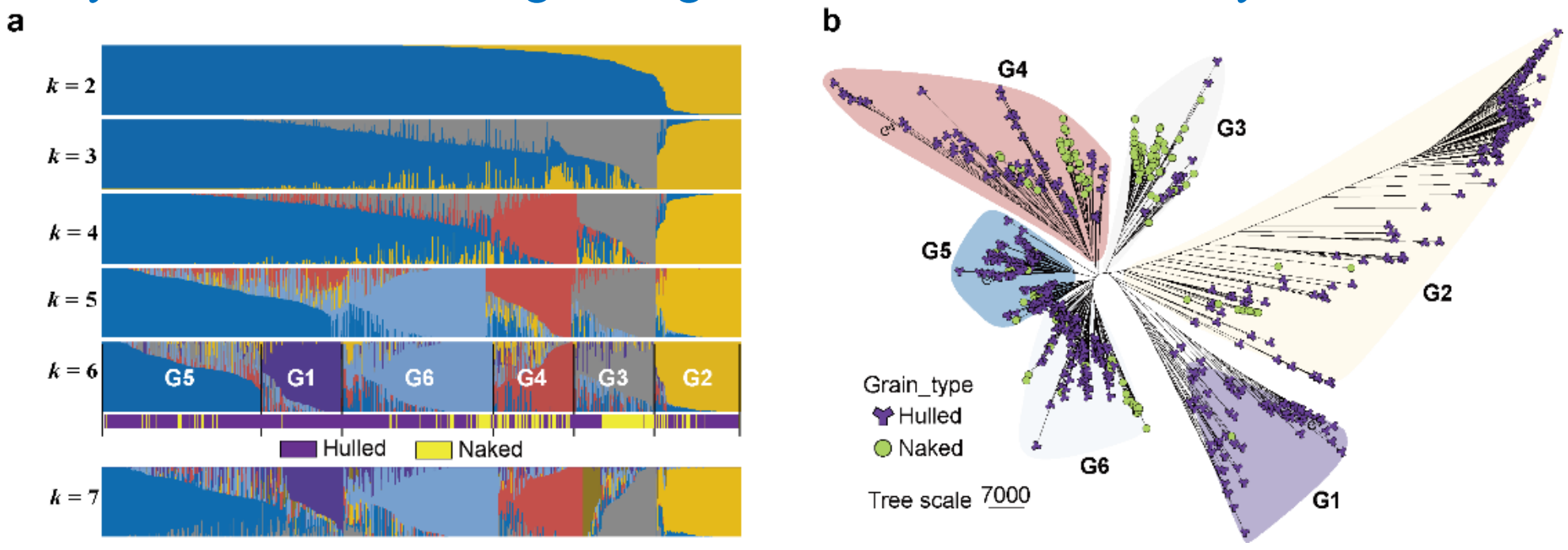

Fig. R1 Population structure analysis of 666 hexaploid oat accessions using 127,563 SNPs (Revised Manuscript Fig. 1b and c).

(a) Genetic ancestry components under different assumed population numbers (K = 2 – 7) and subgroup assignment (G1 – G6) at K = 6 (Revised Manuscript Fig. 1b); (b) Unrooted phylogenetic tree: subgroups (G1 – G6) and hulled/naked grain distribution (Revised Manuscript Fig. 1c).

2. Fst = 0.0701 is described as indicating "weak differentiation" based on the study by Nan et al. (Gigascience, 2022; https://doi.org/10.1093/gigascience/giad061) on hexaploid oat populations. However, upon careful examination of this article, we did not find an explicit classification standard defining thresholds for weak, moderate, or significant genetic differentiation (e.g., Fst < 0.08, 0.08-0.15, > 0.15). Therefore, the use of this specific categorization appears to be unsupported by the cited reference and may require either clarification or citation of an appropriate source that explicitly defines these thresholds.

RESPONSE: We appreciate your careful review and constructive comments, which are of great importance for improving the scientific soundness and academic rigor of our manuscript. After verifying the relevant literature, we confirm that the research on hexaploid oats by Nan et al. (Gigascience, 2022) did not establish any grading thresholds for the genetic differentiation index (F_{st}). We sincerely apologize for the inappropriate literature citation in our original manuscript.

In this study, the levels of genetic differentiation among all populations, as well as our subsequent analyses and discussions, were all assessed by adopting the well-established universal F_{st} classification system originally proposed by Wright (*Evolution and the Genetics of Populations, Volume 4: Variability within and among Natural Populations* 1978 https://doi.org/10.2307/2529965), Hartl et al. (*Principles of Population Genetics* 1997 https://www.sinauer.com/principles-of-population-genetics-4th-edition), and Frankham (*Introduction to Conservation Genetics* 2002 https://doi.org/10.1017/CBO9780511809002). The universally recognized threshold intervals summarized from these classical studies are listed below: $F_{st} < 0.05$: negligible genetic differentiation; $0.05 \leq F_{st} \leq 0.15$: weak to moderate genetic differentiation; $F_{st} > 0.15$: high genetic differentiation; $F_{st} > 0.25$: very strong genetic differentiation.

We have supplemented the citations of these classical references in the Methods section of the revised manuscript, and explicitly clarified the source of the genetic differentiation grading criteria adopted in our study. The relevant revisions are as follows: [L683–L686]

"Population differentiation was classified using the classic criteria of Wright[64] and Hart & Clark[65]: $F_{st} < 0.05$ for negligible differentiation, $0.05 \leq F_{st} \leq 0.15$ for weak-to-moderate differentiation, and $F_{st} > 0.15$ for high differentiation, $F_{st} > 0.25$ for very strong genetic differentiation."

3. Lines 196-199: The authors state that subpopulation GⅣ diverged approximately 6,124 generations ago and underwent an extreme population bottleneck (Ne ≈ 187). While this provides a clear demographic timeframe, such a severe bottleneck is typically accompanied by intense genetic drift. The authors need to clarify whether the observed genetic signal is a result of active anthropogenic selection during domestication, or simply a result of genetic drift associated with a founder effect during geographic isolation. The authors are encouraged to supplement their analysis or discuss within the text whether there are distinct selective sweep regions in the GⅣ group. If it can be demonstrated that Chinese naked oats possess unique selection signals divergent from GⅢ—particularly those associated with key traits such as the naked grain phenotype, crude protein and fat content—the argument for North China as a 'secondary domestication center' would be significantly more compelling and robust.

RESPONSE: We sincerely appreciate your valuable suggestions. Your advice to distinguish between directional artificial selection and genetic drift induced by the founder effect, and to explore GIV subpopulation-specific selection signals in support of the contention that North China is a secondary domestication center, is crucial for refining the evolutionary inferences of this study.

Despite experiencing a strong genetic bottleneck following differentiation, the genomic features of the GIV subpopulation indicate that its genetic and phenotypic differentiation is driven primarily by directional artificial selection rather than random genetic drift. Specifically, the nucleotide diversity of GIV ($\pi = 2.7632 \times 10^{-6}$) is 47.88% lower than that of its ancestral group GIII ($\pi = 4.0862 \times 10^{-6}$), with heterogeneous diversity loss among different chromosomes and regions. Additionally, GIV has a significantly higher proportion of genomic windows with Tajima's $D < 0$ than GIII, and its proportion of long conserved homozygous haplotypes (ROH) is 67.52%—far higher than GIII's 10.91% (Revised Supplementary Tab. S4.6). These lines of evidence confirm that strong directional artificial selection, superimposed on the bottleneck effect, drove significant genetic and phenotypic differentiation, rather than random genomic drift alone.

We combined three methods: F_{st}, the nucleotide diversity ratio (GIII vs GIV), and XP-CLR. After selecting the top 1% of genomic windows detected by at least two methods, we identified 1179 stable GIV-specific selected genes (Revised Manuscript Tab. S4.6). Functional enrichment analysis revealed that these genes were primarily enriched in pathways such as amino acid metabolism, lipid biosynthesis, nutrient transport and accumulation, and cuticular wax synthesis (Fig. R2 and R3 below; also Revised Manuscript Fig. S20 and S21). These pathways correspond to the morphological development, grain quality advantages, and stress adaptation of naked oats in North China's specific environmental conditions. Furthermore, colocalization analysis with published oat QTLs identified 487 selected genes in known QTL intervals.

Of these, 194 are associated with β-glucan content, 75 regulate grain crude protein content, and 128 participate in drought stress response (Tab. R1 and Fig. R4 below; also Revised Supplementary Tab. S4.6 and Revised Manuscript Fig. S22). This confirms multidirectionally that these genes are functional targets of artificial directional improvement.

In summary, the unique genomic variation and phenotypic traits of GIV stem primarily from directional artificial selection during secondary domestication, rather than genetic drift induced by the founder effect. This provides solid genomic evidence for North China as the secondary domestication center of naked oats. We have supplemented the relevant analyses and improved the discussion section of the main text as you suggested. The relevant revisions are as follows: [L467–L489]

"Severe founder bottlenecks are typically associated with genetic drift that may obscure genuine selection signals. Despite experiencing an extreme bottleneck ($N_e \approx$ 187), genome-wide scanning and population differentiation analyses demonstrate that the genetic divergence and phenotypic specialization of GIV were predominantly driven by directional artificial selection. Specifically, nucleotide diversity in GIV is 47.88% lower than that in GIII, with heterogeneous genomic distribution of this diversity loss (Fig. 2g; Tab. S4.2). GIV also harbors a higher proportion of genomic windows with negative Tajima's D values and an elevated proportion of long runs of homozygosity (1 Mb–3 Mb ROH: 67.52% in GIV vs. 10.91% in GIII) (Figs. 2d and S9b; Tab. S4.2), consistent with strong directional selection superimposed on underlying bottleneck effects. A total of 1,179 GIV-specific selected genes identified via integrated screening were significantly enriched in amino acid and lipid metabolic pathways ($q < 0.01$) (Figs. S21, S22; Tab. S4.6), and overlapped with previously reported QTLs underlying grain β-glucan content, crude protein content and drought tolerance, consistent with the phenotypic characteristics of the GIV subpopulation (Fig. S23; Tab. S4.6). Furthermore, the divergence time of GIV from GIII at approximately 6,124 generations ago (~6,000 BP) coincides closely with the eastward dispersal of West Asian cereal crops [34, 36, 37]. Archaeological remains of charred naked oat grains from northern China (4,000–3,500 BP) and historical records of naked oat cultivation dating to the Northern Wei Dynasty further validate this molecular evolutionary timeline [38-41]. Collectively, integrated genetic, archaeological, and historical evidence corroborates northern China as the secondary domestication center of global naked oats."

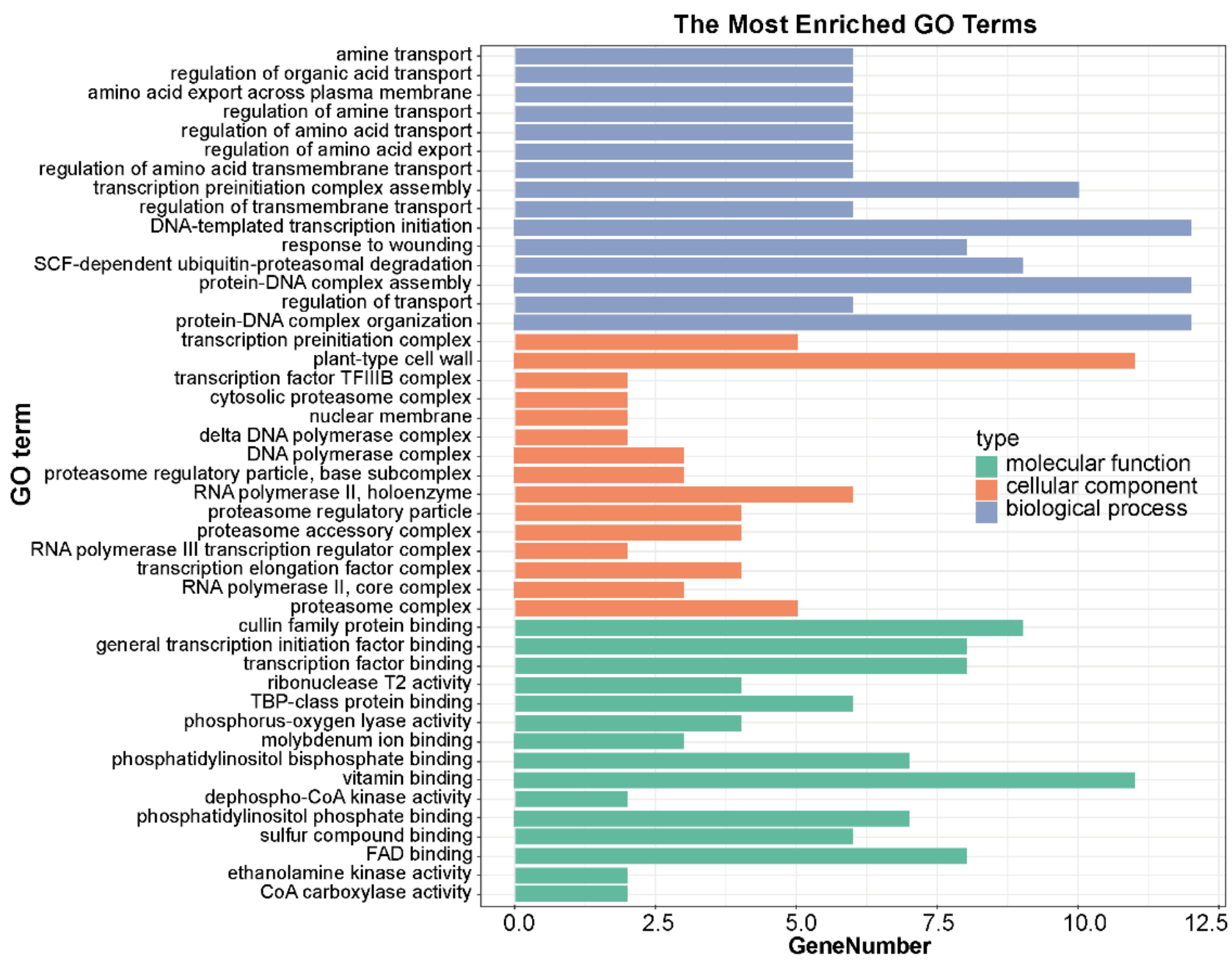

Fig. R2 GO enrichment results of selected genes in GⅣ (the core subgroup of landrace naked oats), showing the top 15 significantly enriched terms in each category of Molecular Function, Cellular Component and Biological Process (*P* < 0.05). (Revised Manuscript Fig. S21).

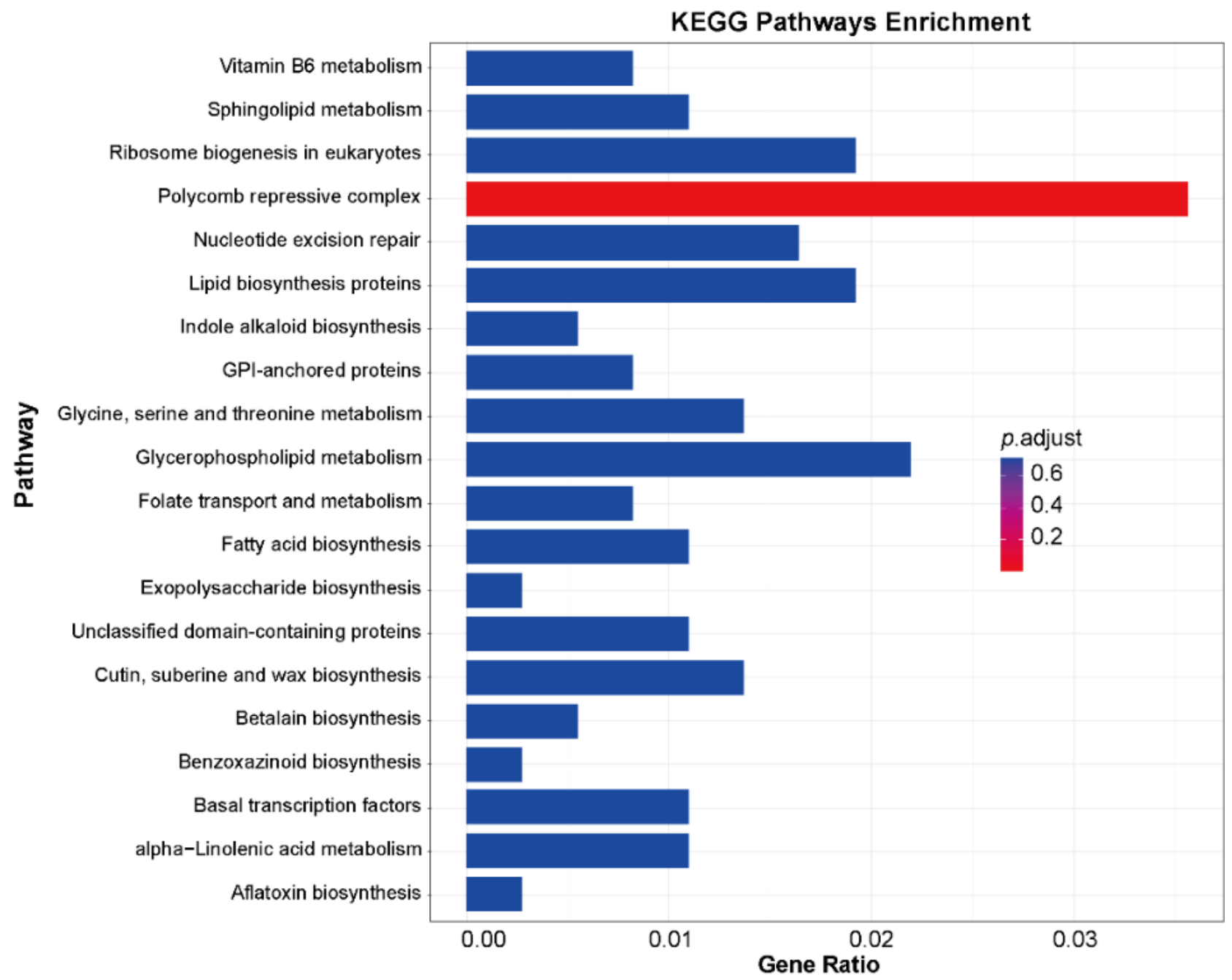

Fig. R3 KEGG enrichment results of genes under selection in GⅣ (the core subgroup of landrace naked oats), showing the top 20 enriched terms. (Revised Manuscript Fig. S22).

Tab R1 Associations between GIV selective sweep genes and multi-trait oat QTLs with GIII as reference background (Revised Supplementary Tab. S4.6)

CHROM	QTLs	Gene No.	Reference
chr1D	UMN28BRV/CDO20(GW)	35	https://doi.org/10.1007/s001220100579
chr6C	S6C_54231389(GW)	39	https://www.x-mol.com/paperRedirect/1723748064953126912
chr7D	S7D_305063624(TGW/GL)	16	https://www.x-mol.com/paperRedirect/1723748064953126912
chr4A	Schr4A_435265955(β-Glucan)	101	https://doi.org/10.1186/s12864-024-11174-5
chr4C	Schr4C_127448389(β-Glucan)	10	https://doi.org/10.1186/s12864-024-11174-5
chr5A	Schr5A_479430988(β-Glucan)	6	https://doi.org/10.1186/s12864-024-11174-5
chr6A	Schr6A_398040302(β-Glucan)	52	https://doi.org/10.1186/s12864-024-11174-5
chr6C	Schr6C_45173202(β-Glucan)	25	https://doi.org/10.1186/s12864-024-11174-5
chr2D	S2D_485481234 (GP)	47	https://doi.org/10.3390/ijms24065581
chr7C	7C_413114334(QTL20/GP)	28	https://doi.org/10.3390/ijms24065581
chr5C	5C_498439125(Drought)	40	https://doi.org/10.1038/s41588-025-02294-z
chr7C	7C_40045934(Drought)	36	https://doi.org/10.1038/s41588-025-02294-z
chr7C	7C_421.1-439.5(Drought)	40	https://doi.org/10.1038/s41588-025-02294-z
chr6A	QHeadingdate.fgl-6A&Drought	12	https://doi.org/10.1002/tpg2.70060

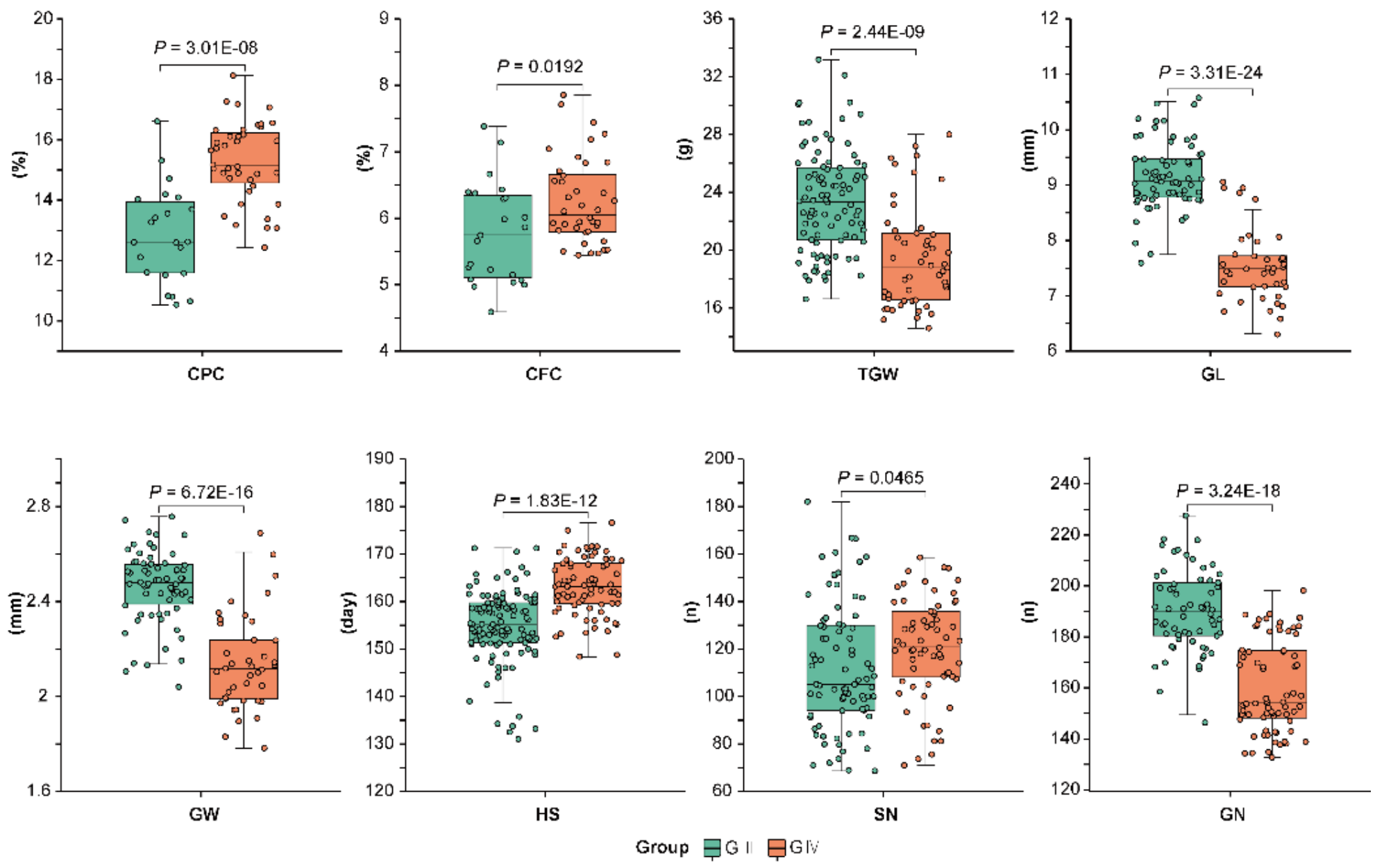

Fig. R4 Boxplots comparing phenotypic trait differences between subgroup GIV (core landrace naked oat subgroup) and subgroup GIII (hulled oats from the Central and Eastern Eurasia) (Revised Manuscript Fig. S23).

4. Lines 202-206: The authors state that "GⅡ's Ne (969) was markedly lower than the model-predicted value (1,860) ... These patterns collectively support GⅡ as the ancestral lineage..." Under typical evolutionary scenarios, ancestral populations should have a higher effective population size (Ne) and greater genetic diversity. However, the authors identify GⅡ as the ancestral group despite its observed Ne being significantly lower than the model-predicted value, which appears logically inconsistent with the 'ancestral diversity center' hypothesis. I suggest the authors briefly explain why the ancestral population GⅡ has a lower Ne. Is it due to recent population contraction in the Mediterranean region, or insufficient sampling representativeness?

RESPONSE: We appreciate your constructive comment. We understand your concern that ancestral populations are generally expected to harbour larger effective population sizes (N_e) under classical evolutionary theory, whereas the observed N_e of our ancestral group GII (969) is lower than the modelled prediction (1860). Accordingly, we have supplemented evolutionary interpretations and supporting references in the Discussion section.

Notably, nucleotide diversity reflects ancient genetic variation accumulated over long-term evolutionary history and serves as a core criterion for identifying ancestral lineages and centres of origin. By contrast, contemporary N_e only represents recent population scale, and the two metrics are not strictly positively correlated. A common pattern in crop evolution is that ancestral populations at origin centres retain the richest ancient genetic variation but frequently undergo recent population contraction driven by Holocene climatic oscillations, habitat changes and human activities, resulting in reduced contemporary N_e. In contrast, derived cultivated populations often exhibit elevated contemporary N_e due to human-mediated dispersal and frequent intraspecific gene flow. This pattern is well-documented in wheat, barley and other crops (*Science Advances* 2019 https://doi.org/10.1126/sciadv.aav0536; *Ecology and Evolution* 2024 https://doi.org/10.1002/ece3.70068).

In our study, GII is characterised by the highest genome-wide nucleotide diversity, most ancient ancestral alleles, basal phylogenetic position and earliest divergence time. Combined with demographic modelling results, multiple lines of evidence robustly support its status as the ancestral lineage. The relatively low contemporary N_e of GII can be explained by subsequent population contraction within its Mediterranean native range. We have integrated this evolutionary reasoning into the corresponding discussion in the manuscript. The relevant revisions are as follows: [L454–L467]

"Population demographic analyses reveal that the effective population size of GII (N_e = 969) is lower than model predictions (Fig. 2j), which does not contradict its ancestral status inferred from nucleotide diversity and other evolutionary indices. Nucleotide diversity reflects long-term accumulated ancient genetic variation, whereas contemporary N_e only captures recent demographic dynamics, and the two metrics are not intrinsically correlated. As observed in numerous crop species, progenitor populations in domestication centers often exhibit reduced N_e under environmental and anthropogenic pressures, while derived lineages commonly show expanded effective population sizes accompanied by subsequent gene flow [34, 35]. Multiple independent lines of evidence support the ancestral position of GII, including its highest nucleotide diversity, most ancient allelic lineages, basal phylogenetic placement, and demographic simulation results (Figs. 2d–k; Tabs. S4.2, S4.3). Its reduced Ne is likely attributable to a recent population contraction within its native Mediterranean range."

5. Lines 209-211: The authors conclude by inferring that: “...followed by secondary domestication in North China (highest π in naked oats)”. Relying solely on the observation that ‘naked oats have the highest diversity in China’ is insufficient to designate China as the center of domestication. It is recommended that the authors supplement the Discussion section by addressing whether any archaeological or phenological evidence supports the presence of oat utilization in North China around the estimated timeframe (~6,124 generations ago). Aligning the genetic timeline with historical and archaeological records would provide a more cohesive and closed-loop argument for the proposed evolutionary scenario.

RESPONSE: We appreciate your valuable suggestion. Integrating evolutionary divergence time, archaeobotanical remains and agricultural historical records for mutual verification is essential for consolidating the argument regarding the secondary domestication center of naked oats.

We estimated that the divergence time between the core naked oat subpopulation GIV and the Eurasian hulled oat subpopulation GIII is approximately 6,124 generations ago. Considering the annual life cycle of oats, this timeframe corresponds to the late Neolithic to early Bronze Age in northern China, which highly matches the eastward dispersal chronology of West Asian dry-farming crops. Such transregional dispersal and secondary domestication patterns have been widely reported in various cereal crops (*Science Advances* 2019 https://doi.org/10.1126/sciadv.aav0536; *The Holocene* 2016 https://doi.org/10.1177/0959683616650268; *Proceedings of the Royal Society B: Biological Sciences* 2014 https://doi.org/10.1098/rspb.2013.3382).

Archaeological evidence corroborates this temporal sequence. Charred oat remains excavated from the Xishanping and Donghuishan sites in Gansu indicate that oats had been introduced and exploited in northern China by the early Bronze Age [R1, R2]. Oat starch grains dating to the late Western Zhou Dynasty from the Xishan site further confirm its sustained local utilization [R3]. Meanwhile, naked oat remains recovered from multiple sites across North China and Inner Mongolia verify that naked oats had developed into a stably cultivated crop in these regions [R4, R5]. With regard to historical records, naked oat cultivation dates back to the Spring and Autumn Period. Textual verification of Qi Min Yao Shu also confirms large-scale cultivation of naked oats across North China during the Northern Wei Dynasty [R6].

Collectively, genetic, archaeobotanical and historical evidence forms a complete closed chain, robustly supporting that northern China is the secondary domestication center of naked oats. We have supplemented the above content in the Discussion section of the revised manuscript. The relevant revisions are as follows: [L482–L489]

“Furthermore, the divergence time of GIV from GIII at approximately 6,124 generations ago (~6,000 BP) coincides closely with the eastward dispersal of West Asian cereal crops [34, 36, 37]. Archaeological remains of charred naked oat grains from northern China (4,000–3,500 BP) and historical records of naked oat cultivation dating to the Northern Wei Dynasty further validate this molecular evolutionary timeline [38-41]. Collectively, integrated genetic, archaeological, and historical evidence confirms northern China as the secondary domestication center of global naked oats.”

In addition, the domestic archaeobotanical and agricultural historical references cited herein lack international DOI numbers, and Chinese academic databases maybe inaccessible to overseas users. To facilitate your inspection of original sources, we have uploaded the corresponding full-text PDFs as supplementary attachments (filenames: Supplementary_Chinese_Oat_Archaeobotanical_Historical_Literatures_R1－R6).

[R1] Li, X. Q. et al. The earliest agricultural diversification in China revealed by biological records from the Xishanping site, Gansu. *Science in China Series D: Earth Sciences* **37**, 934－940 (2007).
[R2] Wei, Y. M. et al. Study on crop remains from Donghuishan and Xihuishan sites in Hexi Corridor, China. *Journal of Triticeae* Crops **40**, 1327 (2020).
[R3] Ge, W., Liu, L., Zhao, C. C., & Jin, Z. Y. Starch residue analysis of artifacts from the Xishan Site. *Archaeology and Cultural Relics* **(4)**, 111－119 (2020).
[R4] Zhao Z. J. Restoration of Production and Management Mode in the Farming-Pastoral Zone During Historical Periods: Flotation Results of Plant Remains from Archaeological Survey in Cangtou River Basin. *Agricultural Archaeology* **(04)**, 7-17 (2020).
[R5] Zhong, H., Dong, X. L., & Wang, Y. Study of plant remains from the Shangjing (Upper Capital) City of the Liao Dynasty. *Sciences of Conservation and Archaeology* **37**, 12－23 (2025).
[R6] Yang, H. P. A textual research on the history of naked oat (*Avena nuda* L.). *Inner Mongolia Agricultural Science and Technology* **(5)**, 33 (1979).

6. Line 264: The statistical approach used to calculate the P-values is not clearly described. We recommend that the authors explicitly specify the method used for significance testing, including the underlying assumptions and parameters, to ensure the robustness and reproducibility of these results.
RESPONSE: We appreciate your rigorous feedback. We apologize for the inadequate description of the significance testing method in the original manuscript. Using 127,563 high-quality SNP loci, we calculated nucleotide diversity (π) and Tajima's D values across all 21 chromosomes of both hulled and naked oats via a sliding window approach (500 kb window size, 50 kb step size). Since genomic window statistics typically deviate from a normal distribution, we used the Wilcoxon rank-sum test (Mann–Whitney U test) to assess intergroup differences. This non-parametric test requires no normality assumption, making it well-suited to the distributional characteristics of genomic window data. All windows within each chromosome were treated as independent analytical units, with a significance threshold set at $P < 0.05$. These statistical methods, parameter settings, and their underlying rationale have been fully integrated into the Materials and Methods section of the revised manuscript. The relevant revisions are as follows: [L677–L683]

"We used BCFtools [63] to extract and convert VCF subfiles for each subpopulation. Nucleotide diversity (π), Tajima's D neutrality test, and the genetic differentiation index (F_{st}) among distinct oat subpopulations were computed using VCFtools [59]. The calculations were performed with a sliding window approach, using a window size of 500 kb and a step size of 50 kb, and intergroup differences in nucleotide diversity between hulled and naked oats were assessed using the Wilcoxon rank-sum test (Mann–Whitney U test), with a significance threshold set at $P < 0.05$."

Reviewer #3 (Remarks to the Author):

I appreciate the authors' efforts to improve the manuscript and the additional analyses provided in this revised version. The deeper exploration of evolutionary history is valuable, and the second part of the manuscript dedicated to phenotypic characterization and RNA-seq analysis has notably improved. This section is now clearer and provides more convincing support for the biological interpretation of the results.
However, several important issues remain in the first part of the manuscript, particularly concerning the population genetic framework and the interpretation of domestication pathways. A clearer explanation of the defined admixture groups is needed, including a more explicit justification of the admixture membership thresholds used to assign individuals to clusters. In particular, the attribution of hulled and naked genotypes should be carefully revised. In some figures, these classifications appear potentially mislabeled, which is a critical issue because these categories are central to the authors' conclusions. In addition, the authors should more clearly highlight the position of the wild accessions within the admixture analysis, as this information is essential to support the proposed domestication scenario.
The analytical order and presentation of results in the first section are still somewhat confusing, with population genetic metrics presented in a way that does not always clearly support the narrative. Improving the logical flow of analyses and ensuring that figures are consistently and correctly referenced would substantially strengthen the manuscript. At present, some figures are not optimally used to support the authors' claims, making it difficult for the reader to follow the argument.
Finally, the abstract would benefit from careful language revision, as some typos and phrasing issues reduce clarity.
Overall, while the second part of the manuscript has clearly improved, the first section still requires clarification and better structure in order to fully support the proposed evolutionary interpretation. Addressing these points would significantly enhance the clarity and impact of the study.

RESPONSE: We greatly appreciate your positive assessment and valuable guidance on our manuscript. Your recognition of the academic significance of this study and the improvements we have made is highly encouraging to us. We have carefully addressed all of your comments point-by-point, and have comprehensively revised and improved the manuscript accordingly. Detailed responses are as follows:
1. Regarding the unclear criteria for defining admixed individuals and individual assignment thresholds in the original manuscript, we apologise for the insufficient descriptions and ambiguous visualisations that caused confusion. In accordance with standard practices in population genetics, we classified subpopulations using a core threshold of > 0.5 single ancestry proportion in Admixture analysis. Accessions exceeding this threshold were assigned to corresponding pure subpopulations, whereas all accessions with all ancestry components below 0.5 were categorised as admixed populations representing intermediate germplasm with recent genetic introgression. Furthermore, we cross-validated the initial grouping results with NJ phylogenetic clustering, geographic origins and germplasm types of all accessions.
In the previous version, the Admixture bar plots were inappropriately sorted according to the topological structure of the phylogenetic tree, which prevented genetically similar accessions from clustering together and obscured the population structure patterns. In the revised manuscript, we have reordered the Admixture results based on ancestry

proportions, and presented the phylogenetic tree as an unrooted tree for clearer visualisation. We have also supplemented the complete criteria for sample classification in the revised manuscript. The relevant revisions are as follows: [L111–L113]

"By integrating Admixture cross-validation (K = 1–20) with neighbor-joining (NJ) phylogenetic analysis using a threshold of > 0.5 for ancestry proportion, we divided these accessions into six subgroups (G1–G6) (Figs 1b, 1c and S2; Tab. S1.2)."

2. In response to your comment regarding the disorganised analytical sequence and ambiguous logical associations between population differentiation and evolutionary inferences in the original manuscript, we have implemented comprehensive revisions. We have corrected all misannotations across figures, supplemented the population classification of wild germplasm, optimised the layout of composite figures in the main text (Figs. R1 and R2 below; also Revised Manuscript Figs 1 and 2), and reorganised the entire narrative logic of the Result section.

(1) In the revised Results section "Population structure and differentiation", we first integrated Admixture, neighbor-joining (NJ) phylogenetic and principal component (PCA) analyses to characterise overall population stratification. We clarified the germplasm composition of each subpopulation and global structural patterns, then evaluated inter-subpopulation genetic differentiation and genetic diversity. Finally, we elucidated the underlying evolutionary drivers that have shaped the present population framework and differentiation patterns. The relevant revisions are as follows: [L108–L171]

"Based on 127,563 bi-allelic SNPs filtered with minor allele frequency (MAF) ≥ 0.05 and missing rate ≤ 0.1 (Fig. S1), we characterized the global population structure and genetic diversity of 666 oat accessions from 65 countries and regions (Fig. 1a; Tab. S1.1). By integrating Admixture cross-validation (K = 1–20) with neighbor-joining (NJ) phylogenetic analysis using a threshold of > 0.5 for ancestry proportion, we divided these accessions into six subgroups (G1–G6) (Figs 1b, 1c and S2; Tab. S1.2). While accessions of each subpopulation exhibit distinct geographic origins, prevalent genetic admixture between hulled and naked oats and cross- regional gene flow are present, with no strict geographic-genetic correspondence. Specifically, G1 represents the population convergence of West Asia (WAS) and Africa (AFR), comprising 57 hulled oats predominantly from Ethiopia and Egypt, 7 wild relatives, and one Chinese naked landrace from Hebei. G2 constitutes a hulled oat landrace core subpopulation from the Mediterranean (MED; 57 accessions) and West Asia (34 accessions). G3 is a subpopulation dominated by Chinese (CHN) naked oats, which account for 79.2% of their accessions. G4 represents trans-Pacific gene flow, consisting of 43 North American (NAM) hulled oats and 41 Chinese naked oats from northern domestication hubs. G5 is a Sino-Eurasian mosaic comprising 53 European (EUR) hulled oat elites and 13 Chinese naked oat landraces from the Shanxi-Hebei-Inner Mongolia region. G6 is a typical geographically adjacent admixed subpopulation, comprising 54 Mediterranean hulled oat landraces, 28 European hulled oats, and 55 Asian accessions (including 28 Chinese naked oats). Principal component analysis (PCA) supports this population structure and widespread genetic admixture: only G2 forms independent clustering on PC1, and G3 (the core naked oat subpopulation) clusters exclusively on PC3; extensive grain-type and geographic overlap further confirms pervasive global germplasm admixture (Fig. 1d; Tab. S1.3).

Hulled oats exhibit significantly higher nucleotide diversity ($\pi = 2.79 \times 10^{-6}$) and faster linkage disequilibrium decay (0.96 Mb) than naked oats ($\pi = 1.64 \times 10^{-6}$; 5.32

Mb) (Figs 1e, 1f; Tab. S2.1). Together with the higher proportion of long runs of homozygosity (ROH, 1 Mb–3 Mb) in naked oats (14.4%) relative to hulled oats (9.5%), naked oats harbour a proportion of loci with Tajima's $D < 0$ that is 18.4-fold that of hulled oats. These results demonstrate that naked oats underwent a stronger genetic bottleneck and intensified domestication selection pressure (Figs. S3c and S4a, S4b; Tabs. S2.2 and S3.1). Among the six subgroups (Figs. 1g–j and S3; Tabs. S2.5, S2.6), G2 exhibited the weakest domestication selection (Tajima's D <0, 1.4%–4.8%) and the highest nucleotide diversity ($\pi = 3.31 \times 10^{-6}$). G3 exhibits intense domestication selection, alongside markedly reduced genetic diversity, with the proportion of loci with Tajima's $D < 0$ 11.7–16.3-fold that of G2. G5 displays the lowest nucleotide diversity ($\pi = 1.26 \times 10^{-6}$) due to intensive artificial breeding and domestication pressure. Weak overall genetic differentiation between hulled and naked oats ($F_{st} = 0.0701$) aligns with their substantial germplasm overlap. Subpopulation differentiation patterns are highly heterogeneous, driven by long-term geographic isolation and divergent selection pressure. Specifically, G2 shows strong genetic differentiation from all other subgroups ($F_{st} > 0.15$). In contrast, G4 shows weak differentiation from most subgroups (F_{st} = 0.05–0.09) and retains high nucleotide diversity ($\pi = 2.07 \times 10^{-6}$).

ROH scanning, ABBA-BABA tests, and TreeMix analysis revealed extensive inter-subpopulation introgression shaping global oat germplasm diversity. We detected robust introgression from hulled oat elites into naked oats ($D = 0.35$, $P < 0.01$), which explains the weak interspecific differentiation and germplasm overlap between the two grain types (Figs. S4, S6a; Tab. S3.1). By contrast, among the six subgroups (Figs. 1k, S5, S6b–r; Tab. S3.2), moderate-to-high introgression from Mediterranean (MED) hulled landraces (G2) is widespread across all geographical regions and all other genetic subgroups. Supported by significant genetic differentiation estimates (D = 0.18–0.48, $P < 0.05$), these findings collectively establish G2 as the primary genetic donor within the global oat germplasm network. Additionally, frequent and bidirectional introgression occurs between G4 (NAM hulled oats) and MED/EUR hulled oats, along with high proportions of shared long homozygous linkage fragments (1–3 Mb; 26.2%–37.8%), which demonstrating that G4 acts as the core fusion hub subgroup of global high-quality oat germplasm resources. Moderate introgression from East Asian (EAS)/CHN oat clades into MED, EUR, and NAM germplasm was also detected. Collectively, multi-directional transcontinental introgression, divergent domestication pressures, and geographic isolation have shaped the global genomic landscape of cultivated oats. Identifying G2 as the primary genetic donor and G4 as a modern breeding hub provides a foundation for future genetic improvement and global utilization of oat germplasm."

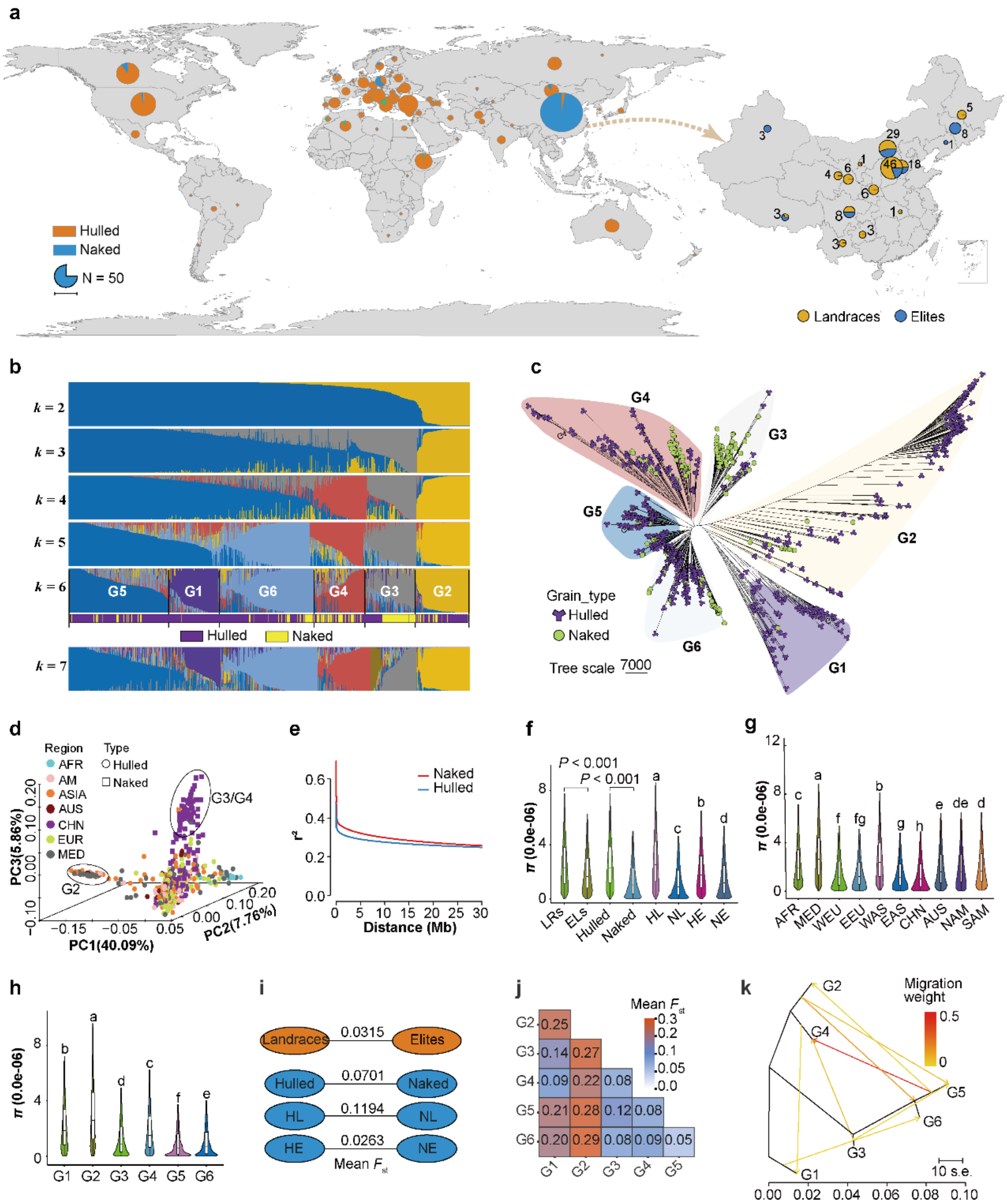

Fig. R1 Population structure and genetic diversity analysis of 666 oat germplasm resources based on 127,563 SNP markers. (a) Genetic ancestry components under different assumed population numbers (K = 2 – 7) and subgroup assignment (G1 – G6) at K = 6; (b) Unrooted phylogenetic tree: subgroups (G1 – G6) and hulled/naked grain distribution; (c) Combined plot of Admixture analysis (K = 6) and neighbor-joining (NJ) phylogenetic tree (rooted with wild accessions) for 666 tested accessions; (d) Three-dimensional (3D) plot of the first three principal components from principal component analysis (PCA) of 666 oat germplasms based on 127,563 SNP markers (Abbreviations: AFR, Africa; AM, Americas; ASIA, Asia; AUS, Australia; CHN, China; EUR, Europe; MED, Mediterranean region); (e) Linkage disequilibrium (LD) half-decay analysis of hulled and naked oats; (f – h) Comparative analysis of nucleotide diversity (π) among different oat groups ($P < 0.05$) (Abbreviations: WEU, Western Europe; EEU, Eastern Europe; WAS, West Asia; EAS, East Asia; NAM, North America; SAM, South America; LRs, landraces; ELs, elite

cultivars; HL, hulled landraces; NL, naked landraces; HE, hulled elites; NE, naked elites); (i, j) Analysis of genetic differentiation among different oat groups based on fixation index (F_{st}); (k) Phylogenetic topology and gene flow direction of G1-G6 subgroups with eight fitted migration events.

(2) In the revised Results section, "Domestication pathway of naked oats", we first conducted population structure and inter-subpopulation gene flow analyses on 366 hulled and naked oat landraces with preserved ancient genetic variation, as well as seven wild hexaploid oat accessions. Our results reveal germplasm admixture and ancient genetic exchange between hulled and naked landraces, indicating that naked oats did not originate independently from cultivated hulled oats. Combined with patterns of genetic diversity and interpopulation differentiation, we further infer that hulled oat landraces from central-eastern Eurasia serve as the direct ancestral donor for Chinese naked oats. Subsequently, we excluded accessions with single ancestry proportions below 0.8, and demographic history simulations using fastsimcoal2 further corroborated these evolutionary conclusions. The relevant revisions are as follows: [L173–L238]

"The domestication origin of naked oats has long remained controversial regarding whether it originated independently from cultivated hulled oats. To minimize confounding gene flow from modern breeding and resolve this controversy, we selected 366 landraces with conserved ancient genetic backgrounds, together with seven wild hexaploid oats, for population evolutionary and gene flow analyses. Population structure analysis partitioned the 373 oats into 5 subpopulations (Figs. 2a – c and S9a; Tab. S4.1): three hulled oat-dominated subpopulations (GI–GIII), corresponding to the Northeast African and West Asian group (GI: 45 accessions, including 7 wild accessions), the Mediterranean Basin group (GII: 77 accessions), and the Central and Eastern Eurasian group (GIII: 110 accessions); a core naked oat subpopulation (GIV), where over 74.0% of Chinese naked oats form a single isolated clade; and a mixed subpopulation (GV) consisting of 63 admixed hulled and naked oats from multiple geographic origins. Although most Chinese naked oats formed a distinct subpopulation, 26.0% of accessions showed genetic admixture with other subpopulations and geographically diverse hulled oats (Tab. S4.1). Gene flow analysis revealed 7.5%–16.8% shared haplotypes across subpopulations and significant multidirectional introgression in most pairwise comparisons ($D > 0.45$, $Z > 9$, $P < 0.001$) (Figs. 2d – f and S9b; Tab. S4.2). Substantial inter-subpopulation gene flow persisted between hulled and naked oat landraces after removing all modern cultivars. These findings reveal that naked oats have not established complete reproductive isolation from hulled oat landraces, and consequently fail to support the hypothesis of fully independent domestication for naked oats.

We next explored patterns of genetic differentiation and diversity among subpopulations to delineate the ancestral hulled oat lineages and the immediate donor lineages of Chinese naked oats. Genetic differentiation analysis identified the Mediterranean Basin group (GII) as the most divergent (F_{st} = 0.35 – 0.40) and genetically diverse ($\pi = 2.75\times10^{-6}$), suggesting it is the ancestral diversity center retaining ancient landrace variation (Figs. 2g, 2h; Tab. S4.2). In contrast, extensive gene flow ($D = 0.45$, $Z = 9.3$, $P < 0.001$), minimal differentiation ($F_{st} = 0.10$), and the highest proportion of shared long ROH fragments (1–3 Mb; 44.7%) occur between the Central and Eastern Eurasian group (GIII) and Chinese naked oats (GIV) (Figs. 2d – h; Tab. S4.2). Phylogenetic analysis based on absolute genetic distance (D_{xy}) further quantified

these relationships: GIV shows the smallest D_{xy} with GIII (0.231), confirming their close genetic affinity, whereas GII exhibits the largest D_{xy} with all other groups (0.279–0.305), consistent with its early divergence (Fig. 2i). These findings position GIII as the most direct genetic source for GIV.

To validate the inferred evolutionary relationships and quantify divergence times, we performed demographic modeling using fastsimcoal2. We pre-filtered accessions within GII, GIII, and GIV with ancestral component contributions below 0.8 to mitigate confounding from recent introgression. The no-migration model was rejected across all pairwise comparisons, verifying extensive post-divergence gene flow between these lineages (Tab. S4.3). The optimal three-population model confirmed that GIV is a recent derivative of the GIII lineage (Figs. 2j, 2k; Tab. S4.3): it diverged from the GIII ancestral pool ~6124 generations ago, underwent an early isolation period of 1184 generations (~1200 generations), and initiated secondary population contact ~4940 generations ago. Subsequently, GIV underwent demographic expansion at 1772 generations ago, while GIII initiated its expansion at 1572 generations ago, with renewed prominent genetic exchange during their recent expansion phases. During early divergence, GIV experienced an extreme founder bottleneck ($N_e \approx 187$), with strong genetic drift accelerating the fixation of unique naked grain traits. This expansion is consistent with the rapid adaptation and diffusion typical of derived lineages post-domestication. GII's N_e (969) was markedly lower than the model-predicted value (1,860), while GIII's Ne (3,625) exceeded expectations (1,572) and GIV's Ne (1,338) closely matched the predicted value (1,772). Integrating archaeological and historical records, the evolutionary chronology of the GIII and GIV lineages aligns well with the eastward spread of West Asian cereal agriculture into North China; additionally, local naked oat landraces in North China exhibit the highest levels of nucleotide diversity and selection pressure among all Chinese naked oat germplasms (Figs. S9c, S9d; Tab. S4.4), and these lines of evidence support North China as a secondary domestication center for naked oats. Synthesizing all population genetic and demographic evidence, we propose a stepwise domestication pathway for oats: Hulled oats underwent primary domestication from wild hexaploid species in the Mediterranean Basin, which subsequently dispersed southeastward to East Africa and eastward to Central and Eastern Eurasia. During this eastward dispersal, naked oats diverged from the Central and Eastern Eurasian hulled oat lineage (GIII) ~6124 generations ago, followed by secondary domestication and rapid expansion in North China."

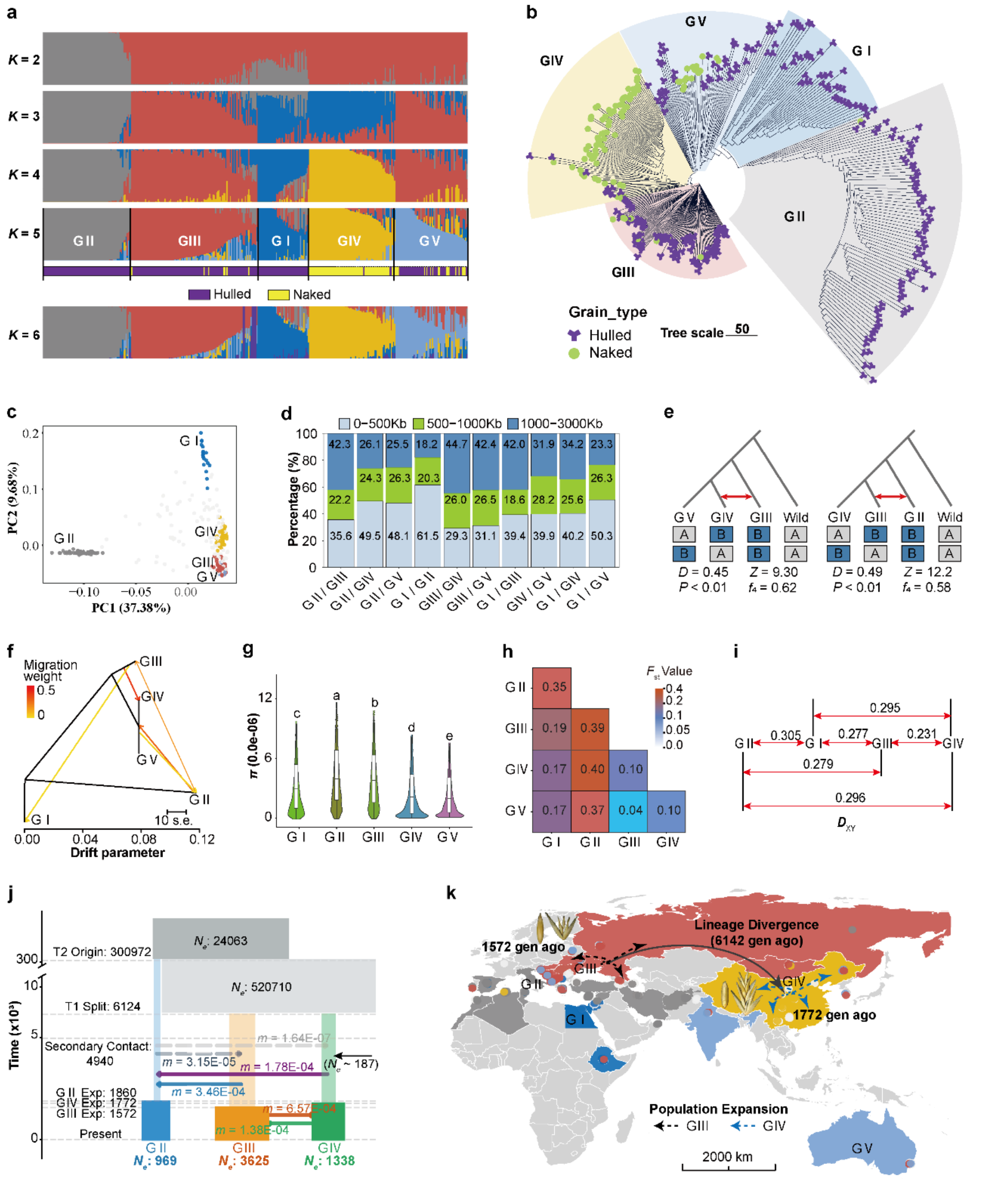

Fig. 2 Analysis of the domestication pathway of naked oats based on 366 landraces and 7 wild accessions of hexaploid oat.

(a) Genetic ancestry components of 366 landraces and 7 wild oats under different assumed population numbers (K = 2 – 6), and subgroup assignment results at K = 5; (b) Unrooted phylogenetic tree of 366 oat landraces: composition and distribution of each subgroup (GI-GV); (c) Population clustering of 366 landraces and 7 wild oats based on the PC1 and PC2 (Gray dots represent admixed accessions with ancestry proportion < 0.8. This definition also applies to Fig. 2k); (d) Proportions of shared homozygous haplotypes of different lengths (10 kb – 3 Mb) among subgroups; (e) ABBA-BABA test revealing significant gene flow between GII and GIII-GIV subgroups, and between GIII and GIV-GV subgroups; (f) Phylogenetic topology and genetic flow direction among clustered subgroups with 5 fitted migration events; (g) Nucleotide diversity levels among different subgroups ($P < 0.05$); (h) Fixation index (F_{st}) values among different subgroups; (i) D_{xy}-based phylogenetic tree showing genetic diversity differentiation among GI – GIV subgroups; (j) Demographic history reconstruction of GII, GIII, and GIV using fastsimcoal2; (k) Geographic origin distribution of accessions in each subgroup (GI – GV), as well as a schematic diagram of the divergence time and recent population expansion between the core subgroup of Chinese naked oats (GIV) and its ancestral subgroup GIII (hulled oats in Central-Eastern Eurasia).

3. We have thoroughly proofread the abstract word by word, corrected all spelling mistakes, grammatical inconsistencies, tense errors and imprecise expressions, and improved the overall accuracy and clarity of the revised manuscript. The relevant revisions are as follows: [L24–L40]

"Oats are valuable for global food and forage security due to their excellent nutrition and polyploidy. Cultivated oats (*Avena sativa* L.) are categorized into hulled and naked types based on lemma morphology, and naked oats possess superior processing and nutritional quality. Here, we deciphered the genetic architecture of 666 globally collected oat accessions via population structure, genetic diversity and gene flow analyses, revealing genetic admixture and lineage differentiation driven by geographic isolation and divergent selection. Population evolutionary analysis indicated that Chinese naked oat landraces diverged from the Central and Eastern Eurasian hulled oats 6,124 generations ago and underwent secondary domestication in North China under strong artificial selection. Multi-environment phenotyping of 19 traits from 2010 to 2021 revealed that hulled oats outperform in grain size and yield, while naked oats have higher crude protein (+25.5%) and crude fat (+16.9%). Genome-wide scans identified 883 directional selection genes related to nutrient metabolism, whereas balancing selection on carbon metabolism genes restricts yield potential. Furthermore, integrated GWAS identified three favorable haplotypes associated with favorable agronomic and quality traits, providing key genetic resources for oat molecular breeding."

Major Comment:

1. L115 it is not clear why you refer to figure 1f in this first paragraph.

RESPONSE: We appreciate your careful scrutiny. Figure 1f (Fig. R1e above; also Revised Manuscript Fig. 1e) in the original manuscript displays the distribution of nucleotide diversity (π) among subpopulations, and is unrelated to ABBA-BABA tests, gene flow estimation or interpopulation differentiation analyses. We apologise for the erroneous citation of this panel.

In the revised manuscript, we have removed this incorrect reference to Figure 1f. We have also systematically verified all figure citations throughout the entire text to ensure that every in-text citation accurately corresponds to its associated analytical results, and eliminate such oversights.

2. L121 The different acronyms of Fig.1 and other, referring to the results of this paragraph, are not explained in the legends nor in the main text.

RESPONSE: We appreciate you pointing out the oversights in our abbreviation definitions. We sincerely apologise for these lapses during manuscript drafting and proofreading. To resolve this issue, we have systematically revised and supplemented all abbreviations across the main text and corresponding figure captions. Detailed changes are provided below:

1) Population Genetic Analyses: Minor Allele Frequency (MAF); Linkage Disequilibrium (LD); Runs of Homozygosity (ROH); Fixation Index (F_{st}); Nucleotide Diversity (π); Tajima's neutrality test statistic (Tajima's D); Principal Component Analysis (PCA); Neighbor-Joining (NJ).

2) Geographic Regions: Mediterranean Basin (MED); America (AM); North America (NAM); Europe (EUR); East Asia (EAS); West Asia (WAS); Africa (AFR); Western Europe (WEU); Eastern Europe (EEU); China (CHN); Australia (AUS); South America (SAM).

3) Oat Germplasm Types: Hulled oat landraces (HL); Naked oat landraces (NL); Hulled oat elites (HE); Naked oat elites (NE).

3. L124 Why did you choose best K=6, in this case?

RESPONSE: We appreciate your careful scrutiny. We determined the optimal K-value for Admixture clustering by integrating cross-validation (CV) error profiles, biological interpretability, and NJ phylogenetic clustering results.

Our analyses demonstrate that CV errors decrease continuously with increasing K-values without a distinct inflection point (Fig. R3 below; also Revised Manuscript Fig. S2). Notably, CV errors decrease substantially from $K = 5$ to $K = 6$, indicating that the model's explanatory power for population structure continues to improve. In contrast, beyond $K = 6$, the decline in CV errors plateaus, and further increasing K-values would partition biologically irrelevant subgroups, leading to model overfitting. Cross-validation based on germplasm types, geographic origins, and NJ unrooted tree clustering reveals that at $K = 6$, the Admixture ancestry component assignments align highly with the genetic backgrounds, germplasm characteristics, and geographic origins of the accessions. The six subgroups have clear primary source materials and their clustering results are highly consistent with the NJ tree topology. Conversely, at $K = 5$, multiple genetically and phenotypically divergent groups are merged together, failing to effectively distinguish the core germplasm clusters (Fig. R4 below; also Revised Manuscript Figs 1b, 1c). Collectively, by integrating the CV error trend, biological interpretability, and independent clustering validation results, we finally determined $K = 6$ as the optimal parameter for population stratification analysis of the 666 accessions.

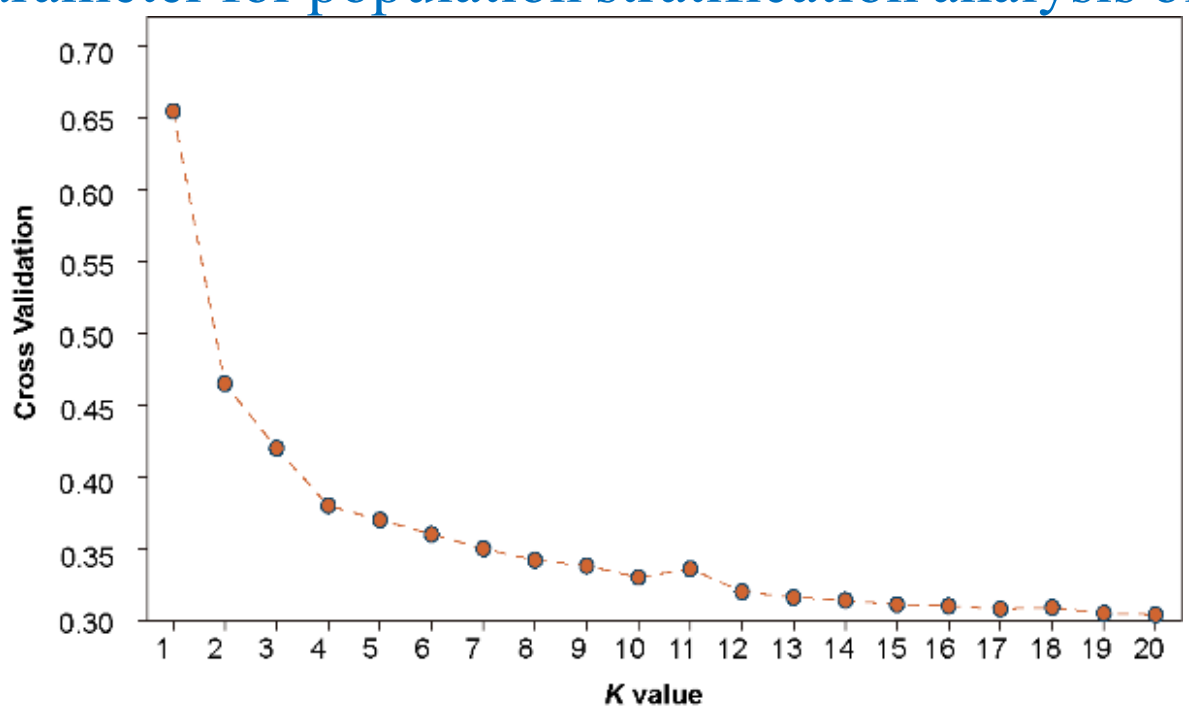

Fig. R3 Admixture cross-validation (CV) error plot for 666 oats (K = 2 – 20) (Revised Manuscript Fig. S2).

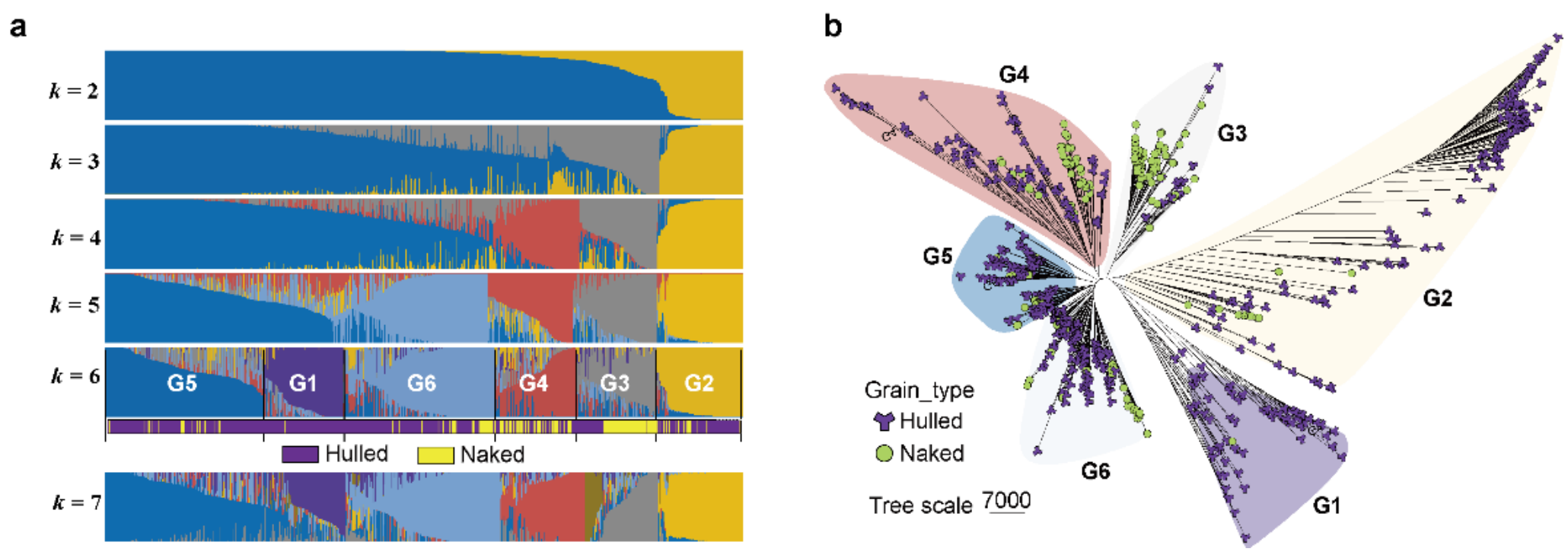

Fig. R4 Population structure analysis of 666 hexaploid oat accessions using 127,563 SNPs (Revised Manuscript Figs. 1b and c).

(a) Genetic ancestry components under different assumed population numbers (K = 2-7) and subgroup assignment (G1-G6) at K = 6 (Revised Manuscript Fig. 1b); (b) Unrooted phylogenetic tree: subgroups (G1-G6) and hulled/naked grain distribution (Revised Manuscript Fig. 1c).

4. L128-157 This section is really hard to follow, a reference to the figures showing the results reported for the different groups is needed (e.g. Fst, nucleotide diversity and Tajima's D). Moreover, form Fig. 1b it seems that the "G" groups are not properly assigned on the basis of the membership to the admixture groups (as it seems from the colors in the plot), comprising both pure and admixed individuals.

RESPONSE: We appreciate your careful comments regarding the obscure and disorganised content in Lines 128–157, the suboptimal layout of the NJ phylogenetic tree and Admixture bar plots in Figure 1b, and non-standard figure citations in the original manuscript. We have comprehensively addressed all of these issues in the revised manuscript.

Firstly, we have optimised the arrangement of the NJ tree and Admixture components in Figure 1b to eliminate visual confusion caused by the original display (Fig. R4 above; also Revised Manuscript Figs. 1b, 1c). Secondly, we have restructured the corresponding Results section to streamline logical flow, supplemented and standardised in-text citations for all population genetic analyses, and corrected irregularities in figure annotations across the entire manuscript (Fig. R1 above; also Revised Manuscript Fig. 1). The relevant revisions are as follows: [L108–L132]

"Based on 127,563 bi-allelic SNPs filtered with minor allele frequency (MAF) ≥ 0.05 and missing rate ≤ 0.1 (Fig. S1), we characterized the global population structure and genetic diversity of 666 oat accessions from 65 countries and regions (Fig. 1a; Tab. S1.1). By integrating Admixture cross-validation ($K = 1$–20) with neighbor-joining (NJ) phylogenetic analysis using a threshold of > 0.5 for ancestry proportion, we divided these accessions into six subgroups (G1–G6) (Figs 1b, 1c and Fig. S2; Tab. S1.2). While accessions of each subpopulation exhibit distinct geographic origins, prevalent genetic admixture between hulled and naked oats and cross-regional gene flow exists, with no strict geographic-genetic correspondence. Specifically, G1 represents the population convergence of West Asia and Africa, comprising 57 hulled oats predominantly from Ethiopia and Egypt, 7 wild relatives, and one Chinese naked landrace from Hebei. G2 constitutes a hulled oat landrace core subpopulation from the Mediterranean (57 accessions) and West Asia (34 accessions). G3 is a subpopulation dominated by Chinese naked oats, which account for 79.2% of its accessions. G4 represents trans-Pacific gene flow, consisting of 43 North American hulled oats and 41 Chinese naked oats from northern domestication hubs. G5 is a Sino-Eurasian mosaic comprising 53 European hulled oat elites and 13 Chinese naked oat landraces from the Shanxi-Hebei-Inner Mongolia region. G6 is a typical geographically adjacent admixed subpopulation, comprising 54 Mediterranean hulled oat landraces, 28 European hulled oats, and 55 Asian accessions (including 28 Chinese naked oats). Principal component analysis (PCA) supports this population structure and widespread genetic admixture: only G2 forms independent clustering on PC1, and G3 (the core naked oat subpopulation) clusters exclusively on PC3; extensive grain-type and geographic overlap further confirms pervasive global germplasm admixture (Fig. 1d; Tab. S1.3)."

5. L159 How have you chosen these 366 lines?

RESPONSE: We appreciate your insightful comments. Regarding the selection of the 366 oat landraces, we provide the following explanation.

We chose landraces for population evolutionary and gene flow analyses primarily because they retain relatively ancient genetic backgrounds. This allows us to eliminate genetic interference caused by modern breeding, so that we can accurately trace the evolutionary history of oats. All 666 accessions in our study have complete and well-documented germplasm information, including collection locality, hulled/naked

phenotype, and germplasm category (Revised Supplementary Tab. S1.2). From the entire panel, we extracted 366 accessions explicitly identified as landraces for subsequent analyses.

6. L162 It is not clear why K=5 is now used instead of K=6. Also, in Fig 2a the color of GV is wrong in the legend.

RESPONSE: We appreciate your careful scrutiny. First, we have corrected the colour annotation error of group GV in the legend of Fig 2a (Fig. R6a below; also Revised Manuscript Fig. 2a). Regarding the difference in selected *K* values, we clarify that the two settings originate from independent datasets and separate analyses, and are not contradictory. For the global population structure analysis covering all 666 accessions, *K* = 6 was identified as the optimal parameter according to CV error patterns, biological interpretability and NJ clustering validation.

In contrast, the analysis discussed here was performed on an independent subset of 366 oat landraces and 7 wild oat accessions. For this subset, combined with its CV error trend, NJ clustering results and geographic germplasm information, *K* = 5 produced the most reasonable and well-resolved population stratification (Figs. R5 and R6 below; also Revised Manuscript Figs. S8 and 2a, 2b). We therefore adopted *K* = 5 as the optimal parameter for this specific analysis.

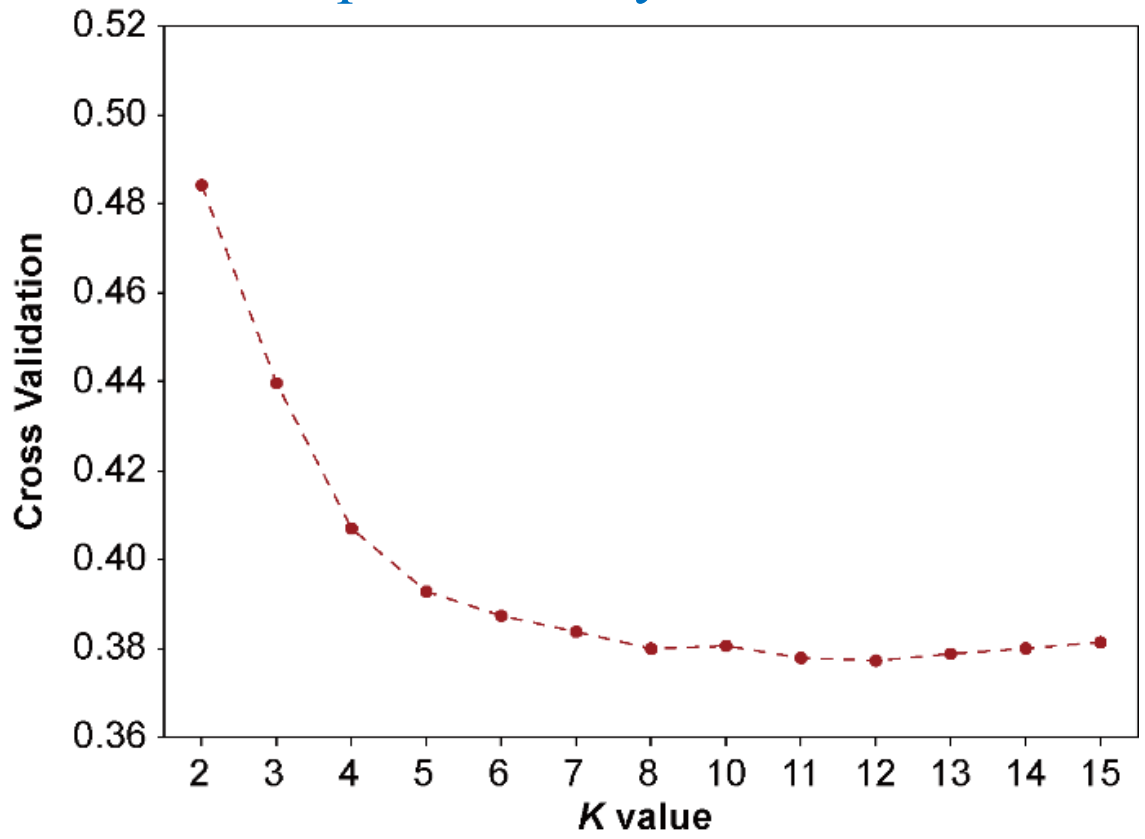

Fig. R5 Admixture cross-validation (CV) error plot for 366 landraces and 7 wild accessions of hexaploid oat (K = 2 – 15) (Revised Manuscript Fig. S8).

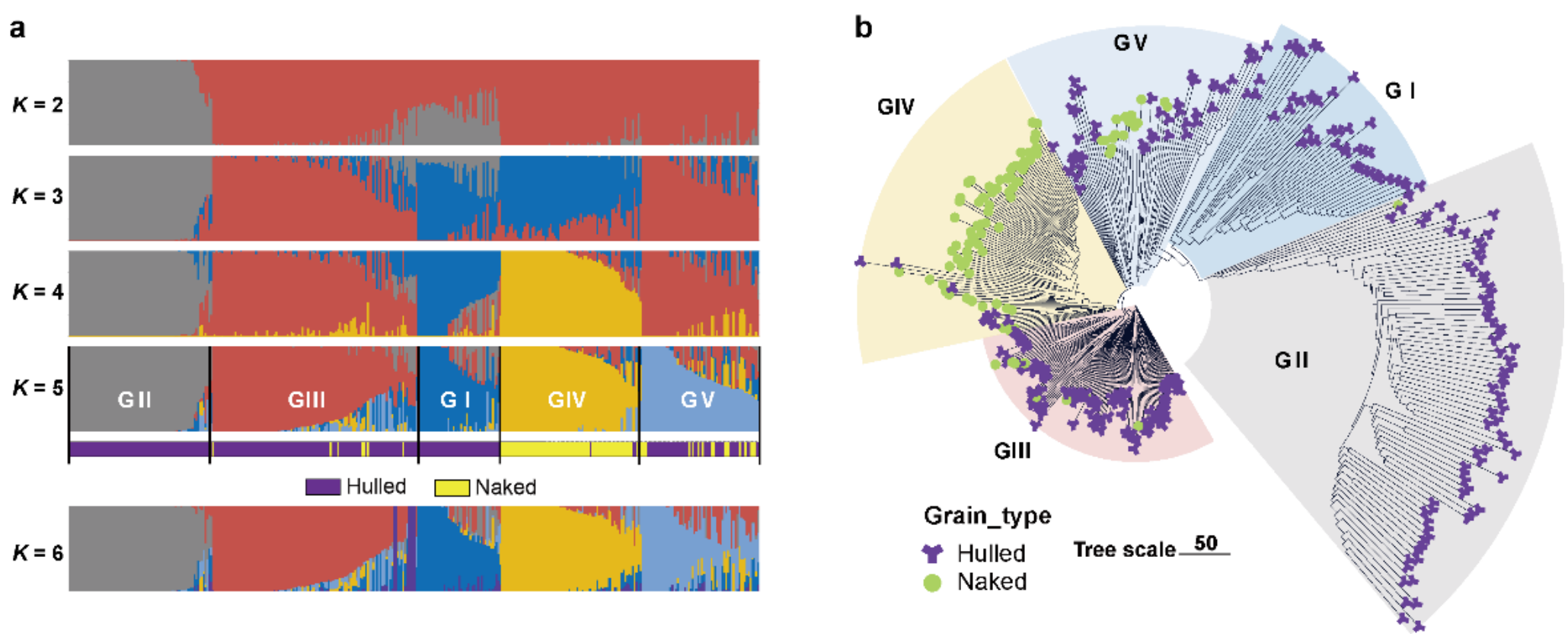

Fig. R6 Population structure analysis of 366 landraces and 7 wild accessions of hexaploid oat (Revised Manuscript Fig. 2a, 2b).

(a) Genetic ancestry components of 366 oat landraces and 7 wild accessions under different assumed population numbers (*K* = 2-6), and subgroup assignment results at *K* = 5 (Revised Manuscript Fig. 2a); (b) Unrooted phylogenetic tree of 366 oat landraces and 7 wild accessions: composition and distribution of each subgroup (GI-GV) (Revised Manuscript Fig. 2b).

7. L165 From the plot, the GIV yellow group is composed by hulled oats, not naked.
RESPONSE: We appreciate your highlighting this crucial issue. The germplasm annotation of the yellow subgroup GIV was erroneous in the original figure, as this subgroup is composed of naked oats instead of hulled oats. Accordingly, we have comprehensively revised the manuscript by rearranging the sample order in Admixture component plots, redrawing the NJ phylogenetic tree of landrace accessions, and standardising sample assignment and subgroup classification (Fig. R6 above; also Revised Manuscript Figs. 2a, 2b). In addition, we have uniformly revised the hulled/naked trait labels for all subgroups to ensure accurate matching between subgroup divisions and germplasm attributes.

8. L174 Fig 2 h-j are referred but not described.
RESPONSE: We appreciate your comment that Figs 2h – 2j were cited without corresponding elaboration in our original manuscript. Specifically, Fig 2h (Fig. R2f above; also Revised Manuscript Fig. 2f) shows inter-subgroup gene flow among oat landraces identified by TreeMix analysis; Fig 2i (Fig. R2j above; also Revised Manuscript Fig. 2j) reflects genetic relatedness among subgroups based on D_{xy} values; Fig 2j (Fig. R2j above; also Revised Manuscript Fig. 2j) depicts the demographic history and gene flow events among GII, GIII and GIV reconstructed via fastsimcoal2 modelling. These results were intended to support the conclusion that continuous gene flow and close genetic relationships exist between hulled and naked oat landraces. We apologise for the ambiguous descriptions and inappropriate citations in the original text, which caused confusion for readers.

In the revised manuscript, we have rearranged the layout of subpanels in Fig 2, restructured the relevant content logic, standardised all figure citations, and clarified the correspondence between textual descriptions and illustrations. Further details can be found in section 2.(2) of the above response to your first comment. (also L173 – 238 of the Revised Manuscript).

9. L182 The Fst values do not match the figure.
RESPONSE: We appreciate you pointing out the inconsistencies between textual statistics and graphical data, which stemmed from oversights during data validation.
In the revised manuscript, we have cross-checked all figures and corrected all F_{st} estimates in the main text to ensure complete consistency with the presented data (Fig. R7 below; also Revised Manuscript Fig. 2h). Furthermore, we have systematically verified the consistency of all genetic parameters, including F_{st}, nucleotide diversity (π) and D_{xy}, across the main text, figures and supplementary materials. All data discrepancies have been fully rectified to prevent such errors from recurring. The relevant revisions are as follows: [L197–L200]

"Genetic differentiation analysis identifies the Mediterranean Basin group (GII) as the most divergent (F_{st} = 0.35 – 0.40) and genetically diverse (π = 2.75×10^{-6}), suggesting it is the ancestral diversity center retaining ancient landrace variation (Figs. 2g, 2h; Tab. S4.2)."

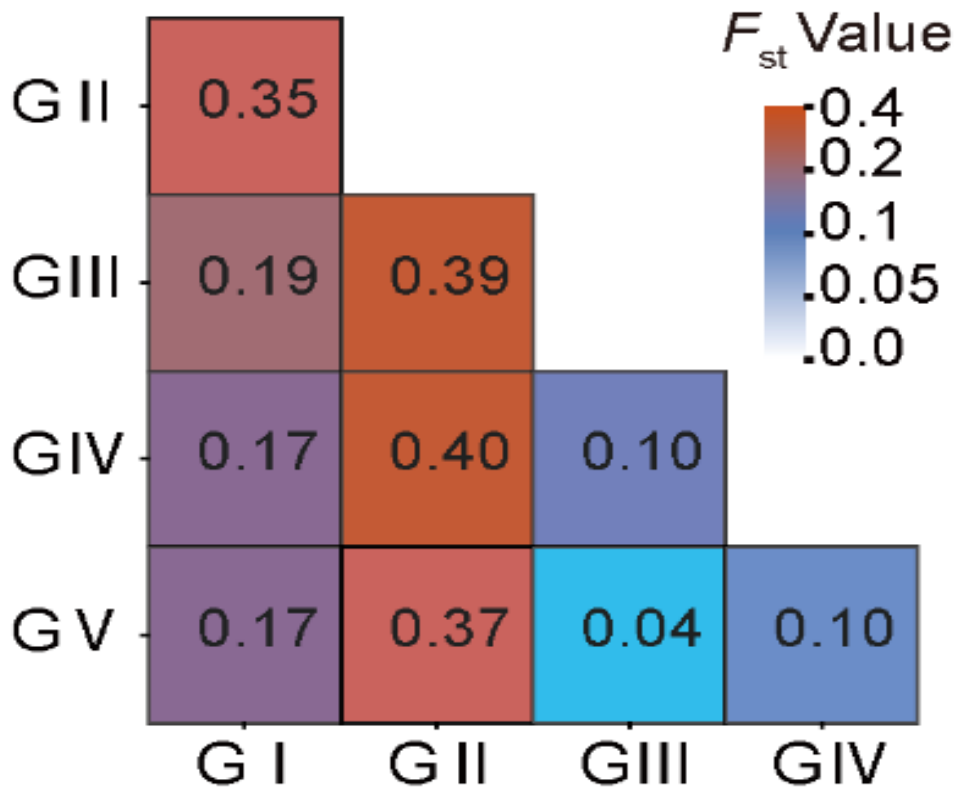

Fig. R7 Fixation index (F_{st}) values among subgroups of GI–GV (Revised Manuscript Fig. 2h)

10. L213-219 A table here would be useful to follow these results.

RESPONSE: We appreciate your valuable suggestion. The summary table of genetic model and heritability results for the 19 agronomic traits has been fully provided in the supplementary materials (Tab. R1 below; also Revised Supplementary Tab. S5.1). Due to limitations on page length and the quota of figures and tables in the main manuscript, we cannot incorporate this supplementary table into the main text. In the revised manuscript, we have improved the in-text citation to guide readers to access all detailed results in the supplementary materials conveniently.

Tab R1 Analysis of genotype-by-environment interaction and broad-sense heritability (H^2) for 19 phenotypic traits. (Revised Supplementary Tab. S5.1)

Trait	Best_Model	Var_Line	Var_Year	Var_Loc	H^2	LRT_P	BIC
H/N	Model 1	0.18	0	0	0.96	0.03	-18184.4
AWN	Model 2	1.11	0	0	0.78	0.0001	4822.54
ST	Model 2	0.63	0.01	0	0.95	0.0001	3812.9
SLT	Model 1	0.18	0	0	0.95	0.0001	-5345.56
PH	Model 3	239.71	40.46	625.05	0.41	4.3E-195	31936.24
PL	Model 3	47.61	0	8.43	0.81	4.7E-206	19009.41
HS	Model 3	63.79	42.21	64.2	0.53	7.1E-162	23778.96
GP	Model 3	70.33	46.54	70.77	0.53	7.1E-162	24082.63
LFL	Model 3	8.18	0	15.71	0.55	4.91E-46	12224.3
WLF	Model 3	0.07	0.12	0.02	0.56	1.11E-61	2367.57
ET	Model 3	18.64	3.4	0	0.62	2.29E-36	31179.7
NN	Model 3	0.37	0	0	0.80	8.4E-115	8329.62
SN	Model 3	164.84	24.04	0	0.71	2.29E-86	25354.43
GN	Model 3	1846.1	698.95	949.94	0.78	6.69E-41	11937.03
TGW	Model 3	17.38	1.49	2.48	0.75	8.8E-48	9431.61
GL	Model 2	0.59	0.31	0	0.79	0.587424	8786.27
GW	Model 2	0.07	0.01	0	0.9	1.9E-203	-317.13
CPC	Model 3	0.02	0	0	0.75	0.000123	1398.82
CFC	Model 2	0	0.01	0	0.69	0.00001	-3206.07

11. L221 What do you mean by "genetic correlation"?

RESPONSE: We appreciate your careful scrutiny. Genetic correlation originates from intrinsic genetic factors, including pleiotropy, linkage disequilibrium and shared regulatory pathways. It reflects the inherent genetic associations between traits and effectively excludes environmental confounding effects.

Based on 666 accessions, whole-genome SNP markers and multi-environment phenotypic data of 19 agronomic and quality traits, we conducted bivariate GREML analysis using GCTA software (v1.93.2). We constructed the genetic relationship matrix (GRM) from genome-wide SNPs, estimated additive genetic variances and covariances for pairwise traits, and calculated genetic correlation coefficients to quantify genetically driven associations among traits (See Lines L586–L592 in the revised manuscript). We have also supplemented relevant explanations in the Results section to clearly distinguish genetic correlation from phenotypic correlation (See Lines L248–L254 in the revised manuscript). The relevant revisions are as follows:

L586 - L592: "Meanwhile, based on the genotypic and phenotypic data of the test materials, the genome-based restricted maximum likelihood (GREML) bivariate model in GCTA software [50] was used to construct a genetic relationship matrix (GRM) with genome-wide SNP data, simultaneously estimate the additive genetic variances and covariances for pairwise traits, and calculate the genetic correlations, which reflects the degree of phenotypic correlation between traits attributable to shared genetic factors."

L248 - L254: "Using whole-genome SNP markers, genetic correlation analysis among the 19 traits implemented in GCTA (v1.93.2) revealed significant negative correlations between yield-related traits (TGW, GL, GW) and quality traits (CFC, CPC) ($r = –0.22$ to $–0.34$, $P < 0.05$). Hulled traits correlated positively with yield traits ($r = 0.28–0.49$, $P < 0.01$), while naked traits correlated positively with quality traits ($r = 0.53–0.67$, $P < 0.01$), reflecting domestication-driven phenotypic specialization (Fig. S9; Tab. S5.2)."

12. L228 There is no Fig.3

RESPONSE: We greatly appreciate your careful detection of this error. In the original manuscript, Figure 3 was erroneously numbered as Figure 2. This figure numbering mistake has been fully corrected. Systematic cross-checks of all figure numbering throughout the manuscript have been performed to ensure complete consistency between textual content and figure identifiers. We sincerely apologise for any inconvenience caused during your review.

13. L239-257 PCA plots would be useful to follow these results.

RESPONSE: We greatly appreciate your valuable suggestion. Incorporating the phenotypic principal component analysis (PCA) plot can intuitively demonstrate the population phenotypic differentiation pattern, and further corroborate the systematic association between phenotypic PCA and molecular marker PCA in our study.

In response to your comment, we have supplemented a phenotypic PCA scatter plot in the phenotypic differentiation section of the main manuscript (Fig. R8 below; also Revised Manuscript Fig. S11). Based on 19 phenotypic traits, the plot shows the distribution of accessions along PC1 vs PC2 and PC1 vs PC3 axes. Six genetically clustered subgroups are labelled with distinct colours, clearly presenting the phenotypic distribution of germplasms with different genetic backgrounds.

As observed from the results, G2 (Mediterranean hulled landraces) and G3 (core Chinese naked oat accessions) are distinctly separated, which is highly consistent with molecular-based population structure analysis (Fig. R9 below; also Revised Manuscript

Fig. 1d). In addition, G4 (North American hulled oat cultivars) is interspersed between the above two groups. Combined with analyses of inter-subgroup gene flow and phenotypic variation (Fig. R10 below; also Revised Manuscript Fig. S12), this result further supports our conclusion that North American hulled oats serve as a germplasm exchange bridge during modern breeding, which has pyramided multiple elite genetic foundations and endowed the germplasms with high yield and broad adaptability.

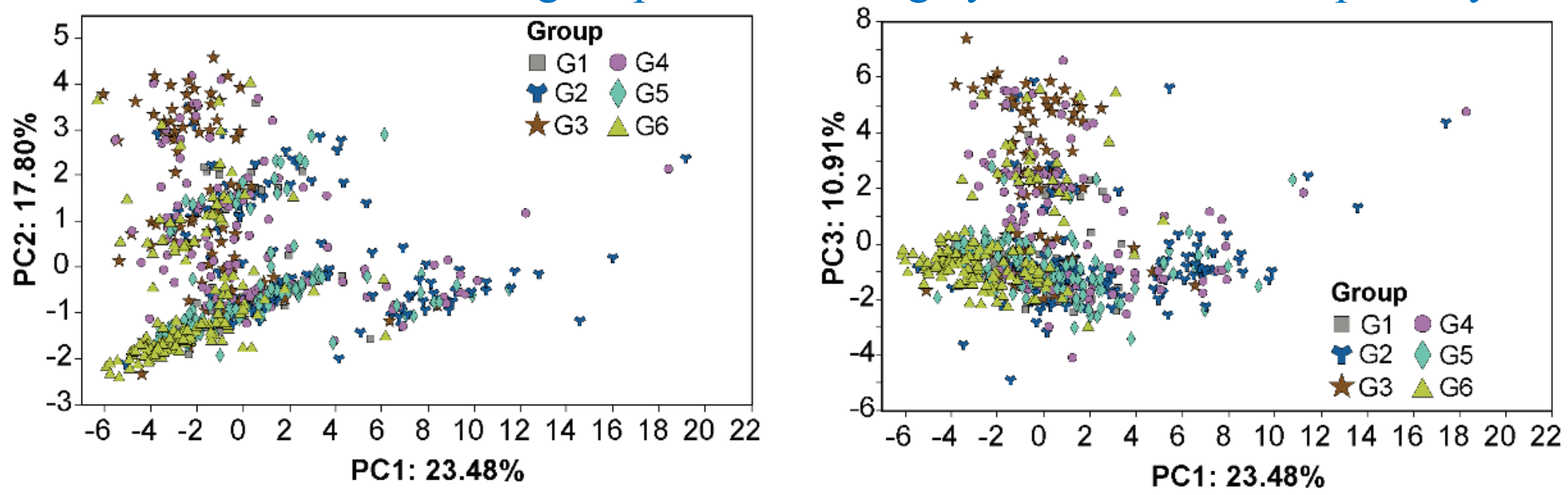

Fig. R8 Score plot of the first three principal components from PCA based on 19 phenotypic traits in 666 oat accessions (Revised Manuscript Fig. S11).

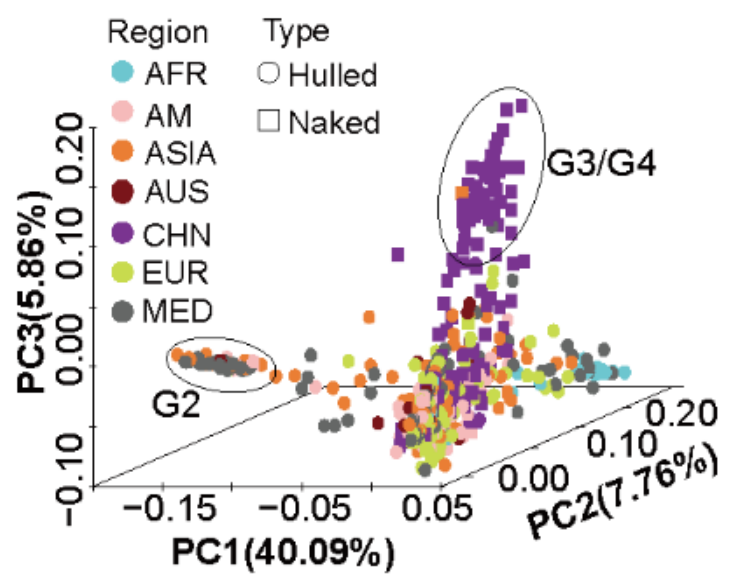

Fig. R9 Three-dimensional (3D) plot of the first three principal components from principal component analysis (PCA) of 666 oat germplasms based on 127,563 SNP markers (Revised Manuscript Fig. 1d).

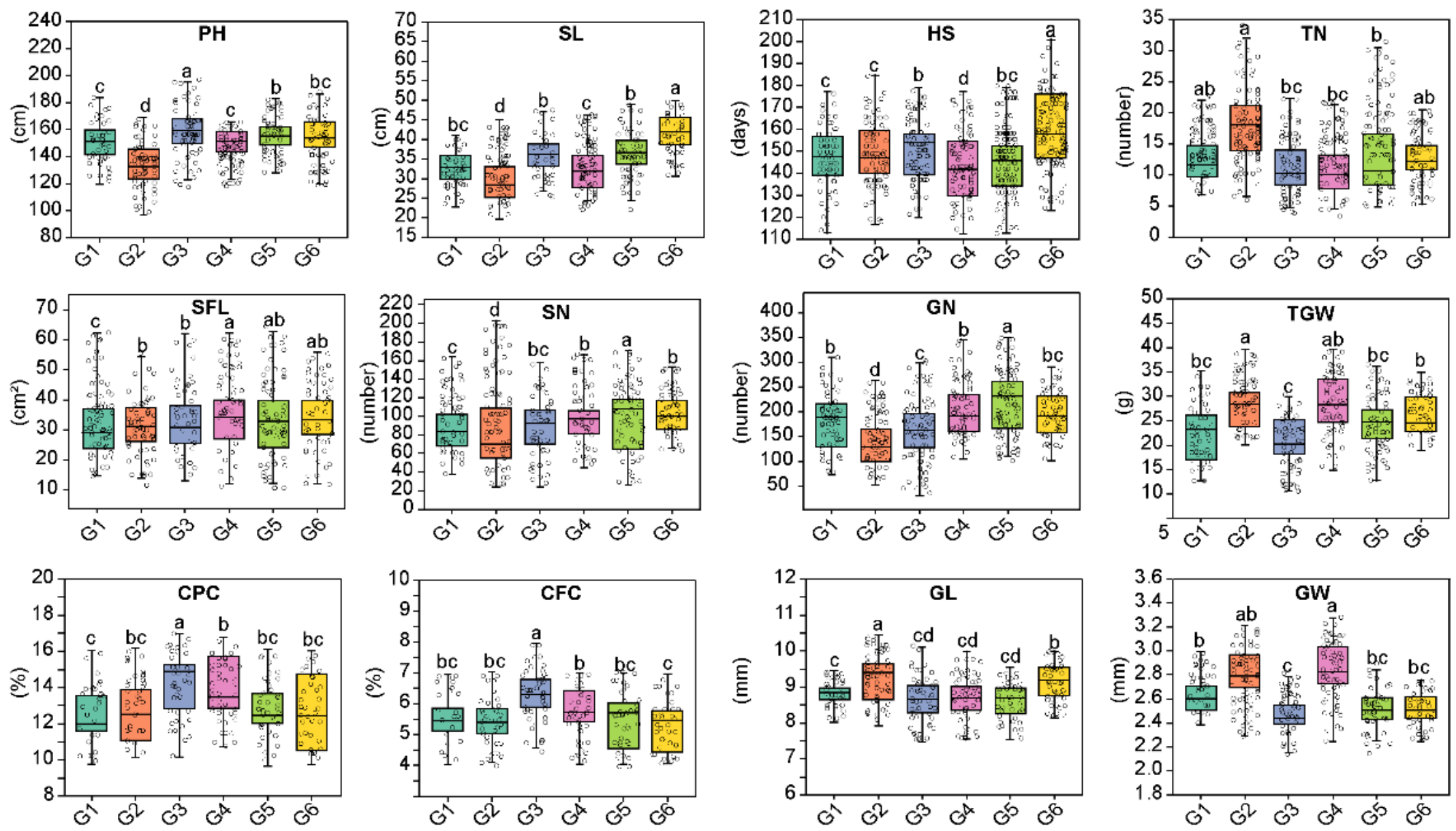

Fig. R10 Comparative analysis of key traits across genetic subgroups. (Box plots showing differences in key traits among the genetic subgroups) (Revised Manuscript Fig. S12).

We sincerely thank both reviewers for their professional and constructive comments and for recognizing the value of our study. We have carefully considered and fully addressed all the comments and suggestions raised by Reviewers 2 and 3, and have thoroughly revised the manuscript accordingly. A point-by-point response is provided below. We have made every effort to resolve all remaining concerns through revisions and detailed explanations. We believe these modifications have significantly improved the overall quality and academic rigor of the manuscript.

Reviewers' Comments:

Reviewer #2 (Remarks to the Author):

The work is novel and of broad interest to the community, particularly because it advances our understanding of naked oat domestication and provides valuable genomic resources and analytical frameworks that will likely facilitate future research. Overall, I believe this study represents a significant contribution to the field and will be of interest to researchers working in related areas. The findings are likely to stimulate further investigations and may influence future studies in oat genomics, evolution, and trait improvement. I appreciate the authors' careful revisions and responses. I fully support publication of this manuscript in its current form.

RESPONSE: We would like to express our sincere gratitude to Reviewer #2 for the positive and insightful comments, as well as for the strong endorsement of our work. We highly appreciate your recognition of the novelty and broad relevance of our study, which advances our understanding of naked oat domestication and provides valuable genomic resources and analytical frameworks to support future research on oat genomics, evolutionary analysis, and agronomic trait improvement. We are truly thankful for your approval of our revisions and your full support for the publication of this manuscript.

Reviewer #3 (Remarks to the Author):

The revised manuscript has clearly improved, and the authors have addressed the comments raised in my previous review. Overall, I am satisfied with the revisions, and I do not have any further comments, except for a careful check of the figure references and captions throughout the manuscript. In particular, Figure 1a-c seems to be mislabeled, as the captions of panels a-c do not match the corresponding plots.

RESPONSE: We greatly appreciate your positive assessment of our revised manuscript. We sincerely thank you for your careful reminder to thoroughly check all figure citations, labels and legends throughout the paper, and particularly for pointing out the mismatch between the subplots and their captions in Figure 1a–c. Your valuable suggestions are crucial for enhancing the overall rigor and standardization of our manuscript. We apologize for the careless oversights in our original version. In the revised manuscript, we have carefully reviewed all in-text figure citations, panel labels and legend descriptions throughout the full text. We have specifically corrected the mismatched content of Figure 1a–c and standardized the consistency between all figures and their legends in the manuscript. Detailed revisions are described as follows:

1). We have fixed the legend misalignment of panels a–c in original Figure 1a–c (Fig. R1 below; Revised Manuscript Figs. 1a–c).

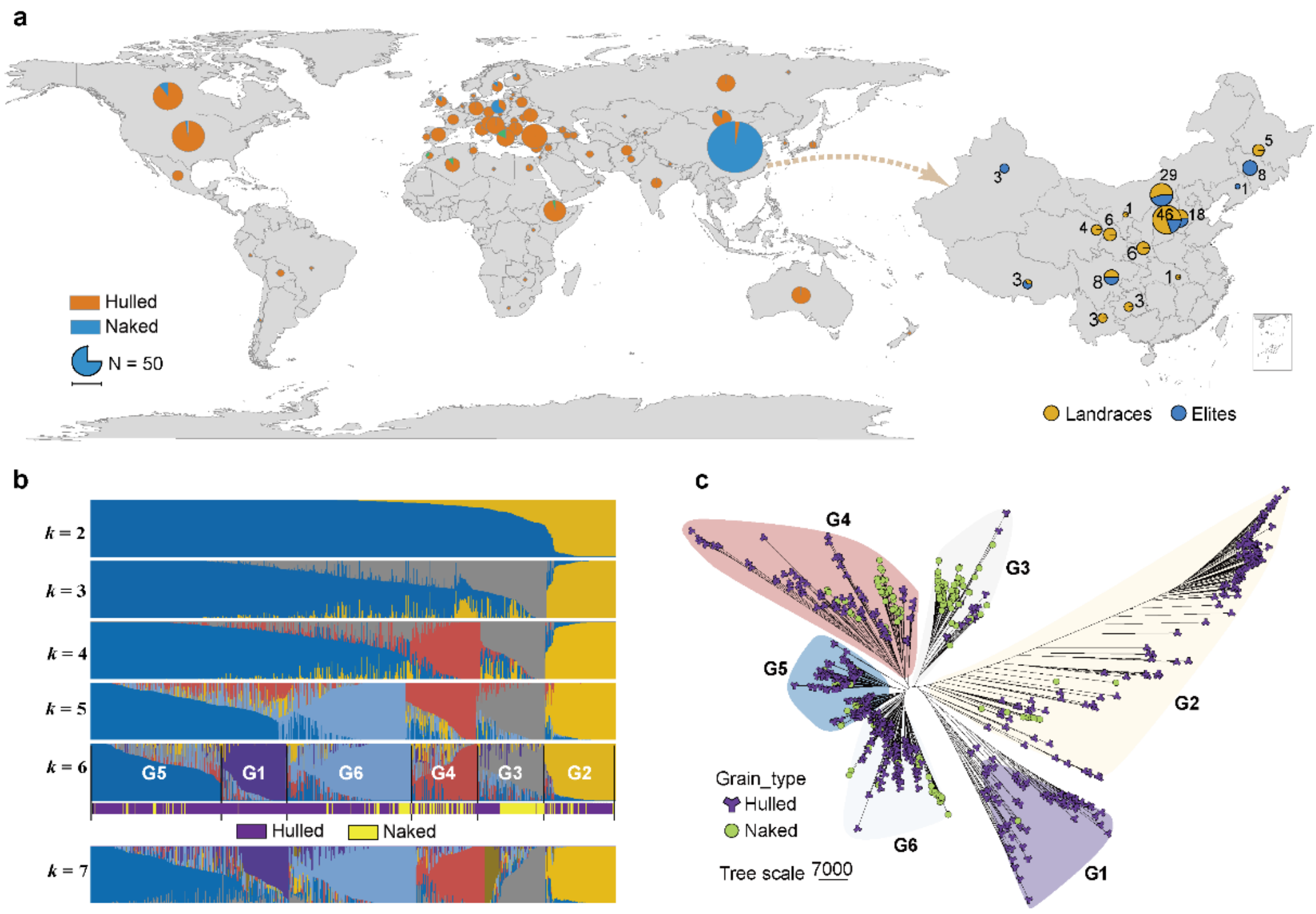

Fig. R1 Population structure analysis of 666 hexaploid oat accessions using 127,563 SNPs (Revised Manuscrip Figs. 1a－c).

(a) Global distribution of 666 tested accessions and distribution map of 162 Chinese naked oat accessions (Revised Manuscript Fig. 1a); (b) Genetic ancestry components under different assumed population numbers (*K* = 2–7) and subgroup assignment (G1–G6) at *K* = 6 (Revised Manuscript Fig. 1b); (c) Unrooted phylogenetic tree: subgroups (G1–G6) and hulled/naked grain distribution (Revised Manuscript Fig. 1c).

2). We have fixed the incorrect labeling of the sample size of the tested materials in the legend of original Figure 2b (Fig. R2 below; Revised Manuscript Fig. 2b).

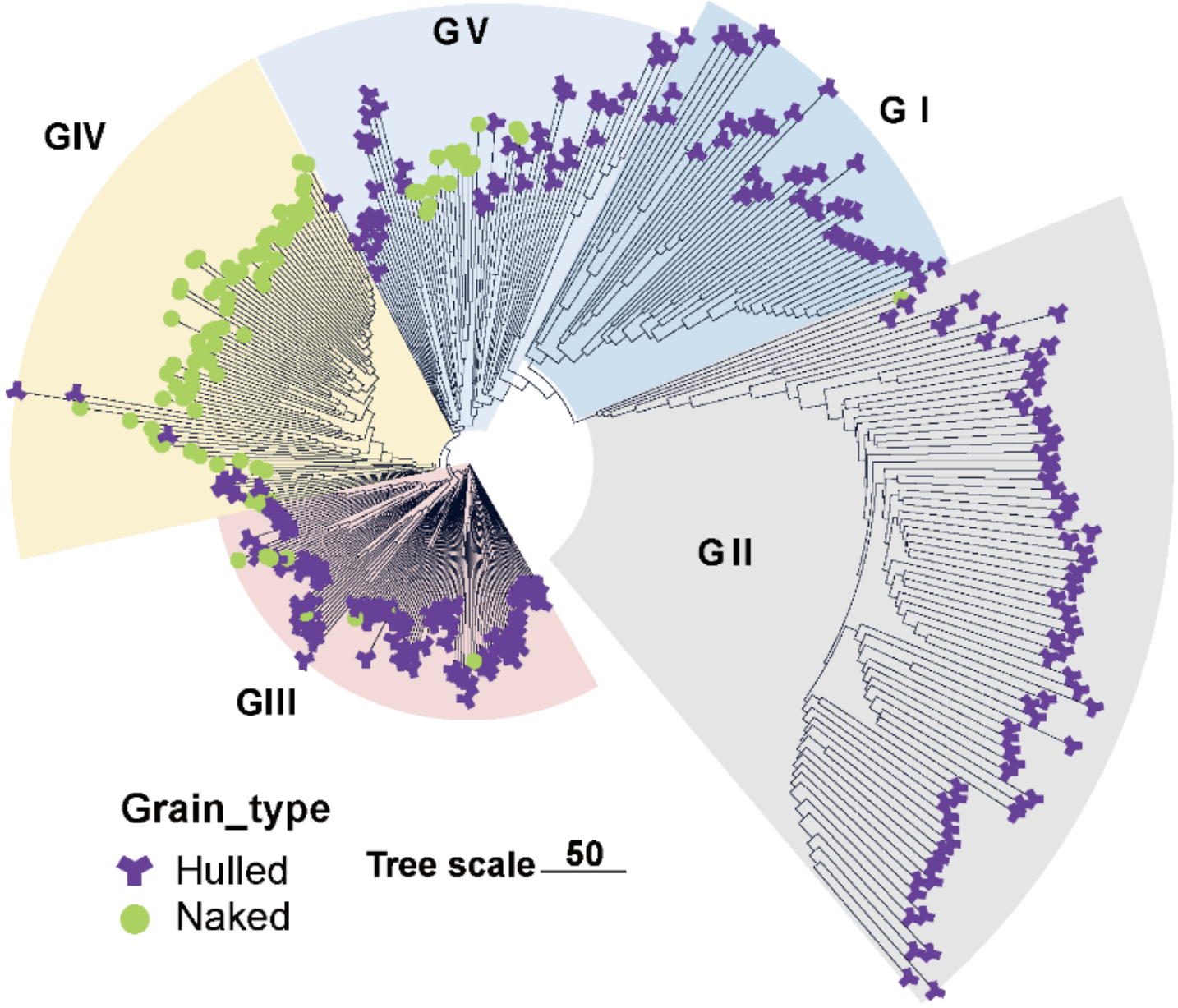

Fig. R2 Unrooted phylogenetic tree of 373 oats: composition and distribution of each subgroup (GI-GV) (Revised Manuscrip Fig. 2b).

3). We have fixed the legend misalignment of panels d–f in original Figure 5d–f (Fig. R3 below; Revised Manuscript Figs. 5d–f).

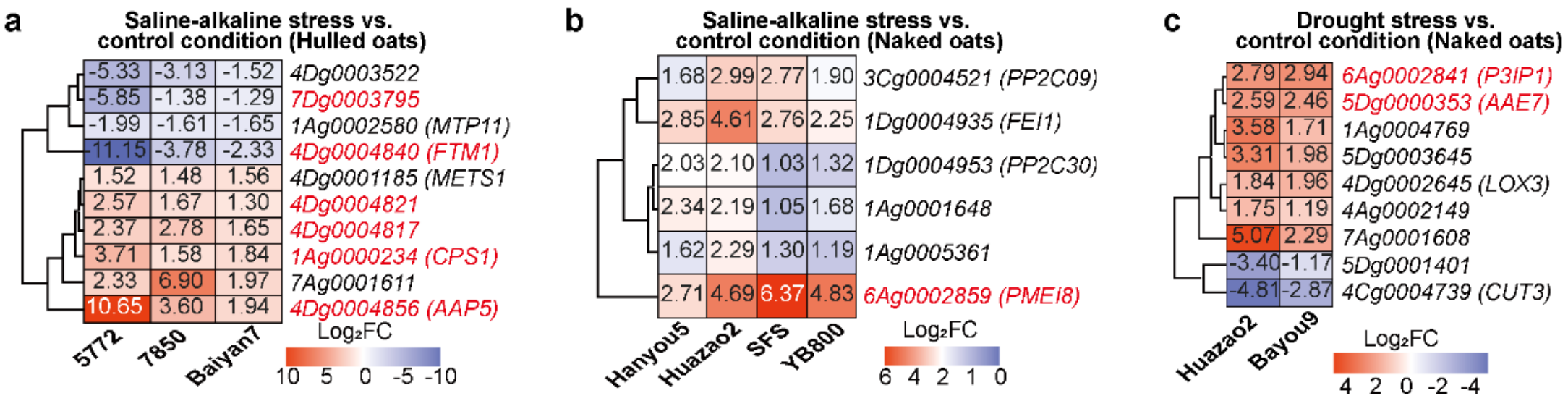

Fig. R3 Expression heatmap of selected and differentially expressed genes in naked oats based on Log2FC values (Genes marked in red are associated with previously published oat QTLs) (Revised Manuscrip Figs. 5d－f).

(a) Intersection of DEGs from saline-alkali stress transcriptome of hulled oats and selected genes in naked oats (Revised Manuscript Fig. 5d); (b) Intersection of DEGs from saline-alkali stress transcriptome of naked oats and selected genes in naked oats (Revised Manuscript Fig. 5e); (c) Intersection of DEGs from drought stress transcriptome of naked oats and selected genes in naked oats (Revised Manuscript Fig. 5f).

4). We have fixed the legend misalignment of panels a and b in original Supplementary Figs. 9a and b (Fig. R4 below; Revised Manuscript Supplementary Figs. 9a and b).

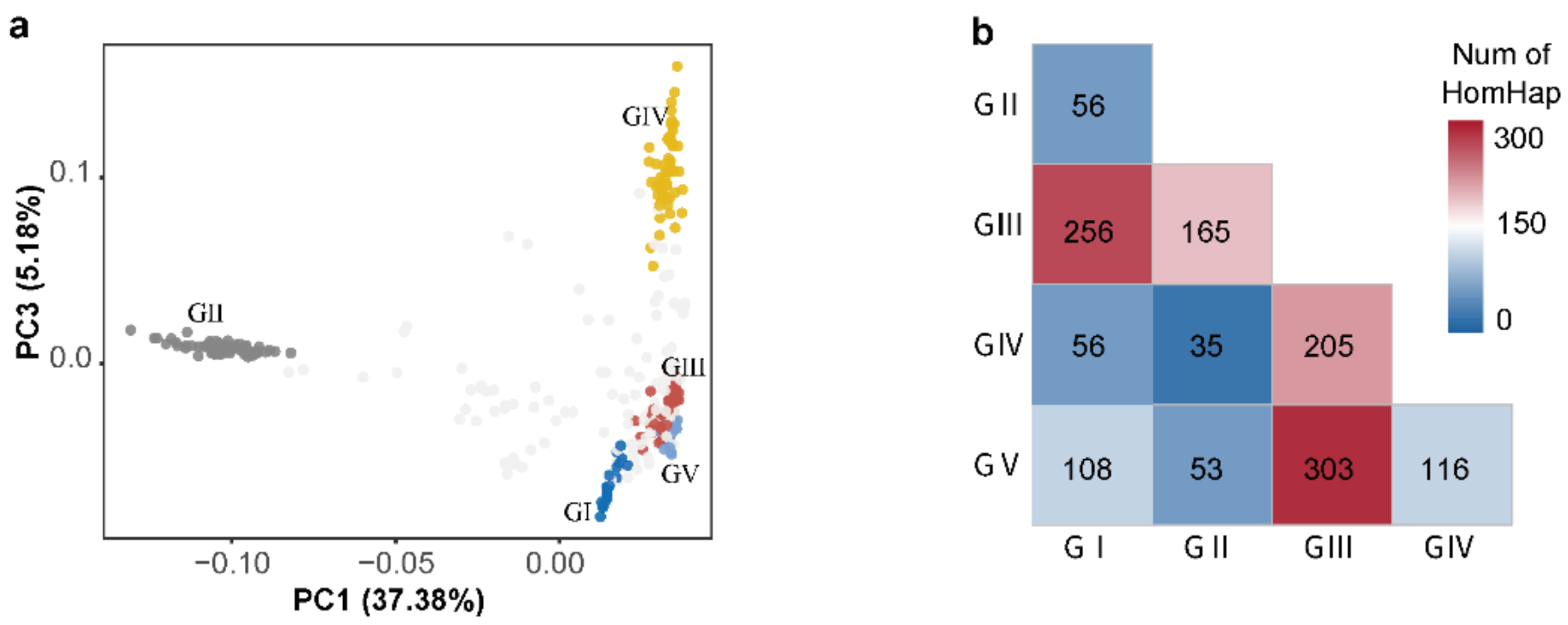

Fig. R4 PCA and shared homozygous haplotypes of 366 hexaploid oat landraces and 7 wild accessions (Revised Supplementary Figs. 9a and b).

(a) Distribution of the 366 landrace and 7 wild accessions along PCA1 and PCA3 (Revised Supplementary Fig. 9a); (b) Number of shared homozygous haplotypes (10kb–3 Mb) among subgroups (Revised Manuscript Supplementary Fig9.b).

5). We have fixed the legend mislabelling in original Supplementary Fig. 18c (Fig. R5 below; Revised Manuscript Supplementary Fig. 18c).

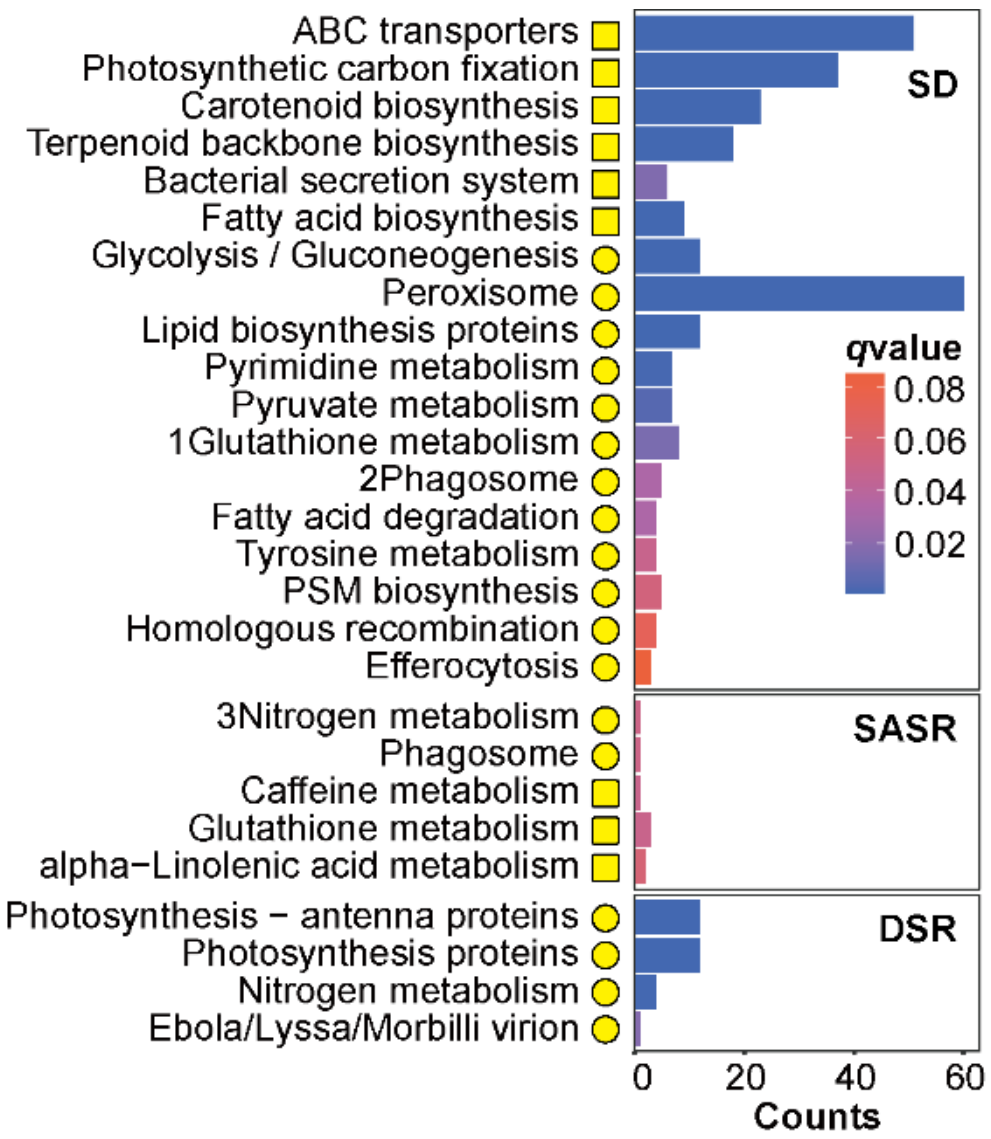

Fig. R5 KEGG enrichment for genes specifically down-regulated in hulled or naked oats under salt-alkali or drought stress (Revised Supplementary Fig. 18c).

Review

In the manuscript "Bridging the Yield and Nutritional Gap in Oat: Insights from Naked Oat (Avena sativa ssp. nuda) Domestication", the authors Dong et al. evaluate the population structure, genetic diversity, and gene flow of 666 oat accessions from 65 countries using 127,563 SNPs obtained through GBS.
The main objective of this work was to assess the genetic origin and phenotypic divergence between hulled oat (Avena sativa) and naked oat (Avena sativa var. nuda). The panel consists of 293 bred varieties (227 hulled oat [H] and 66 naked oat [N]), 366 landraces (270 H and 96 N), and 7 wild materials (H).
The study used 19 agronomic and quality traits evaluated over 11 years and found that hulled oats generally have higher yield, while naked oats exhibit higher nutritional quality (e.g., crude protein and fat content). Genome-wide scans and RNA-seq analyses identified numerous genes and markers involved in metabolite synthesis and abiotic stress resistance in naked oats.

Major Comments

The authors fail to clearly explain, for readers not familiar with oat, the difference between hulled oat (A. sativa) and naked oat (A. sativa var. nuda). A comparison table summarizing the populations used throughout the manuscript is necessary. It is unclear whether the main goal was to compare hulled versus naked oats, or to evaluate differences among countries or improvement levels (wild materials, landraces, and cultivars).

Throughout the manuscript, numerical values for Fst and genetic diversity are missing. The authors instead use vague terms such as â€œweak genetic differentiationâ€ or â€œlow genetic diversity.â€ These quantitative results should be explicitly reported in tables.

Overall, the figures are difficult to interpret and appear unnecessarily complex. For instance, the Pearson correlation in Fig. S6a is unclear, and the Circos plotsâ€”especially those for Fst (Fig. 1f, Fig. S4b, and Fig. S4e)â€”are hard to follow. Fst values and other intra/inter-genetic metrics should be summarized in a table.

I have not access any supplementary tables, and many of the results described in the manuscript are missing. All supplementary materials should be deposited in Figshare or a similar public repository.

1. Population Structure

In the PCA (Fig. 1d), there is a clear separation of a cluster along PC1, but the authors do not provide any information about this group of accessions.

L116: What statistical test supports the claim of breeding introgression in Gâ…¥, which includes 13 Chinese naked oats and 61 hulled cultivars from Oceania and North America?

2. Domestication Pathway

Why did the authors not use the classical definition of gene flow, N_m = (1 â€“ Fst)/(4 Ã— Fst)?

L135: The authors mention â€œsubgroup divergence chronology shows Mediterranean hulled groups (G2/G3),â€ but do not provide collection or development dates for all accessions. What evidence supports this statement?

Of the 19 evaluated traits, 17 are agronomic and only two are quality traits (crude protein and crude fat content). Therefore, it is difficult to draw strong conclusions regarding quality.

L693: I suggest replacing Fig. S6b with a table showing the eigenvectors (Fig. 3d), since the current table is difficult to interpret and fails to capture genetic variance effectively.

3. Phenotypic Analysis

There is no multi-environment genetic modeling or other analyses that elucidate how divergent selection drives yieldâ€“quality trade-offs or environmental adaptation between hulled and naked oats.

The authors do not include analyses of broad-sense heritability or genetic correlations among traits.

L688: Agronomic traits should be grouped and discussed by yield and quality.

L222: â€œThe results suggest that naked oats and hulled oats have developed distinct stress response traits through directional selection in their respective cultivation environments.â€ However, no stress conditions were tested, and the functional enrichment analysis is insufficient to support this conclusion.

L180: Why did the authors not compare improvement levels or countries directly, rather than relying solely on clustering? Groups GIâ€“GX were generated from a Neighbor-Joining (NJ) analysis based on 127,563 markers, including both neutral and non-neutral variants, and each cluster contains mixed accession types.
What is the LD decay for the full dataset and for each population (naked vs. hulled)?
How was the threshold for Fst and Pi ratio chosen in Figure 4a?

4. Genome-Wide Scanning and RNA-seq

L187: Why did the authors perform a genome-wide selective sweep analysis rather than a genome-wide association study (GWAS)? Selection scans are generally more informative in advanced populations after several selection cycles where Fst changes within specific genomic regions.

L227: The RNA-seq analysis of 22 genetically and geographically diverse accessions is interesting, but why were tissues (root, stem, leaf, spike) pooled across different developmental stages? Using pooled tissues increases background noise and makes it difficult to identify differentially expressed genes (DEGs). The 22 accessions could have been sequenced separately with replicates instead.

If the goal was to identify DEGs in spikes and relate them to oat type, only spike tissue should have been used.

L247: It is unclear how the transcriptomic findings align with the genome-wide selection scans. How many associated markers were overlaping with DEG? and how to correlate the role of the 19 traits with the Genome-Wide Scanning and RNA-seq analysis?

L270: The rationale for using both KASP markers and qPCR is unclear. Typically, qPCR is used to validate DEGs.

Minor Comments

L100: Replace â€œAncestral component analysisâ€ with â€œADMIXTURE analysis.â€

Fig. 1 L664: â€œAncestral componentâ€ refers to a structure analysis with multiple K. Include sample origin in the PCA (e.g., AFR for Africa). Please maintain consistent geographic abbreviations across figuresâ€”e.g., Fig. S5 uses â€œCHNâ€ for China and â€œAFâ€ for Africa, but other figures use different labels.
Panels (c) and (d) could be replotted using an interactive 3D Plotly visualization. Panel (f) (Circos plot) could be replaced with a table or matrix for easier comparison.

L686: â€œSpike type (ST)â€ and â€œSpikelet type (SLT)â€ are mislabeled in Fig. 3b. The figure also lacks axis labels.

L161: The morphological differences between hulled and naked oats (e.g., multiflowered spikelets and long awns) are not clearly visible in Fig. 3b
